# Target-agnostic SAR mapping and immunological evaluation of (−)-FR252921 and analogs against primary human immune cells

**DOI:** 10.1039/d5sc09554a

**Published:** 2026-04-27

**Authors:** Iakovos Saridakis, Manuel Schupp, Haoqi Zhang, Thomas Leischner, Laura Marie Gail, Konstantin Günther, Martina Drescher, Florian Doubek, Daniel Kaiser, Georg Stary, Nuno Maulide

**Affiliations:** a Institute of Organic Chemistry, University of Vienna Währinger Straße 38 1090 Vienna Austria nuno.maulide@univie.ac.at; b CeMM Research Center for Molecular Medicine of the Austrian Academy of Sciences Lazarettgasse 14, AKH BT 25.3 1090 Vienna Austria gstary@cemm.oeaw.ac.at; c Vienna Doctoral School in Chemistry, University of Vienna Währinger Straße 42 1090 Vienna Austria; d Department of Dermatology, Medical University of Vienna Währinger Gürtel 18-20 1090 Vienna Austria; e Christian Doppler Laboratory for Chronic Inflammatory Skin Diseases Vienna 1090 Austria

## Abstract

The macrocyclic immunosuppressive natural product (−)-FR252921 was isolated in 2003 from a liquid culture of *Pseudomonas fluorescens*. Despite promising preliminary immunological insights, in-depth studies that advance our understanding of structure–activity relationship (SAR) have been scarce. Herein we document a detailed SAR mapping of (−)-FR252921 by the syntheses of 14 analogs and their subsequent evaluation. The composed library of analogs was designed to address crucial questions regarding the roles of explicit molecular features of the natural product while following a target-agnostic efficacy approach. Whereas previous efforts have focused on murine T cell assays, our endeavors transit to a primary human cell-based immunological platform using peripheral blood mononuclear cells (PBMCs), enabling direct assessment of immunosuppressive activity in B, natural killer (NK), and T cells *via* cytokine suppression. We emphasize that a new fully synthetic (−)-FR252921 analog (fs-FR4) profoundly outperformed the natural product in suppressing the response of both stimulated B cells (21-fold) and NK cells (16-fold) within the context the biological assays used, validating the power of our platform and standing as a lead candidate for further preclinical development. Lastly, we highlight that our studies included significant improvement in the synthetic efficacy of previously reported building blocks and key synthetic steps towards (−)-FR252921 and analogs.

## Abstract 2

Fifteen synthetic analogs of the immunosuppressant (−)-FR252921 were designed to map its SAR, resulting in the discovery of the highly potent fs-FR4. This compound showed 21-fold higher potency in suppressing cytokine production in human PBMCs.

## Graphical abstract

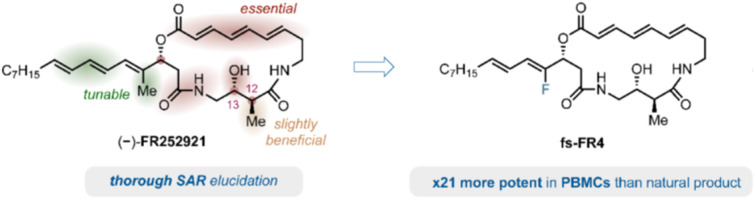

## Introduction

**Fig. 1:**
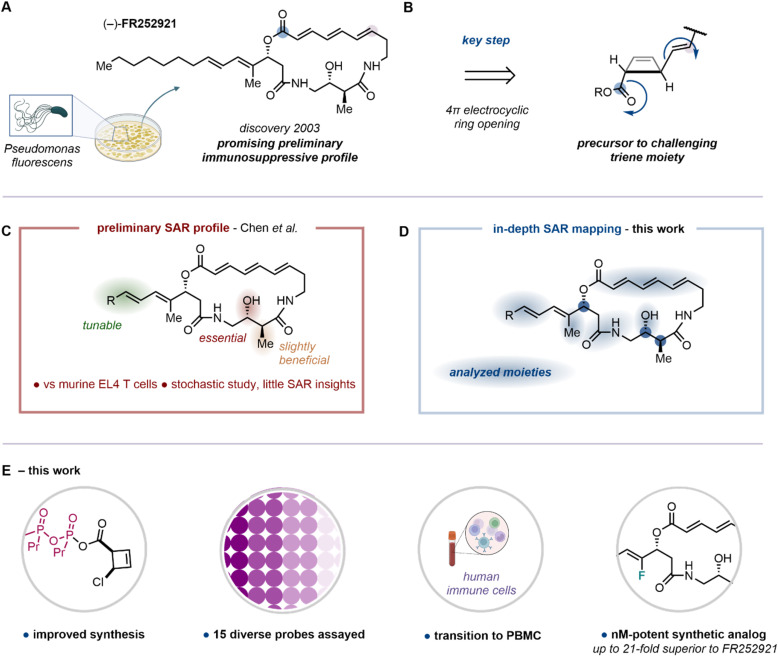
(A) Discovery of immunosuppressive natural product (–)-FR252921 by Fujisawa Pharmaceuticals. (B) Key synthetic step featuring 4π-electrocyclic ring opening as established in our previous work. (C) Preliminary structure–activity relationship (SAR) was assayed in murine T cells. (D and E) This work: in-depth, systematic SAR analysis spanning the entirety of molecular architectures of the natural product. Improved synthetic routes enhanced the efficacy and allowed the synthesis of 15 diverse probes for immunological evaluation in peripheral blood mononuclear cells (PBMC) and the development of the, thus far, most potent (–)-FR252921 derivative.

Autoimmune and organ transplantation-associated diseases have a tremendous impact on human health, with profound societal and economic consequences. Despite advances in prevention, diagnosis and treatment, a gap remains between uncovering the disease-specific immunological mechanisms and ways to specifically target them.^1^ Recently adopted therapeutic strategies include biologics and small molecule inhibitors, which are often administered in combination.^2–4^ Indeed, state-of-the-art small-molecule immunosuppressive drugs such as rapamycin (sirolimus), FK506 (tacrolimus) and CsA (cyclosporin A) have been at the frontline of immunosuppression since their introduction into the clinic in the 1980's and 1990's.^5–8^ Although these drugs have been proven powerful, their well-documented side effects eventually cause further complications, with the most common being nephrotoxicity (FK506 & CsA),^7,9^ and susceptibility to opportunistic infections (all immunosuppressants).^8^ It is therefore well-recognized that the development of novel immunosuppressive agents, with mechanisms of action distinct from the state-of-the-art drugs, bears great potential for improving patient survival and quality of life.^10^ In 2003, Fujisawa Pharmaceutical Company, from a liquid culture of *Pseudomonas fluorescens* no. 408813, isolated the macrocyclic natural product (−)-FR252921, containing a distinct architecture featuring an (*E*,*E*,*E*)-trienoic lactone within a 19-membered ring (Fig. 1A). Notably, preliminary evaluation of its inhibitory activity on the proliferation of EL4 T cells, a mouse-derived T cancer cell line, revealed potential immunosuppressive function.^11^ Further studies by the same researchers moreover pointed towards a synergistic profile of FR252921 with FK506, therefore suggesting a distinct mode of action.^12,13^ The promising preliminary profile of this natural product inspired the synthetic community and resulted in a series of total syntheses, formal syntheses, and advances towards FR252921.^14–20^ In 2019, our group reported the total synthesis of (−)-FR252921, developing an approach which relied on a torquoselective electrocyclic ring opening of a *cis*-configured cyclobutene intermediate for the stereoselective construction of the triene system of the macrocycle (Fig. 1B).^19,21–27^ Employing this unconventional macrocyclization method, the natural product along with nine non-natural analogs had been synthesized and examined with regard to their antiproliferative effect in a cell viability assay using murine EL4 T cells over 72 hours. Although this allowed preliminary insights into the structure–activity relationship (SAR) of this family of macrocycles (Fig. 1C), a more comprehensive understanding has yet to be reached. Indeed, the initial exploratory investigation only monitored moieties in a stochastic manner, delivering little understanding.

Herein, we provide a wider, detailed and systematic SAR analysis probing side chain sterics and electronics, macrocyclic rigidity, integrity and saturation, stereochemical configuration and hydrogen bonding motifs (Fig. 1D). By virtue of chemical synthesis in a purely target-agnostic manner, the designed analogs were set to answer essential questions with regard to the potential role of explicit molecular features. Notably, to provide translational relevance as a step toward therapeutic development, we transitioned from murine cells to human peripheral blood mononuclear cells (PBMCs), evaluating immunosuppressive potency by means of cytokine suppression (Fig. 1E). Moreover, our synthetic endeavors included significant improvements towards previously reported building blocks and key synthetic steps towards (−)-FR252921 and its analogs. Finally, we highlight that, among the 15 diverse macrocycles assayed, a novel synthetic analog (fs-FR4) was proven superior to the natural product (up to 21-fold) in achieving cytokine suppression.

## Results and discussion

### Analog design

**Fig. 2:**
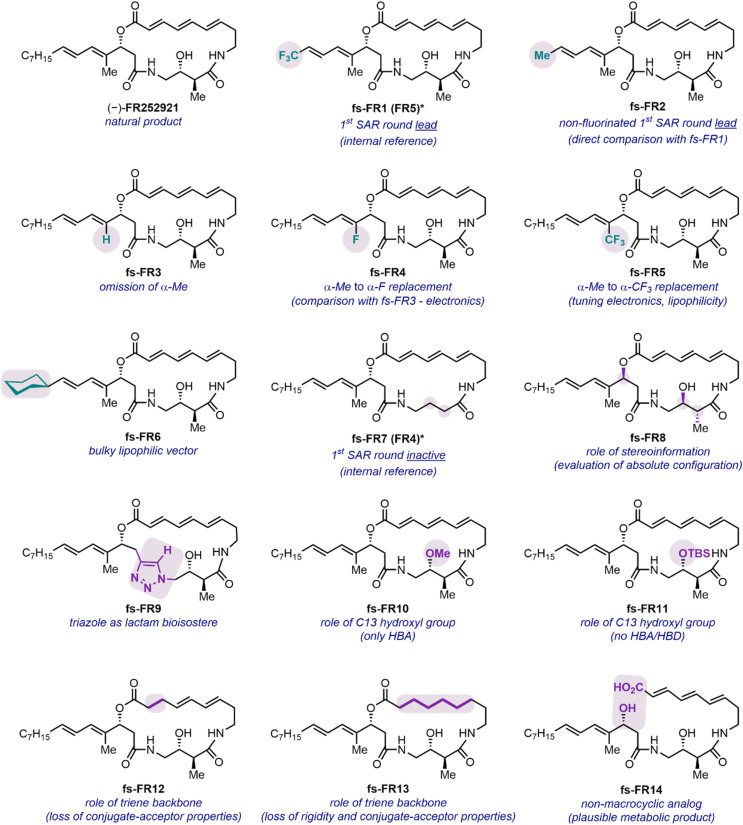
Overview of the synthesized compounds for biological evaluation against PBMC, addressing essential questions regarding the potential role of explicit molecular motifs. *fs-FR1 and fs-FR7 were first reported and termed in our prior work as FR5 and FR4, respectively, and served in this work as internal references. TBS: *tert*-butyldimethylsilyl; HBD: hydrogen bond donor; HBA: hydrogen bond acceptor.

The design of the analog library, consisting of the natural product, two previously assessed analogs and 12 new entities, was strategically orchestrated around crucial questions which would help to further decipher the SAR of the FR252921 family (Fig. 2). The synthetic platform previously established by our group offered facile tunability with regard to the side chain of the macrocycle, which was the first point of focus for the current study with different electronic and steric parameters considered.^19^ Synthetic analog fs-FR1 was previously reported to be slightly more potent against murine EL4 T cell proliferation than (−)-FR252921,^19^ raising the question whether the presence of the CF_3_-group alone, the shortened aliphatic chain, or a combination thereof was responsible for the improved activity. To address this question, its non-fluorinated analog fs-FR2, was synthesized by virtue of a modified sidechain precursor (see SI for synthetic details). The natural product as well as fs-FR1 were also synthesized to serve as internal references. The preliminary SAR profile elucidated by our prior work highlighted the importance of the aliphatic side chain, which, however, was found to be modifiable. Accordingly, we designed and synthesized four new analogs covering the spectrum of electronic and steric signatures of the sidechain (Fig. 2, see SI for synthetic details): fs-FR3 (α-H), fs-FR4 (α-F), fs-FR5 (α-CF_3_) and fs-FR6 (δ-Cy). Specifically, fs-FR3, fs-FR4 and fs-FR5 replace the α-Me of the side chain with a hydrogen atom, a fluorine atom, or a trifluoromethyl group, respectively, probing the stability of the allylic carboxylate ester, as well as subtly modifying the steric demand and electronic environment of the α-position. On the other hand, fs-FR6 introduces a bulky lipophilic vector aimed to bridge the knowledge gap between the previously synthesized analogs bearing an aromatic sidechain^19^ and the linear natural *n*-heptyl residue found in FR252921.

Considerations regarding protein binding being dependent on the absolute configuration of the small-molecule drug led to the synthesis of fs-FR8 (*ent*-FR252921). Alongside this investigation of stereochemical effects, structural variations resulting in altered ability to participate in hydrogen-bonding interactions were introduced in a range of analogs. fs-FR9 saw the replacement of the western lactam moiety with a 1,4-disubstituted triazole, in principle capable of similar hydrogen bond donor (HBD)-/hydrogen bond acceptor (HBA)-interactions as the lactam,^28^ while concomitantly enlarging the ring to a 20-membered macrocycle and introducing a rigid aromatic scaffold into the backbone of the molecule. Additionally, potential HBD-/HBA-activity of the C-13-hydroxyl group was probed. Whereas, on the one hand, the HBD-capabilities were impaired by the introduction of a methoxy group onto C-13, leading to fs-FR10, fs-FR11 probed the electronic effects of the oxygen atom connected to C-13, as the presence of a bulky TBS group shields any HBA-interactions by virtue of steric demand.

Furthermore, the role of the (*E*,*E*,*E*)-configured triene backbone of the macrocycle beseeched to be thoroughly probed. Initial considerations with regard to stereochemical stability of the FR compounds identified a possible Michael/retro-Michael process onto the α,β-double bond, leading to the strain-reduced (*Z*,*E*,*E*)-isomer of FR252921, as a likely process. Such a phenomenon was reported by both groups reporting the initial total syntheses of FR252921.^14,15^ Saturation of the α,β-double bond led to fs-FR12, and complete omission of all three core double bonds led to fs-FR13, which introduced a flexible aliphatic backbone into the macrocycle of the parent scaffold. Finally, we pondered whether the lactone of the natural product is a metabolic weak point that leads to a hydrolyzed, linear scaffold as the actual active species. To examine this question, we constructed fs-FR14 (Fig. 2).^14,15^ The previously reported and inactive fs-FR7 was also synthesized to serve as an additional, inactive internal reference.^19^

### Biological evaluation

**Fig. 3:**
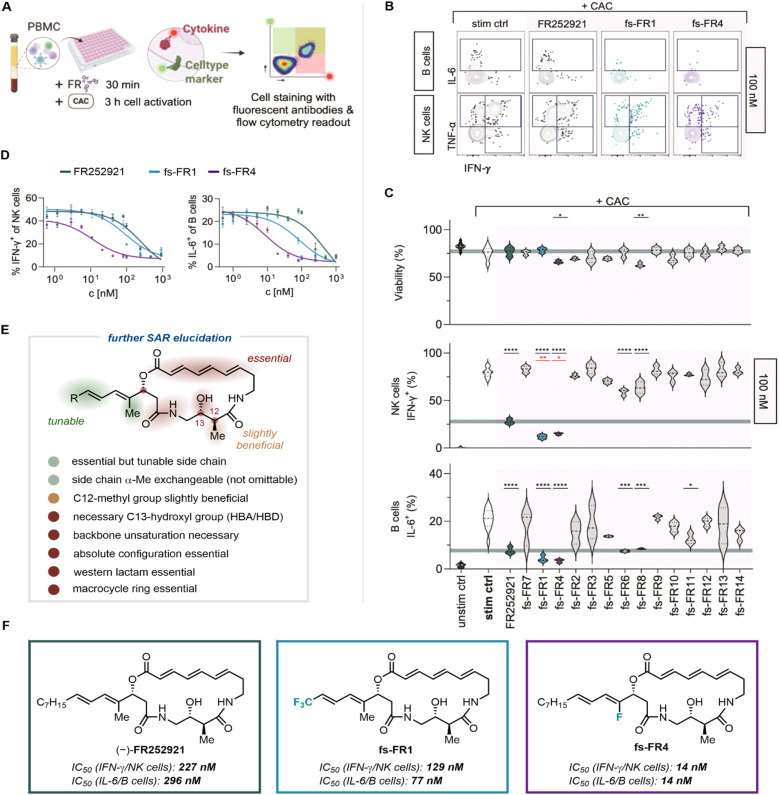
(A) Schematic of the *in vitro* assay determining inhibition of cytokine production in activated PBMC by (–)-FR252921 and its derivatives through flow cytometry. (B) Representative plots showing gating strategy for cytokine-positive B (CD20+ HLA-DR+) and NK (CD56+ HLA-DR−) cells in the indicated conditions at 100 nM; stimulated control (stim ctrl) refers to cells treated with cell activation cocktail (CAC) without incubation of FR compounds (for further detail see SI). (C) Violin plots (median ± quartiles) showing cell viability of total PBMC and cytokine expression of NK and B cells at 100 nM at indicated conditions. Significance compared to positive control (stimulated control (stim ctrl) bold) is indicated; data represent three technical replicates per condition; *p*-value * < 0.05, ** < 0.01, *** < 0.001, **** < 0.0001; ordinary one-way ANOVA with Dunnett's multiple comparisons test; dark green line indicates mean value of (–)-FR252921; red indicates significant difference of a given compound to (–)-FR252921 (for more details see the SI). (D) *XY* charts showing non-linear dose–response regression curves against normalized cytokine expression in NK and B cells for the indicated concentrations and compounds used to determine IC_50_ values (*n* = 3–6 technical replicates per concentration, combined data of two independent experiments). (E) Structural summary of the probed SAR. (F) IC_50_ results for the three most active compounds, (–)-FR252921, fs-FR1 and fs-FR4, as obtained from a sole, healthy human donor. See SI for further details. (A) was created in Biorender (Stary, G. (2026) https://BioRender.com/s8hfw94, licensced under CC BY 4.0).

Initial biological evaluation of FR252921 and the first generation of analogs was conducted against EL4 T cells, an immortalized cancer cell line derived from murine T cells, by quantifying the compounds' ability to inhibit proliferation of said cell line.^19^ While this assay is easily conducted and provides a first insight into potential application of compounds in an immunosuppressive context, it lacks the experimental depth required by the complexity of the immune system. To enable a more thorough investigation of the immunosuppressive properties of the established and novel analogs of FR252921, we developed an immunological assay based on the inhibition of activation of peripheral blood mononuclear cells (PBMC). PBMC comprise the majority of immune cell types responsible for mediating autoimmune diseases and transplant rejection and can be easily isolated from whole blood of healthy human donors. The assay is based on the ability of different types of immune cells (T cells, B cells and NK cells, among others) to produce cytokines upon stimulation through a cell activation cocktail (CAC) containing ionomycin, phorbol 12-myristate 13-acetate (PMA) and Brefeldin A. Ionomycin and PMA lead to the activation of the cells, resulting in the production of cytokines, while the addition of Brefeldin A prevents their secretion, enabling the measurement of intracellular concentrations of cytokines. In order to assess the FR analogs' immunosuppressive potential, we measured cytotoxicity and suppression of cytokine production of FR-treated, CAC-activated PBMC using flow-cytometry (Fig. 3).^29^ Specifically, we measured the production of pro-inflammatory cytokines IFN-γ, TNF-α and IL-6, which are released by immune cells such as T, NK or B cells upon activation and during inflammation. Therefore, reduced production of these cytokines is a measure of the immunosuppressive potency of a given molecule.

The panel of analogs of FR252921 (fs-FR1 to fs-FR14), as well as FR252921 itself, were evaluated against PBMC from one healthy human donor (Fig. 3A–C). Freshly isolated PBMC were incubated with FR compounds and activated using CAC. The cells were then stained with fluorescent antibodies targeting cell type markers and cytokines for flow cytometric analysis (Fig. 3A; see SI for experimental details). The selection of NK cells and B cells was based upon their characteristic expression of surface markers, such as CD56+ for NK cells and CD20+ HLA-DR+ for B cells. The most pronounced effect of the FR compounds could be observed in NK cells and B cells, as represented by the shift in stained cell population (Fig. 3B). All 15 compounds were tested at six concentrations, ranging from 10 µM to 10 nM, to assess their dose response in PBMC (with representative data at 100 nM shown in Fig. 3C).^29^

First, immune cell viability upon stimulation and incubation with the compounds was investigated to select cytotoxic compounds from immunosuppressive FR analogs. It was shown that, at 100 nM concentration, only two synthetic analogs (fs-FR4 and fs-FR8) significantly reduced viability beyond stress-induced cell death in the positive control, indicating cytotoxicity at this concentration (Fig. 3C). Further evaluation of these two cytotoxic compounds revealed that fs-FR8 does not lead to pronounced suppression of cytokine production at lower concentrations and is therefore not a competent immunosuppressant. Besides FR252921 and fs-FR1, we found significant cytokine suppression in cells treated with fs-FR4, fs-FR6, fs-FR8 and, to some extent, fs-FR11 at 100 nM. However, only fs-FR1 and fs-FR4 showed superior activity compared to FR252921. Since our goal was to improve upon the properties of FR252921, we chose to further investigate these two compounds. For fs-FR4, we observed no more cytotoxicity at and below 50 nM, whereas significant cytokine suppression was maintained. The two analogs showing higher activity than FR252921 across a range of cell types and cytokines, *i.e.*, fs-FR1 and fs-FR4 (*p* < 0.01 and *p* < 0.05 respectively against NK cells; non-significant at 100 nM against B cells; *p* < 0.001 for fs-FR4 against B cells at 10 nM; for data see SI), were further evaluated to establish their IC_50_, alongside the determination of the IC_50_ of FR252921 in primary human immune cells. As we observed the best dose response for B cell IL-6 production and NK cell IFN-γ production, we used these values to determine the IC_50_ (Fig. 3D). For FR252921, the IC_50_ with regard to the PBMCs used was established to be 227 nM for IFN-γ and 296 nM for IL-6, whereas fs-FR1 was more active, with IC_50_ values of 129 nM and 77 nM, respectively. All acquired information concerning the SAR is summarized in Fig. 3E and F. To our delight, the novel synthetic analog fs-FR4 displayed the highest activity, with an IC_50_ of 14 nM for both cytokines. To validate our findings by eliminating the possibility of a donor-specific activity of the FR molecules, we confirmed the activity of the active analogs at 100 nM and 10 nM using samples from three additional healthy human donors (Fig. S1). Additionally, FR252921 and fs-FR4 were evaluated for their cytotoxic properties against non-immune cells and were found to be non-cytotoxic against the cells used (Fig. S6).

Overall, the new insights enabled by this evaluation further elucidate the SAR of the FR252921 class of compounds (Fig. 3E): the positive impact of the necessary side chain is enhanced by the presence of fluorine atoms, possibly due to an increase in lipophilic character; the hydroxyl group is necessary and most likely participates in essential hydrogen bonding; the C12-methyl group is slightly beneficial for potency; the α-Me group at the side chain is replaceable (*e.g.*, with CF_3_) but not omittable (*i.e.*, replacement with a hydrogen atom); the unsaturated system at the backbone of the macrocycle is essential; the absolute configuration at the three stereocenters is suggested to be crucial when compared to the enantiomer of the natural product; the western lactam and the presence of the macrocycle are essential for potency.

### Chemical synthesis

**Fig. 4:**
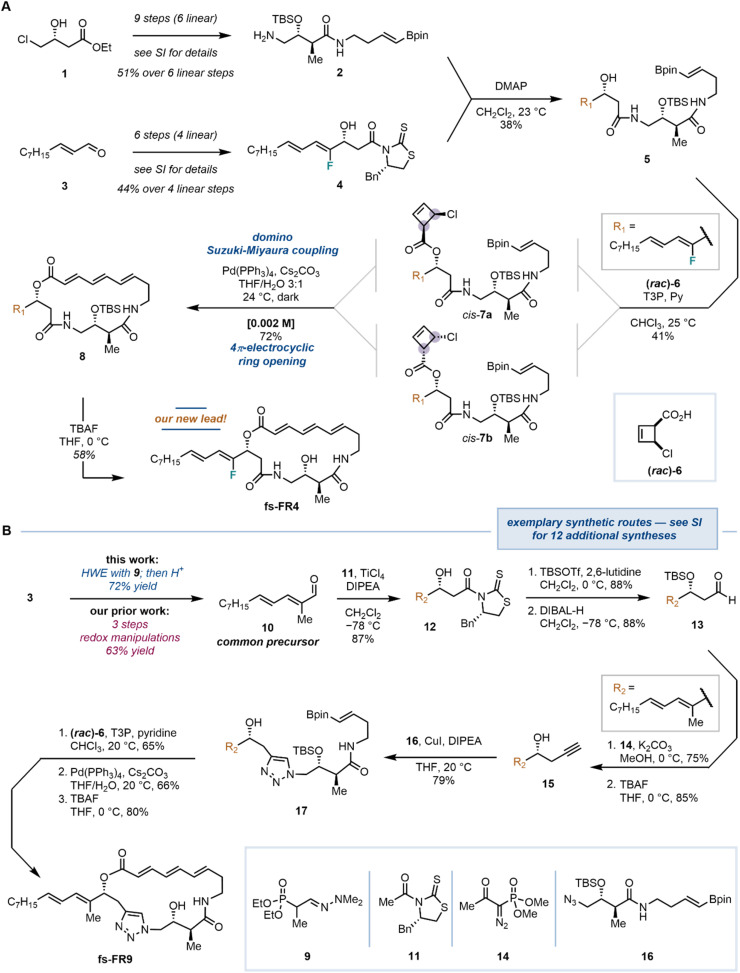
Exemplary syntheses (see SI for 12 additional syntheses) of our novel lead compound fs-FR4 (A) and triazole-containing analog fs-FR9 (B), featuring a key domino Suzuki–Miyaura/4π-electrocyclic ring opening macrocyclization. See SI for details. Bpin: boron pinacolato; Me: methyl; THF: tetrahydrofuran; TBSOTf: *tert*-butyldimethylsilyl triflate; Et: ethyl; DMAP: 4-dimethylaminopyridine; T3P: propanephosphonic acid anhydride; Py: pyridine; Ph: phenyl; TBAF: tetrabutylammonium fluoride; HWE: Horner–Wadsworth–Emmons reaction; DIBAL-H: diisobutylaluminium hydride; DIPEA: diisopropylethylamine.

The synthesis of the fully synthetic analogs, along with the natural product, was based on our established protocol, but incorporated newly optimized conditions and readapted routes that improved the overall synthetic efficiency.^19^ The variability in the synthetic route, being applicable to a range of diverse analogs, is exemplified by the two syntheses (novel lead compound fs-FR4, and fs-FR9) shown in Fig. 4 (see SI for synthetic details of all analogs). Commercially available, enantiopure alcohol 1 was initially transformed into advanced intermediate 2, which was isolated in 51% overall yield, over nine steps (six linear). In parallel, aldehyde 3 was converted to Crimmins–Evans aldol product 4 in 44% yield over a sequence of six steps (four linear). The two fragments were combined by means of acyl transfer in the presence of a catalytic amount of DMAP, producing the linear alcohol 5, before undergoing a key esterification with cyclobutene carboxylic acid (±)-6. Notably, we recognized during our previous work that the esterification reactions between the alcohol intermediates and the cyclobutene carboxylic acid (±)-6 in the presence of Ghosez's reagent (1-chloro-*N*,*N*,2-trimethylprop-1-en-1-amine), despite it being the coupling agent of choice, constituted a bottleneck for the syntheses of most analogs (see SI for details): despite meticulous efforts, undesired epimerization α- to the activated carboxylates, induced by the inherently α-acidic acyl chloride formed *in situ* (not shown herein, see SI for details), afforded undesired mixtures of diastereoisomers on the cyclobutene (*e.g.*, *trans*-configured cyclobutene 7). Moreover, the coeluting Ghosez's reagent-derived *N*,*N*-dimethylisobutyramide by-product rendered isolations cumbersome and impractical. To tackle this obstacle, we considered that propanephosphonic acid anhydride (T3P)-activated carboxylates could provide intermediates less prone to epimerization, while also eliminating coelution issues with water-soluble phosphonate by-products.^30^ To this end, alcohol 5 was treated with a racemic mixture of cyclobutene carboxylate 6, T3P and pyridine, leading to the clean and selective formation of the two desired *cis*-1,2-disubstituted cyclobutenes *cis*-7a/b, which were isolated in 41% yield. The highlight of our synthetic strategy involved a domino Suzuki–Miyaura cross-coupling/4π-electrocyclic ring opening with both diastereomeric cyclobutenes *cis*-7a and *cis*-7b converging to the 19-membered macrocycle 8 with the desired (*E*,*E*,*E*)-configuration on the triene system. Lastly, a facile TBAF-mediated removal of the TBS group unveiled the novel lead compound fs-FR4.

The synthesis of fs-FR9 constitutes another showcase of the variability of the developed synthetic route and allows demonstration of a second significant improvement in the synthetic efficacy of the FR252921 family (Fig. 4B): a common precursor for the synthesis of the natural product and several of our synthetic analogs, aldehyde 10, was previously obtained in 63% yield over three steps including a costly and efficiency-reducing reduction/oxidation sequence.^19^ In our improved synthesis, the efficiency is rescued by virtue of deploying hydrazone 9 as a Horner–Wadsworth–Emmons reagent, delivering aldehyde 10 (72% yield) in one facile operation followed by treatment with HCl. The synthesis of fs-FR9 was realized by introducing a small extension of the sidechain preparation: protection of the hydroxyl group of 12 with TBSOTf/lutidine, followed by careful reduction of the thiazolidine-2-thione-bound carbonyl yielded 13. Aldehyde 13 was subsequently transformed using the Bestmann–Ohira modification of the Seyferth–Gilbert homologation to yield a terminal alkyne,^31,32^ and the resulting compound was treated with TBAF to cleave the silyl protecting group and yield propargyl alcohol 15, amenable to Cu-mediated azide–alkyne cycloaddition.^33^ Coupling of azide 16 (a common intermediate in the syntheses of most of our analogs) with alkyne 15 was conducted in the presence of stoichiometric amounts of CuI and DIPEA, delivering the linear precursor 17. This compound was then taken through the three remaining steps of the standard synthetic sequence (esterification, domino macrocyclization, TBS removal) to furnish fs-FR9. The synthetic routes for all other new analogs can be found in the Supplementary Information document of this report.

## Conclusions

In summary, a library of fully synthetic analogs of (−)-FR252921 was designed and synthesized to address crucial questions that decipher the SAR of the macrocyclic natural product. Lacking any knowledge on the structural or functional properties of the molecular target of (−)-FR252921, the designed analogs answered questions with regard to plausible roles of explicit molecular architectures of the macrocycles on potency, and allowed us to identify moieties rendered tunable, essential, or of low significance. This level of SAR depth offers a strategic blueprint for future analog development and highlights key pharmacophores essential for immunosuppressive activity. The 15 diverse probes were immunologically evaluated against primary human immune cells (PBMC) for their ability to suppress the production of cytokines upon cell stimulation, in contrast to prior preliminary investigations which focused solely on murine T cells. Notably, our endeavors led to the discovery of a new lead compound, fs-FR4, showing an up to 21-fold higher potency compared to the parent compound (−)-FR252921 in the assays applied. Lastly, from a synthetic perspective, we note that we were also able to significantly improve the efficacy of certain operations and common building block syntheses. Overall, this work demonstrates the power of synthesis in drug discovery and its ability to drive SAR studies by virtue of strategic analog design without knowledge of the target and sets the stage for development of even more potent immunosuppressive analogs of (−)-FR252921.

## Ethical statement

Blood leukocytes were obtained as by-products of apheresis from anonymous healthy volunteer donors at the Department of Transfusion Medicine, Medical University of Vienna, Austria. The use of these residual blood products for research purposes was approved by the donors through written informed consent, which was reviewed and approved by the Ethics Committee and the Legal Department of the Medical University of Vienna. All experiments were performed in accordance with the guidelines of the Medical University of Vienna and applicable Austrian national regulations.

## Author contributions

I. S., M. S. and N. M. designed and managed the project. I. S. and M. S. designed analogs and provided major synthetic contributions. H. Z. and T. L. designed analogs and contributed to their synthesis. K. G., M. D. and F. D. contributed to the synthetic work. L. M. G. and G. S. conceptualized the biological evaluation. L. M. G. performed and analyzed all biological experiments. M.S. contributed to biological experiments. I. S., M. S., H. Z. and L. M. G. contributed to the first draft of the manuscript and all figures. All authors contributed to reviewing and editing the manuscript and figures. D. K. supported project supervision. N. M. supervised the project and acquired funding. G. S. supervised the biological evaluation and acquired funding.

## Conflicts of interest

There are no conflicts to declare.

## Supplementary Material

SC-OLF-D5SC09554A-s001

## Data Availability

The data supporting this article have been included in the main text and as part of the supplementary information (SI). Supplementary information: additional synthetic routes to all analogs. See DOI: https://doi.org/10.1039/d5sc09554a.
