# Supplementary material for: Target-agnostic SAR mapping and immunological evaluation of (−)-FR252921 and analogs against primary human immune cells
Source: Chem Sci. 2026 Apr 27;17(20):9912–20. doi: 10.1039/d5sc09554a (PMC13120711; doi:10.1039/d5sc09554a)
Supplement: SC-017-D5SC09554A-s001 [file SC-017-D5SC09554A-s001.pdf]

Supplementary Information (SI) for Chemical Science.
This journal is © The Royal Society of Chemistry 2026

# Target-agnostic SAR Mapping and Immunological Evaluation of (–)-FR252921 and Analogs against Primary Human Immune Cells

Iakovos Saridakis,[1,3,‡] Manuel Schupp,[1,2,3,‡] Haoqi Zhang,[1,3,§] Thomas Leischner,[1,§] Laura Marie Gail,[2,4,§] Konstantin Günther,[1,2,3] Martina Drescher,[1] Florian Doubek,[1] Daniel Kaiser,[1] Georg Stary[2,4] and Nuno Maulide[1,2,*]

[1]Institute of Organic Chemistry, University of Vienna, Währinger Straße 38, 1090 Vienna (Austria), [2]CeMM Research Center for Molecular Medicine of the Austrian Academy of Sciences, Lazarettgasse 14, AKH BT 25.3, 1090 Vienna (Austria), [3]Vienna Doctoral School in Chemistry (DoSChem), University of Vienna, Währinger Straße 42, 1090 Vienna, Austria, [4]Department of Dermatology, Medical University of Vienna, Währinger Gürtel 18-20, 190 Vienna, Austria

[‡,§]These authors contributed equally

E-Mail: nuno.maulide@univie.ac.at, Homepage: http://maulide.univie.ac.at

# Table of Contents

1. General Information ........ 2
2. General Procedures ........ 3
3. General Building Blocks & FR252921 ........ 13
4. Fully Synthetic FR252921 Analogs ........ 22
5. Role of T3P in esterification with cyclobutene (*rac*)-6. ........ 92
6. NMR Spectra ........ 94
7. Immunological Evaluation ........ 166
8. References ........ 176

# 1. General Information

Unless otherwise stated, all solvents were distilled from appropriate drying agents prior to use or used as received, if anhydrous. All reactions were carried out under an atmosphere of argon in flame-dried glass vessels, unless otherwise stated. All reagents were used as received from commercial suppliers, unless otherwise stated. Pyridine was distilled prior to use from $CaH_2$ under an atmosphere of argon and was subsequently stored in the dark under an atmosphere of argon. DIPEA was distilled twice prior to use (distillation from ninhydrin, followed by distillation from KOH) under an atmosphere of argon and was subsequently stored in the dark under an atmosphere of argon. $TiCl_4$ (1 M in $CH_2Cl_2$) was used as received from the supplier after being transferred to a Schlenk flask for storage. Ghosez's reagent (1-chloro-*N*,*N*,2-trimethyl-1-propenylamine) was distilled prior to use under an atmosphere of argon and used without intermediate storage.

Reaction progress was monitored by thin layer chromatography (TLC) performed on aluminium plates coated with silica gel F254 with 0.2 mm thickness. Chromatograms were visualised by fluorescence quenching with UV light at 254 nm or by staining using potassium permanganate, phosphomolybdic acid or ninhydrin. Flash column chromatography was performed using silica gel 60 (230-400 mesh, Merck and co.) or prepacked columns (Chromabond silica) using a Biotage Selekt Flash Purification System.

Neat infrared spectra were recorded using a Perkin-Elmer Spectrum 100 FT-IR spectrometer. Wavenumbers ($\nu_{max}$) are reported in $cm^{-1}$. Mass spectra were obtained using a Finnigan MAT 8200 or (70 eV) or an Agilent 5973 (70 eV) spectrometer, using electrospray ionisation (ESI). Optical rotation measurements were performed on a Perkin Elmer 341 polarimeter using a 100 mm path-length cell at l = 589 nm. All $^{1}H$-NMR, $^{13}C$-NMR and $^{19}F$-NMR spectra were recorded using Bruker AV-400, AV-500, AV-600 or AV-700 spectrometers at 300K. Chemical shifts are given in parts per million (ppm, δ), referenced to the solvent peak of $CDCl_3$, defined at δ = 7.26 ppm ($^{1}H$-NMR) and δ = 77.16 ppm ($^{13}C$-NMR), MeOH-$d_4$, defined at δ = 3.31 ppm ($^{1}H$-NMR) and δ = 49.00 ppm ($^{13}C$-NMR), and DMSO-$d_6$, defined at δ = 2.50 ppm ($^{1}H$-NMR) and δ = 39.52 ppm ($^{13}C$-NMR). Coupling constants are quoted in Hz (*J*). $^{1}H$-, $^{13}C$- and $^{19}F$-NMR splitting patterns are designated as singlet (s), doublet (d), triplet (t), quartet (q) as they appeared in the spectrum. If the appearance of a signal differs from the expected splitting pattern, the observed pattern is designated as apparent (app). Splitting patterns that could not be interpreted or easily visualised are designated as multiplet (m) or broad (br). Room temperature refers to 20 °C.

# 2. General Procedures

## 2.1. General Procedure A – Synthesis of chiral auxiliary 1-(4-benzyl-2-thioxothiazolidin-3-yl)ethan-1-one (S-3)

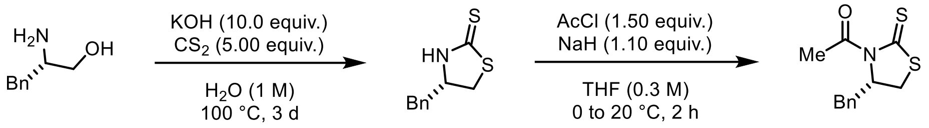


To an aqueous solution of KOH (10.0 equiv.) in water (5 M) at 20 °C was added enantiopure alcohol **S-1** (1.00 equiv.) followed by $CS_2$ (5.00 equiv.). The resulting reaction mixture was heated to 100 °C and stirring at the indicated temperature was continued for 72 h. Afterwards, the reaction mixture was allowed to cool to room temperature and was extracted with $CH_2Cl_2$ (4 x equal volume to $H_2O$). The combined organic layers were dried over $Na_2SO_4$, the solids were filtered off and the solvents were removed *in vacuo* to yield the crude material. The crude product was purified by recrystallization from $Et_2O$ to yield 4-benylthiazolidine-4-thione (**S-2**; average yield 80%).

4-Benylthiazolidine-4-thione **S-2** (1.00 equiv.) was dissolved in THF (0.3 M) and the resulting yellow solution was cooled to 0 °C. NaH (60% w/w in paraffin) (1.10 equiv.) was carefully added to the cold solution and the resulting mixture was stirred at 0 °C for one hour or until $H_2$ evolution ceased. Subsequently, AcCl (1.50 equiv.) was added to the cold reaction mixture and the resulting solution was allowed to warm to room temperature. Stirring was continued for 2 h or until the starting material had been consumed (as indicated by TLC analysis) and the reaction mixture was subsequently quenched by careful addition of a saturated aqueous solution of $NH_4Cl$. The quenched reaction mixture was diluted with EtOAc and the layers were separated. The aqueous layer was extracted with EtOAc (3 x equal volume to THF), and the combined organic layers were washed with brine (1 x equal volume to THF). The organic layers were dried over $Na_2SO_4$, the solids were filtered off and the solvents were removed *in vacuo* to yield the crude auxiliary **S-3**. Purification was achieved by recrystallization from $Et_2O$ to yield **S-3** as yellow crystals (average yield 85%).

## 2.2. General Procedure B – Curtius Rearrangement

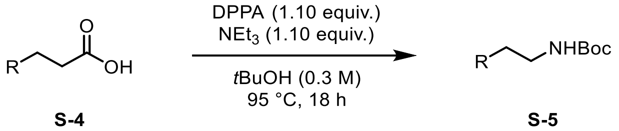


To a two-necked round bottom flask were added 3 Å molecular sieves, the carboxylic acid **S-4** (1.00 equiv.) and *t*BuOH (0.3 M) as a solvent, and the solution was stirred at 30 °C for 30 min. Then, diphenyl phosphoryl azide (DPPA) (1.10 equiv.) and triethylamine (1.10 equiv.) were added, and the resulting mixture was heated at reflux for 18 h. After that period, the mixture was concentrated under reduced pressure, and the crude mixture was purified by column chromatography (silica gel, 25% EtOAc in heptanes) to afford the desired Boc-protected amine **S-5**.

## 2.3. General Procedure C – Hydroboration

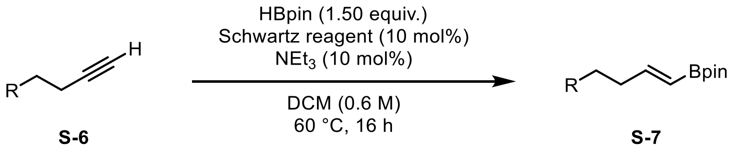


To a flame-dried three-necked round bottom flask under argon fitted with a reflux condenser and two septa was added alkyne **S-6** (1.00 equiv.) and $CH_2Cl_2$ (0.6 M). To the resulting mixture was added HBpin (1.50 equiv.) followed by $Zr(Cp)_2HCl$ (10 mol%, Schwartz's reagent) and $NEt_3$ (10 mol%). The reaction mixture was heated at reflux (typically 60 °C) and stirring was continued at the same temperature for 16 h or until the starting material had been consumed (as indicated by $^1$H-NMR analysis). Afterwards, the reaction mixture was allowed to cool to room temperature and the solvents were removed *in vacuo*. The crude material was purified by flash column chromatography ($SiO_2$, 30% EtOAc in heptane) to yield the desired alkenyl boronic acid pinacol ester **S-7**.

## 2.4. General Procedure D – Boc Removal

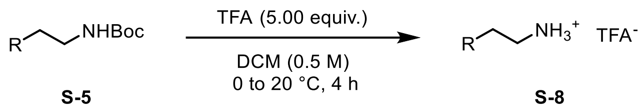

To a solution of *N*-Boc protected amine **S-5** (1.00 equiv.) in $CH_2Cl_2$ (0.5 M) at 0 °C was added TFA (5.00 equiv.) and the reaction mixture was stirred and was allowed to warm to room temperature until the starting material had been consumed (as indicated by TLC analysis). Afterwards, the volatiles were removed *in vacuo* and the resulting ammonium trifluoroacetate **S-8** was used without further purification.

## 2.5. General Procedure E – Synthesis of Azidoalcohols

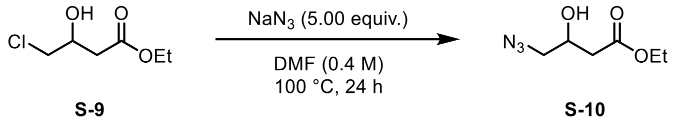

To a solution of chlorohydrine **S-9** (1.00 equiv.) in DMF (0.4 M) was added $NaN_3$ (3.00 equiv.) and the resulting mixture was heated to 100 °C. Stirring was continued at 100 °C until the starting material had been consumed (as indicated by $^1$H-NMR analysis). Afterwards, the reaction mixture was cooled to room temperature and the solids were removed by filtration. The residue was washed with EtOAc and the filtrate was concentrated under reduced pressure. The afforded crude material was purified by flash column chromatography ($SiO_2$, 35% EtOAc in heptane) to yield azidoalcohol **S-10**.

## 2.6. General Procedure F – Fráter-Seebach Stereoselective Alkylation

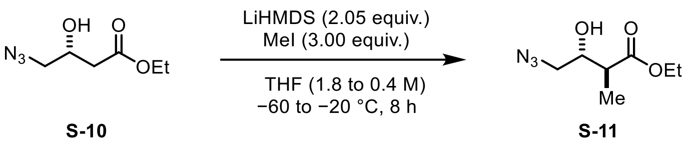

To a solution of azidoalcohol **S-10** (1.00 equiv.) in THF (1.8 M) at −60 °C was slowly added dropwise LiHMDS (1 M in THF) (2.05 equiv.) and the resulting mixture was stirred vigorously at −60 °C for an additional 30 min after complete addition of LiHMDS. Afterwards, the reaction mixture was warmed to −35 °C and stirred at the indicated temperature for 30 min before being cooled to −60 °C. After reaching −60 °C, MeI (2.00 equiv.) was added dropwise and the resulting reaction mixture was stirred at −60 °C for 2 h.

Subsequently, the reaction solution was warmed to −40 °C and stirred for 2 h and then to −20 °C and was stirred at −20 °C for 2 h. After the reaction time had elapsed, the reaction was quenched by addition of an aqueous solution of citric acid (15 wt%) at −20 °C and the pH was adjusted to pH = 3-4. The quenched reaction mixture was warmed to room temperature and the layers were separated. The aqueous layer was extracted with EtOAc (3 x double reaction solvent volume) and the combined organic layers were dried over $Na_2SO_4$. The solids were filtered off, the solvents were removed *in vacuo* and the crude material was purified by flash column chromatography ($SiO_2$, 25% EtOAc in heptane) to yield methylated azidoalcohol **S-11**.

## 2.7. General Procedure G – TBS-protection of Alcohols

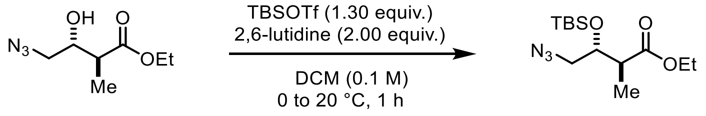


**S-11** **S-12**

To a solution of unprotected alcohol **S-11** in $CH_2Cl_2$ (0.1 M) at 0 °C was added TBSOTf (1.30 equiv.) followed by addition of 2,6-lutidine (2.00 equiv.). The resulting mixture was stirred at 0 °C for 1 to 4 h or until the starting material had been consumed (as indicated by TLC analysis). The reaction was quenched by addition of a sat. aq. solution of $NH_4Cl$ (1 x reaction solvent volume) and the layers were separated. The aqueous layer was extracted with $CH_2Cl_2$ (3 x reaction solvent volume) and the combined organic layers were washed with brine (1 x reaction solvent volume). The combined organic layers were dried over $NaSO_4$, the solids were filtered off and the solvents were removed *in vacuo* to afford the crude material. The product was isolated through flash column chromatography ($SiO_2$, 6% EtOAc in heptane) to yield protected alcohol **S-12**.

## 2.8. General Procedure H – Ester Hydrolysis

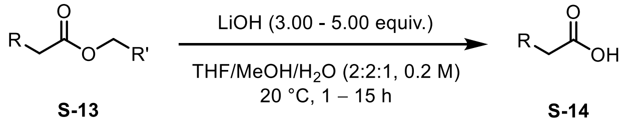


**S-13** **S-14**

To a solution of ester **S-13** (1.00 equiv.) in THF/MeOH/$H_2O$ (2:2:1, 0.2 M) was added LiOH (4.00 equiv., either LiOH or LiOH·$H_2O$) and the resulting reaction mixture was stirred at room temperature until the

starting material had been consumed (typically 1 to 15 h, as indicated by TLC analysis). Afterwards, THF and MeOH were removed under reduced pressure and the remaining slurry was adjusted to pH = 2-3 by addition of 1 M HCl. The slurry was diluted with EtOAc and $H_2O$ and the layers were separated. The aqueous layer (at pH = 2-3) was extracted with EtOAc (5 x reaction solvent volume) and the combined organic layers were washed with brine (1 x reaction solvent volume). The combined organic layers were dried over $Na_2SO_4$, the solids were filtered off and the solvents were removed *in vacuo* to afford the carboxylic acid **S-14** which was used without further purification.

## 2.9. General Procedure I – HATU-mediated Amide Coupling

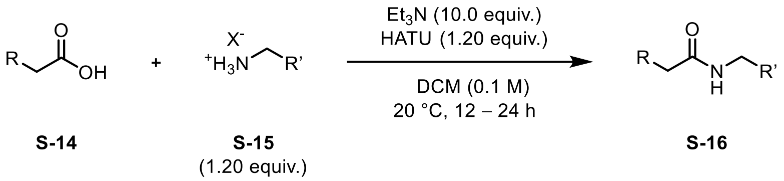


To a solution of carboxylic acid **S-14** (1.00 equiv.) and ammonium salt **S-15** (1.20 equiv.) in $CH_2Cl_2$ (0.1 M) at 0 °C was added $Et_3N$ (10.0 equiv.) followed by addition of HATU (1.20 equiv.). The reaction mixture was allowed to warm to room temperature and was stirred for 12 to 24 h. Afterwards, the reaction was quenched by addition of 1 M HCl (11.0 eq) and the aqueous layer was extracted with $CH_2Cl_2$ (3 x reaction solvent volume). The combined organic layers were washed with brine (1 x reaction solvent volume), dried over $Na_2SO_4$, the solids were filtered off and the solvents were removed *in vacuo* to afford the crude amide. The crude material was purified by flash column chromatography ($SiO_2$, 45% to 80% EtOAc in heptane) to afford amide **S-16**.

## 2.10. General Procedure J – Azide Reduction with Zn dust

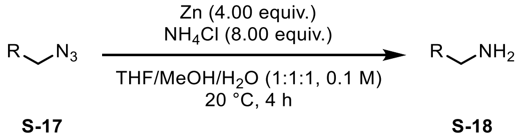


To a solution of azide **S-17** (1.00 equiv.) in THF/MeOH/$H_2O$ (1:1:1, 0.1 M) was added activated Zn dust (4.00 equiv.) followed by addition of $NH_4Cl$ (8.00 equiv.). The resulting slurry was stirred at room temperature for 4 h or until the starting material had been consumed (as indicated by TLC analysis).

Afterwards, the volatiles were removed under reduced pressure and the resulting slurry was basified by addition of a sat. aq. solution of $NaHCO_3$ to pH = 9 and the resulting aqueous phase was extracted with $CH_2Cl_2$ (5 x reaction solvent volume). The combined organic layers were washed with brine, dried over $Na_2SO_4$, the solids were filtered off and the solvents were removed *in vacuo* to yield primary amine **S-18** which was used without further purification.

## 2.11. General Procedure K – Evans–Crimmins Aldol Reaction

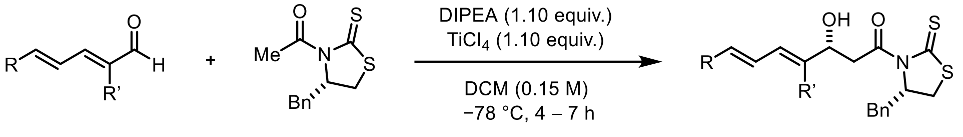


**S-19** (1.03 - 1.10 equiv.) **S-3** (1.00 equiv.) **S-20**

To a flame-dried Schlenk-tube under argon containing activated molecular sieves 3 Å (1 g/mmol) was added auxiliary **S-3** (1.00 equiv.) and $CH_2Cl_2$ (0.3 M). The resulting yellow solution was cooled to −78 °C before $TiCl_4$ (1 M in $CH_2Cl_2$) (1.10 equiv.) was carefully added dropwise to yield a dark orange slurry and stirring was continued for 10 min after complete addition. To the activated reaction mixture DIPEA (1.10 equiv.) was subsequently carefully added dropwise at −78 °C resulting in a change of color to deep red/dark brown of the reaction mixture. The resulting reaction mixture was stirred for 30 min at −78 °C before a solution of aldehyde **S-19** (1.03 equiv.) was carefully added dropwise (*Note: the addition may occur more quickly if the solution of aldehyde is cooled to −78 °C prior to addition and is added via transfer cannula. This is especially recommended on scales exceeding 2.00 mmol*). After the aldehyde had been added, the reaction was stirred at −78 °C for 4 to 7 h or until all starting material had been consumed (as indicated by TLC analysis). The reaction was quenched by careful addition of a sat. aq. solution of $NH_4Cl$ at −78 °C (equal volume to reaction solvent) and stirring was continued for 5 min before the reaction was warmed to room temperature. The reaction mixture was filtered into a separatory funnel to remove the molecular sieves and the layers were separated. The aqueous layer was extracted with $CH_2Cl_2$ (4 x reaction solvent volume) and the combined organic layers were washed with brine (1 x reaction solvent volume). The combined organic layers were dried over $Na_2SO_4$, the solids were filtered off and the solvents were removed *in vacuo*. The crude material was purified by flash column chromatography ($SiO_2$, 10 to 30% EtOAc in heptane) to yield the aldol product **S-20**.

## 2.12. General Procedure L – Acyl Transfer

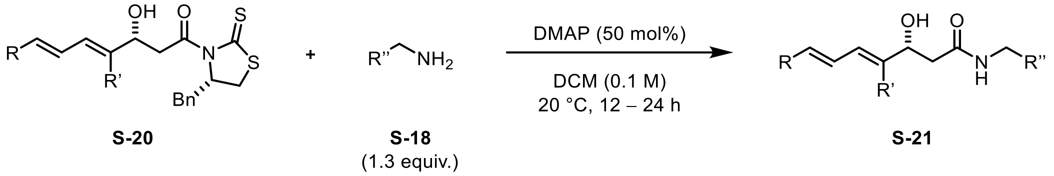

Aldol product **S-20** (1.00 equiv.) and primary amine **S-18** (1.30 equiv.) were dissolved in $CH_2Cl_2$ (0.1 M) and DMAP (50 mol%) was added at room temperature. The resulting mixture was stirred for 12 to 24 h or until the starting material had been consumed (as indicated by TLC analysis). The reaction was quenched by addition of water (1 x reaction solvent volume) and the layers were separated. The aqueous layer was extracted with $CH_2Cl_2$ (5 x reaction solvent volume) and the combined organic layers were washed with brine (1 x reaction solvent volume), dried over $Na_2SO_4$, the solids were filtered off and the solvents were removed under reduced pressure to yield the crude material. The product was isolated by flash column chromatography ($SiO_2$, 33% to 66% EtOAc in heptane followed by 5% to 25% *i*PrOH in heptane) to yield amide **S-21**.

## 2.13. General Procedure M – T3P-mediated Esterification with (±)-*cis*-chlorocyclobutene acid

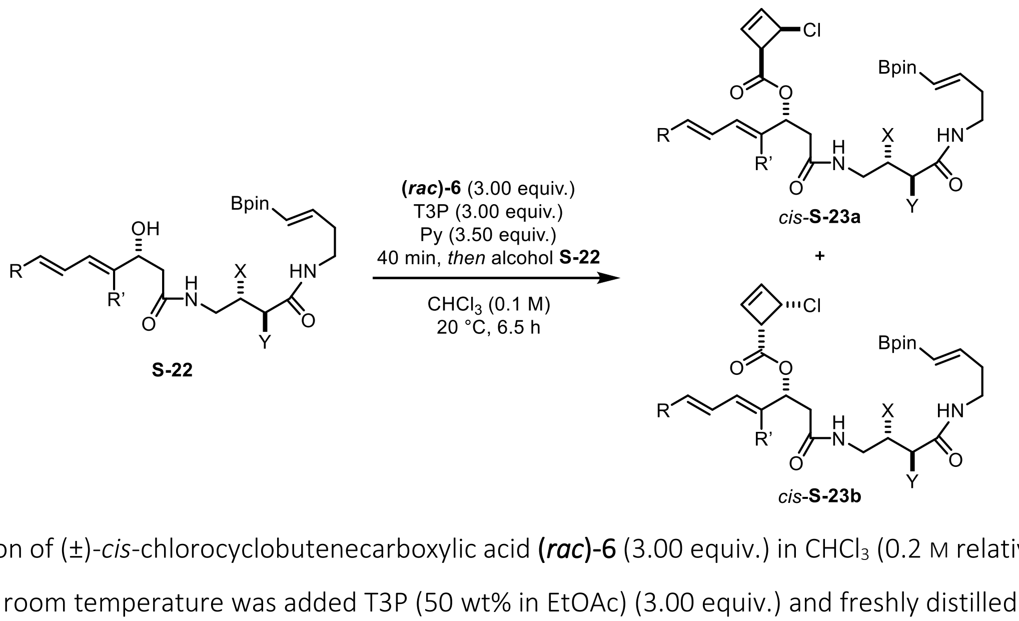

To a solution of (±)-*cis*-chlorocyclobutenecarboxylic acid **(*rac*)-6** (3.00 equiv.) in $CHCl_3$ (0.2 M relative to the alcohol) at room temperature was added T3P (50 wt% in EtOAc) (3.00 equiv.) and freshly distilled pyridine (3.50 equiv.). The resulting mixture was stirred at room temperature for 40 min before a solution of alcohol

**S-22** (1.00 equiv.) in $CHCl_3$ (0.2 M) was. The resulting reaction mixture was stirred for 7 h or until the starting material had been consumed (as indicated by TLC analysis). Afterwards, the reaction was quenched by addition of 1.00 mL of MeOH and stirring was continued for 15 min before water was added. The aqueous layer was extracted with $CH_2Cl_2$ (3 x reaction solvent volume) and the combined organic layers were washed with a sat. aq. solution of $NaHCO_3$ (2 x reaction solvent volume) and brine (2 x reaction solvent volume) before being dried over $Na_2SO_4$. The solids were removed by filtration and the solvents were evaporated under reduced pressure (*Note: the bath temperature must not exceed 25 °C to avoid opening of the cyclobutene*). The crude material was purified by flash column chromatography ($SiO_2$, heptane/EtOAc, 10-15 mL $c_v$ ($c_v$ = column volume) per 0.1 mmol of alcohol, gradient: 30% EtOAc (3 $c_v$) → 35% (2 $c_v$) → 40% (4 $c_v$) → 45% (2 $c_v$) → 50% (4 $c_v$)) to yield pure *cis*-cyclobutene esters **S-23a/b**. (*Note: cis-configured cyclobutene esters are more polar than trans-configured isomers*.)

## 2.14. General Procedure N – Domino Suzuki-Miyaura Macrocyclization

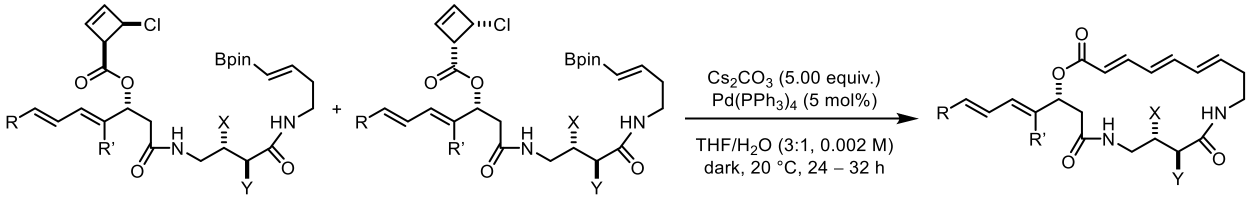


The mixture of *cis*-configured cyclobutene ester *cis*-**S-23a/b** was dissolved in $THF/H_2O$ (3:1 v/v, 0.002 M) and was degassed with argon for 15 minutes before then $Cs_2CO_3$ (5.00 equiv.) was added. The resulting mixture was stirred at room temperature for 10 min before $Pd(PPh_3)_4$ (5 mol%) was added. The reaction flask was then covered with aluminium foil and stirred at room temperature for 24-32 h, after which the reaction was stopped by addition of a saturated aqueous solution of ammonium chloride and the mixture was extracted with $CH_2Cl_2$ (5 x 25% of reaction solvent volume). The resulting organic layers were combined, washed by brine (1 x 25% of reaction solvent volume), dried over anhydrous $MgSO_4$, filtered, and concentrated under reduced pressure. Purification by column chromatography (silica gel, 30% to 50% EtOAc in heptanes) afforded the desired macrocyclic product **S-24** with all-(*E*)-configuration on the triene system.

## 2.15. General Procedure O – TBS Removal

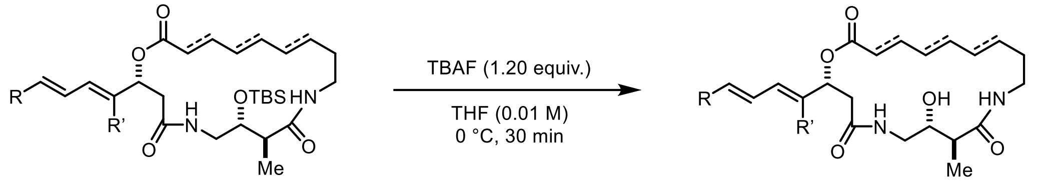


S-25 S-26

To a solution of protected alcohol **S-25** (1.00 equiv., typically 5 – 15 mg) in THF (1.00 mL) at 0 °C was added TBAF (1 M in THF) (1.20 equiv.) and the resulting mixture was stirred at 0 °C for 10 to 30 min or until the starting material had been consumed (as indicated by TLC analysis, 10% MeOH in $CHCl_3$). The reaction was quenched by addition of one drop of a saturated aqueous solution of $NH_4Cl$ and the solvents were removed. The crude material was purified by flash column chromatography ($SiO_2$, 5% MeOH in $CHCl_3$) to yield the desired natural product analogues **S-26**.

## 2.16. General Procedure P – Shiina Macrocyclization

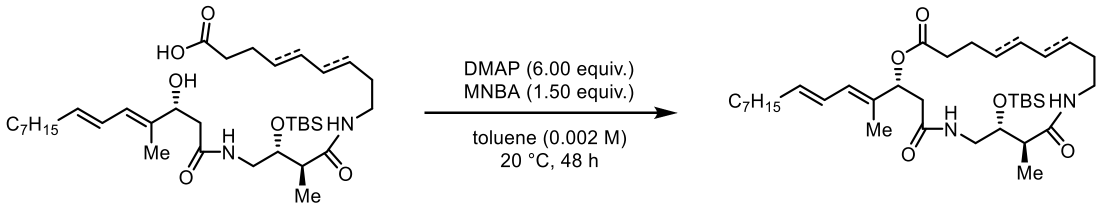


S-27 S-28

To a flame-dried Schlenk tube equipped with molecular sieves 4 Å, DMAP (6.00 equiv.) and MNBA (1.50 equiv.) were added under argon flow. Dry toluene (0.002 M) was then added, followed by macrocyclization precursor **S-27** (1.00 equiv.) dissolved in dry toluene via a syringe, and the mixture was stirred at ambient temperature for 48 h. Then, the reaction mixture was diluted with EtOAc and filtered to remove the molecular sieves. The organic phase was washed twice with a saturated aqueous solution of sodium bicarbonate and brine, dried over anhydrous magnesium sulfate, filtered, and the solvents were evaporated under reduced pressure. The product was purified by column chromatography (silica gel, 10% → 15% → 60% EtOAc in heptanes) delivering the desired macrolactone **S-28**.

## 2.17. General Procedure Q – Horner–Wadsworth–Emmons Olefination

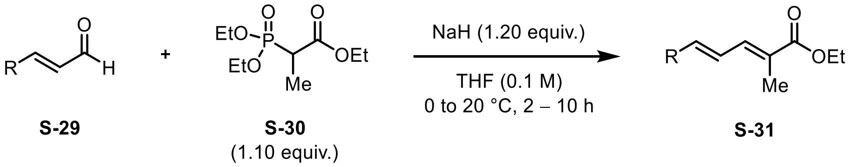


Phosphonate **S-30** (1.10 equiv.) was added to a suspension of NaH (60% w/w in paraffin) (1.20 equiv.) in THF (0.2 M) at 0 °C. The resulting mixture was stirred for one hour or until evolution of $H_2$ ceased before a solution of aldehyde **S-29** (1.00 equiv.) in THF (0.2 M) was added dropwise at 0 °C and the resulting mixture was allowed to warm to room temperature. After 2–10 hours, the starting material had been consumed (as indicated by TLC analysis) and the reaction mixture was carefully quenched by the dropwise addition of water at 0 °C. The quenched reaction mixture was further diluted with water and was subsequently extracted with EtOAc (3 x equal volume to THF). The combined organic layers were dried over $Na_2SO_4$, the solids were filtered off and the solvents were removed *in vacuo* to yield the crude product. The crude product was purified by flash column chromatography ($SiO_2$, 10% EtOAc in heptane) to yield the allylic esters **S-31**.

## 2.18. General Procedure R – Ester Reduction

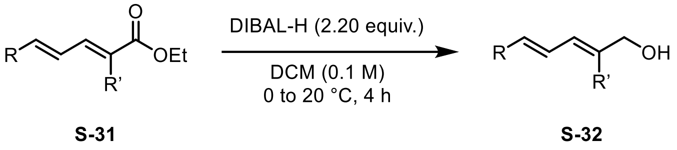


To a solution of allylic ester **S-31** (1.00 equiv.) in $CH_2Cl_2$ (0.1 M) at 0 °C was carefully added DIBAL (1 M in solvent; $CH_2Cl_2$, toluene or $Et_2O$) (2.20 equiv.). Stirring was maintained at 0 °C for 4 h or until the starting material had been consumed (as indicated by TLC analysis) before the reaction was quenched by either of two methods.

<u>Method A</u>: The reaction was quenched by careful addition of 0.2 mL of water and stirring was continued for 30 min. Then, $MgSO_4$ was added to dry the quenched reaction mixture and stirring was continued until an opaque gel formed (typically 15 to 30 min). The gel was filtered over a short pad of Celite® and the filter cake was washed with EtOAc. The filtrate was concentrated and the thusly afforded crude material was purified by flash column chromatography ($SiO_2$, heptane/EtOAc) to afford the allylic alcohol **S-32**.

Method B: The reaction was quenched by careful addition of (2 x solvent volume) mL of a saturated aqueous solution of Rochelle's salt at 0 °C and stirring was continued until two phases formed. The layers were separated and the aqueous layer was extracted with EtOAc (3 x equal volume to $CH_2Cl_2$). The combined organic layers were dried over $Na_2SO_4$, the solids were filtered off and the solvents were removed *in vacuo* to yield the crude product. The crude material was purified by flash column chromatography ($SiO_2$, heptane/EtOAc) to yield the pure allylic alcohols **S-32**.

### 2.19. General Procedure S – Allylic Alcohol Oxidation

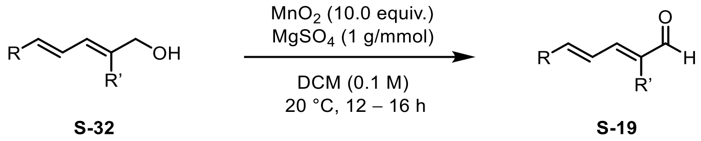


To a solution of allylic alcohol **S-32** (1.00 equiv.) in $CH_2Cl_2$ (0.1 M) at 20 °C was added $MgSO_4$ (1 g/mmol) followed by addition of $MnO_2$ (10.0 equiv.). The reaction mixture was subsequently stirred at 20 °C for 12-16 h or until the starting material had been consumed (as indicated by TLC analysis or $^1$H-NMR). Subsequently, the reaction mixture was filtered through a short pad of Celite® to remove the solids and the filtrate was concentrated to yield the crude product. The crude material was subsequently purified by flash column chromatography ($SiO_2$, 5% EtOAc in heptane) to yield the desired allylic aldehydes **S-19**.

# 3. General Building Blocks & FR252921

### 3.1. (±)-(1*S*,4*S*)-4-Chlorocyclobut-2-ene-1-carboxylic acid (*rac*)-6

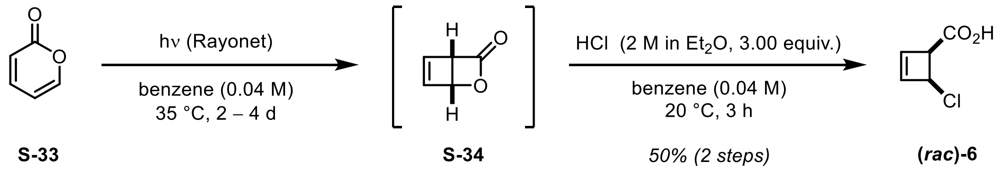


*Representative procedure*: 2-Pyrone **S-33** (650 mg, 6.76 mmol, 1.00 equiv.) was dissolved in benzene (180 mL, 0.04 M, degassed with argon bubbling) and the solution was irradiated with 300 nm in a Rayonet apparatus for 2–4 days until conversion of the 2-pyrone had stopped (followed by $^1$H-NMR). The resulting mixture was quickly filtered, and the filtrate was added to a flame-dried Schlenk tube equipped with 4 Å

molecular sieves under argon flow. To the resulting mixture, containing the desired cyclobutene lactone **S-34**, was added dry HCl (2.0 M in $Et_2O$) (10.1 mL, 20.3 mmol, 3.00 equiv.) and the mixture was vigorously stirred for 3 h at room temperature. Afterwards, the crude mixture was filtered through a paper filter and concentrated under reduced pressure (*Note: The temperature of the water bath of the rotatory evaporator should not exceed 25* °C *to avoid undesired ring opening*). Purification by column chromatography ($SiO_2$, 3% AcOH + 20% EtOAc in heptane) delivered the desired *cis*-configured cyclobutene carboxylic acid **(*rac*)-6** (450 mg, 3.50 mmol, 50%) as a colorless solid.

All spectroscopic data in accordance with the literature.[1]

**$^1$H NMR** (400 MHz, $CDCl_3$)**:** δ 9.70 (br s, 1H), 6.32 – 6.28 (m, 2H), 5.12 (d, *J* = 4.1 Hz, 1H), 4.15 (d, *J* = 4.3 Hz, 1H) ppm.

## 3.2. (*S*)-1-(4-Benzyl-2-thioxothiazolidin-3-yl)ethan-1-one ((*S*)-S-3)

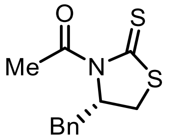


Synthesized according to **General Procedure A** on 54.4 mmol scale to yield (*S*)-**S-3** (12.9 g, 51.3 mmol, 94%) as yellow crystals.

All spectroscopic data in accordance with the literature.[2]

**$^1$H NMR** (400 MHz, $CDCl_3$): δ 7.38 – 7.32 (m, 2H), 7.31 – 7.26 (m, 3H), 5.42 – 5.35 (m, 1H), 3.39 (dd, *J* = 11.6, 7.3 Hz, 1H), 3.22 (dd, *J* = 13.2, 3.7 Hz, 1H), 3.04 (dd, *J* = 13.2, 10.6 Hz, 1H), 2.89 (d, *J* = 11.6 Hz, 1H), 2.80 (s, 3H) ppm.

## 3.3. *tert*-Butyl but-3-yn-1-ylcarbamate (S-35)

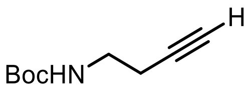


Synthesized according to **General Procedure B** on 90.4 mmol scale to yield **S-35** (13.6 g, 80.5 mmol, 89%) as a colorless, viscous oil.

All spectroscopic data in accordance with the literature.[1]

**$^1$H NMR** (400 MHz, $CDCl_3$): δ 4.83 (s, 1H), 3.28 (dd, *J* = 12.4, 6.1 Hz, 2H), 2.38 (td, *J* = 6.5, 2.6 Hz, 2H), 1.99 (t, *J* = 2.6 Hz, 1H), 1.45 (s, 9H) ppm.

### 3.4. *tert*-Butyl (*E*)-(4-(4,4,5,5-tetramethyl-1,3,2-dioxaborolan-2-yl)but-3-en-1-yl)-carbamate (S-36)

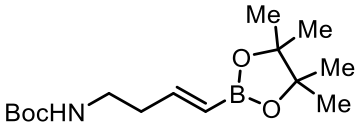


Synthesized according to **General Procedure C** on 37.6 mmol scale to yield **S-36** (10.2 g, 32.0 mmol, 85%) as a colorless, viscous oil.

All spectroscopic data in accordance with the literature.[1]

$^1$**H NMR** (400 MHz, $CDCl_3$): δ 6.55 (dt, *J* = 18.0, 6.6 Hz, 1H), 5.51 (dt, *J* = 18.0, 1.4 Hz, 1H), 4.54 (br. s, 1H), 3.29 – 3.13 (m, 2H), 2.39 – 2.24 (m, 2H), 1.43 (s, 9H), 1.27 (s, 12H) ppm.

### 3.5. (*E*)-4-(4,4,5,5-Tetramethyl-1,3,2-dioxaborolan-2-yl)but-3-en-1-ammonium trifluoroacetate (S-37)

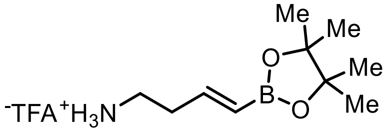


Synthesized according to **General Procedure D** on 29.0 mmol scale to yield **S-37** (9.06 g, 29.0 mmol, quantitative) as a colorless, viscous oil.

All spectroscopic data in accordance with the literature.[1]

$^1$**H NMR** (400 MHz, $CDCl_3$): δ 6.48 (dt, *J* = 18.0, 6.6 Hz, 1H), 5.66 (d, *J* = 18.0 Hz, 1H), 3.23 (dd, *J* = 12.0, 6.0 Hz, 2H), 2.61 – 2.54 (m, 2H), 1.28 (s, 12H) ppm.

### 3.6. Ethyl (*R*)-4-azido-3-hydroxybutanoate (S-38)

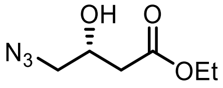


Synthesized according to **General Procedure E** on 180 mmol scale to yield **S-38** (25.3 g, 146 mmol, 81%) as a colorless oil.

All spectroscopic data in accordance with the literature.[1]

$^1$**H NMR** (400 MHz, $CDCl_3$): δ 4.24 – 4.15 (m, 3H), 3.41 – 3.30 (m, 2H), 3.13 (d, *J* = 4.2 Hz, 1H), 2.54 (dd, *J* = 6.2, 2.9 Hz, 2H), 1.29 (t, *J* = 7.1 Hz, 3H) ppm.

### 3.7. Ethyl (2*S*,3*R*)-4-azido-3-hydroxy-2-methylbutanoate (S-39)

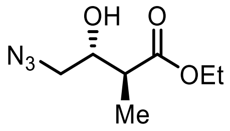

Synthesized according to **General Procedure F** on 53.4 mmol scale to yield **S-39** (7.06 g, 37.9 mmol, 71%) as a colorless oil.

All spectroscopic data in accordance with the literature.[1]

$^{1}$**H NMR** (400 MHz, $CDCl_3$): δ 4.19 (q, *J* = 7.1 Hz, 2H), 3.87 (qd, *J* = 6.3, 3.9 Hz, 1H), 3.42 (dd, *J* = 12.7, 3.8 Hz, 1H), 3.34 (dd, *J* = 12.7, 6.2 Hz, 1H), 3.15 (d, *J* = 6.1 Hz, 1H), 2.66 (p, *J* = 7.2 Hz, 1H), 1.28 (t, *J* = 7.1 Hz, 3H), 1.22 (d, *J* = 7.2 Hz, 3H) ppm.

### 3.8. Ethyl (2*S*,3*R*)-4-azido-3-((*tert*-butyldimethylsilyl)oxy)-2-methylbutanoate (S-40)

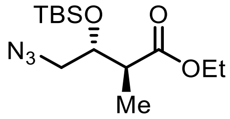

Synthesized according to **General Procedure G** on 47.8 mmol scale to yield **S-40** (14.4 g, 47.8 mmol, quantitative) as a colorless oil.

All spectroscopic data in accordance with the literature.[1]

$^{1}$**H NMR** (400 MHz, $CDCl_3$): δ 4.13 (q, *J* = 7.1 Hz, 2H), 4.03 (td, *J* = 5.8, 3.8 Hz, 1H), 3.40 (dd, *J* = 12.8, 3.8 Hz, 1H), 3.23 (dd, *J* = 12.8, 5.5 Hz, 1H), 2.80 – 2.70 (m, 1H), 1.27 (t, *J* = 7.1 Hz, 3H), 1.12 (d, *J* = 7.2 Hz, 3H), 0.89 (s, 9H), 0.13 (s, 3H), 0.08 (s, 3H) ppm.

### 3.9. (2*S*,3*R*)-4-Azido-3-((*tert*-butyldimethylsilyl)oxy)-2-methylbutanoic acid (S-41)

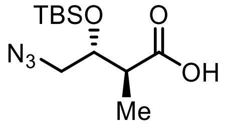

Synthesized according to **General Procedure H** on 26.1 mmol scale to yield **S-41** (6.90 g, 25.1 mmol, 96%) as a colorless oil.

All spectroscopic data in accordance with the literature.[1]

$^{1}$**H NMR** (400 MHz, $CDCl_3$): δ 4.02 (td, *J* = 5.6, 4.1 Hz, 1H), 3.44 (dd, *J* = 12.8, 4.0 Hz, 1H), 3.27 (dd, *J* = 12.8, 5.4 Hz, 1H), 2.85 – 2.76 (m, 1H), 1.18 (d, *J* = 7.2 Hz, 3H), 0.90 (s, 9H), 0.14 (s, 3H), 0.10 (s, 3H) ppm.

### 3.10. (2*S*,3*R*)-4-Azido-3-((*tert*-butyldimethylsilyl)oxy)-2-methyl-*N*-((*E*)-4-(4,4,5,5-tetramethyl-1,3,2-dioxaborolan-2-yl)but-3-en-1-yl)butanamide (16)

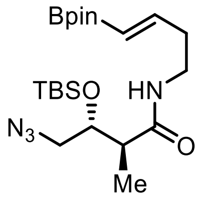

Synthesized according to **General Procedure I** on 16.4 mmol scale to yield **16** (6.31 g, 15.1 mmol, 78%) as a colorless oil.

All spectroscopic data in accordance with the literature.[1]

**$^1$H NMR** (400 MHz, $CDCl_3$): δ 6.55 (dt, *J* = 18.0, 6.5 Hz, 1H), 5.92 (s, 1H), 5.52 (dt, *J* = 18.0, 1.4 Hz, 1H), 3.91 (dt, *J* = 6.2, 4.5 Hz, 1H), 3.47 (ddd, *J* = 13.0, 10.1, 5.3 Hz, 2H), 3.28 – 3.18 (m, 2H), 2.50 – 2.40 (m, 1H), 2.36 (qd, *J* = 6.7, 1.4 Hz, 2H), 1.27 (s, 12H), 1.11 (d, *J* = 7.1 Hz, 3H), 0.90 (s, 9H), 0.13 (s, 3H), 0.08 (s, 3H) ppm.

### 3.11. (2*S*,3*R*)-4-Amino-3-((*tert*-butyldimethylsilyl)oxy)-2-methyl-*N*-((*E*)-4-(4,4,5,5-tetramethyl-1,3,2-dioxaborolan-2-yl)but-3-en-1-yl)butanamide (2)

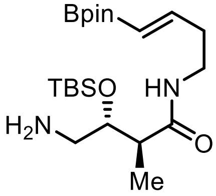

Synthesized according to **General Procedure J** on 9.95 mmol scale to yield **2** (4.25 g, 7.97 mmol, quant.) as a colorless, viscous oil.

All spectroscopic data in accordance with the literature.[1]

**$^1$H NMR** (400 MHz, $CDCl_3$): δ 6.43 (dt, *J* = 17.9, 6.5 Hz, 1H), 6.20 (s, 1H), 5.37 (d, *J* = 18.0 Hz, 1H), 3.64 – 3.57 (m, 1H), 3.36 – 3.23 (m, 1H), 3.11 (dt, *J* = 20.0, 6.7 Hz, 1H), 2.72 – 2.60 (m, 2H), 2.48 – 2.33 (m, 1H), 2.27 – 2.18 (m, 2H), 1.13 (s, 12H), 1.00 (d, *J* = 7.2 Hz, 3H), 0.77 (s, 9H), −0.03 (s, 3H), −0.05 (s, 3H) ppm.

### 3.12. (*E*)-1,1-Dimethyl-2-((2*E*,4*E*)-2-methyldodeca-2,4-dien-1-ylidene)hydrazine (10a)

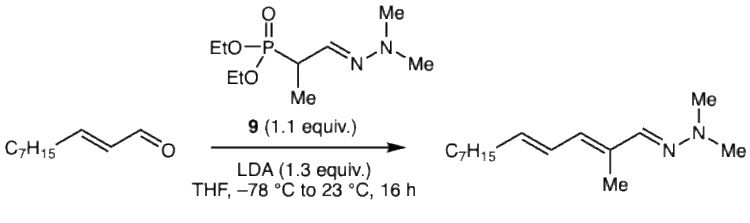


Diethyl (*E*)-(1-(2,2-dimethylhydrazineylidene)propan-2-yl)phosphonate was prepared in accordance with previously reported procedures.[3]

To a solution of freshly distilled diisopropylamine (0.73 mL, 5.20 mmol, 1.30 equiv.) in THF (0.2 M) *n*-butyllithium (2.08 mL, 5.20 mmol, 1.30 equiv., 2.5 M in hexanes) was added dropwise at 0 °C and the reaction mixture stirred for 5 min. A solution of diethyl (*E*)-(1-(2,2-dimethylhydrazineylidene)propan-2-yl)phosphonate **9** (1.04 g, 4.40 mmol, 1.10 equiv.) in THF (0.2 M) was added dropwise and the reaction mixture stirred for 2 hours at 0 °C. *Trans*-2-decenal (0.77 mL, 4.00 mmol, 1.00 equiv.) was added and the resulting mixture stirred at room temperature for 16 h or until full conversion of the starting material was observed (as indicated by $^1$H-NMR or TLC analysis). After the starting material had been consumed, the reaction was quenched by addition of 30 mL of water. The resulting mixture was extracted with EtOAc (3 x 50 mL), and the combined organic layers were washed with brine (1 x 75 mL). The combined organic layers were dried over $NaSO_4$, the solids were filtered off and the solvents were removed in vacuo to afford the crude material. The product was isolated through flash column chromatography ($SiO_2$, 5% EtOAc in heptane) to yield **10a** (821 mg, 3.47 mmol, 87%) as a yellow oil.

**$^1$H NMR** (700 MHz, $CDCl_3$): δ 7.01 (s, 1H), 6.42 (ddt, *J* = 15.1, 11.1, 1.5 Hz, 1H), 6.09 (d, *J* = 10.0 Hz, 1H), 5.74 (dt, *J* = 14.6, 7.2 Hz, 1H), 2.84 (s, 6H), 2.14 (q, *J* = 8.1 Hz, 2H), 1.94 (s, 3H), 1.44 – 1.37 (m, 2H), 1.34 – 1.24 (m, 8H), 0.88 (t, *J* = 7.2 Hz, 3H) ppm.

**$^{13}$C-NMR** (176 MHz, $CDCl_3$): δ 139.5, 135.7, 133.5, 131.0, 126.9, 43.2, 33.4, 32.0, 29.6, 29.3, 29.3, 22.8, 14.2, 12.0 ppm.

**HRMS (ESI$^+$):** calculated for $[M+H]^+$ ($C_{15}H_{29}N_2^+$) requires *m/z* 237.2325, found *m/z* 237.2319.

**FT-IR (neat) $ν_{max}$:** 2954, 2922, 2852, 2783, 1556, 1466, 1442, 1362, 1263, 1133, 1032, 961, 903, 867, 834 cm$^{-1}$.

### 3.13. (2*E*,4*E*)-2-Methyldodeca-2,4-dienal (10)

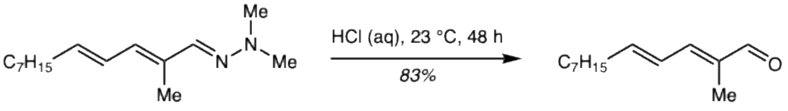


**10a** **10**

To a solution of hydrazone **10a** (591 mg, 2.50 mmol, 1.00 equiv.) in aq. HCl (1.0 M) pentane (0.1 M) was added and the reaction mixture stirred for 48 h at room temperature. The layers were separated and the aqueous layer returned to the reaction vessel. Pentane (0.1 M) was added and the reaction mixture stirred for 2 h at room temperature. The layers were separated, and the aqueous layer was extracted with pentane (3 x 25 mL). The combined organic layers were dried over $NaSO_4$, the solids were filtered off and the solvents were removed in vacuo to afford the crude material. The product was isolated through flash column chromatography ($SiO_2$, 5% EtOAc in heptane) to yield **10** (402 mg, 2.07 mmol, 83%) as a yellowish oil. All spectroscopic data in accordance with the literature.[1]

### 3.14. (*R*,4*E*,6*E*)-1-((*S*)-4-Benzyl-2-thioxothiazolidin-3-yl)-3-hydroxy-4-methyltetradeca-4,6-dien-1-one (12)

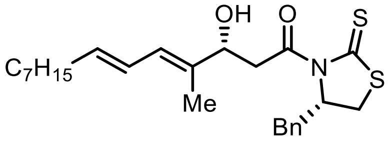


Synthesized according to **General Procedure K** on 20.2 mmol scale to yield **12** (7.29 g, 16.4 mmol, 81%, single diasteromer isolated) as a yellow oil (overll: 13:1 d.r., 7.83 g, 17.6 mmol, 87%).

All spectroscopic data in accordance with the literature.[1]

**$^1$H NMR** (400 MHz, $CDCl_3$): δ 7.38 – 7.32 (m, 2H), 7.29 (d, *J* = 7.1 Hz, 3H), 6.25 (dd, *J* = 14.9, 10.8 Hz, 1H), 6.11 (d, *J* = 10.8 Hz, 1H), 5.78 – 5.67 (m, 1H), 5.38 (ddd, *J* = 10.6, 7.1, 3.9 Hz, 1H), 4.64 (d, *J* = 8.2 Hz, 1H), 3.56 (dd, *J* = 17.4, 2.7 Hz, 1H), 3.48 – 3.37 (m, 2H), 3.25 (dd, *J* = 13.1, 3.8 Hz, 1H), 3.09 – 3.01 (m, 1H), 2.90 (d, *J* = 11.5 Hz, 1H), 2.11 (app. dd, *J* = 14.2, 7.1 Hz, 2H), 1.78 (s, 3H), 1.42 – 1.34 (m, 2H), 1.28 (br. s, 8H), 0.88 (t, *J* = 6.8 Hz, 3H) ppm.

### 3.15. (*R*,4*E*,6*E*)-*N*-((2*R*,3*S*)-2-((*tert*-Butyldimethylsilyl)oxy)-3-methyl-4-oxo-4-(((*E*)-4-(4,4,5,5-tetramethyl-1,3,2-dioxaborolan-2-yl)but-3-en-1-yl)amino)butyl)-3-hydroxy-4-methyltetradeca-4,6-dienamide (S-42)

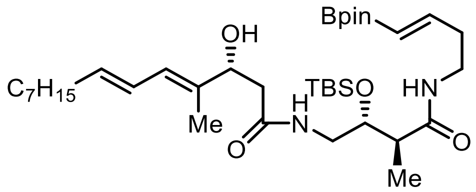


Synthesized according to **General Procedure L** on 1.25 mmol scale to yield **S-42** (479 mg, 0.722 mmol, 57%) as a colorless, viscous oil.

All spectroscopic data in accordance with the literature.[1]

**$^1$H NMR** (400 MHz, $CDCl_3$): δ 6.69 – 6.63 (m, 1H), 6.52 (dt, *J* = 18.0, 6.5 Hz, 1H), 6.25 (t, *J* = 5.6 Hz, 1H), 6.23 – 6.14 (m, 1H), 6.05 (d, *J* = 10.8 Hz, 1H), 5.72 – 5.61 (m, 1H), 5.49 (d, *J* = 18.0 Hz, 1H), 4.40 (dt, *J* = 12.3, 6.3 Hz, 1H), 3.84 (dt, *J* = 8.1, 4.2 Hz, 1H), 3.73 – 3.63 (m, 1H), 3.45 – 3.32 (m, 1H), 3.30 – 3.19 (m, 1H), 2.93 – 2.81 (m, 1H), 2.44 – 2.30 (m, 5H), 2.07 (app. dd, *J* = 14.0, 6.9 Hz, 2H), 1.72 (s, 3H), 1.35 (app. dd, *J* = 17.4, 11.5 Hz, 2H), 1.28 – 1.22 (m, 20H), 1.11 (d, *J* = 7.2 Hz, 3H), 0.89 – 0.82 (m, 12H), 0.14 (s, 3H), 0.07 (s, 3H) ppm.

### 3.16. (*R*,4*E*,6*E*)-1-(((2*R*,3*S*)-2-((*tert*-Butyldimethylsilyl)oxy)-3-methyl-4-oxo-4-(((*E*)-4-(4,4,5,5-tetramethyl-1,3,2-dioxaborolan-2-yl)but-3-en-1-yl)amino)butyl)amino)-4-methyl-1-oxotetradeca-4,6-dien-3-yl (1*S*,4*R*)-4-chlorocyclobut-2-ene-1-carboxylate (*cis*-S-43a/b)

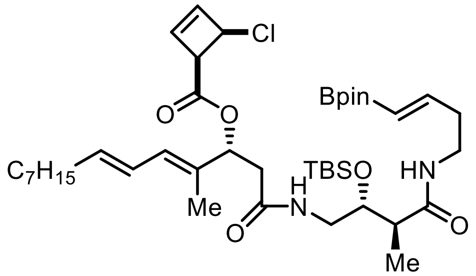


Synthesized according to **General Procedure M** on 0.280 mmol scale to yield *cis*-**S-43a/b** (146 mg, 0.224 mmol, 79%) as a colorless foam.

All spectroscopic data in accordance with the literature.[1]

**$^1$H NMR** (400 MHz, $CDCl_3$): δ 6.55 (dt, *J* = 18.0, 6.4 Hz, 1H), 6.29 – 6.10 (m, 5H), 5.72 (dt, *J* = 13.8, 6.8 Hz, 1H), 5.63 (dd, *J* = 7.6, 5.6 Hz, 1H), 5.51 (d, *J* = 18.0 Hz, 1H), 5.08 (d, *J* = 4.2 Hz, 1H), 4.07 (d, *J* = 4.3 Hz, 1H), 3.83 – 3.77 (m, 1H), 3.77 – 3.70 (m, 1H), 3.43 (td, *J* = 13.3, 6.7 Hz, 1H), 3.26 (td, *J* = 12.8, 6.6 Hz, 1H),

2.83 – 2.73 (m, 1H), 2.66 (dd, $J$ = 14.4, 7.9 Hz, 1H), 2.50 (dd, $J$ = 14.4, 5.5 Hz, 1H), 2.41 – 2.31 (m, 3H), 2.07 (dd, $J$ = 14.5, 7.4 Hz, 2H), 1.77 (s, 3H), 1.37 (t, $J$ = 12.5 Hz, 2H), 1.25 (s, 20H), 1.10 (d, $J$ = 7.1 Hz, 3H), 0.91 – 0.84 (m, 12H), 0.16 – 0.11 (m, 3H), 0.08 (s, 3H) ppm.

### 3.17. (2*R*,7*R*,8*S*,13*E*,15*E*,17*E*)-7-((*tert*-Butyldimethylsilyl)oxy)-2-((2*E*,4*E*)-dodeca-2,4-dien-2-yl)-8-methyl-1-oxa-5,10-diazacyclononadeca-13,15,17-triene-4,9,19-trione (fs-FR11)

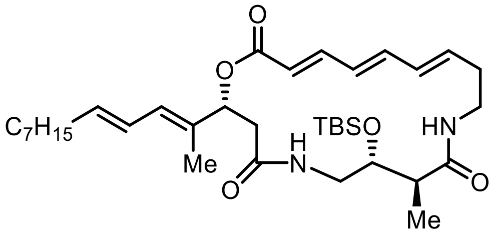


Synthesized according to **General Procedure N** on 24.8 µmol scale to yield **fs-FR11** (13.7 mg, 22.3 µmol, 90%) as a colorless foam.

All spectroscopic data in accordance with the literature.[1]

**$^1$H NMR** (400 MHz, $CDCl_3$): δ 7.29 – 7.20 (m, 1H), 6.45 (dd, $J$ = 14.9, 10.9 Hz, 1H), 6.16 (qd, $J$ = 24.4, 11.4 Hz, 4H), 5.99 (d, $J$ = 8.1 Hz, 1H), 5.91 (dd, $J$ = 18.4, 7.1 Hz, 1H), 5.83 – 5.77 (m, 1H), 5.72 (dd, $J$ = 15.2, 9.2 Hz, 2H), 5.63 (dd, $J$ = 11.0, 3.0 Hz, 1H), 4.14 – 3.99 (m, 2H), 3.47 (dt, $J$ = 15.9, 8.1 Hz, 1H), 2.99 – 2.97 (m, 1H), 2.59 – 2.35 (m, 5H), 2.24 – 2.14 (m, 1H), 2.10 (dd, $J$ = 14.3, 7.1 Hz, 2H), 1.79 (s, 3H), 1.42 – 1.33 (m, 2H), 1.33 – 1.23 (m, 8H), 1.12 (d, $J$ = 6.7 Hz, 3H), 0.90 – 0.85 (m, 12H), 0.08 (s, 3H), 0.05 (s, 3H) ppm.

### 3.18. (–)-FR252921

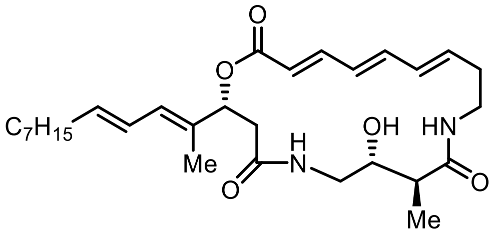


Synthesized according to **General Procedure O** on 12.7 µmol scale to yield **(–)-FR252921** (5.73 mg, 11.4 µmol, 90%) as a white powder.

All spectroscopic data in accordance with the literature.[1]

**$^1$H NMR** (400 MHz, $CDCl_3$): δ 7.32 – 7.22 (m, 1H), 6.51 (dd, *J* = 14.9, 10.8 Hz, 1H), 6.28 – 6.02 (m, 5H), 5.95 (s, 1H), 5.83 – 5.63 (m, 5H), 4.13 (ddd, *J* = 17.2, 13.8, 5.1 Hz, 1H), 3.85 – 3.76 (m, 1H), 3.33 (td, *J* = 10.7, 2.7 Hz, 1H), 3.06 – 2.97 (m, 1H), 2.67 – 2.46 (m, 4H), 2.31 – 2.15 (m, 2H), 2.10 (app. dd, *J* = 14.0, 7.0 Hz, 2H), 1.79 (s, 3H), 1.38 (br. s, 2H), 1.27 (s, 8H), 0.98 (d, *J* = 7.0 Hz, 3H), 0.88 (t, *J* = 6.7 Hz, 3H) ppm.

# 4. Fully Synthetic FR252921 Analogs

## 4.1. Fs-FR1 Synthesis

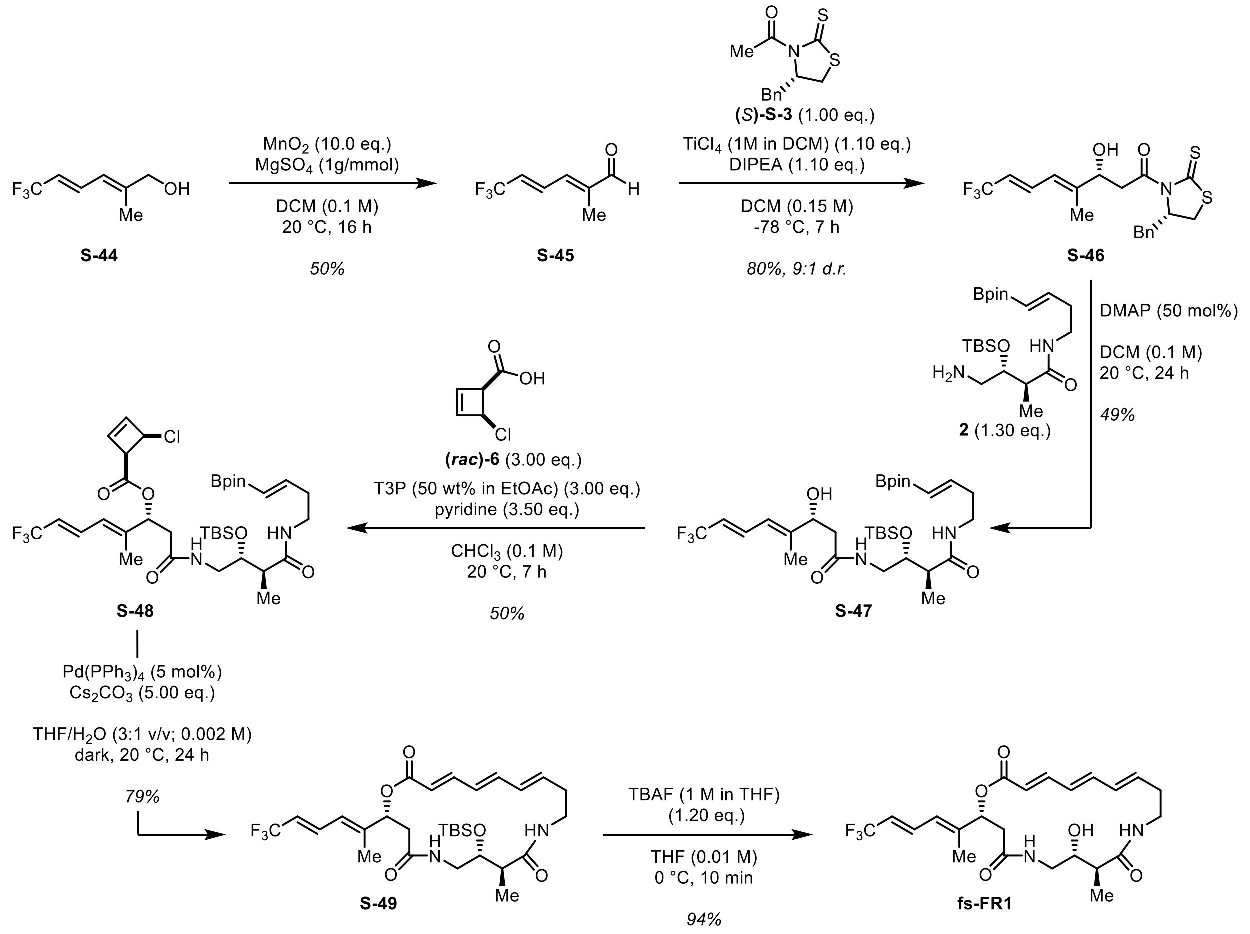


### 4.1.1. (2*E*,4*E*)-6,6,6-Trifluoro-2-methylhexa-2,4-dienal (S-45)

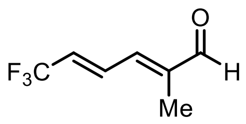

Synthesized according to **General Procedure S** on 3.37 mmol scale to yield **S-45** (277 mg, 1.69 mmol, 50%) as a colorless, volatile liquid.

All spectroscopic data in accordance with the literature.[1]

**$^1$H NMR** (400 MHz, $CDCl_3$): δ 9.54 (s, 1H), 7.20 (ddd, *J* = 15.3, 11.4, 1.9 Hz, 1H), 6.84 (d, *J* = 11.3 Hz, 1H), 6.12 (dq, *J* = 13.6, 6.7 Hz, 1H), 1.94 (s, 3H) ppm.

### 4.1.2. (*R*,4*E*,6*E*)-1-((*S*)-4-Benzyl-2-thioxothiazolidin-3-yl)-8,8,8-trifluoro-3-hydroxy-4-methylocta-4,6-dien-1-one (S-46)

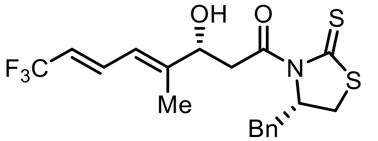


Synthesized according to **General Procedure K** on 1.65 mmol scale to yield **S-46** (549 mg, 1.32 mmol, 80%, 9:1 d.r.) as a yellow oil.

All spectroscopic data in accordance with the literature.[1]

**$^1$H NMR** (400 MHz, $CDCl_3$): δ 7.38 – 7.33 (m, 2H), 7.31 – 7.27 (m, 3H), 7.02 (ddq, *J* = 15.3, 11.0, 6.5 Hz, 1H), 6.25 (d, *J* = 11.3 Hz, 1H), 5.72 (dq, *J* = 13.9, 6.5 Hz, 1H), 5.41 (ddd, *J* = 10.7, 7.0, 4.1 Hz, 1H), 4.67 (d, *J* = 9.4 Hz, 1H), 3.72 (dd, *J* = 17.6, 2.4 Hz, 1H), 3.42 (dd, *J* = 11.8, 7.4 Hz, 1H), 3.31 – 3.22 (m, 2H), 3.06 (dd, *J* = 13.1, 10.5 Hz, 1H), 2.96 – 2.86 (m, 2H), 1.88 (s, 3H) ppm.

### 4.1.3. (*R*,4*E*,6*E*)-*N*-((2*R*,3*S*)-2-((*tert*-Butyldimethylsilyl)oxy)-3-methyl-4-oxo-4-(((*E*)-4-(4,4,5,5-tetramethyl-1,3,2-dioxaborolan-2-yl)but-3-en-1-yl)amino)butyl)-8,8,8-trifluoro-3-hydroxy-4-methylocta-4,6-dienamide (S-47)

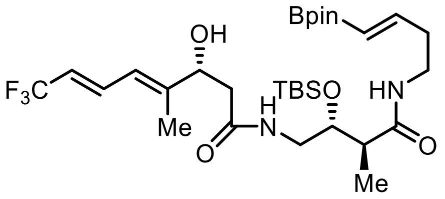


Synthesized according to **General Procedure L** on 1.32 mmol scale to yield **S-47** (411 mg, 0.650 mmol, 49%) as a colorless foam.

All spectroscopic data in accordance with the literature.[1]

**$^1$H NMR** (600 MHz, $CDCl_3$): δ 6.99 (t, *J* = 13.5 Hz, 1H), 6.60 (br. s, 1H), 6.55 (dt, *J* = 17.8, 6.4 Hz, 1H), 6.20 – 6.27 (m, 2H), 5.71 – 5.66 (m, 1H), 5.52 (d, *J* = 18.0 Hz, 1H), 4.48 (d, *J* = 8.9 Hz, 1H), 4.36 (s, 1H), 3.88 – 3.82

(m, 1H), 3.71 – 3.63 (m, 1H), 3.33 – 3.23 (m, 1H), 2.96 – 2.88 (m, 1H), 2.46 – 2.32 (m, 4H), 2.20 (br. s, 1H), 2.03 (br. s, 1H), 1.83 (s, 3H), 1.25 (s, 12H), 1.14 (d, *J* = 7.2 Hz, 3H), 0.89 (s, 9H), 0.15 (s, 3H), 0.09 (s, 3H) ppm.

#### 4.1.4. (*R*,4*E*,6*E*)-1-(((2*R*,3*S*)-2-((*tert*-Butyldimethylsilyl)oxy)-3-methyl-4-oxo-4-(((*E*)-4-(4,4,5,5-tetramethyl-1,3,2-dioxaborolan-2-yl)but-3-en-1-yl)amino)butyl)amino)-8,8,8-trifluoro-4-methyl-1-oxoocta-4,6-dien-3-yl (1*R*,4*R*)-4-chlorocyclobut-2-ene-1-carboxylate (S-48)

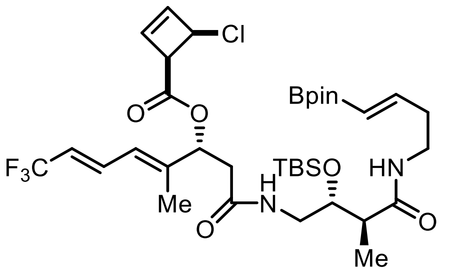


Synthesized according to **General Procedure M** on 237 µmol scale to yield **S-48** (88.5 mg, 118 µmol, 50%) as a colorless foam.

All spectroscopic data in accordance with the literature.[1]

**$^1$H NMR** (600 MHz, $CDCl_3$): δ 6.98 – 6.89 (m, 1H), 6.54 (dt, *J* = 18.0, 6.5 Hz, 1H), 6.39 (br. s, 1H), 6.29 – 6.22 (m, 4H), 5.75 – 5.66 (m, 2H), 5.51 (d, *J* = 18.0 Hz, 1H), 5.10 (d, *J* = 4.3 Hz, 1H), 4.10 (d, *J* = 4.3 Hz, 1H), 3.82 – 3.72 (m, 2H), 3.47 – 3.42 (m, 1H), 3.30 – 3.24 (m, 1H), 2.80 – 2.74 (m, 1H), 2.64 (dd, *J* = 14.6, 8.0 Hz, 1H), 2.52 (dd, *J* = 14.6, 5.2 Hz, 1H), 2.43 – 2.33 (m, 3H), 1.88 (s, 3H), 1.25 (s, 12H), 1.13 (d, *J* = 7.2 Hz, 3H), 0.90 (s, 9H), 0.16 (s, 3H), 0.08 (s, 3H) ppm.

#### 4.1.5. (2*R*,7*R*,8*S*,13*E*,15*E*,17*E*)-7-((*tert*-Butyldimethylsilyl)oxy)-8-methyl-2-((2*E*,4*E*)-6,6,6-trifluorohexa-2,4-dien-2-yl)-1-oxa-5,10-diazacyclononadeca-13,15,17-triene-4,9,19-trione (S-49)

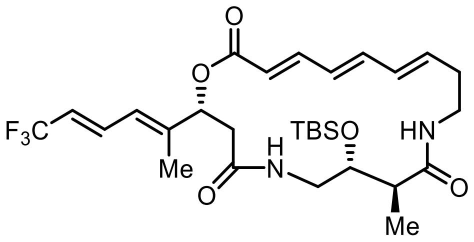


Synthesized according to **General Procedure N** on 12.6 µmol scale to yield **S-49** (5.8 mg, 9.92 µmol, 79%) as a colorless foam.

All spectroscopic data in accordance with the literature.[1]

**$^1$H NMR** (600 MHz, $CDCl_3$): δ 7.28 (dd, *J* = 15.3, 11.2 Hz, 1H), 7.02 – 6.96 (m, 1H), 6.47 (dd, *J* = 14.8, 10.9 Hz, 1H), 6.19 – 6.08 (m, 2H), 5.98 (br. s, 1H), 5.92 (m, 1H), 5.75 – 5.70 (m, 2H), 5.65 (d, *J* = 8.6 Hz, 1H), 5.37 – 5.31 (m, 2H), 4.10 – 4.02 (m, 2H), 3.52 – 3.45 (m, 1H), 2.97 (dm, *J* = 13.6 Hz, 1H), 2.60 (dd, *J* = 13.6, 3.4 Hz, 1H), 2.55 (dm, *J* = 13.9 Hz, 1H), 2.47 (m, 1H), 2.40 (dt, *J* = 13.7, 4.7 Hz, 1H), 2.35 (dd, *J* = 13.5, 11.5 Hz, 1H), 2.25 – 2.19 (m, 1H), 2.04 – 1.97 (m, 1H), 1.89 (s, 3H), 1.13 (d, *J* = 7.2 Hz, 3H), 0.87 (s, 9H), 0.07 (s, 3H), 0.05 (s, 3H) ppm.

### 4.1.6. Fs-FR1

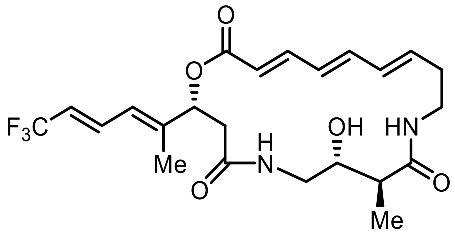


Synthesized according to **General Procedure O** on 9.50 µmol scale to yield **Fs-FR1** (4.2 mg, 8.93 µmol, 94%) as a white powder.

All spectroscopic data in accordance with the literature.[1]

**$^1$H NMR** (600 MHz, $CDCl_3$): δ 7.30 (dd, *J* = 15.3, 11.2 Hz, 1H), 7.02 – 6.96 (m, 1H), 6.53 (dd, *J* = 14.9, 10.8 Hz, 1H), 6.29 (d, *J* = 8.8 Hz, 1H), 6.22 – 6.18 (m, 2H), 6.08 (dd, *J* = 15.0, 11.0 Hz, 1H), 5.99 (s, 1H), 5.80 (ddd, *J* = 15.5, 11.6, 3.8 Hz, 1H), 5.77 (d, *J* = 15.4 Hz, 1H), 5.70 – 5.67 (m, 3H), 4.20 – 4.10 (m, 1H), 3.83 (t, *J* = 10.9 Hz, 1H), 3.32 (td, *J* = 10.4, 2.7 Hz, 1H), 3.02 (dm, *J* = 14.3 Hz, 1H), 2.65 – 2.59 (m, 3H), 2.45 (dd, *J* = 13.8, 11.7 Hz, 1H), 2.28 – 2.20 (m, 2H), 1.91 (s, 3H), 0.99 (d, *J* = 7.2 Hz, 3H) ppm.

## 4.2. Fs-FR2 Synthesis

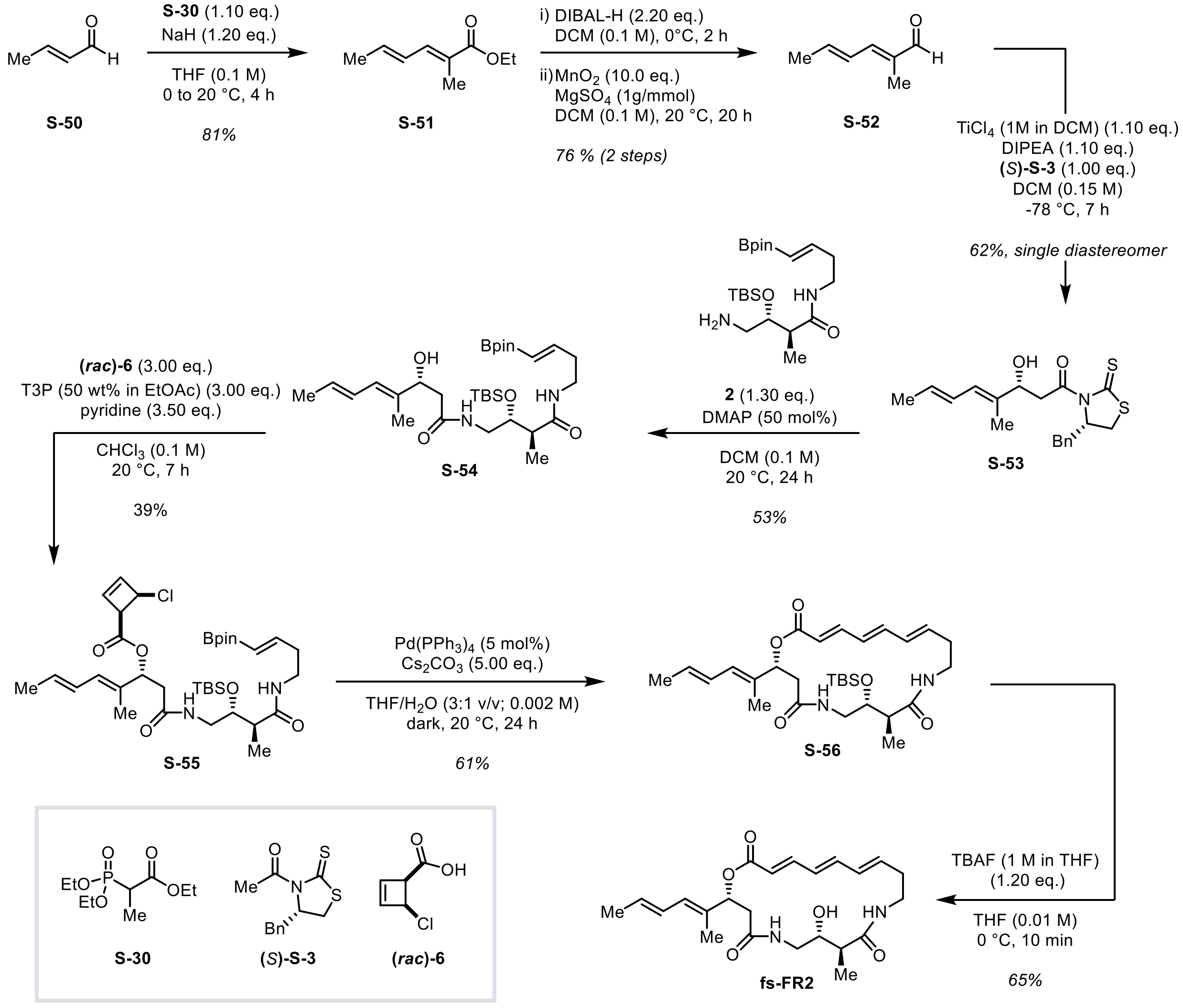


### 4.2.1. Ethyl (2*E*,4*E*)-2-methylhexa-2,4-dienoate (S-51)

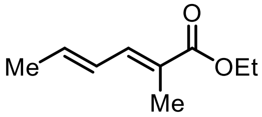

Synthesized according to the **General Procedure Q** on 18.2 mmol scale to yield **S-51** (2.25 g, 14.6 mmol, 81%) as a colorless oil.

**$^{1}$H-NMR** (600 MHz, $CDCl_3$): δ 7.15 (d, *J* = 11.3 Hz, 1H), 6.36 (ddq, *J* = 14.6, 11.3 ,1.6 Hz, 1H), 6.12 – 6.06 (m, 1H), 4.20 (q, *J* = 7.1 Hz, 2H), 1.92 (s, 3H), 1.87 (d, *J* = 6.8 Hz, 3H), 1.30 (t, *J* = 7.1 Hz, 3H) ppm.

**$^{13}$C-NMR** (151 MHz, $CDCl_3$): δ 168.9, 138.6, 137.7, 127.6, 125.1, 60.6, 19.0, 14.5, 12.7 ppm.

**HRMS (ESI$^+$):** calculated for $[M+Na]^+$ ($C_9H_{14}O_2Na^+$) required *m/z* 177.0892, found *m/z* 177.0894.

**FT-IR (neat)** $\nu_{max}$: 2983, 2957, 2927, 2853, 1740, 1702, 1642, 1444, 1390, 1367, 1290, 1230, 1167, 1101, 968, 745, 732 cm$^{-1}$.

### 4.2.2. (2*E*,4*E*)-2-Methylhexa-2,4-dien-1-ol (S-51a)

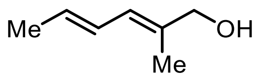

Synthesized according to the **General Procedure R** on 20.0 mmol scale to yield **S-51a** (2.21 g, 19.7 mmol, 99%) as a colorless oil.

All spectroscopic data in accordance with the literature.[4]

**$^1$H-NMR** (400 MHz, CDCl$_3$): δ 6.35 – 6.21 (m, 1H), 6.02 (d, *J* = 10.8 Hz, 1H), 5.71 (dq, *J* = 13.5, 6.6 Hz, 1H), 4.05 (d, *J* = 5.2 Hz, 2H), 1.81 – 1.77 (m, 6H) ppm.

### 4.2.3. (2*E*,4*E*)-2-Methylhexa-2,4-dienal (S-52)

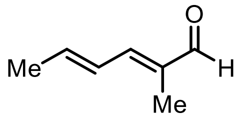

Synthesized according to the **General Procedure S** on 10.0 mmol scale to yield **S-52** (841 mg, 7.63 mmol, 76%) as a colorless, volatile oil.

All spectroscopic data in accordance with the literature.[5]

**$^1$H-NMR** (400 MHz, CDCl$_3$): δ 9.41(s, 1H), 6.81 (d, *J* = 11.1 Hz, 1H), 6.57 – 6.51 (m, 1H), 6.25 (dq, *J* = 14.0, 9.5 Hz, 1H), 1.93 (d, *J* = 6.9 Hz, 3H), 1.82 (s, 3H) ppm.

### 4.2.4. ((*R*,4*E*,6*E*)-1-((*S*)-4-Benzyl-2-thioxothiazolidin-3-yl)-3-hydroxy-4-methylocta-4,6-dien-1-one (S-53)

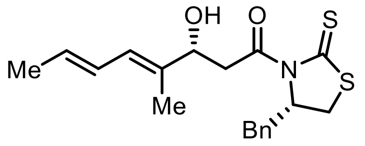

Synthesized according to the **General Procedure K** on 3.00 mmol scale to yield **S-53** (677 mg, 1.87 mmol, 62%, single diastereomer isolated) as a yellow oil.

**$^1$H-NMR** (600 MHz, CDCl$_3$): δ 7.37 – 7.34 (m, 2H), 7.30 – 7.27 (d, *J* = 7.3 Hz, 3H), 6.27 (ddd, *J* = 14.9, 10.9, 1.6 Hz, 1H), 6.10 (d, *J* = 11.0 Hz, 1H), 5.74 (dq, *J* = 13.4, 6.6 Hz, 1H), 5.38 (ddd, *J* = 10.8, 7.1, 4.0 Hz, 1H), 4.63

(d, $J$ = 9.6 Hz, 1H), 3.56 (dd, $J$ = 17.4, 2.6 Hz, 1H), 3.44 – 3.39 (m, 2H, 3.24 (dd, $J$ = 13.2, 3.9 Hz, 1H), 3.05 (dd, $J$ = 13.2, 10.5 Hz, 1H), 2.90 (d, $J$ = 11.5 Hz, 1H), 2.54 (d, $J$ = 3.5 Hz, 1H), 1.80-1.77 (m, 6H) ppm.

**$^{13}$C-NMR** (151 MHz, $CDCl_3$): δ 201.5, 173.0, 136.6, 135.0, 130.5, 129.6, 129.1, 127.4, 127.3, 125.8, 72.9, 68.6, 44.5, 37.0, 32.3, 18.6, 13.0 ppm.

**HRMS (ESI$^+$):** calculated for $[M+Na]^+$ ($C_{19}H_{23}NO_2S_2Na^+$) required *m/z* 384.1062, found *m/z* 384.1061.

**FT-IR (neat) $ν_{max}$:** 3510, 3474, 3025, 2924, 2852, 1693, 1495, 1453, 1437, 1342, 1318, 1292, 1257, 1227, 1191, 1163, 1136, 1092, 1043, 964, 894, 748, 723, 702, 620, 565, 555, 510, 482, 466 cm$^{-1}$.

**[a]$_D^{20}$** = +59.2° (c = 1.00, $CHCl_3$).

#### 4.2.5. (*R*,4*E*,6*E*)-*N*-((2*R*,3*S*)-2-((*tert*-Butyldimethylsilyl)oxy)-3-methyl-4-oxo-4-(((*E*)-4-(4,4,5,5-tetramethyl-1,3,2-dioxaborolan-2-yl)but-3-en-1-yl)amino)butyl)-3-hydroxy-4-methylocta-4,6-dienamide (S-54)

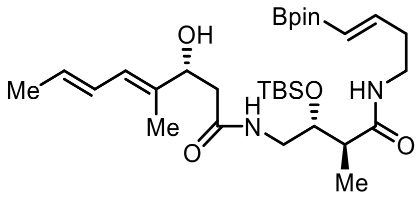


Synthesized according to the **General Procedure L** on 1.82 mmol scale to yield **S-54** (557 mg, 0.963 mmol, 53%) as a colorless foam.

**$^1$H-NMR** (600 MHz, $CDCl_3$): δ 6.55 (dt, $J$ = 17.9, 6.6 Hz, 1H), 6.42 – 6.40 (m, 1H), 6.25 (ddd, $J$ = 14.9, 10.9, 1.5 Hz, 1H), 6.12 (t, $J$ = 5.4 Hz, 1H), 6.07 (d, $J$ = 10.8 Hz, 1H), 5.71 (dq, $J$ = 13.6, 6.7 Hz, 1H), 5.52 (d, $J$ = 18.0 Hz, 1H), 4.43 (d, $J$ = 8.8 Hz, 1H), 3.90 – 3.86 (m, 1H), 3.72 (ddd $J$ = 13.7, 7.6, 4.2 Hz, 1H), 3.49 (d, $J$ = 2.7 Hz, 1H), 3.44 (td, $J$ = 13.4, 6.7 Hz, 1H), 3.27 (dt, $J$ = 12.0, 6.5 Hz, 1H), 2.93 (ddd, $J$ = 13.5, 7.5, 4.4 Hz, 1H), 2.47 – 2.32 (m, 5H), 1.78 (d, $J$ = 6.6 Hz, 3H), 1.74 (s, 3H), 1.25 (s, 12H), 1.14 – 1.13 (d, $J$ = 7.2 Hz, 3H), 0.90 (s, 9H), 0.15 – 0.07 (m, 6H) ppm.

**$^{13}$C-NMR** (151 MHz, $CDCl_3$): δ 174.4, 172.1, 150.4, 135.3, 130.2, 127.3, 125.6, 121.6 (*deduced from HSQC*), 83.3 (2C), 73.5, 72.0, 44.7, 42.8, 41.5, 37.8, 35.6, 25.9 (3C), 25.0, 24.9 (2C), 24.8 (2C), 18.4, 15.2, 12.6, −4.54, −5.00 ppm.

**HRMS (ESI$^+$):** calculated for $[M+Na]^+$ ($C_{30}H_{55}BN_2O_6SiNa^+$) required *m/z* 601.3820, found *m/z* 601.3829.

**FT-IR (neat) $ν_{max}$:** 3309, 2976, 2953, 2929, 2884, 2856, 1642, 1541, 1471, 1462, 1446, 1397, 1389, 1360, 1322, 1255, 1144, 1102, 1028, 1004, 967, 937, 884, 837, 812, 778, 679, 666, 641, 623 cm$^{-1}$.

**[a]$_D^{20}$** = +30.1° (c = 1.00, $CHCl_3$).

### 4.2.6. (*R*,4*E*,6*E*)-1-(((2*R*,3*S*)-2-((*tert*-Butyldimethylsilyl)oxy)-3-methyl-4-oxo-4-(((*E*)-4-(4,4,5,5-tetramethyl-1,3,2-dioxaborolan-2-yl)but-3-en-1-yl)amino)butyl)amino)-4-methyl-1-oxoocta-4,6-dien-3-yl (1*R*,4*R*)-4-chlorocyclobut-2-ene-1-carboxylate (S-55)

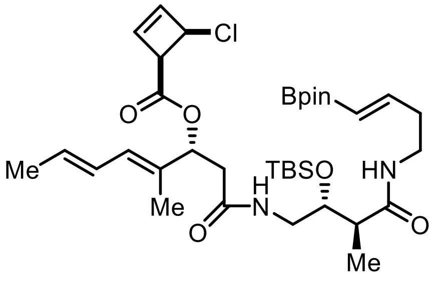


Synthesized according to the **General Procedure M** on 0.200 mmol scale to yield **S-55** (54.0 mg, 77.9 µmol, 39%) as a colorless foam.

**$^1$H-NMR** (600 MHz, $CDCl_3$): δ 6.55 (dt, *J* = 18.0, 6.5 Hz, 1H), 6.29 – 6.24 (m, 2H), 6.22 – 6.17 (m, 2H), 6.19 – 6.15 (m, 1H), 6.12 (d, *J* = 11.0 Hz, 1H), 5.74 (dq, *J* = 13.3, 6.6 Hz, 1H), 5.63 (dd, *J* = 7.5, 5.9 Hz, 1H), 5.51 (dt, *J* = 17.9, 1.3 Hz, 1H), 5.08 (d, *J* = 4.3 Hz, 1H), 4.07 (dd, *J* = 4.3, 0.7 Hz, 1H), 3.82 – 3.79 (m, 1H), 3.79 – 3.74 (m, 1H), 3.42 (td, *J* = 13.3, 6.9 Hz, 1H), 3.27 (qd, *J* = 12.3, 6.7 Hz, 1H), 2.82 – 2.76 (m, 1H), 2.67 (dd, *J* = 14.4, 7.8 Hz, 1H), 2.50 (dd, *J* = 14.3, 5.7 Hz, 1H), 2.36 (m, 3H), 1.76 (d, *J* = 7.9 Hz, 6H), 1.25 (s, 12H), 1.10 (d, *J* = 7.2 Hz, 3H), 0.90 (s, 9H), 0.15 (s, 3H), 0.08 (s, 3H) ppm.

**$^{13}$C-NMR** (151 MHz, $CDCl_3$): δ 174.7, 169.3, 168.8, 150.3, 140.6, 136.6, 131.5, 130.7, 128.9, 127.0, 121.4 (*deduced from HSQC*), 83.4 (2C), 77.0, 72.1, 56.4, 54.0, 44.5, 43.0, 40.9, 38.0, 35.8, 26.0 (3C), 24.9 (4C), 18.6, 18.1, 15.5, 12.7, −4.3, −4.9 ppm.

**HRMS (ESI$^+$):** calculated for $[M+Na]^+$ ($C_{35}H_{58}BClN_2O_7SiNa^+$) required *m/z* 715.3692, found *m/z* 715.3695.

**FT-IR (neat) $\nu_{max}$:** 3345, 2930, 2857, 1733, 1642, 1536, 1470, 1358, 1322, 1288, 1257, 1234, 1168, 1141, 908, 727 cm$^{-1}$.

**$[a]_D^{20}$** = +16.6° (c = 1.30, $CHCl_3$).

### 4.2.7. (2*R*,7*R*,8*S*,13*E*,15*E*,17*E*)-7-((*tert*-Butyldimethylsilyl)oxy)-2-((2*E*,4*E*)-hexa-2,4-dien-2-yl)-8-methyl-1-oxa-5,10-diazacyclononadeca-13,15,17-triene-4,9,19-trione (S-56)

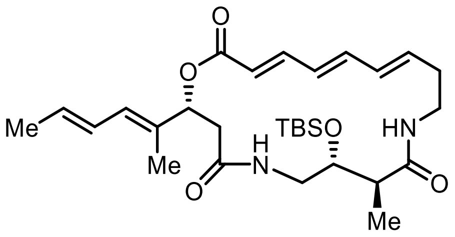


Synthesized according to the **General Procedure N** on 33.0 µmol scale to yield **S-56** (10.6 mg, 20.0 µmol, 61%) as a colorless foam.

**$^{1}$H-NMR** (600 MHz, $CDCl_3$): δ 7.27 – 7.22 (m, 1H), 6.46 (dd, *J* = 14.9, 10.9 Hz, 1H), 6.25 (ddd, *J* = 15.1, 10.9, 1.5 Hz, 1H), 6.16 (dd, *J* = 14.9, 11.2 Hz, 1H), 6.13 – 6.06 (m, 2H), 6.01 (d, *J* = 10.2 Hz, 1H), 5.94 – 5.86 (m, 2H), 5.75 (dd, *J* = 14.8, 7.0 Hz, 1H), 5.71 (d, *J* = 15.4 Hz, 1H), 5.62 (dd, *J* = 11.1, 3.1 Hz, 1H), 4.11 – 4.02 (m, 1H), 4.00 (s, 1H), 3.47 (dt, *J* = 13.9, 8.0 Hz, 1H), 2.96 (d, *J* = 13.9 Hz, 1H), 2.56 – 2.50 (m, 2H), 2.47 (ddd, *J* = 14.8, 7.3, 3.2 Hz, 1H), 2.43 – 2.40 (m, 1H), 2.24 – 2.13 (m, 1H), 1.81 – 1.77 (m, 6H), 1.13 (d, *J* = 7.3 Hz, 3H), 0.87 (s, 9H), 0.08 (s, 3H), 0.05 (s, 3H) ppm.

**$^{13}$C NMR** (151 MHz, $CDCl_3$): δ 174.2, 169.9, 166.4, 146.6, 143.2, 138.8, 132.0, 131.6, 131.0, 127.6, 127.1, 126.4, 119.6, 76.3 (*deduced from HSQC*), 72.4, 45.3, 43.0, 42.3, 37.5, 34.5, 25.9 (3C), 18.6, 18.0, 13.4, 12.5 (*deduced from HSQC*), −4.5, −4.8 ppm.

**HRMS (ESI$^+$):** calculated for $[M+Na]^+$ ($C_{29}H_{46}N_2O_5SiNa^+$) required *m/z* 553.3074, found *m/z* 553.3065.

**FT-IR (neat) $\nu_{max}$:** 3385, 2955, 2927, 2854, 1712, 1646, 1552, 1540, 1467, 1437, 1389, 1361, 1259, 1240, 1218, 1094, 1052, 1021, 985, 965, 873, 838 cm$^{-1}$.

**$[a]_D^{20}$** = −142° (c = 0.45, $CHCl_3$).

### 4.2.8. Fs-FR2

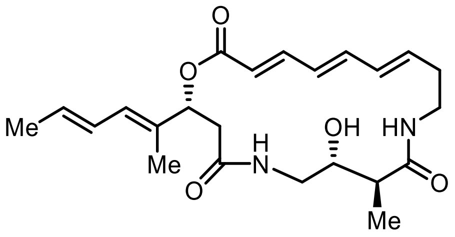


Synthesized according to the **General Procedure O** on 20.0 µmol scale to yield **Fs-FR2** (5.40 mg, 13.0 µmol, 65%) as a white solid.

**$^1$H-NMR** (600 MHz, $CDCl_3$): δ 7.29 – 7.23 (m, 1H), 6.51 (dd, *J* = 14.9, 10.8 Hz, 1H), 6.22 (ddd, *J* = 25.9, 15.0, 9.7 Hz, 3H), 6.13 (d, *J* = 10.9 Hz, 1H), 6.07 (dd, *J* = 14.9, 11.2 Hz, 1H), 5.81 – 5.64 (m, 5H), 4.13 (ddd, *J* = 23.5, 13.4, 5.1 Hz, 1H), 3.82 – 3.76 (m, 1H), 3.36 – 3.30 (m, 1H), 3.01 (d, *J* = 15.0 Hz, 1H), 2.66 – 2.47 (m, 4H), 2.29 – 2.18 (m, 2H), 1.78 (app. d, *J* = 7.4 Hz, 6H), 0.98 (d, *J* = 7.1 Hz, 3H) ppm.

**$^{13}$C-NMR** (151 MHz, $CDCl_3$): δ 176.0, 169.4, 165.4, 144.6, 140.8, 136.0, 133.0, 131.8, 130.9, 128.7, 127.1, 126.6, 121.3, 76.1,71.6, 44.1, 43.1, 42.0, 36.9, 34.6, 18.6, 13.1, 11.1 ppm.

**HRMS (ESI$^+$):** calculated for $[M+Na]^+$ ($C_{23}H_{32}N_2O_5Na^+$) required *m/z* 439.2209, found *m/z* 439.2206.

**FT-IR (neat) $\nu_{max}$:** 2957, 2922, 2852, 1709, 1648, 1639, 1613, 1552, 1537, 1465, 1435, 1377, 1338, 1299, 1259, 1233, 1214, 1101, 1062, 1008, 964, 927, 884, 836 cm$^{-1}$.

**[a]$_D^{20}$** = –10.0°(c = 0.1, $CHCl_3$)

## 4.3. Fs-FR3 Synthesis

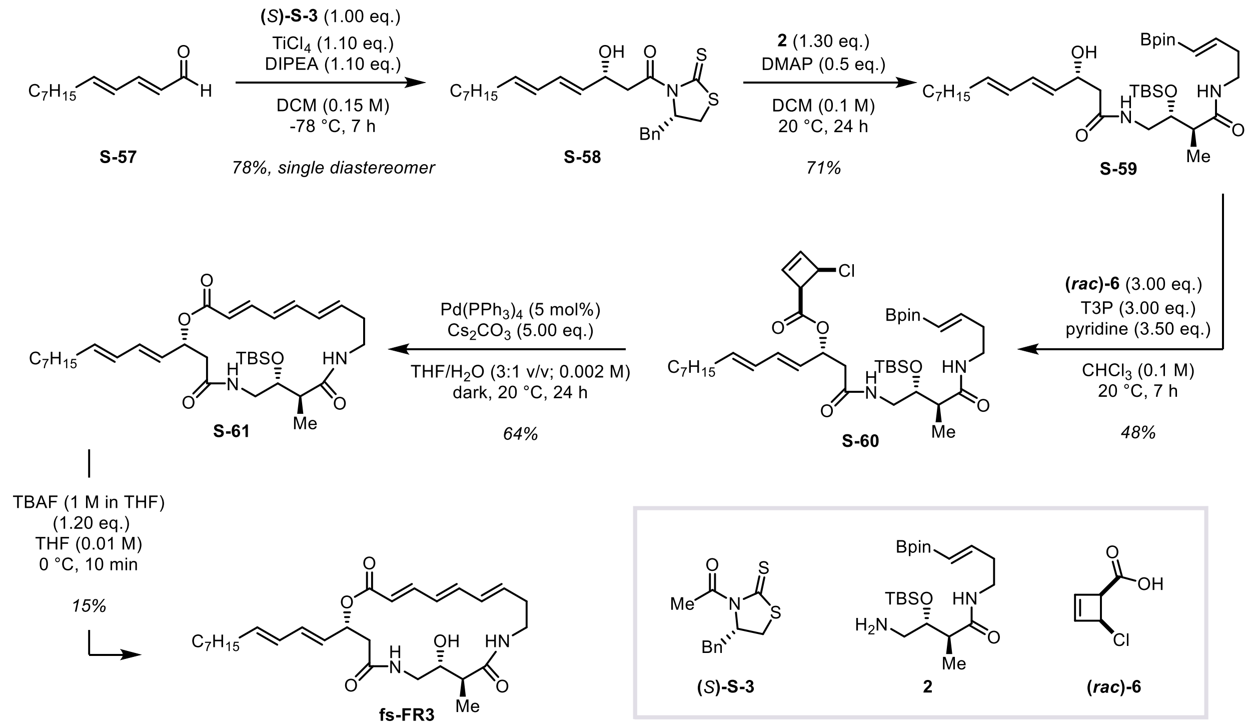


### 4.3.1. (*R*,4*E*,6*E*)-1-((*S*)-4-Benzyl-2-thioxothiazolidin-3-yl)-3-hydroxytetradeca-4,6-dien-1-one (S-58)

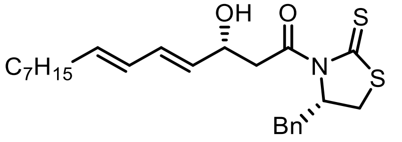

Synthesized according to the **General Procedure K** on 5.03 mmol scale to yield **S-58** (1.69 g, 3.92 mmol, 78%) as a yellow oil.

**$^1$H-NMR** (400 MHz, $CDCl_3$): δ 7.37 – 7.32 (m, 2H), 7.31 – 7.27 (m, 3H), 6.27 (dd, *J* = 15.4, 10.3 Hz, 1H), 6.03 (dd, *J* = 14.9, 10.6 Hz, 1H), 5.78 – 5.69 (m, 1H), 5.64 (dd, *J* = 15.3, 6.2 Hz, 1H), 5.39 (ddd, *J* = 10.6, 6.8, 4.0 Hz, 1H), 4.72 (app. t, *J* = 7.0 Hz, 1H), 3.63 (dd, *J* = 17.6, 3.0 Hz, 1H), 3.44 – 3.32 (m, 2H), 3.23 (dd, *J* = 13.2, 3.9 Hz, 1H), 3.05 (dd, *J* = 13.2, 10.5 Hz, 1H), 2.90 (d, *J* = 11.5 Hz, 1H), 2.67 (br. s, 1H), 2.08 (app. q, *J* = 6.9 Hz, 2H), 1.41 – 1.34 (m, 2H), 1.33 – 1.22 (m, 8H), 0.88 (t, *J* = 6.9 Hz, 3H) ppm.

**$^{13}$C-NMR** (101 MHz, $CDCl_3$): δ 201.5, 172.7, 136.6, 136.5, 131.6, 130.9, 129.6 (2C), 129.4, 129.1 (2C), 127.4, 68.6, 68.5, 45.9, 37.0, 32.8, 32.3, 32.0, 29.3, 29.3, 29.3, 22.8, 14.2 ppm.

**HRMS (ESI$^+$):** calculated for $[M+Na]^+$ ($C_{24}H_{33}NO_2S_2Na^+$) requires *m/z* 454.1845, found *m/z* 454.1845.

**FT-IR (neat) $\nu_{max}$:** 3728, 3705, 3625, 3597, 2923, 2852, 1691, 1436, 1342, 1258, 1163, 1042, 989, 720, 201 $cm^{-1}$.

$[a]_D^{20}$ = +34.3°(c = 0.1, $CHCl_3$)

### 4.3.2. (*R*,4*E*,6*E*)-*N*-((2*R*,3*S*)-2-((*tert*-Butyldimethylsilyl)oxy)-3-methyl-4-oxo-4-(((*E*)-4-(4,4,5,5-tetramethyl-1,3,2-dioxaborolan-2-yl)but-3-en-1-yl)amino)butyl)-3-hydroxytetradeca-4,6-dienamide (S-59)

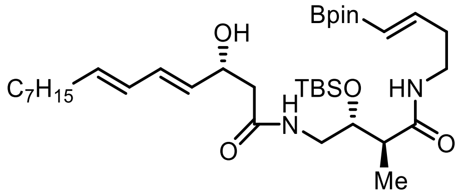


Synthesized according to the **General Procedure L** on 1.08 mmol scale to yield **S-59** (498 mg, 0.768 mmol, 71%) as a yellow foam.

**$^1$H-NMR** (600 MHz, $CDCl_3$): δ 6.69 (s, 1H), 6.52 (dt, *J* = 18.0, 6.5 Hz, 1H), 6.28 (s, 1H), 6.21 (dd, *J* = 15.2, 10.5 Hz, 1H), 5.97 (dd, *J* = 15.0, 10.6 Hz, 1H), 5.72 – 5.63 (m, 1H), 5.54 (dd, *J* = 15.2, 6.3 Hz, 1H), 5.49 (dd, *J* = 18.0, 1.0 Hz, 1H), 4.51 (s, 1H), 3.98 (s, 1H), 3.89 – 3.80 (m, 1H), 3.73 – 3.64 (m, 1H), 3.40 (td, *J* = 13.1, 6.6 Hz, 1H), 3.25 (td, *J* = 12.5, 6.1 Hz, 1H), 2.94 – 2.85 (m, 1H), 2.46 – 2.39 (m, 2H), 2.38 – 2.31 (m, 3H), 2.04 (dd, *J* = 14.4, 7.2 Hz, 2H), 1.37 – 1.30 (m, 2H), 1.28 – 1.19 (m, 20H), 1.11 (d, *J* = 7.2 Hz, 3H), 0.90 – 0.83 (m, 12H), 0.13 (s, 3H), 0.07 (s, 3H) ppm.

**$^{13}$C-NMR** (151 MHz, $CDCl_3$): δ 174.7, 172.0, 150.3, 136.1, 131.5, 131.2, 129.4, 121.6 (*deduced from HSQC*), 83.4, 72.2, 69.2, 44.6, 43.0, 42.9, 37.9, 35.7, 32.7, 31.9, 29.3, 29.2, 29.2, 26.0 (3C), 24.9 (2C), 24.9 (2C), 22.7, 18.0, 15.5, 14.2, −4.4, −4.9 ppm.

**HRMS (ESI$^+$):** calculated for $[M+Na]^+$ ($C_{35}H_{65}BN_2O_6SiNa^+$) requires *m/z* 671.4597, found *m/z* 671.4607.

**FT-IR (neat) $\nu_{max}$**: 3330, 3094, 2954, 2927, 2856, 1640, 1539, 1464, 1398, 1358, 1321, 1254, 1185, 1143, 1104, 1036, 990, 970, 952, 836, 680 $cm^{-1}$.

$[a]_D^{20}$ = +30.8°(c = 0.1, $CHCl_3$)

### 4.3.3. (*R*,4*E*,6*E*)-1-(((2*R*,3*S*)-2-((*tert*-Butyldimethylsilyl)oxy)-3-methyl-4-oxo-4-(((*E*)-4-(4,4,5,5-tetramethyl-1,3,2-dioxaborolan-2-yl)but-3-en-1-yl)amino)butyl)amino)-1-oxotetradeca-4,6-dien-3-yl (1*S*,4*R*)-4-chlorocyclobut-2-ene-1-carboxylate (S-60)

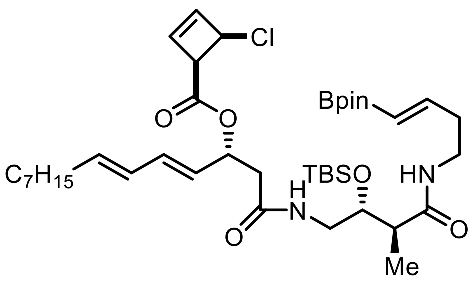


Synthesized according to the **General Procedure M** on 338 µmol scale to yield **S-60** (124 mg, 162 µmol, 48%, mixture of *cis*-diastereomers) as a colorless foam.

**$^1$H-NMR** (600 MHz, $CDCl_3$): δ 6.54 (dt, *J* = 18.1, 6.5 Hz, 1H), 6.33 – 6.20 (m, 5H), 6.00 – 5.92 (m, 1H), 5.76 – 5.68 (m, 2H), 5.54 (dt, *J* = 28.3, 12.4 Hz, 2H), 5.09 (d, *J* = 4.3 Hz, 1H), 4.09 (d, *J* = 4.2 Hz, 1H), 3.83 – 3.78 (m, 1H), 3.74 (ddd, *J* = 18.6, 9.2, 5.2 Hz, 1H), 3.46 – 3.38 (m, 1H), 3.26 (td, *J* = 12.5, 6.6 Hz, 1H), 2.85 – 2.79 (m, 1H), 2.63 (dt, *J* = 13.9, 6.9 Hz, 1H), 2.54 (dd, *J* = 14.4, 5.7 Hz, 1H), 2.40 (m, 1H), 2.35 (app. q, *J* = 6.4 Hz, 2H), 2.04 (app. dd, *J* = 14.1, 7.0 Hz, 2H), 1.38 – 1.32 (m, 2H), 1.25 (s, 20H), 1.10 (d, *J* = 7.2 Hz, 3H), 0.89 (s, 9H), 0.87 (t, *J* = 7.0 Hz, 3H), 0.15 (s, 3H), 0.08 (s, 3H) ppm.

**$^{13}$C NMR** (151 MHz, $CDCl_3$): δ 174.6, 169.2, 168.9, 150.3, 140.6, 137.6, 136.6, 134.4, 129.0, 126.6, 121.6, 83.3 (2C), 72.6, 72.1, 56.5, 54.0, 44.5, 43.1, 42.0, 38.0, 35.8, 32.8, 31.9, 29.3, 29.3, 29.2, 26.0 (3C), 24.9 (2C), 24.9 (2C), 22.8, 18.1, 15.6, 14.2, −4.4, −4.9 ppm.

**HRMS (ESI$^+$):** calculated for $[M+Na]^+$ ($C_{40}H_{68}BClN_2O_7SiNa^+$) requires *m/z* 785.4470, found *m/z* 785.4446.

**FT-IR (neat) $\nu_{max}$:** 3310, 2954, 2928, 2856, 1736, 1644, 1537, 1463, 1438, 1397, 1389, 1359, 1288, 1172, 1143, 1112, 1102, 988, 970, 950, 902, 837, 810, 776, 757, 733, 679, 667, 645 cm$^{-1}$.

### 4.3.4. (2*R*,7*R*,8*S*,13*E*,15*E*,17*E*)-7-((*tert*-Butyldimethylsilyl)oxy)-8-methyl-2-((1*E*,3*E*)-undeca-1,3-dien-1-yl)-1-oxa-5,10-diazacyclononadeca-13,15,17-triene-4,9,19-trione (S-61)

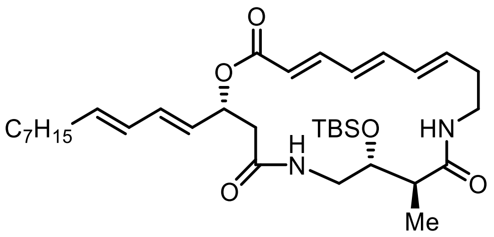

Synthesized according to the **General Procedure N** on 27.3 µmol scale to yield **S-61** (10.0 mg, 17.5 µmol, 64%) as a colorless foam.

**$^1$H-NMR** (700 MHz, $CDCl_3$): δ 7.22 (dd, *J* = 15.4, 11.2 Hz, 1H), 6.44 (dd, *J* = 14.9, 10.9 Hz, 1H), 6.30 (dd, *J* = 15.2, 10.5 Hz, 1H), 6.15 (dd, *J* = 14.9, 11.3 Hz, 1H), 6.11 – 6.07 (m, 1H), 6.01 (dd, *J* = 15.1, 10.5 Hz, 1H), 5.92 – 5.89 (m, 2H), 5.77 – 5.63 (m, 5H), 4.02 (m, 2H), 3.44 – 3.39 (m, 1H), 3.00 (m, 1H), 2.56 – 2.50 (m, 3H), 2.46 (app. dd, *J* = 7.3, 2.9 Hz, 1H), 2.38 (dd, *J* = 13.8, 10.8 Hz, 1H), 2.24 – 2.19 (m, 1H), 2.07 (dd, *J* = 14.4, 7.2 Hz, 2H), 1.37 (d, *J* = 6.7 Hz, 2H), 1.28 (m, 8H), 1.12 (d, *J* = 7.3 Hz, 3H), 0.89 – 0.87 (m, 12H), 0.07 (s, 3H), 0.05 (s, 3H) ppm.

**$^{13}$C NMR** (176 MHz, $CDCl_3$): δ 173.9, 169.4, 166.4, 146.9, 143.2, 138.9, 137.3, 133.1, 131.5, 129.1, 127.5 (2C), 119.5, 73.4, 72.2, 45.5, 43.4, 42.9, 37.5, 34.5, 32.8, 32.0, 29.8, 29.3, 29.3, 25.8 (3C), 22.8, 18.0, 14.7, 14.2, −4.5, −4.7 ppm.

**HRMS (ESI$^+$):** calculated for $[M+Na]^+$ ($C_{34}H_{56}N_2O_5SiNa^+$) requires *m/z* 623.3851, found *m/z* 623.3841.

**FT-IR (neat) $ν_{max}$:** 3321, 2953, 2926, 2855, 1713, 1650, 1540, 1462, 1437, 1377, 1361, 1253, 1175, 1140, 1100, 1004, 989, 939, 912, 865, 835, 774, 723, 712, 703, 667 $cm^{-1}$.

**$[a]_D^{20}$** = −9.2 (c = 0.53, $CHCl_3$)

### 4.3.5. Fs-FR3

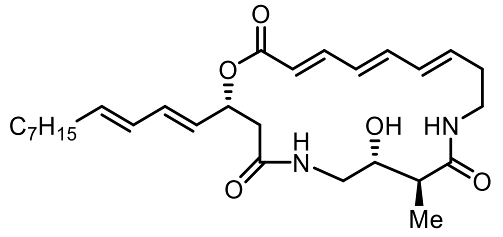


Synthesized according to the **General Procedure O** on 13.7 µmol scale to yield **Fs-FR3** (1.00 mg, 2.06 µmol, 15%) as a white powder.

**$^1$H-NMR** (700 MHz, $CDCl_3$): δ 7.27 – 7.25 (m, 1H), 6.49 (dd, *J* = 14.9, 10.8 Hz, 1H), 6.31 (dd, *J* = 15.3, 10.5 Hz, 1H), 6.26 (d, *J* = 8.5 Hz, 1H), 6.18 (dd, *J* = 14.9, 11.3 Hz, 1H), 6.07 (dd, *J* = 14.8, 11.6 Hz, 1H), 6.00 (dd, *J* = 15.1, 10.5 Hz, 1H), 5.81 – 5.72 (m, 4H), 5.68 (d, *J* = 7.2 Hz, 1H), 5.60 (dd, *J* = 15.3, 6.5 Hz, 1H), 4.10 (ddd, *J* = 17.4, 14.9, 5.1 Hz, 1H), 3.80 (t, *J* = 10.0 Hz, 1H), 3.35 – 3.28 (m, 1H), 3.04 (d, *J* = 13.8 Hz, 1H), 2.65 – 2.60 (m, 1H), 2.58 (dd, *J* = 13.8, 2.8 Hz, 2H), 2.48 (dd, *J* = 13.9, 11.3 Hz, 1H), 2.29 – 2.18 (m, 2H), 2.07 (dd, *J* = 14.0, 6.8 Hz, 2H), 1.39 – 1.35 (m, 2H), 1.31 – 1.24 (m, 8H), 0.98 (d, *J* = 7.1 Hz, 3H), 0.88 (t, *J* = 7.1 Hz, 3H) ppm.

**$^{13}$C NMR** (176 MHz, $CDCl_3$): δ 176.0, 169.2, 165.6, 144.8, 140.9, 137.3, 136.1, 133.3, 132.9, 129.1, 128.6, 127.4, 121.1, 72.3, 71.6, 44.1, 43.1, 43.0, 37.0, 34.6, 32.8, 32.0, 29.3, 29.3, 29.3, 22.8, 14.2, 11.2 ppm.
**HRMS (ESI$^+$):** calculated for $[M+Na]^+$ ($C_{28}H_{42}N_2O_5Na^+$) requires *m/z* 509.2986, found *m/z* 509.2985.
**FT-IR (neat) $\nu_{max}$:** 3314, 2957, 2921, 2851, 1739, 1702, 1645, 1630, 1614, 1540, 1234, 1216, 1009, 774 cm$^{-1}$.
**$[a]_D^{20}$** = −67.1 (c = 0.07, $CHCl_3$)

## 4.4. Fs-FR4 Synthesis

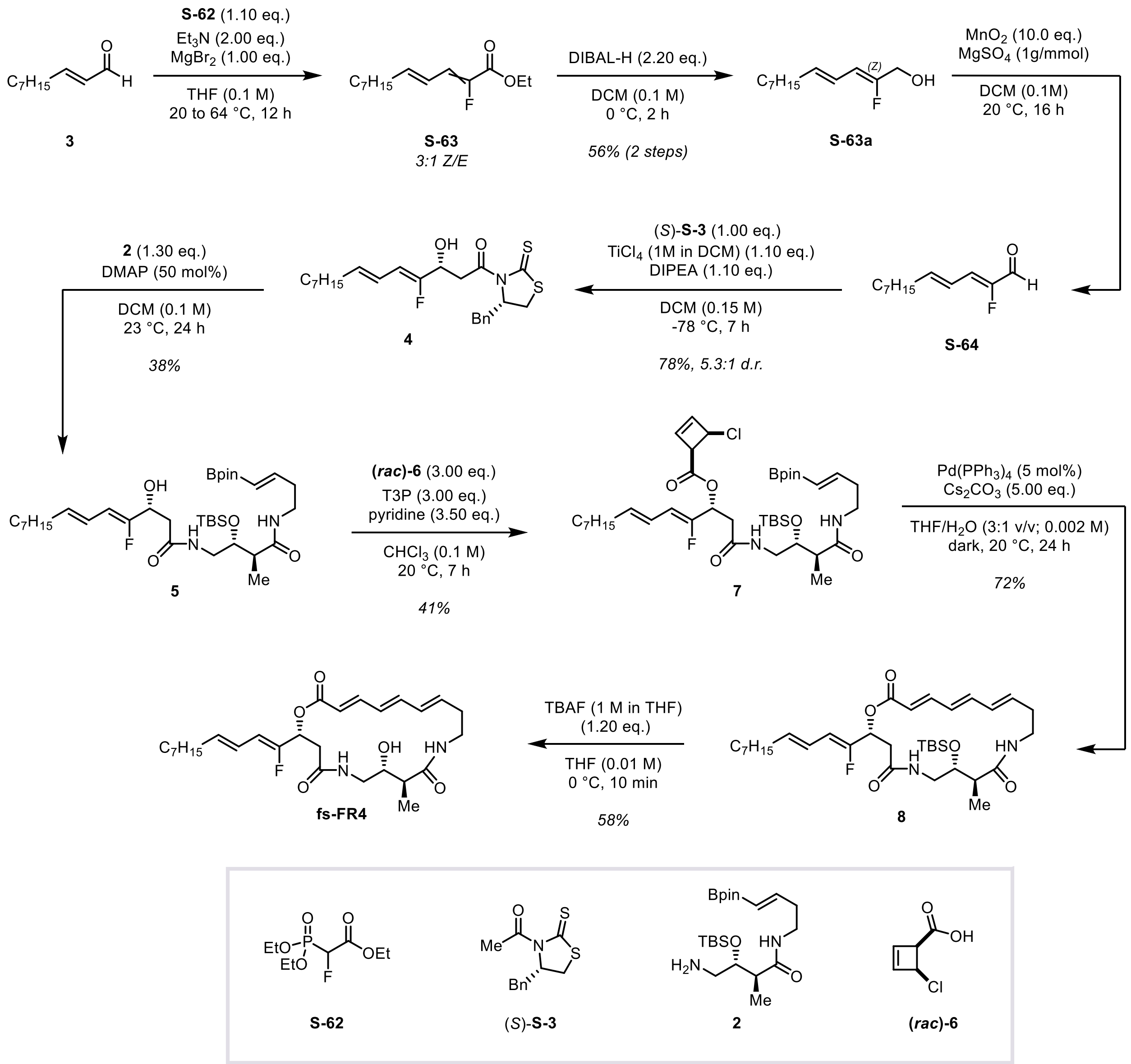


### 4.4.1. Ethyl (2*Z*,4*E*)-2-fluorododeca-2,4-dienoate ((*Z*)-S-63) & Ethyl (2*E*,4*E*)-2-fluorododeca-2,4-dienoate ((*E*)-S-63)

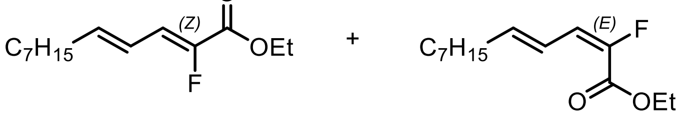

To a solution of triethyl 2-fluoro-2-phosphonoacetate **S-62** (11.8 g, 44.0 mmol, 1.10 equiv.) in THF (0.2 M) at 20 °C was added $Et_3N$ (11.2 mL, 80.0 mmol, 2.00 equiv.), followed by $MgBr_2$ (7.36 g, 40.0 mmol,

1.00 equiv.) and the resulting mixture was stirred for 10 min. Afterwards, *trans*-2-decenal **3** (7.34 mL, 40.0 mmol, 1.00 equiv.) was added and the reaction was heated at reflux for 12 h. Upon full consumption of the starting material (as indicated by $^{19}$F-NMR analysis), the reaction was allowed to cool to room temperature before being diluted with $Et_2O$ and the resulting suspension was filtered over a short pad of Celite®. The filtrate was washed with a sat. aq. solution of $NH_4Cl$ (100 mL) and the aqueous layer was extracted with $Et_2O$ (2 x 100 mL). The combined organic layers were washed with brine (1 x 100 mL), dried over $Na_2SO_4$, the solids were filtered off and the solvents were removed *in vacuo* to yield crude **S-63** with a *Z*/*E* ratio of 3:1, which was used in the next step without further purification.

**$^1$H-NMR** (400 MHz, $CHCl_3$): δ 6.98-6.89 (m, 1H, *E* isomer), 6.58 (dd, *J* = 31.6, 11.3 Hz, 1H, *Z* isomer), 6.45-6.31 (m, 1H each, *E* + *Z* isomer), 6.14-5.96 (m, 1H each, *E* + *Z* isomer), 4.30 (app dq, *J* = 14.3, 7.1 Hz, 2H, *E* + *Z* isomer), 2.18 (q, *J* = 7.1 Hz, 2H, *E* + *Z* isomer), 1.47-1.39 (m, 2H, *E* + *Z* isomer), 1.33-1.24 (m, 10H, *E* + *Z* isomer), 0.88 (t, *J* = 6.8 Hz, 3H, *E* + *Z* isomer) ppm.

**$^{19}$F-NMR** (376 MHz, $CHCl_3$): δ −126.9 (d, *J* = 20.1 Hz, *E* isomer), −131.2 (d, *J* = 31.7 Hz, *Z* isomer) ppm.

### 4.4.2. (2*Z*,4*E*)-2-Fluorododeca-2,4-dien-1-ol (S-63a)

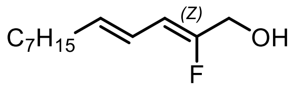


Synthesized according to the **General Procedure R** on 40.0 mmol scale to yield **S-63a** (4.44 g, 22.2 mmol, 56% over two steps) as a colorless oil.

**$^1$H-NMR** (600 MHz, $CDCl_3$): δ 6.26 (dd, *J* = 15.4, 10.8 Hz, 1H), 5.76 – 5.68 (m, 1H), 5.48 (dd, *J* = 35.3, 10.8 Hz, 1H), 4.15 (dd, *J* = 15.8, 6.4 Hz, 1H), 2.10 (q, *J* = 7.2 Hz, 2H), 1.63 (t, *J* = 6.4 Hz, 1H), 1.38 (m, 2H), 1.28 (dd, *J* = 7.5, 3.1 Hz, 8H), 0.88 (t, *J* = 6.9 Hz, 3H) ppm.

**$^{13}$C-NMR** (151 MHz, $CDCl_3$): δ 156.0 (d, *J* = 261 Hz), 136.0 (d, *J* = 3.2 Hz), 120.9 (d, *J* = 4.6 Hz), 108.9 (d, *J* = 10.7 Hz), 61.5 (d, *J* = 31.1 Hz), 33.0, 32.0, 29.3 (3C), 22.8, 14.2 ppm.

**$^{19}$F-NMR** (565 MHz, $CDCl_3$): δ −118.95 (dt, *J* = 32.4, 15.9 Hz) ppm.

**HRMS (ESI$^+$):** could not be detected by mass spectrometry.

**FT-IR (neat) $\nu_{max}$:** 3460, 2954, 2925, 2856, 1726, 1464, 1376, 1233, 1165, 1044, 974 cm$^{-1}$.

### 4.4.3. (2*Z*,4*E*)-2-Fluorododeca-2,4-dienal (S-64)

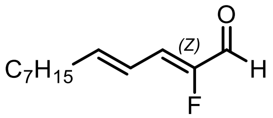

Synthesized according to the **General Procedure S** on 3.98 mmol scale to yield **S-64** (642 mg, 3.24 mmol, 81%) as a colorless oil and was quickly used in the next step without further purification.

**$^1$H-NMR** (400 MHz, $CDCl_3$): δ 9.21 (dd, *J* = 18.4 Hz, 1H), 6.51 (ddt, *J* = 13.6, 10.8, 1.2 Hz, 1H), 6.35 (dd, *J* = 30.1, 10.5 Hz, 1H), 6.32 – 6.24 (m, 1H), 2.24 (q, *J* = 7.2 Hz, 2H), 1.52 – 1.41 (m, 2H), 1.35-1.22 (m, 8H), 0.88 (t, *J* = 6.9 Hz, 3H) ppm.

**$^{19}$F-NMR** (376 MHz, $CDCl_3$): δ −134.01 (dd, *J* = 30.6, 18.3 Hz) ppm.

### 4.4.4. (*R*,4*Z*,6*E*)-1-((*S*)-4-Benzyl-2-thioxothiazolidin-3-yl)-4-fluoro-3-hydroxytetradeca-4,6-dien-1-one (4)

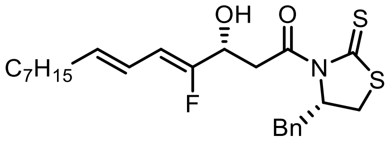

Synthesized according to the **General Procedure K** on 3.15 mmol scale to yield **4** (942 mg, 2.09 mmol, 66% single diastereomer, overall 5.3:1 d.r.; 78%) as a yellow oil.

**$^1$H-NMR** (600 MHz, $CDCl_3$): δ 7.36 – 7.34 (m, 2H), 7.29 – 7.26 (m, 3H), 6.26 (ddt, *J* = 15.2, 10.9, 1.3 Hz, 1H), 5.77 – 5.72 (m, 1H), 5.59 (dd, *J* = 36.2, 10.8 Hz, 1H), 5.39 (ddd, *J* = 10.7, 7.0, 4.1 Hz, 1H), 4.76 – 4.71 (m, 1H), 3.72 (dd, *J* = 17.7, 2.8 Hz, 1H), 3.51 (dd, *J* = 17.7, 9.0 Hz, 1H), 3.42 (ddd, *J* = 11.5, 7.2, 0.7 Hz, 1H), 3.23 (dd, *J* = 13.2, 3.9 Hz, 1H), 3.05 (dd, *J* = 13.2, 10.5 Hz, 1H), 2.93 (d, *J* = 1.6 Hz, 1H), 2.91 (d, *J* = 8.1 Hz, 1H), 2.10 (q, *J* = 7.1 Hz, 2H), 1.42 – 1.36 (m, 2H), 1.31 – 1.25 (m, 8H), 0.88 (t, *J* = 7.0 Hz, 3H) ppm.

**$^{13}$C-NMR** (151 MHz, $CDCl_3$): δ 201.4, 172.1, 156.7 (d, *J* = 261.7 Hz), 136.5, 136.2 (d, *J* = 3.0 Hz), 129.6 (2C), 129.1 (2C), 127.5, 120.7 (d, *J* = 5.0 Hz), 107.7 (d, *J* = 9.7 Hz), 68.5, 66.7 (d, *J* = 31.2 Hz), 43.1, 37.0, 33.0, 32.3, 32.0, 29.3 (3C), 22.8, 14.2 ppm.

**$^{19}$F-NMR** (576 MHz, $CDCl_3$): δ −121.89 (dd, *J* = 36.3, 11.7 Hz) ppm.

**HRMS (ESI$^+$):** calculated for $[M+Na]^+$ ($C_{24}H_{32}NO_2FS_2Na^+$) required *m/z* 472.1756, found *m/z* 472.1752.

**FT-IR (neat) $\nu_{max}$:** 2954, 2923, 2853, 1686, 1496, 1454, 1437, 1357, 1341, 1318, 1291, 1261, 1192, 1165, 1136, 1087, 1045, 969, 914, 890, 854, 744, 701, 683, 658, 641, 591, 554, 504, 480 cm$^{-1}$.

**$[\alpha]_D^{20}$** = +115 (c = 1.00, $CDCl_3$).

### 4.4.5. (*R*,4*Z*,6*E*)-*N*-((2*R*,3*S*)-2-((*tert*-Butyldimethylsilyl)oxy)-3-methyl-4-oxo-4-(((*E*)-4-(4,4,5,5-tetramethyl-1,3,2-dioxaborolan-2-yl)but-3-en-1-yl)amino)butyl)-4-fluoro-3-hydroxytetradeca-4,6-dienamide (5)

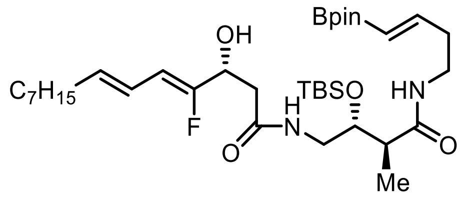


Synthesized according to the **General Procedure L** on 0.700 mmol scale to yield **5** (179 mg, 0.268 mmol, 38%) as a yellow foam.

**$^1$H-NMR** (600 MHz, $CDCl_3$): δ 6.55 (dt, *J* = 18.0, 6.5 Hz, 1H), 6.46 (m, 1H), 6.22 (dd, *J* = 15.4, 10.9 Hz, 1H), 6.14 (t, *J* = 5.5 Hz, 1H), 5.73 – 5.68 (m, 1H), 5.58 (dd, *J* = 36.6, 10.9 Hz, 1H), 5.52 (d, *J* = 18.0 Hz, 1H), 4.55 (t, *J* = 9.4 Hz, 1H), 4.33 (d, *J* = 3.7 Hz, 1H), 3.89 – 3.85 (m, 1H), 3.68 (ddd, *J* = 13.6, 7.4, 4.3 Hz, 1H), 3.45 (td, *J* = 13.3, 6.6 Hz, 1H), 3.27 (td, *J* = 12.3, 6.4 Hz, 1H), 2.96 (ddd, *J* = 12.6, 7.6, 4.6 Hz, 1H), 2.59 (dd, *J* = 15.2, 3.3 Hz, 1H), 2.49 (dd, *J* = 15.2, 8.5 Hz, 1H), 2.41 (ddd, *J* = 14.4, 7.2, 4.9 Hz, 1H), 2.36 (dd, *J* = 13.2, 6.6 Hz, 2H), 2.08 (dd, *J* = 14.2, 7.0 Hz, 2H), 1.38 – 1.34 (m, 2H), 1.29 – 1.25 (m, 20H), 1.14 – 1.12 (m, 3H), 0.92 – 0.86 (m, 12H), 0.14 – 0.09 (m, 6H) ppm.

**$^{13}$C-NMR** (151 MHz, $CDCl_3$): δ 174.6, 171.5, 157.1 (d, *J* = 261.5 Hz), 150.4, 135.8 (d, *J* = 2.8 Hz), 120.75 (d, *J* = 5.0 Hz), 120.72 (*deducted from HSQC*), 107.3 (d, *J* = 9.4 Hz), 83.4 (2C), 72.1, 67.3 (d, *J* = 31.8 Hz), 44.8, 42.9, 39.7, 37.9, 35.8, 33.0, 32.0 (2C), 29.32, 29.29, 26.00 (3C), 25.01, 24.92 (2C), 24.91, 22.8, 18.1, 15.3, 14.2, -4.4, -4.8 ppm.

**$^{19}$F-NMR** (576 MHz, $CDCl_3$): δ −122.73 (ddd, *J* = 73.8, 36.5, 11.0 Hz) ppm.

**HRMS (ESI$^+$):** calculated for $[M+Na]^+$ ($C_{35}H_{64}N_2O_6BFSiNa^+$) required *m/z* 689.4503, found *m/z* 689.4494.

**FT-IR (neat) $\nu_{max}$:** 3307, 2975, 2954, 2927, 2856, 1640, 1540, 1463, 1397, 1388, 1359, 1321, 1254, 1183, 1143, 1099, 1026, 1003, 970, 952, 847, 836, 812, 778, 750, 721, 680, 667, 648, 577 cm$^{-1}$.

**$[\alpha]_D^{20}$** = +215 (c = 1.00, $CHCl_3$).

### 4.4.6. (*R*,4*Z*,6*E*)-1-(((2*R*,3*S*)-2-((tert-Butyldimethylsilyl)oxy)-3-methyl-4-oxo-4-(((*E*)-4-(4,4,5,5-tetramethyl-1,3,2-dioxaborolan-2-yl)but-3-en-1-yl)amino)butyl)amino)-4-fluoro-1-oxotetradeca-4,6-dien-3-yl (1*S*,4*S*)-4-chlorocyclobut-2-ene-1-carboxylate (7)

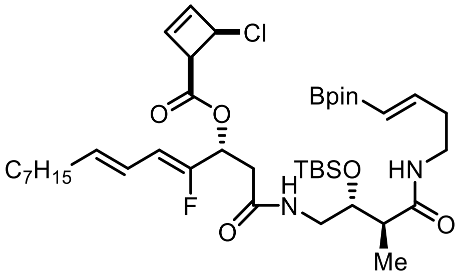


Synthesized according to the **General Procedure M** on 100 µmol scale to yield **7** (31.9 mg, 41.0 µmol, 41%, mixture of *cis*-diastereomers) as a colorless foam.

**$^1$H-NMR** (700 MHz, $CDCl_3$): δ 6.55 (dt, *J* = 18.0, 6.5 Hz, 1H), 6.29 – 6.22 (m, 2H), 6.22 – 6.15 (m, 2H), 6.11 (s, 1H), 5.80 – 5.72 (m, 2H), 5.65 (dd, *J* = 35.2, 10.8 Hz, 1H), 5.51 (d, *J* = 18.0 Hz, 1H), 5.09 (d, *J* = 4.3 Hz, 1H), 4.13 – 4.03 (m, 1H), 3.85 – 3.80 (m, 1H), 3.76 (ddd, *J* = 13.6, 8.1, 4.1 Hz, 1H), 3.43 (dt, *J* = 13.2, 6.6 Hz, 1H), 3.30 – 3.23 (m, 1H), 2.84 (ddd, *J* = 13.6, 7.6, 4.1 Hz, 1H), 2.74 (dd, *J* = 14.6, 7.6 Hz, 1H), 2.66 (dd, *J* = 14.6, 6.2 Hz, 1H), 2.40 – 2.32 (m, 3H), 2.07 (dd, *J* = 14.2, 7.1 Hz, 2H), 1.41 – 1.33 (m, 2H), 1.32 – 1.21 (m, 21H), 1.10 (d, *J* = 7.2 Hz, 3H), 0.90 (s, 9H), 0.87 (t, *J* = 7.1 Hz, 4H), 0.15 (s, 3H), 0.08 (s, 3H) ppm.

**$^{13}$C-NMR** (176 MHz, $CDCl_3$): δ 174.5, 168.6, 168.4, 153.3, 151.8, 150.3, 140.7, 137.7, 137.7, 136.4, 120.48 (*deducted from HSQC*), 120.5, 112.1, 112.0, 83.4, 72.1, 70.3, 70.2, 56.4, 53.8, 44.7, 43.0, 38.6, 38.0, 35.8, 33.1, 31.9, 29.9, 29.3, 29.3, 29.2, 26.0, 24.9, 24.9, 22.8, 18.1, 15.4, 14.2, −4.4, −4.9 ppm.

**$^{19}$F-NMR** (659 MHz, $CDCl_3$): δ −126.2 (dd, *J* = 35.2, 22.2 Hz) ppm.

**HRMS (ESI$^+$):** calculated for $[M+Na]^+$ ($C_{40}H_{67}N_2O_7BClFSiNa^+$) required *m/z* 803.4375, found *m/z* 803.4371.

**FT-IR (neat)** $\nu_{max}$: 3310, 2954, 2927, 2855, 1742, 1643, 1538, 1463, 1389, 1359, 1323, 1289, 1256, 1235, 1165, 1143, 1117, 1025, 994, 970, 899, 838, 809, 776, 736, 680 cm$^{-1}$.

### 4.4.7. (2*R*,7*R*,8*S*,13*E*,15*E*,17*E*)-7-((*tert*-Butyldimethylsilyl)oxy)-2-((1*Z*,3*E*)-1-fluoroundeca-1,3-dien-1-yl)-8-methyl-1-oxa-5,10-diazacyclononadeca-13,15,17-triene-4,9,19-trione (8)

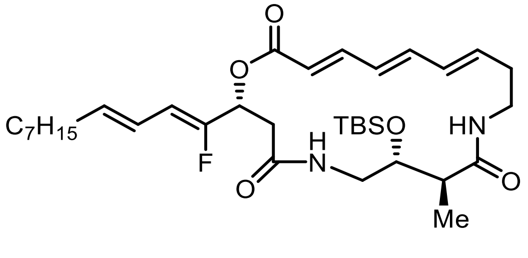

Synthesized according to the **General Procedure N** on 32.0 µmol scale to yield **8** (9.30 mg, 15.0 µmol, 72%) as a colorless solid.

**$^1$H-NMR** (600 MHz, $CDCl_3$): δ 7.31 – 7.26 (m, 1H), 6.48 (dd, *J* = 14.8, 10.9 Hz, 1H), 6.25 – 6.07 (m, 4H), 6.00 (s, 1H), 5.92 (ddd, *J* = 14.7, 11.0, 3.4 Hz, 1H), 5.79 – 5.60 (m, 3H), 4.13 – 4.01 (m, 2H), 3.48 (dt, *J* = 13.8, 8.1 Hz, 1H), 3.02 – 2.91 (m, 1H), 2.68 (dd, *J* = 13.7, 11.7 Hz, 1H), 2.54 (dd, *J* = 13.8, 3.3 Hz, 2H), 2.49 (qd, *J* = 7.2, 3.1 Hz, 1H), 2.41 (dt, *J* = 13.7, 4.4 Hz, 1H), 2.19 (ddd, *J* = 15.2, 9.7, 4.7 Hz, 1H), 2.09 (q, *J* = 7.1 Hz, 2H), 1.72 (s, 3H), 1.43 – 1.34 (m, 2H), 1.28 (m, 10H), 1.13 (d, *J* = 7.3 Hz, 3H), 0.91 – 0.81 (m, 14H), 0.08 (s, 3H), 0.05 (s, 3H) ppm.

**$^{13}$C-NMR** (151 MHz, $CDCl_3$): δ 174.1, 168.9, 166.0, 154.6, 152.9, 147.5, 143.8, 139.2, 137.3, 137.3, 131.5, 127.5, 120.6, 120.6, 118.8, 110.4, 110.3, 72.3, 71.0, 70.8, 45.3, 43.0, 40.1, 37.5, 34.5, 33.1, 32.0, 29.3, 29.3, 29.2, 25.8, 22.8, 18.0, 14.2, −4.5, −4.8 ppm.

**$^{19}$F-NMR** (565 MHz, $CDCl_3$): −124.5 (dd, *J* = 35.3, 19.5 Hz) ppm.

**HRMS (ESI$^+$):** calculated for $[M+Na]^+$ ($C_{34}H_{55}N_2O_5FSiNa^+$) required *m/z* 641.3762, found *m/z* 641.3758.

**FT-IR (neat) $\nu_{max}$:** 3323, 2928, 2856, 2341, 2206, 2056, 1965, 1713, 1614, 1538, 1434, 1249, 1102, 1012, 970, 833, 775 cm$^{-1}$.

**$[\alpha]_D^{20}$** = +9.8 (c = 0.11, $CHCl_3$).

### 4.4.8. Fs-FR4

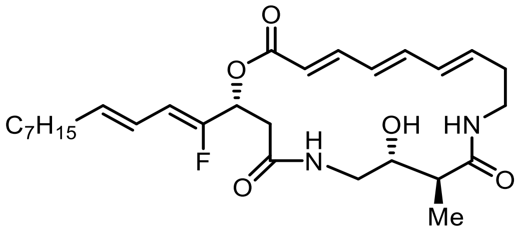


Synthesized according to the **General Procedure O** on 15.0 µmol scale to yield **Fs-FR4** (4.40 mg, 8.70 µmol, 58%) as a colorless solid.

**$^1$H-NMR** (600 MHz, $CDCl_3$): δ 7.31 – 7.27 (m, 1H), 6.51 (dd, *J* = 14.8, 10.8 Hz, 1H), 6.32 (d, *J* = 8.8 Hz, 1H), 6.20 (ddd, *J* = 26.4, 15.1, 11.2 Hz, 2H), 6.07 (dd, *J* = 14.9, 11.0 Hz, 1H), 5.97 (s, 1H), 5.83 – 5.73 (m, 4H), 5.72 – 5.56 (m, 3H), 4.16 – 4.10 (m, 1H), 3.82 (t, *J* = 10.2 Hz, 1H), 3.33 – 3.26 (m, 1H), 3.01 (d, *J* = 13.3 Hz, 1H), 2.77 (dd, *J* = 13.9, 11.9 Hz, 1H), 2.64 – 2.56 (m, 3H), 2.30 – 2.16 (m, 4H), 2.09 (dd, *J* = 14.2, 7.1 Hz, 2H), 1.56 (s, 6H), 1.38 (m, 2H), 1.31 – 1.21 (m, 14H), 0.97 (d, *J* = 7.1 Hz, 3H), 0.90 – 0.78 (m, 11H) ppm.

**$^{13}$C-NMR** (151 MHz, $CDCl_3$): δ 176.0, 168.4, 165.2, 154.4, 152.7, 145.4, 141.3, 137.4, 137.4, 136.5, 132.9, 128.6, 120.6, 120.6, 120.5, 110.7, 110.6, 71.6, 70.0, 44.1, 43.0, 39.7, 36.9, 34.7, 33.1, 32.0, 29.3, 29.2, 22.8, 19.8, 14.2, 11.0 ppm.

**$^{19}$F-NMR** (565 MHz, $CDCl_3$): δ −124.9 (dd, *J* = 35.3, 20.2 Hz) ppm.

**HRMS (ESI$^+$):** calculated for $[M+Na]^+$ ($C_{28}H_{41}N_2O_5FNa^+$) required *m/z* 527.2897, found *m/z* 527.2886.

**FT-IR (neat) $\nu_{max}$:**3304, 2925, 2854, 1706, 1639, 1615, 1541. 1250, 1066, 1007, 970 $cm^{-1}$.

**$[\alpha]_D^{20}$** = −231 (c = 0.50, $CHCl_3$).

## 4.5. Fs-FR5 Synthesis

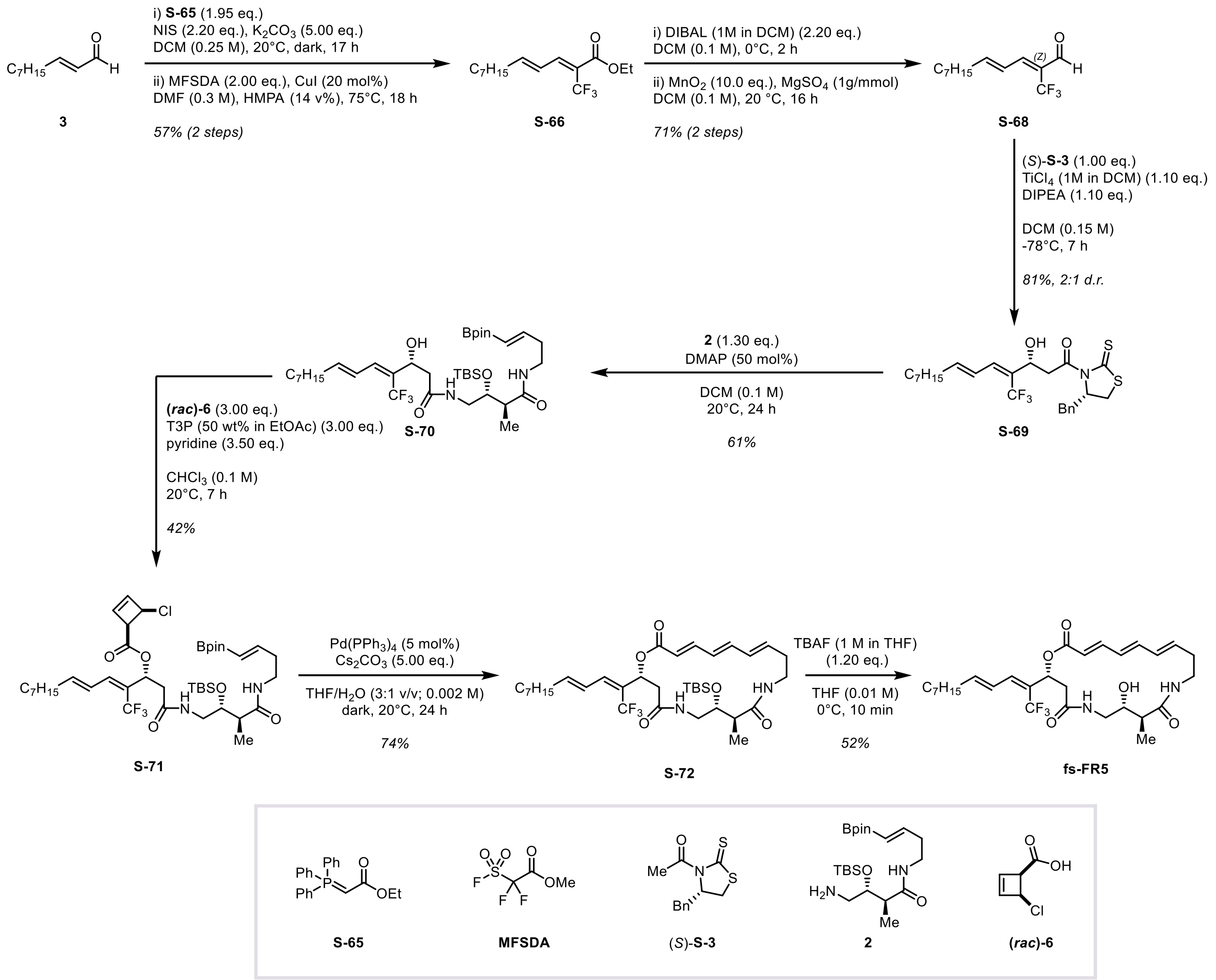

### 4.5.1. Ethyl (2Z,4E)-2-(trifluoromethyl)dodeca-2,4-dienoate (S-66)

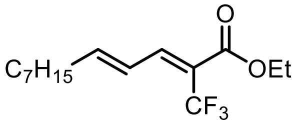

All reaction steps and work-up procedures were carried out in the dark. To a solution of ethyl (triphenylphosporanylidene)acetate (**S-65**) (16.3 g, 46.8 mmol, 1.95 equiv.) in $CH_2Cl_2$ (0.25 M) at −20 °C was added NIS (11.9 g, 52.8 mmol, 2.20 equiv.) and the resulting reaction mixture was stirred at −20 °C for 20 min. Afterwards, $K_2CO_3$ (16.6 g, 120 mmol, 5.00 equiv.) was added followed by *trans*-2-decenal (**3**) (4.40 mL, 24.0 mmol, 1.00 equiv.). The resulting reaction mixture was allowed to warm to 20 °C and stirring was continued for 17 h. After the reaction time had elapsed, the reaction mixture was filtered through a short pad of Celite® and the filter cake was washed with copious amounts of $CH_2Cl_2$. The filtrate was

concentrated, and the crude material was purified by flash column chromatography ($SiO_2$, heptane/EtOAc) to afford a mixture of *trans*-2-decenal and C11-I-**S-66** which was used in the next step without further purification.

C11-I-**S-66** (5.25 g, 15.0 mmol, 1.00 equiv.) was dissolved in DMF (0.3 M) at room temperature and HMPA (11.3 mL, 65.2 mmol, 4.35 equiv., 14 v% relative to DMF) and CuI (574 mg, 3.00 mmol, 0.200 equiv.) were added. The resulting reaction mixture was heated to 75 °C before MFSDA (3.94 mL, 30.0 mmol, 2.00 equiv.) was added. Stirring was continued at 75 °C for 14 h, after which the reaction mixture was allowed to cool to 20 °C. The reaction mixture was diluted with $Et_2O$ (50.0 mL) and the layers were separated. The organic layer was washed with water (5 x 50.0 mL), EDTA solution (1 x 50.0 mL) and brine (1 x 50.0 mL), dried over $Na_2SO_4$, the solids were filtered off and the solvents were removed *in vacuo* to yield the crude product as a mixture with *trans*-2-decenal. The crude product was purified by flash column chromatography ($SiO_2$, heptanes/$CH_2Cl_2$) to yield **S-66** (2.49 g, 8.52 mmol, 36%; 57% brsm) as a colourless oil.
**$^{1}$H-NMR** (700 MHz, $CDCl_3$): δ 7.50 (d, *J* = 11.9 Hz, 1H), 6.68 (dddd, *J* = 15.1, 13.4, 3.2, 1.4 Hz, 1H), 6.44 – 6.39 (m, 1H), 4.28 (q, *J* = 7.1 Hz, 2H), 2.26 (q, *J* = 7.3 Hz, 2H), 1.49 – 1.43 (m, 2H), 1.34 – 1.24 (m, 11H), 0.88 (t, *J* = 7.1 Hz, 3H) ppm.
**$^{13}$C-NMR** (176 MHz, $CDCl_3$): δ 163.75 (d, *J* = 4.0, 2.0 Hz), 153.61 (app. d, *J* = 1.4 Hz), 147.78 (dd, *J* = 3.9, 1.9 Hz), 124.61 (q, *J* = 3.8 Hz), 123.05 (q, *J* = 273.6 Hz), 117.53 (q, *J* = 31.4 Hz), 61.62, 33.64, 31.88, 29.29, 29.19, 28.55, 22.76, 14.26, 14.20 ppm.
**$^{19}$F-NMR** (658 MHz, $CDCl_3$): δ −58.09 (s) ppm.
**HRMS (ESI$^+$):** calculated for $[M+Na]^+$ ($C_{15}H_{23}F_3O_2Na^+$) required *m/z* 315.1548, found *m/z* 315.1541.
**FT-IR (neat) $\nu_{max}$:** 2957, 2857, 1725, 1634, 1252, 1133, 1043, 984 $cm^{-1}$

### 4.5.2. (2Z,4E)-2-(Trifluoromethyl)dodeca-2,4-dien-1-ol (S-67)

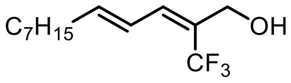


Synthesized according to **General Procedure R** on 5.03 mmol scale and was used quickly in the next step without further purification.

### 4.5.3. (2*Z*,4*E*)-2-(Trifluoromethyl)dodeca-2,4-dienal (S-68)

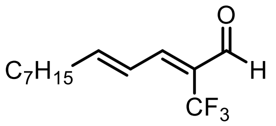

Synthesized according to **General Procedure O**utlined S on 5.03 mmol scale to yield **S-68** (881 mg, 3.55 mmol, 71%) as a colourless oil which was unstable and used without further purification.

**$^1$H-NMR** (400 MHz, $CDCl_3$): δ 9.52 (q, *J* = 1.7 Hz, 1H), 7.26 (d, *J* = 11.7 Hz, 1H), 6.74 (dddd, *J* = 13.1, 11.6, 2.7, 1.4 Hz, 1H), 6.63 – 6.54 (m, 1H), 2.32 (q, *J* = 7.2 Hz, 2H), 1.55 – 1.40 (m, 2H), 1.37 – 1.22 (m, 8H), 0.92 – 0.81 (m, 3H) ppm.

### 4.5.4. (*R*,4*Z*,6*E*)-1-((*S*)-4-Benzyl-2-thioxothiazolidin-3-yl)-3-hydroxy-4-(trifluoromethyl)tetradeca-4,6-dien-1-one (S-69)

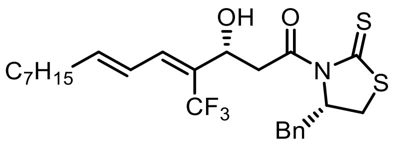

Synthesized according to **General Procedure K** on 0.920 mmol scale to yield **S-69** (247 mg, 0.494 mmol, 54%) as a yellow oil (overall: d.r. 2:1, 81%).

**$^1$H-NMR** (600 MHz, $CDCl_3$): δ 7.39 – 7.33 (m, 2H), 7.31 – 7.27 (m, 3H) 6.64 (d, *J* = 11.6 Hz, 1H), 6.48 – 6.45 (m, 1H), 6.08 – 6.01 (m, 1H), 5.42 – 5.35 (m, 1H), 4.93 (d, *J* = 9.3 Hz, 1H), 3,72 (dd, *J* = 17.9, 2.3 Hz, 1H), 3.48 – 3.34 (m, 2H), 3.24 (dd, *J* = 13.2, 3.9 Hz,1H), 3.09 – 3.01 (m, 2H), 2.92 (d, *J* = 11.5 Hz, 1H), 2.22 – 2.14 (m, 2H), 1.45 – 1.39 (m, 2H), 1.34 – 1.22 (m, 8H), 0.89 (t, *J* = 7.0 Hz, 3H) ppm.

**$^{13}$C-NMR** (151 MHz, $CDCl_3$): δ 201.4, 172.3, 145.1, 136.5, 136.1 (q, *J* = 3.3 Hz), 129.6 (2C), 129.1 (2C), 127.5, 126.5 (q, *J* = 28.1 Hz), 124.5 (d, *J* = 1.5 Hz), 124.0 (q, *J* = 275 Hz), 68.4, 66.1 (dd, *J* = 2.9 Hz), 45.8, 37.0, 33.1, 32.3, 31.9, 29.3, 29.2, 28.9, 22.8, 14.2 ppm.

**$^{19}$F-NMR** (565 MHz, $CDCl_3$): δ −58.16 (s) ppm.

**HRMS (ESI$^+$):** calculated for $[M+Na]^+$ ($C_{25}H_{32}F_3NO_2S_2Na^+$) required *m/z* 522.1724, found *m/z* 522.1721.

**FT-IR (neat) $\nu_{max}$:** 3065, 3027, 2954, 2925, 2854, 1696, 1636, 1601, 1493, 1455, 1440, 1404, 1365, 1341, 1319, 1295, 1273, 1210, 1178, 1141, 1086, 1060, 1018, 1001, 952, 914, 891, 863, 847, 747, 701, 672, 608, 573, 513, 486 $cm^{-1}$.

**$[\alpha]_D^{20}$** = +165 (c = 1.70, $CHCl_3$).

### 4.5.5. (*R*,4*Z*,6*E*)-*N*-((2*R*,3*S*)-2-((*tert*-Butyldimethylsilyl)oxy)-3-methyl-4-oxo-4-(((*E*)-4-(4,4,5,5-tetramethyl-1,3,2-dioxaborolan-2-yl)but-3-en-1-yl)amino)butyl)-3-hydroxy-4-(trifluoromethyl)tetradeca-4,6-dienamide (S-70)

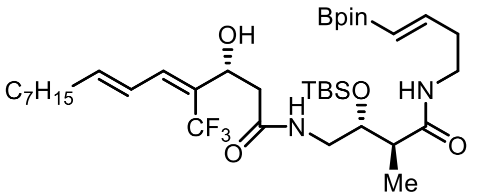


Synthesized according to **General Procedure L** on 0.494 mmol scale to yield **S-70** (217 mg, 0.303 mmol, 61%) as a yellow foam.

**$^1$H-NMR** (600 MHz, $CDCl_3$): δ 6.71 (dd, *J* = 7.1, 4.8 Hz, 1H), 6.63 (d, *J* = 11.5 Hz, 1H), 6.51 (dt, *J* = 18.0, 6.5 Hz, 1H), 6.44 – 6.37 (m, 1H), 6.28 (t, *J* = 5.7 Hz, 1H), 6.01 – 5.94 (m, 1H), 5.48 (d, *J* = 18.0 Hz, 1H), 4.87 (d, *J* = 2.6 Hz, 1H), 4.70 (d, *J* = 8.7 Hz, 1H), 3.83 (dt, *J* = 8.2, 4.3 Hz, 1H), 3.65 (ddd, *J* = 13.5, 7.4, 4.3 Hz, 1H), 3.38 (td, *J* = 13.2, 6.7 Hz, 1H), 3.24 (dt, *J* = 12.6, 6.5 Hz, 1H), 2.91 – 2.86 (m, 1H), 2.53 (dd, *J* = 15.2, 2.7 Hz, 1H), 2.43 – 2.37 (m, 1H), 2.32 (dt, *J* = 12.4, 5.3 Hz, 2H), 2.12 (q, *J* = 7.1 Hz, 2H), 1.41 – 1.34 (m, 2H), 1.29 – 1.20 (m, 24H), 1.11 (d, *J* = 7.2 Hz, 3H), 0.87 (s, 9H), 0.13 (s, 3H), 0.07 (s, 3H) ppm.

**$^{13}$C-NMR** (151 MHz, $CDCl_3$): δ 174.7, 171.7, 150.2, 144.4, 135.5 (d, *J* = 3.3 Hz), 127.1 (q, *J* = 27.6 Hz), 124.5, 124.2 (q, *J* = 275.3 Hz), 121.6 (*deduced from HSQC*), 83.3 (2C), 72.1, 66.8 (q, *J* = 3.0 Hz), 44.6, 42.9, 42.5, 38.0, 35.7, 33.1, 31.9, 29.3, 29.2, 29.0, 26.0, 25.0 (3C), 24.92 (2C), 24.91 (2C), 22.8, 15.2, 14.2, −4.38, −4.81 ppm.

**$^{19}$F-NMR** (565 MHz, $CDCl_3$): δ −57.97 (s) ppm.

**HRMS (ESI$^+$):** calculated for $[M+Na]^+$ ($C_{36}H_{64}BF_3N_2O_6SiNa^+$) required *m/z* 739.4477, found *m/z* 739.4455.

**FT-IR (neat) $\nu_{max}$:** 3351, 2927, 2856, 1710, 1650, 1538, 1362, 1252, 1223, 1100, 1007, 836, 778, 530 $cm^{-1}$.

**$[\alpha]_D^{20}$** = +8.7 (c = 8.50, $CHCl_3$).

### 4.5.6. (*R*,4*Z*,6*E*)-1-(((2*R*,3*S*)-2-((*tert*-Butyldimethylsilyl)oxy)-3-methyl-4-oxo-4-(((*E*)-4-(4,4,5,5-tetramethyl-1,3,2-dioxaborolan-2-yl)but-3-en-1-yl)amino)butyl)amino)-1-oxo-4-(trifluoromethyl)tetradeca-4,6-dien-3-yl (1*R*,4*R*)-4-chlorocyclobut-2-ene-1-carboxylate (S-71)

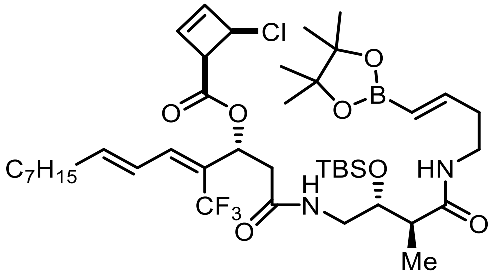


Synthesized according to **General Procedure M** on 0.140 mmol scale to yield **S-71** (49.0 mg, 0.059 mmol, 42%) as a yellow foam.

**$^{1}$H-NMR** (600 MHz, $CDCl_3$): δ 6.65 (d, *J* = 11.5 Hz, 1H), 6.55 (dt, *J* = 18.0, 6.5 Hz, 1H), 6.39 (t, *J* = 13.3 Hz, 1H), 6.25 (app. s, 2H), 6.05 (m, 1H), 5.90 (dd, *J* = 8.3, 5.4 Hz, 1H), 5.51 (d, *J* = 18.0 Hz, 1H), 5.08 (d, *J* = 4.3 Hz, 1H), 4.07 (d, *J* = 4.2 Hz, 1H), 3.84 (m, 1H), 3.80 – 3.72 (m, 1H), 3.43 (dt, *J* = 13.2, 6.6 Hz, 1H), 3.26 (dt, *J* = 13.3, 6.6 Hz, 1H), 2.85 (ddd, *J* = 13.7, 7.2, 4.0 Hz, 1H), 2.73 (dd, *J* = 14.9, 8.4 Hz, 1H), 2.62 (m, 1H), 2.41 – 2.30 (m, 3H), 2.15 (m, 2H), 1.69 (s, 1H), 1.45 – 1.35 (m, 2H), 1.35 – 1.17 (m, 20H), 1.14 (m, 1H), 1.09 (d, *J* = 7.1 Hz, 3H), 0.93 – 0.84 (m, 12H), 0.13 (s, 3H), 0.07 (s, 3H) ppm.

**$^{13}$C-NMR** (151 MHz, $CDCl_3$): δ 174.4, 168.6, 168.5, 150.3, 146.6, 140.7, 140.3, 136.4, 124.2, 122.8 (m, 1C), 120.9 (*deducted from HSQC*), 83.4 (2C), 72.2, 71.7, 56.2, 53.8, 44.8, 42.9, 41.3, 38.0, 35.8, 33.2, 31.9, 29.3, 29.2, 28.8, 26.0 (3C), 24.9 (4C), 22.8, 18.1, 15.2, 14.2, -4.4, -4.9 ppm.

**$^{19}$F-NMR** (565 MHz, $CDCl_3$): δ −56.7 (s) ppm.

**HRMS (ESI$^+$):** calculated for $[M+Na]^+$ ($C_{41}H_{67}BClF_3N_2O_7SiNa^+$) required *m/z* 853.4343, found *m/z* 853.4315.

**FT-IR (neat) $\nu_{max}$:** 3313, 2955, 2929, 2857, 1743, 1649, 1537, 1359, 1119, 746 cm$^{-1}$.

### 4.5.7. (2*R*,7*R*,8*S*,13*E*,15*E*,17*E*)-7-((*tert*-Butyldimethylsilyl)oxy)-8-methyl-2-((2*Z*,4*E*)-1,1,1-trifluorododeca-2,4-dien-2-yl)-1-oxa-5,10-diazacyclononadeca-13,15,17-triene-4,9,19-trione (S-72)

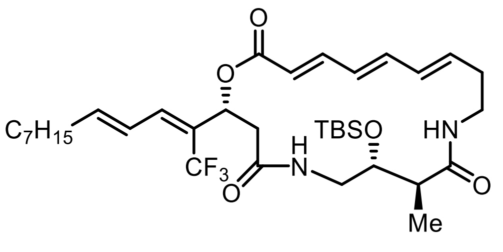

Synthesized according to the **General Procedure N** on 24.1 µmol scale to yield **S-72** (12.0 mg, 17.9 µmol, 74%) as a colorless solid.

**$^1$H-NMR** (700 MHz, $CDCl_3$): δ 7.24 (m, 1H), 6.62 (d, *J* = 11.5 Hz, 1H), 6.46 (m, 2H), 6.17 (dd, *J* = 14.8, 11.3 Hz, 1H), 6.08 (m, 2H), 5.92 (m, 2H), 5.83 (dd, *J* = 11.2, 3.3 Hz, 1H), 5.75 (s, 1H), 5.70 (d, *J* = 15.4 Hz, 1H), 4.10 – 4.01 (m, 2H), 3.48 (dt, *J* = 13.8, 8.2 Hz, 1H), 3.00 – 2.93 (m, 1H), 2.60 (dd, *J* = 13.8, 3.5 Hz, 1H), 2.53 (m, 2H), 2.51 – 2.46 (m, 1H), 2.46 – 2.40 (m, 1H), 2.20 – 2.11 (m, 3H), 1.59 (s, 3H), 1.44 – 1.38 (m, 2H), 1.28 (m, 15H), 1.12 (d, *J* = 7.3 Hz, 4H), 0.91 – 0.80 (m, 18H), 0.07 (s, 3H), 0.05 (s, 3H) ppm.

**$^{13}$C-NMR** (176 MHz, $CDCl_3$): δ 173.9, 168.9, 165.8, 147.2, 146.0, 143.6, 139.3, 138.2, 131.4, 127.5, 124.3, 123.9 (m), 118.9, 72.5, 72.2, 45.4, 43.2, 42.9, 37.5, 34.6, 33.2, 31.9, 29.9, 29.3, 29.2, 28.9, 25.8 (3C), 22.8 (4C), 14.2, -4.5, -4.8 ppm.

**$^{19}$F-NMR** (659 MHz, $CDCl_3$): δ −57.0 (s) ppm.

**HRMS (ESI$^+$):** calculated for $[M+Na]^+$ ($C_{35}H_{55}F_3N_2O_5SiNa^+$) required *m/z* 691.3725, found *m/z* 691.3723.

**FT-IR (neat) $\nu_{max}$:** 3316, 2954, 2926, 2855, 1710, 1648, 1613, 1536, 1117, 8357, 765 cm$^{-1}$.

**$[\alpha]_D^{20}$** = −20.8 (c = 1.00, $CHCl_3$).

### 4.5.8. Fs-FR5

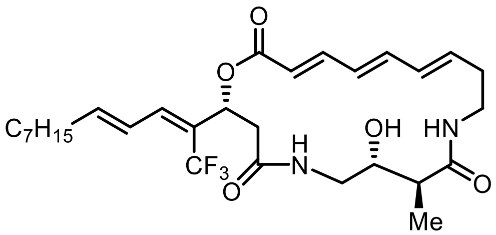


Synthesized according to **General Procedure O** on 15.7 µmol scale to yield **Fs-FR5** (4.5 mg, 8.10 µmol, 52%) as a colorless solid.

**$^1$H-NMR** (600 MHz, $CDCl_3$): δ 7.31 – 7.27 (m, 1H), 6.62 (d, *J* = 11.5 Hz, 1H), 6.53 (dd, *J* = 14.8, 10.8 Hz, 1H), 6.44 (m, 1H), 6.27 (d, *J* = 8.8 Hz, 1H), 6.19 (dd, *J* = 14.8, 11.3 Hz, 1H), 6.06 (m, 2H), 5.96 (s, 1H), 5.88 (dd, *J* = 11.1, 3.1 Hz, 1H), 5.81 (m, 1H), 5.76 (d, *J* = 15.4 Hz, 1H), 5.69 (d, *J* = 8.4 Hz, 1H), 4.14 (m, 1H), 3.84 (m, 1H), 3.31 (m, 1H), 3.01 (m, 1H), 2.62 (m, 4H), 2.30 – 2.19 (m, 2H), 2.17 (dd, *J* = 14.3, 7.2 Hz, 2H), 1.54 (s, 8H), 1.44 – 1.39 (m, 2H), 1.33 – 1.22 (m, 15H), 0.98 (d, *J* = 7.1 Hz, 3H), 0.88 (t, *J* = 6.9 Hz, 4H) ppm.

**$^{13}$C-NMR** (151 MHz, $CDCl_3$): δ 176.0, 168.5, 165.0, 146.0, 145.3, 141.3, 138.2, 136.5, 132.9, 128.6, 124.4, 124.0, 123.8, 120.6, 71.7, 71.3, 44.0, 43.0, 42.8, 36.9, 34.6, 33.2, 31.9, 29.9, 29.3, 29.2, 28.9, 22.8, 14.2, 11.1 ppm.

**$^{19}$F-NMR** (565 MHz, $CDCl_3$): δ −57.2 ppm.

**HRMS (ESI$^+$):** calculated for $[M+Na]^+$ ($C_{29}H_{41}F_3N_2O_5Na^+$) required *m/z* 577.2860, found *m/z* 577.2854.

**FT-IR (neat) $\nu_{max}$:** 3304, 2956, 2924, 2854, 1711, 1640, 1540, 1228, 1006, 730 $cm^{-1}$.

**$[\alpha]_D^{20}$** = −256.5 (c = 1.00, $CHCl_3$).

## 4.6. Fs-FR6 Synthesis

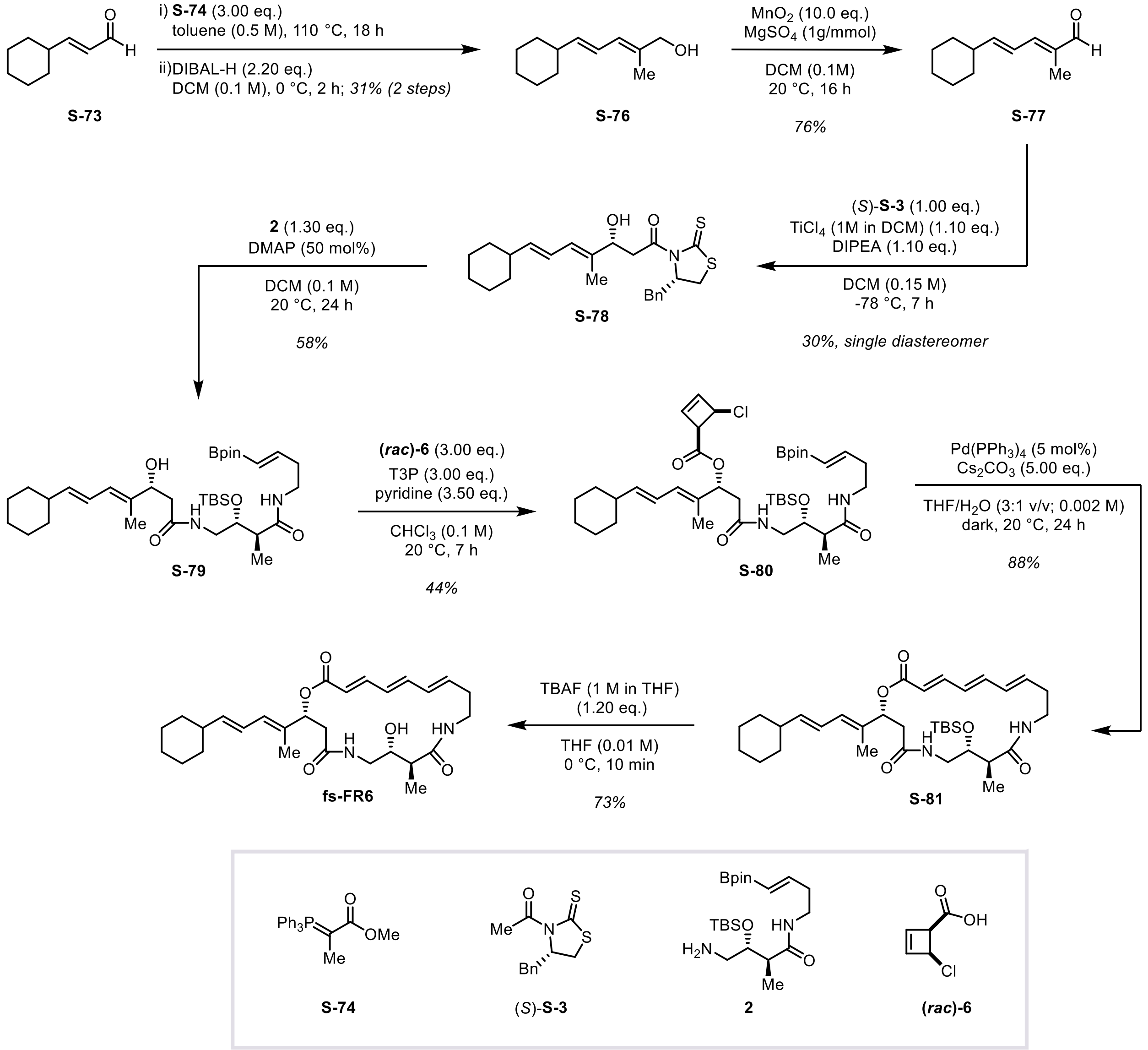


### 4.6.1. Methyl (2*E*,4*E*)-5-cyclohexyl-2-methylpenta-2,4-dienoate (S-75)

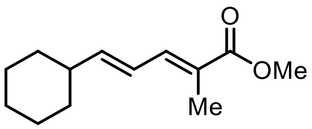

(*E*)-3-Cyclohexylacrylaldehyde **S-73** (3.41 g, 24.7 mmol, 1.00 eq) and methyl 2-(triphenyl-l$^{5}$-phosphaneylidene)propanoate **S-74** (25.8 g, 74.1 mmol, 3.00 eq) were dissolved in toluene (0.5 M) and the resulting mixture was heated at reflux for 18 h. Afterwards, the reaction mixture was concentrated and

filtered through a short pad of silica, which was washed with 20% EtOAc in heptane. The filtrate was concentrated and the resulting crude **S-75** was used without further purification in the next step.

### 4.6.2. (2*E*,4*E*)-5-Cyclohexyl-2-methylpenta-2,4-dien-1-ol (S-76)

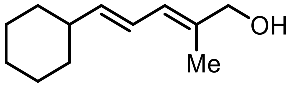


Synthesized according to **General Procedure R** on 14.8 mmol scale to yield **S-76** (821 mg, 4.55 mmol, 31% over two steps) as a colourless oil.

**$^{1}$H-NMR** (600 MHz, $CDCl_3$): δ 6.21 (ddd, *J* = 15.2, 10.8, 1.0 Hz, 1H), 5.99 (d, *J* = 10.8 Hz, 1H), 5.64 (dd, *J* = 15.2, 7.1 Hz, 1H), 4.03 (s, 2H), 2.03-1.99 (m, 1H), 1.76 (s, 3H), 1.73 – 1.70 (m, 4H), 1.66 – 1.62 (m, 1H), 1.30 – 1.26 (m, 1H), 1.19 – 1.06 (m, 4H) ppm.

**$^{13}$C-NMR** (151 MHz, $CDCl_3$): δ 141.1, 135.0, 125.7, 123.4, 68.8, 41.2, 33.1 (2C), 26.3, 26.1, 21.1, 14.3 ppm.

**HRMS (ESI$^{-}$):** calculated for $[M]^{-}$ ($C_{12}H_{19}O^{-}$) required *m/z* 179.1441, found *m/z* 179.1429.

**FT-IR (neat) $\nu_{max}$:** 3307, 3023, 2921, 2850, 1447, 1386, 1349, 1302, 1259, 1222, 1066, 1005, 966, 892, 749, 667, 624 $cm^{-1}$.

### 4.6.3. (2*E*,4*E*)-5-Cyclohexyl-2-methylpenta-2,4-dienal (S-77)

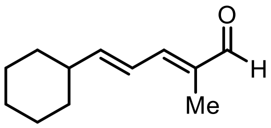


Synthesized according to **General Procedure S** on 3.00 mmol scale to yield **S-77** (408 mg, 2.29 mmol, 76%) as a colourless oil.

**$^{1}$H-NMR** (600 MHz, $CDCl_3$): δ 9.41 (s, 1H), 6.81 (d, *J* = 11.1 Hz, 1H), 6.47 (ddd, *J* = 15.2, 11.1, 1.2 Hz, 1H), 6.18 (dd, *J* = 15.2, 7.1 Hz, 1H), 2.16 (dtd, *J* = 14.2, 7.3, 3.6 Hz, 1H), 1.82 (d, *J* = 0.8 Hz, 3H), 1.80 – 1.74 (m, 4H), 1.69 – 1.67 (m, 1H), 1.32 – 1.27 (m, 2H), 1.22 – 1-13 (m, 3H) ppm.

**$^{13}$C-NMR** (151 MHz, $CDCl_3$): δ 195.2, 151.4, 149.9, 136.2, 123.5, 41.7, 32.4 (2C), 26.1 (2C), 25.9, 9.52 ppm.

**HRMS (ESI$^{+}$):** calculated for $[M+Na]^{+}$ ($C_{12}H_{18}ONa^{+}$) required *m/z* 201.1250, found *m/z* 201.1248.

**FT-IR (neat) $\nu_{max}$:** 2922, 2850, 1674, 1630, 1447, 1405, 1379, 1355, 1328, 1260, 1223, 1202, 1173, 1148, 1128, 1098, 1009, 993, 964, 898, 834, 792, 774, 687, 563, 528, 490, 452, 441, 426 $cm^{-1}$.

### 4.6.4. (*R*,4*E*,6*E*)-1-((*S*)-4-Benzyl-2-thioxothiazolidin-3-yl)-7-cyclohexyl-3-hydroxy-4-methylhepta-4,6-dien-1-one (S-78)

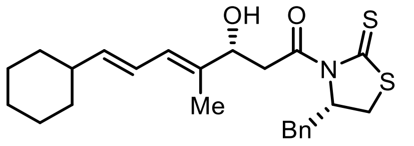


Synthesized according to **General Procedure K** on 3.94 mmol scale to yield **S-78** (504 mg, 1.17 mmol, 30%) as a yellow oil.

**$^1$H-NMR** (600 MHz, $CDCl_3$): δ 7.37 – 7.34 (m, 2H), 7.30 – 7.27 (m, 3H), 6.22 (ddd, *J* = 15.1, 10.8, 1.1 Hz, 1H), 6.10 (bd, *J* = 10.9 Hz, 1H), 5.68 (dd, *J* = 15.1, 7.1 Hz, 1H), 5.38 (ddd, *J* = 10.7, 7.0, 4.0 Hz, 1H), 4.64 (dt, *J* = 10.0, 2.8 Hz, 1H), 3.55 (dd, *J* = 17.2, 2.6 Hz, 1H), 3.43 (dd, *J* = 17.4, 9.6 Hz, 1H), 3.42 – 3.39 (m, 1H), 3.25 (dd, *J* = 13.2, 3.8 Hz, 1H), 3.05 (dd, *J* = 13.2, 10.5 Hz, 1H), 2.90 (d, *J* = 11.5 Hz, 1H), 2.50 (d, *J* = 3.6 Hz, 1H), 2.06 – 2.01 (m, 1H), 1.79 (s, 3H), 1.73 (bd, *J* = 10.5 Hz, 4H), 1.65 (d, *J* = 12.8 Hz, 1H), 1.31 – 1.06 (m, 5H) ppm.

**$^{13}$C-NMR** (151 MHz, $CDCl_3$): δ 201.5, 173.0, 142.0, 136.6, 135.4, 129.6 (2C), 129.1 (2C), 127.4, 126.2, 123.3, 72.9, 68.7, 44.5, 41.3, 37.0, 33.1, 32.3, 26.3 (2C), 26.1 (2C), 13.1 ppm.

**HRMS (ESI$^+$):** calculated for $[M+Na]^+$ ($C_{24}H_{31}NO_2S_2Na^+$) required *m/z* 452.1688, found *m/z* 452.1694.

**FT-IR (neat) $\nu_{max}$**: 2922, 2850, 2362, 2335, 1692, 1633, 1495, 1448, 1342, 1318, 1292, 1259, 1226, 1191, 1163, 1137, 1075, 1043, 1012, 966, 894, 748, 724, 701, 668, 580, 521, 511, 478, 418 $cm^{-1}$.

**$[a]_D^{20}$** = +8.20° (c = 1.00, $CHCl_3$).

### 4.6.5. (*R*,4*E*,6*E*)-*N*-((2*R*,3*S*)-2-((*tert*-Butyldimethylsilyl)oxy)-3-methyl-4-oxo-4-(((*E*)-4-(4,4,5,5-tetramethyl-1,3,2-dioxaborolan-2-yl)but-3-en-1-yl)amino)butyl)-7-cyclohexyl-3-hydroxy-4-methylhepta-4,6-dienamide (S-79)

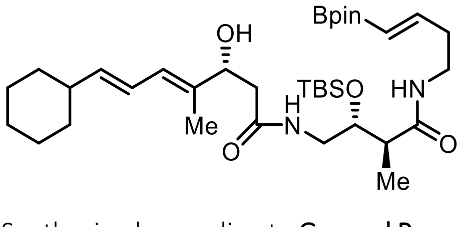


Synthesized according to **General Procedure L** on 0.97 mmol scale to yield **S-79** (364 mg, 0.563 mmol, 58%) as a colourless foam.

**$^1$H-NMR** (600 MHz, $CDCl_3$): δ 6.55 (dt, *J* = 18.0, 6.5 Hz, 1H), 6.44 (dd, *J* = 7.1, 4.3 Hz, 1H), 6.19 (ddd, *J* = 15.2, 10.8, 1.0 Hz, 1H), 6.14 (t, *J* = 5.5 Hz, 1H), 6.07 (d, *J* = 10.8 Hz, 1H), 5.65 (dd, *J* = 15.1, 7.0 Hz, 1H), 5.52 (d,

*J* = 18.0 Hz, 1H), 4.43 (dd, *J* = 8.9, 3.0 Hz, 1H), 3.88 (dt, *J* = 7.7, 4.4 Hz, 1H), 3.72 (ddd, *J* = 13.7, 7.7, 4.2 Hz, 1H), 3.47 – 3-42 (m, 1H), 3.30-3.24 (m, 1H), 2.93 (ddd, *J* = 13.6, 7.6, 4.5 Hz, 1H), 2.44 – 2.34 (m, 5H), 2.05 – 1.99 (m, 1H), 1.75 – 1.71 (m, 7H), 1.27 – 1.25 (m, 15H), 1.16 – 1-06 (m, 3H), 1.14 (d, *J* = 7.2 Hz, 3H), 0.92 – 0.88 (m, 9H), 0.17 – 0.07 (m, 6H) ppm.

**$^{13}$C-NMR** (151 MHz, $CDCl_3$): δ 174.6, 172.3, 150.4, 141.8, 135.7, 126.0, 123.3, 121.7 (*deducted from HSQC*), 83.4 (2C), 73.7, 72.2, 44.8, 43.0, 41.6, 41.3, 38.0, 35.8, 33.1 (2C), 26.3 (2C), 26.1 (3C), 26.0 (3C), 25.0, 24.9, 18.1, 15.4, 12.8, −4.4, −4.8 ppm.

**HRMS (ESI$^+$):** calculated for $[M+Na]^+$ ($C_{35}H_{63}BN_2O_6SiNa^+$) required *m/z* 669.4441, found *m/z* 669.4450.

**FT-IR (neat) $\nu_{max}$**: 3335, 3307, 2976, 2927, 2854, 2361, 2340, 1643, 1540, 1471, 1448, 1397, 1389, 1360, 1322, 1257, 1144, 1102, 1042, 1023, 1003, 967, 836, 812, 778, 749, 681, 667, 655, 579 cm$^{-1}$.

**$[a]_D^{20}$** = +27.6° (c = 1.00, $CHCl_3$).

### 4.6.6. (*R*,4*E*,6*E*)-1-(((2*R*,3*S*)-2-((*tert*-Butyldimethylsilyl)oxy)-3-methyl-4-oxo-4-(((*E*)-4-(4,4,5,5-tetramethyl-1,3,2-dioxaborolan-2-yl)but-3-en-1-yl)amino)butyl)amino)-7-cyclohexyl-4-methyl-1-oxohepta-4,6-dien-3-yl (1*R*,4*R*)-4-chlorocyclobut-2-ene-1-carboxylate (S-80)

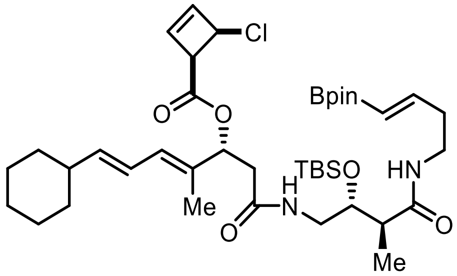


Synthesized according to **General Procedure M** on 79.0 µmol scale to yield **S-80** (26.3 mg, 34.5 µmol, 44%) as a colourless foam.

**$^1$H-NMR** (600 MHz, $CDCl_3$): δ 6.55 (dt, *J* = 17.9, 6.4 Hz, 1H), 6.29 – 6.23 (m, 2H), 6.17 (t, *J* = 5.7 Hz, 1H), 6.16 – 6.09 (m, 3H), 5.69 – 5.65 (m ,1H), 5.63 (dd, *J* = 7.8, 5.4 Hz, 1H), 5.51 (d, *J* = 18.0 Hz, 1H), 5.08 (d, *J* = 4.2 Hz, 1H), 4.08 (d, *J* = 4.3 Hz, 1H), 3.83 – 3.80 (m, 1H), 3.75 (ddd, *J* = 13.4, 8.0, 4.0 Hz, 1H), 3.45 – 3.39 (m, 1H), 3.27 (td, *J* = 12.5, 6.7 Hz, 1H), 2.81 (ddd, *J* = 13.5, 7.7, 4.2 Hz, 1H), 2.66 (dd, *J* = 14.5, 7.9 Hz, 1H), 2.50 (dd, *J* = 14.5, 5.3 Hz, 1H), 2.38 – 2.33 (m, 3H), 2.03 – 1.97 (m, 1H), 1.77 (s, 3H), 1.70 (s, 5H), 1.64 (d, *J* = 12.6 Hz, 1H), 1.25 (s, 12H), 1.14 (d, *J* = 6.4 Hz, 2H), 1.10 (d+m, *J* = 7.1 Hz, 5H), 0.90 (s, 9H), 0.14 (s, 3H), 0.08 (s, 3H) ppm.

**$^{13}$C-NMR** (151 MHz, $CDCl_3$): δ 174.5, 169.3, 168.8, 150.4, 142.9, 140.6, 136.7, 130.9, 129.2, 122.9, 121.7 (*deduced from HSQC*), 83.6 (2C), 77.0, 72.1, 56.4, 54.1, 44.6, 43.0, 41.3, 40.9, 38.0, 35.9, 33.0, 32.9, 26.3, 26.1, 26.0 (3C), 25.9, 24.94 (4C), 18.1, 15.5, 12.8, −4.3, −4.9 ppm.

**HRMS (ESI$^+$):** calculated for $[M+Na]^+$ ($C_{40}H_{66}BClN_2O_7SiNa^+$) required *m/z* 783.4319, found *m/z* 783.4324.

### 4.6.7. (2*R*,7*R*,8*S*,13*E*,15*E*,17*E*)-7-((*tert*-Butyldimethylsilyl)oxy)-2-((2*E*,4*E*)-5-cyclohexylpenta-2,4-dien-2-yl)-8-methyl-1-oxa-5,10-diazacyclononadeca-13,15,17-triene-4,9,19-trione (S-81)

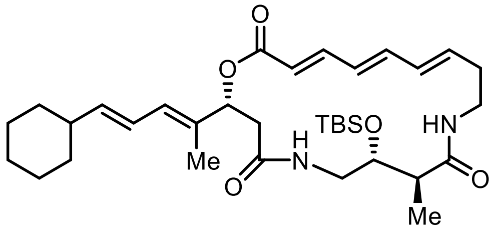


Synthesized according to **General Procedure N** on 15.8 µmol scale to yield **S-81** (8.30 mg, 13.9 µmol, 88%) as a colourless foam.

**$^1$H-NMR** (700 MHz, $CDCl_3$): δ 7.29 – 7.23 (m, 1H), 6.45 (dd, *J* = 14.9, 10.9 Hz, 1H), 6.22 – 6.17 (m, 1H), 6.15 (dd, *J* = 13.2, 9.5 Hz, 1H), 6.14 – 6.07 (m, 3H), 6.02 (bs, 1H), 5.90 (ddd, *J* = 14.7, 10.9, 3.4 Hz, 1H), 5.71 (d, *J* = 15.6 Hz, 1H), 5.69 (dd, *J* = 15.3, 6.9 Hz, 1H), 5.62 (dd, *J* = 11.1, 3.2 Hz, 1H), 4.11 – 4.04 (m, 1H), 3.51 – 3.40 (m, 2H), 2.96 (bd, *J* = 13.9 Hz, 1H), 2.56 – 2.36 (m, 4H), 2.23 – 2.15 (m, 1H), 2.05 – 1.99 (m, 1H), 1.79 (s, 3H), 1.72 (bd, *J* = 10.2 Hz, 4H), 1.68 – 1.63 (m, 2H), 1.25 (s, 3H), 1.14 (dd, *J* = 6.9, 3.4 Hz, 3H), 1.12 – 1.05 (m, 2H), 0.87 (s, 9H), 0.09 (s, 3H), 0.06 (s, 3H) ppm.

**$^{13}$C-NMR** (176 MHz, $CDCl_3$): δ 176.6, 174.4, 170.0, 166.4, 146.7, 143.2, 142.4, 138.7, 132.3, 131.6, 127.7, 126.8, 123.1, 119.5, 76.4, 72.4, 45.2, 43.1, 42.2, 41.3, 37.5, 34.4, 33.9, 29.9, 25.9, 19.4, 18.7, 18.0, 14.3 (3C), 13.4, −4.5, −4.8 ppm.

**HRMS (ESI$^+$):** calculated for $[M+Na]^+$ ($C_{34}H_{54}N_2O_5SiNa^+$) required *m/z* 621.3700, found *m/z* 621.3685.

**FT-IR (neat) $ν_{max}$:** 2957, 2925, 2853, 1712, 1651, 1614, 1538, 1467, 1378, 1361, 1256, 1228, 1218, 1186, 1144, 1101, 1009, 966, 837, 776, 751 $cm^{-1}$.

**$[a]_D^{20}$** = −85.9° (c = 0.300, $CHCl_3$).

### 4.6.8. Fs-FR6

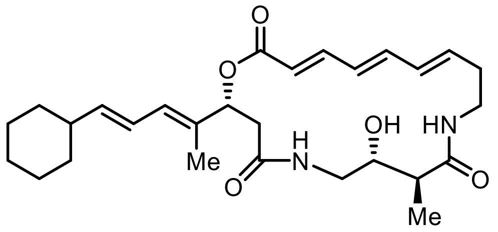

Synthesized according to **General Procedure O** on 17.9 µmol scale to yield **Fs-FR6** (6.30 mg, 13.0 µmol, 73%) as a white solid.

**$^{1}$H-NMR** (700 MHz, $CDCl_3$): δ 7.28 – 7.25 (m, 1H), 6.50 (dd, *J* = 14.8, 10.8 Hz, 1H), 6.26 (d, *J* = 8.5 Hz, 1H), 6.21 – 6.16 (m, 2H), 6.12 (d, *J* = 10.8 Hz, 1H), 6.07 (dd, *J* = 14.6, 11.2 Hz, 1H), 5.96 (bs, 1H), 5.81 – 5.74 (m, 1H), 5.76 (d, *J* = 15.4 Hz, 1H), 5.71 – 5.65 (m, 3H), 4.14 (tdd, *J* = 13.6, 10.4, 5.1 Hz, 1H), 3.83 – 3.78 (m, 1H), 3.33 (td, *J* = 10.5, 2.9 Hz, 1H), 3.04 – 2.98 (m, 1H), 2.65 – 2.61 (m, 1H), 2.61 – 2.56 (m, 1H), 2.56 (dd, *J* = 14.0, 3.2, Hz, 1H), 2.50 (dd, *J* = 13.8, 11.4 Hz, 1H), 2.29 – 2.23 (m, 1H), 2.23 – 2.17 (m, 1H), 2.06 – 2.00 (m, 1H), 1.79 (s, 3H), 1.72 (d, *J* = 10.5 Hz, 1H), 1.64 (dt, *J* = 15.1, 7.5 Hz, 3H), 1.34 – 1.25 (m, 4H), 1.12 – 1.05 (m, 2H), 0.98 (d, *J* = 7.1 Hz, 3H) ppm.

**$^{13}$C-NMR** (176 MHz, $CDCl_3$): δ 176.0, 169.4, 165.4, 144.5, 142.4, 140.8, 136.0, 133.0, 132.2, 128.7, 127.0, 123.1, 121.3, 76.1, 71.6, 44.1, 43.1, 42.0, 41.3, 36.9, 34.6, 32.1, 29.9, 26.1, 24.9, 22.9, 13.3, 11.1 ppm.

**HRMS (ESI$^{+}$):** calculated for $[M+Na]^{+}$ ($C_{28}H_{40}N_2O_5Na^{+}$) required *m/z* 507.2835, found *m/z* 507.2838.

**FT-IR (neat) $\nu_{max}$:** 3020, 2961, 2923, 2852, 2359, 1705, 1643, 1630, 1613, 1540, 1249, 1229, 1218, 1005, 767, 755 $cm^{-1}$.

**$[a]_D^{20}$** = −242° (c = 0.050, $CHCl_3$).

## 4.7. Fs-FR7 Synthesis

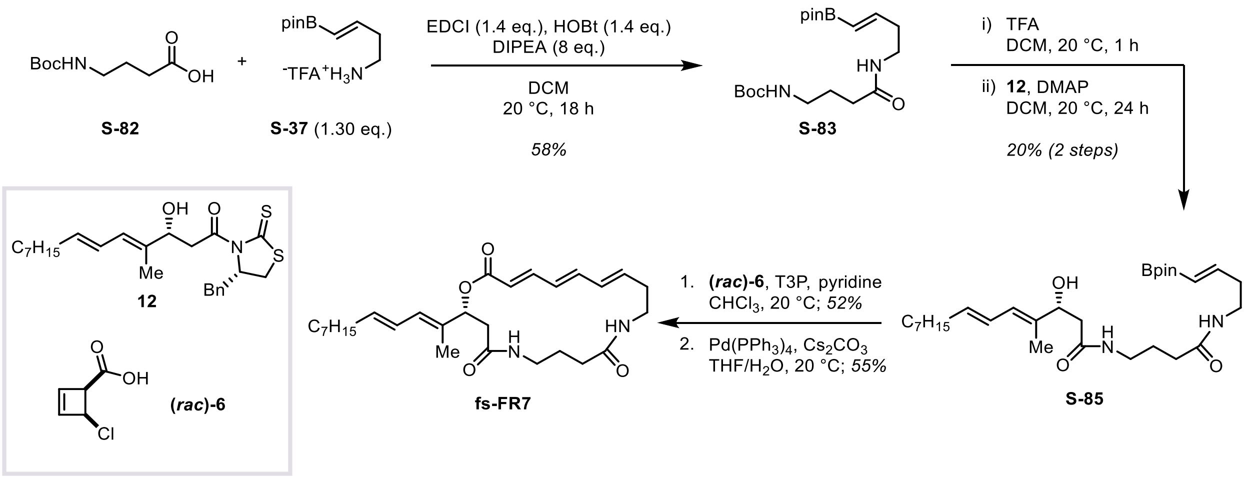


### 4.7.1. *tert*-Butyl (*E*)-(4-oxo-4-((4-(4,4,5,5-tetramethyl-1,3,2-dioxaborolan-2-yl)but-3-en-1-yl)amino)butyl)carbamate (S-83)

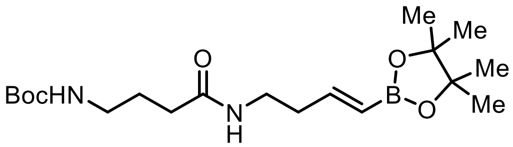


To a solution of 4-((*tert*-butoxycarbonyl)amino)butanoic acid **S-82** (0.793 g, 3.90 mmol, 1.00 equiv.) and ammonium salt **S-37** (1.00 g, 5.07 mmol, 1.30 equiv.) in $CH_2Cl_2$ (0.2 M) at 0 °C was added DIPEA (5.40 mL, 31.2 mmol, 8.00 equiv.), followed by EDCI (1.12 g, 5.85 mmol, 1.40 equiv.) and HOBt (88% w/w, 0.900 g, 5.85 mmol, 1.40 equiv.). The resulting reaction mixture was allowed to warm to room temperature and stirring was continued for 18 h. After the starting material had been consumed, the reaction was diluted with water and the layers were separated. The aqueous layer was extraced with $CH_2Cl_2$ (3 x 15 mL) and the combined organic layers were washed with brine before being dried over $Na_2SO_4$. The solids were filtered off and the volatiles were removed under reduced pressure to yield the crude material, which was subsequently purified by column chromatography ($SiO_2$, $CH_2Cl_2$/EtOAc 4% → 10%) to yield amide **S-83** (861 mg, 2.26 mmol, 58%) as a colorless foam.

All spectroscopic data in accordance with the literature.[1]

**$^1$H NMR** (600 MHz, $CDCl_3$): δ 6.55 (dt, *J* = 18.0, 6.5 Hz, 1H), 5.99 (br. s, 1H), 5.51 (d, *J* = 18.0 Hz, 1H), 4.76 (br. s, 1H), 3.35 (q, *J* = 6.6 Hz, 2H), 3.15 (q, *J* = 6.6 Hz, 2H), 2.37 (q, *J* = 6.6 Hz, 2H), 2.18 (t, *J* = 6.2 Hz, 2H), 1.79 (p, *J* = 6.8 Hz, 2H), 1.43 (s, 9H), 1.26 (s, 12H) ppm.

### 4.7.2. *tert*-Butyl (*E*)-(4-oxo-4-((4-(4,4,5,5-tetramethyl-1,3,2-dioxaborolan-2-yl)but-3-en-1-yl)ammonium trifluoroacetate (S-84)

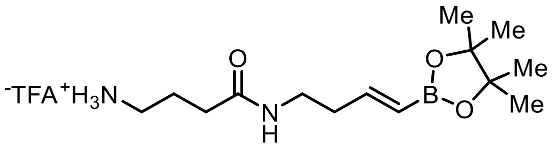


Synthesized according to **General Procedure D** on 1.90 mmol scale to yield **S-84**, which was used in the next step without further purification.

### 4.7.3. (*R*,4*E*,6*E*)-3-Hydroxy-4-methyl-*N*-(4-oxo-4-(((*E*)-4-(4,4,5,5-tetramethyl-1,3,2-dioxaborolan-2-yl)but-3-en-1-yl)amino)butyl)tetradeca-4,6-dienamide (S-85)

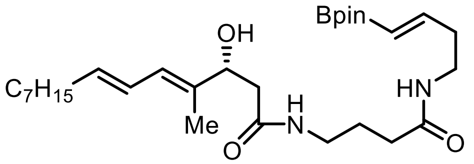


Synthesized according to **General Procedure L** on 366 µmol scale to yield **S-85** (38.0 mg, 73.3 µmol, 20%) as a colorless foam.

All spectroscopic data in accordance with the literature.[1]

**$^1$H NMR** (600 MHz, $CDCl_3$): δ 6.59 – 6.52 (m, 2H), 6.22 (dd, *J* = 15.0, 10.9 Hz, 1H), 6.13 (br. s, 1H), 6.08 (d, *J* = 10.8 Hz, 1H), 5.73 – 5.66 (m, 1H), 5.52 (d, *J* = 18.0 Hz, 1H), 4.43 (d, *J* = 8.8 Hz, 1H), 3.38 – 3.24 (m, 4H), 2.40 – 2.34 (m, 4H), 2.21 (t, *J* = 6.6 Hz, 2H), 2.09 (q, *J* = 7.2 Hz, 2H), 1.86 – 1.80 (m, 2H), 1.73 (s, 3H), 1.40 – 1.33 (m, 2H), 1.31 – 1.21 (m, 22H), 0.88 (t, *J* = 7.0 Hz, 3H) ppm.

### 4.7.4. (*R*,4*E*,6*E*)-4-Methyl-1-oxo-1-((4-oxo-4-(((*E*)-4-(4,4,5,5-tetramethyl-1,3,2-dioxaborolan-2-yl)but-3-en-1-yl)amino)butyl)amino)tetradeca-4,6-dien-3-yl (1*R*,4*R*)-4-chlorocyclobut-2-ene-1-carboxylate (S-86)

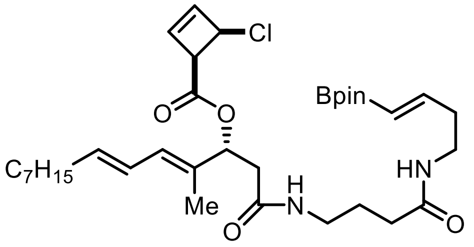

Synthesized according to **General Procedure M** on 154 µmol scale to yield **S-86** (51.7 mg, 79.6 µmol, 52%, mixture of *cis*-diastereomers) as a colorless foam.

All spectroscopic data in accordance with the literature.[1]

**$^{1}$H NMR** (600 MHz, $CDCl_3$): δ 6.54 (dt, *J* = 18.0, 6.5 Hz, 1H), 6.25 (br. s, 3H), 6.18 – 6.10 (m, 3H), 5.71 (dt, *J* = 14.2, 6.9 Hz, 1H), 5.61 (dd, *J* = 8.1, 5.0 Hz, 1H), 5.51 (d, *J* = 18.0 Hz, 1H), 5.08 (d, *J* = 4.3 Hz, 1H), 4.06 (d, *J* = 4.3 Hz, 1H), 3.35 (q, *J* = 6.6 Hz, 2H), 2.65 (dd, *J* = 14.6, 8.3 Hz, 1H), 2.51 (dd, *J* = 14.7, 5.0 Hz, 1H), 2.37 (q, *J* = 6.6 Hz, 2H), 2.16 (t, *J* = 6.8 Hz, 2H), 2.07 (t, *J* = 7.2 Hz, 2H), 1.76 (s, 3H), 1.79 – 1.73 (masked, 2H), 1.40 – 1.32 (m, 2H), 1.30 – 1.23 (m, 22H), 0.88 (t, *J* = 6.9 Hz, 3H) ppm.

### 4.7.5. Fs-FR7

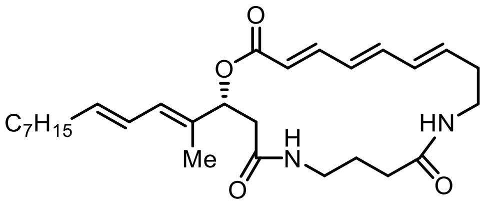


Synthesized according to **General Procedure N** on 26.9 µmol scale to yield **Fs-FR7** (7.0 mg, 14.8 µmol, 55%) as a white powder.

**$^{1}$H NMR** (400 MHz, $CDCl_3$): δ 7.21 (dd, *J* = 15.3, 11.3 Hz, 1H), 6.47 (dd, *J* = 14.9, 10.5 Hz, 1H), 6.27 – 6.05 (m, 4H), 5.87 – 5.69 (m, 3H), 5.60 (dd, *J* = 11.3, 3.5 Hz, 1H), 5.53 – 5.48 (m, 1H), 5.27 (dd, *J* = 9.7, 2.1 Hz, 1H), 4.15 – 3.97 (m, 1H), 3.29 – 3.16 (m, 1H), 3.02 – 2.90 (m, 2H), 2.61 – 2.51 (m, 2H), 2.43 (dd, *J* = 13.9, 11.3 Hz, 1H), 2.22 – 2.06 (m, 4H), 1.89 – 1.79 (m, 2H), 1.78 (s, 3H), 1.43 – 1.34 (m, 2H), 1.33 – 1.24 (m, 8H), 0.88 (t, *J* = 6.9 Hz, 4H) ppm.

**$^{13}$C NMR** (151 MHz, $CDCl_3$): δ 172.7, 169.4, 165.6, 144.9, 141.5, 137.1, 136.8, 132.6, 131.9, 128.2, 126.8, 125.6, 120.7, 76.2, 41.9, 39.8, 37.2, 34.8, 33.8, 33.2, 32.0, 31.4, 31.1, 29.9, 29.50, 29.33, 26.7, 22.8, 14.3, 13.3 ppm.

## 4.8. Fs-FR8 Synthesis

### 4.8.1. (*R*)-1-(4-Benzyl-2-thioxothiazolidin-3-yl)ethan-1-one ((*R*)-S-3)

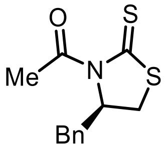


To an aqueous solution of KOH (10.0 equiv.) in water (5 M) at 20 °C was added enantiopure alcohol (*R*)-**S-1** (1.00 equiv.) followed by $CS_2$ (5.00 equiv.). The resulting reaction mixture was heated to 100 °C and stirring at the indicated temperature was continued for 72 h. Afterwards, the reaction mixture was allowed to cool to room temperature and was extracted with $CH_2Cl_2$ (4 x equal volume to $H_2O$). The combined organic layers were dried over $Na_2SO_4$, the solids were filtered off and the solvents were removed *in vacuo* to yield the crude material. The crude product was purified by recrystallisation from $Et_2O$ to yield (*R*)-4-benylthiazolidine-4-thione (not shown).

(*R*)-4-benylthiazolidine-4-thione (1.00 equiv.) was dissolved in THF (0.3 M) and the resulting yellow solution was cooled to 0 °C. NaH (60 wt% in paraffin) (1.10 equiv.) was carefully added to the cold solution and the resulting mixture was stirred at 0 °C for one hour or until $H_2$ evolution ceased. Subsequently, AcCl (1.50 equiv.) was added to the cold reaction mixture and the resulting solution was allowed to warm to room temperature. Stirring was continued for two hours or until the starting material had been consumed (as indicated by TLC analysis) and the reaction mixture was subsequently quenched by careful addition of a saturated aqueous solution of $NH_4Cl$. The quenched reaction mixture was diluted with EtOAc and the layers were separated. The aqueous layer was extracted with EtOAc (3 x equal volume to THF) and the combined organic layers were washed with brine (1 x equal volume to THF). The organic layers were dried over $Na_2SO_4$, the solids were filtered off and the solvents were removed *in vacuo* to yield the crude auxiliary (*R*)-**S-3**. Purification was achieved by recrystallisation from $Et_2O$ to yield (*R*)-**S-3** as yellow crystals.

Synthesis conducted on 26.0 mmol scale to yield 3.55 g (14.0 mmol, 54% (2 steps)) of (*R*)-**S-3**.
All spectroscopic data in accordance with the literature.[1]
**[a]**$_D^{20}$ = −210° (c = 1.00, $CHCl_3$).

### 4.8.2. (*S*,4*E*,6*E*)-1-((*R*)-4-Benzyl-2-thioxothiazolidin-3-yl)-3-hydroxy-4-methyltetradeca-4,6-dien-1-one (*ent*-12)

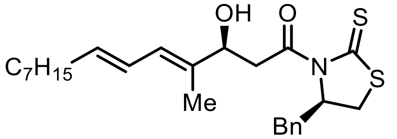


Synthesized according to **General Procedure K** on 0.398 mmol scale to yield ***ent*-12** (117 mg, 0.263 mmol, 66%) as a yellow oil.

All spectroscopic data in accordance with the literature.[1]

**[a]**$_D^{20}$ = −61.4° (c = 1.00, $CHCl_3$).

### 4.8.3. Ethyl (*S*)-4-azido-3-hydroxybutanoate ((*S*)-S-38)

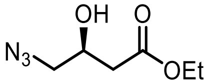


Synthesized according to **General Procedure E** on 28.5 mmol scale to yield (*S*)-**S-38** (4.00 g, 23.1 mmol, 81%) as a colourless oil.

All spectroscopic data in accordance with the literature.[1]

**[a]**$_D^{20}$ = −17.4° (c = 0.44, $CHCl_3$).

### 4.8.4. Ethyl (2*R*,3*S*)-4-azido-3-hydroxy-2-methylbutanoate (*ent*-S-39)

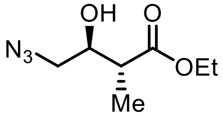


Synthesized according to **General Procedure F** on 21.2 mmol scale to yield ***ent*-S-39** (2.20 g, 11.0 mmol, 52%) as a colourless oil.

All spectroscopic data in accordance with the literature.[1]

**[a]**$_D^{20}$ = −3.8° (c = 1.25, $CHCl_3$).

### 4.8.5. Ethyl (2*R*,3*S*)-4-azido-3-((*tert*-butyldimethylsilyl)oxy)-2-methylbutanoate (*ent*-S-40)

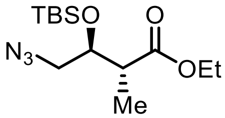

Synthesized according to **General Procedure G** on 12.0 mmol scale to yield ***ent*-S-40** (3.60 g, 11.9 mmol, 99%) as a colourless oil.

All spectroscopic data in accordance with the literature.[1]

$[a]_D^{20}$ = −25.7° (c = 1.10, $CHCl_3$).

### 4.8.6. (2*R*,3*S*)-4-Azido-3-((*tert*-butyldimethylsilyl)oxy)-2-methylbutanoic acid (*ent*-S-41)

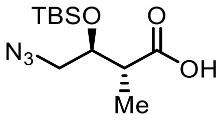


Synthesized according to **General Procedure H** on 11.9 mmol scale to yield ***ent*-S-41** (3.10 g, 11.3 mmol, 95%) as a colourless oil.

All spectroscopic data in accordance with the literature.[1]

$[a]_D^{20}$ = −18.9° (c = 1.07, $CHCl_3$).

### 4.8.7. (2*R*,3*S*)-4-Azido-3-((*tert*-butyldimethylsilyl)oxy)-2-methyl-*N*-((*E*)-4-(4,4,5,5-tetramethyl-1,3,2-dioxaborolan-2-yl)but-3-en-1-yl)butanamide (*ent*-16)

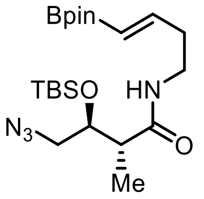


Synthesized according to **General Procedure I** on 5.00 mmol scale to yield ***ent*-16** (1.97 g, 4.35 mmol, 87%) as a colorless oil.

All spectroscopic data in accordance with the literature.[1]

$[a]_D^{20}$ = −12.9° (c = 1.87, $CHCl_3$).

### 4.8.8. (2*R*,3*S*)-4-Amino-3-((*tert*-butyldimethylsilyl)oxy)-2-methyl-*N*-((*E*)-4-(4,4,5,5-tetramethyl-1,3,2-dioxaborolan-2-yl)but-3-en-1-yl)butanamide (*ent*-2)

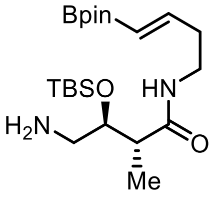

Synthesized according to **General Procedure J** on 4.35 mmol scale to yield ***ent*-2** as a colorless oil, which was used without further purification.

All spectroscopic data in accordance with the literature.[1]

### 4.8.9. (*S*,4*E*,6*E*)-*N*-((2*S*,3*R*)-2-((*tert*-Butyldimethylsilyl)oxy)-3-methyl-4-oxo-4-(((*E*)-4-(4,4,5,5-tetramethyl-1,3,2-dioxaborolan-2-yl)but-3-en-1-yl)amino)butyl)-3-hydroxy-4-methyltetradeca-4,6-dienamide (*ent*-S-42)

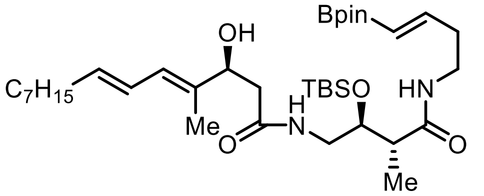


Synthesized according to **General Procedure L** on 125 µmol scale to yield ***ent*-S-42** (550 mg, 82.5 µmol, 66%) as a colorless oil.

All spectroscopic data in accordance with the literature.[1]

**[a]**$_D^{20}$ = −22.9° (c = 0.35, $CHCl_3$).

### 4.8.10. (*S*,4*E*,6*E*)-1-(((2*S*,3*R*)-2-((*tert*-Butyldimethylsilyl)oxy)-3-methyl-4-oxo-4-(((*E*)-4-(4,4,5,5-tetramethyl-1,3,2-dioxaborolan-2-yl)but-3-en-1-yl)amino)butyl)amino)-4-methyl-1-oxotetradeca-4,6-dien-3-yl (1*R*,4*S*)-4-chlorocyclobut-2-ene-1-carboxylate (*ent*-S-43)

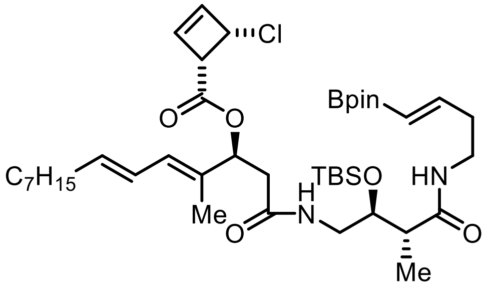


Synthesized according to **General Procedure M** on 140 µmol scale to yield ***ent*-S-43** (23.0 mg, 29.6 µmol, 21%, mixture of *cis*-diastereomers) as a colorless oil.

All spectroscopic data in accordance with the literature.[1]

### 4.8.11. (2*S*,7*S*,8*R*,13*E*,15*E*,17*E*)-7-((*tert*-butyldimethylsilyl)oxy)-2-((2*E*,4*E*)-dodeca-2,4-dien-2-yl)-8-methyl-1-oxa-5,10-diazacyclononadeca-13,15,17-triene-4,9,19-trione (*ent*-fs-FR11)

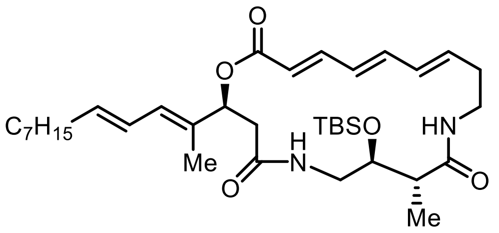


Synthesized according to **General Procedure N** on 49.0 µmol scale to yield ***ent*-fs-FR11** (13.4 mg, 21.8 µmol, 45%) as a colorless foam.

All spectroscopic data in accordance with the literature.[1]

**[a]**$_D^{29}$ = +56.0° (c = 1.00, $CHCl_3$).

### 4.8.12. Fs-FR8 (*ent*-FR252921)

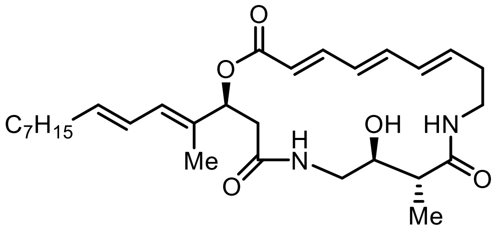


Synthesized according to **General Procedure O** on 10.6 µmol scale to yield **Fs-FR8** (4.90 mg, 9.79 µmol, 92%) as a colorless foam.

All spectroscopic data in accordance with the literature.[1]

**[a]**$_D^{20}$ = +83.8° (c = 1.00, $CHCl_3$).

## 4.9. Fs-FR9 Synthesis

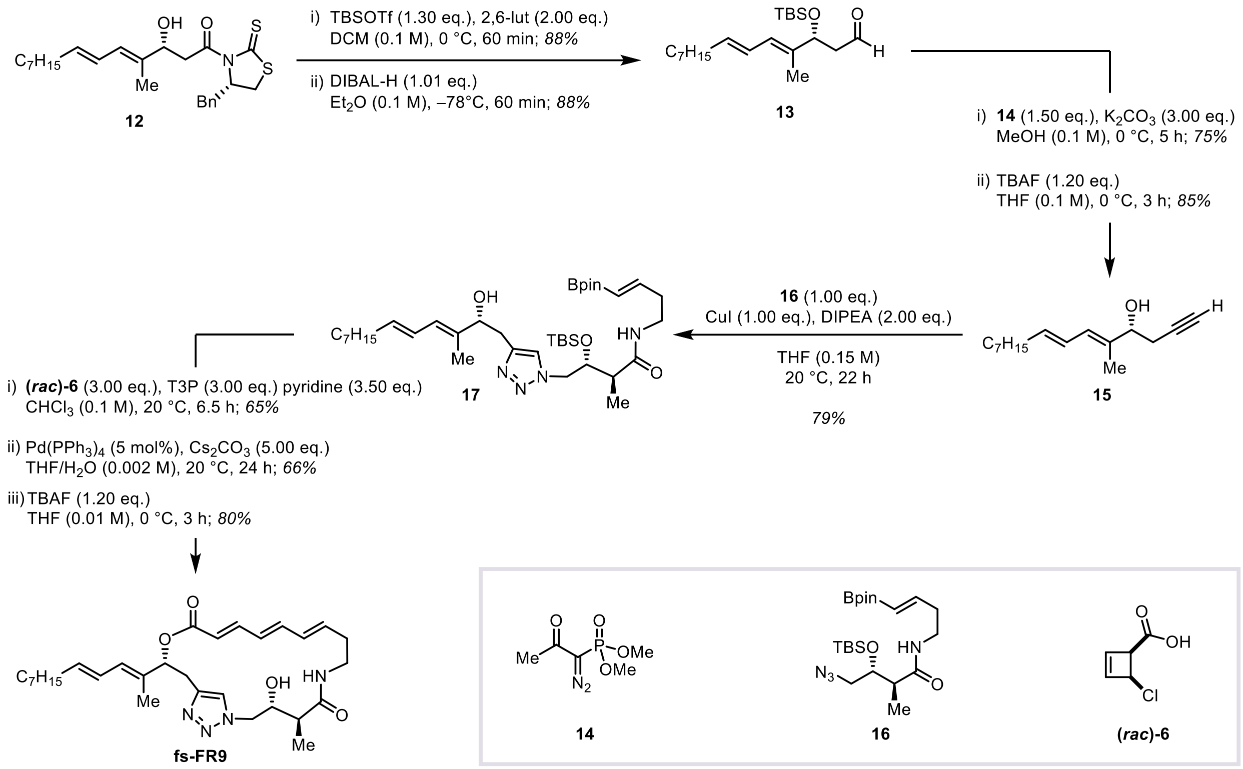


### 4.9.1. (*R*,4*E*,6*E*)-1-((*S*)-4-Benzyl-2-thioxothiazolidin-3-yl)-3-((*tert*-butyldimethylsilyl)oxy)-4-methyltetradeca-4,6-dien-1-one (S-87)

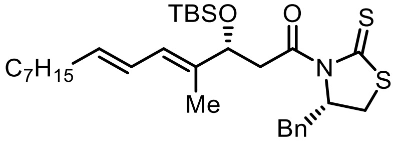

Synthesized according to **General Procedure G** on 2.00 mmol scale to yield **S-87** (1.02 g, 1.76 mmol, 88%) as a yellow oil.

**$^1$H-NMR** (600 MHz, $CDCl_3$): δ 7.35 (t, *J* = 7.4 Hz, 2H), 7.29 (d, *J* = 7.5 Hz, 3H), 6.20 (dd, *J* = 15.0, 10.8 Hz, 1H), 5.98 (d, *J* = 10.8 Hz, 1H), 5.66 (dt, *J* = 15.1, 7.5 Hz, 1H), 5.19 (ddd, *J* = 10.6, 6.9, 3.6 Hz, 1H), 4.70 (dd, *J* = 9.1, 3.5 Hz, 1H), 3.91 (dd, *J* = 15.8, 9.2 Hz, 1H), 3.32 (dd, *J* = 11.3, 7.1 Hz, 1H), 3.26 (dd, *J* = 13.2, 2.4 Hz, 1H), 3.03 (dd, *J* = 13.1, 10.9 Hz, 1H), 2.95 (dd, *J* = 15.8, 3.6 Hz, 1H), 2.87 (d, *J* = 11.5 Hz, 1H), 2.09 (q, *J* = 7.2 Hz, 2H), 1.73 (s, 3H), 1.38 – 1.36 (m, 2H), 1.32 – 1.27 (m, 8H), 0.89 – 0.84 (m, 12H), 0.06 (s, 3H), 0.01 (s, 3H) ppm.

**$^{13}$C-NMR** (151 MHz, $CDCl_3$): δ 201.3, 172.1, 136.8, 136.6, 135.6, 129.6 (2C), 129.1 (2C), 127.3, 126.1, 126.0, 75.3, 69.1, 45.5, 36.7, 33.1, 32.4, 32.0, 29.5, 29.4, 29.3, 25.9 (3C), 22.8, 18.2, 14.2, 12.1, −4.6, −5.0 ppm.

**HRMS (ESI$^+$):** calculated for $[M+Na]^+$ ($C_{31}H_{49}NO_2S_2SiNa^+$) required *m/z* 582.2872, found *m/z* 582.2862.

**FT-IR (neat) $\nu_{max}$:** 3027, 2953, 2925, 2854, 1698, 1496, 1455, 1438, 1359, 1340, 1283, 1251, 1225, 1190, 1161, 1136, 1076, 1039, 1006, 965, 941, 894, 835, 811, 776, 744, 701, 666, 610, 561, 548, 508, 483 $cm^{-1}$.

**[a]$_D^{20}$** = +128° (c = 1.00, $CHCl_3$).

### 4.9.2. (*R*,4*E*,6*E*)-3-((*tert*-Butyldimethylsilyl)oxy)-4-methyltetradeca-4,6-dienal (13)

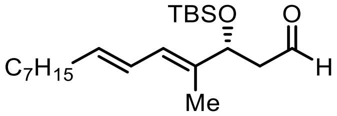


To a solution of TBS-aldol product **S-87** (1.71 g, 3.05 mmol, 1.00 equiv.) in $Et_2O$ (0.1 M) at −78 °C was added dropwise over five minutes DIBAL-H (1 M in toluene) (3.08 mL, 3.08 mmol, 1.01 equiv.). The resulting mixture was stirred at −78 °C for 60 min or until full conversion of the starting material was observed (as indicated by $^1$H-NMR or TLC analysis). After the starting material had been consumed, the reaction was quenched by addition of 0.200 mL of water. The resulting mixture was stirred at −78 °C for 5 min before being allowed to warm to 20 °C. Afterwards, $MgSO_4$ was added and the reaction mixture was stirred until an opaque gel formed. The opaque gel was then filtered through a short pad of Celite® and the filter cake was washed with $Et_2O$. The filtrate was concentrated under reduced pressure and the crude product was purified by flash column chromatography ($SiO_2$, EtOAc/heptane or $Et_2O$/pentane) to afford **13** (946 mg, 2.68 mmol, 88%) as a colourless oil.

**$^1$H-NMR** (600 MHz, $CDCl_3$): δ 9.75 (t, *J* = 2.5 Hz, 1H), 6.19 (dd, *J* = 15.0, 10.9 Hz, 1H), 6.01 (d, *J* = 10.8 Hz, 1H), 5.68 (dt, *J* = 14.9, 7.4 Hz, 1H), 4.55 (dd, *J* = 8.2, 4.1 Hz, 1H), 2.67 (ddd, *J* = 15.4, 8.3, 2.9 Hz, 1H), 2.42 (ddd, *J* = 15.4, 4.1, 2.0 Hz, 1H), 2.10 (q, *J* = 7.2 Hz, 2H), 1.71 (s, 3H), 1.40 – 1.37 (m, 2H), 1.31 – 1.27 (m, 8H), 0.89 – 0.87 (m, 12H), 0.05 (s, 3H), 0.00 (s, 3H) ppm.

**$^{13}$C-NMR** (151 MHz, $CDCl_3$): δ 202.1, 135.9, 135.8, 126.1, 125.7, 73.8, 50.2, 33.2, 32.0, 29.5, 29.4, 29.3, 25.9 (3C), 22.8, 18.2, 14.2, 12.1, −4.5, −5.1 ppm.

**HRMS (ESI$^+$):** no fitting mass could be detected.

**FT-IR (neat) $\nu_{max}$:** 2954, 2923, 2853, 1728, 1681, 1599, 1461, 1377, 1252, 1075, 1002, 965,836, 776, 740, 669 $cm^{-1}$.

**[a]$_D^{20}$** = +2.13° (c = 1.00, $CHCl_3$).

### 4.9.3. *tert*-Butyldimethyl(((*R*,5*E*,7*E*)-5-methylpentadeca-5,7-dien-1-yn-4-yl)oxy)silane (S-88)

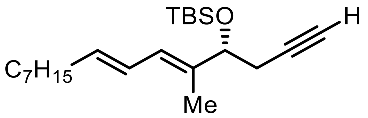


To a solution of aldehyde **13** (896 mg, 2.54 mmol, 1.00 equiv.) in MeOH (0.1 M) at 0 °C was added dimethyl-(1-diazo-2-oxopropyl)phosphonate (Bestmann-Ohira reagent, 10 wt% in MeCN) (9.15 mL, 3.81 mmol, 1.50 equiv.) and $K_2CO_3$ (1.05 g, 7.62 mmol, 3.00 equiv.). The resulting mixture was stirred at 0 °C for 3 h before being diluted with $Et_2O$ (25.0 mL) and water (25.0 mL). The layers were separated and the aqueous layer was extracted with $Et_2O$ (3 x 25.0 mL) and the combined organic layers were washed with brine (1 x 25.0 mL). The combined organic layers were dried over $Na_2SO_4$, the solids were filtered off and the solvents were removed under reduced pressure. The crude product was purified by flash column chromatography ($SiO_2$, EtOAc/heptane) to afford **S-88** (668 mg, 1.92 mmol, 75%) as a colourless oil.

**$^1$H-NMR** (600 MHz, $CDCl_3$): δ 6.21 (dd, *J* = 15.0, 10.9 Hz, 1H), 5.97 (d, *J* = 10.8 Hz, 1H), 5.67 (dt, *J* = 15.0, 7.5 Hz, 1H), 4.18 (t, *J* = 6.5 Hz, 1H) 2.40 (ddd, *J* = 16.6, 7.2, 2.6 Hz, 1H), 2.35 (ddd, *J* = 16.6, 6.0, 2.6 Hz, 1H), 2.10 (q, *J* = 7.2 Hz, 2H), 1.94 (t, *J* = 2.6 Hz, 1H), 1.69 (s, 3H), 1.30 – 1.37 (m, 2H), 1.31 – 1.26 (m, 8H), 0.90 – 0.84 (m, 12H), 0.07 (s, 3H), 0.01 (s, 3H) ppm.

**$^{13}$C-NMR** (151 MHz, $CDCl_3$): δ 136.1, 135.3, 126.3, 125.9, 82.1, 77.2, 69.8, 33.2, 32.0, 29.9, 29.5, 29.4, 27.2, 25.9 (3C), 22.8, 18.4, 14.3, 11.7, −4.6, −4.8 ppm.

**HRMS (ESI$^+$):** calculated for $[M+Na]^+$ ($C_{22}H_{40}OSiNa^+$) required *m/z* 371.2746, found *m/z* 371.2739.

**FT-IR (neat) $\nu_{max}$:** 3314,2954, 2925, 2854, 1713, 1683, 1462, 1361, 1251, 1221, 1073, 1006, 965, 936, 890, 836, 810, 776, 739, 668, 635 cm$^{-1}$.

**[a]$_D^{20}$** = −7.35° (c = 1.00, $CHCl_3$).

### 4.9.4. (*R*,5*E*,7*E*)-5-Methylpentadeca-5,7-dien-1-yn-4-ol (15)

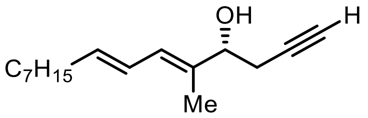


Synthesized according to **General Procedure O** on 0.220 mmol scale in THF (0.2 M) to yield **15** (45.0 mg, 0.192 mmol, 85%) as a colourless oil.

**$^1$H-NMR** (600 MHz, $CDCl_3$): δ 6.23 (ddt, *J* = 14.9, 10.8, 1.4 Hz, 1H), 6.08 (d, *J* = 10.9 Hz, 1H), 5.75-5.71 (m, 1H), 4.23 (t, *J* = 6.4 Hz, 1H), 2.48 (dd, *J* = 6.4, 2.6 Hz, 2H), 2.11 (q, *J* = 7.1 Hz, 2H), 2.05 (t, *J* = 2.6 Hz, 1H), 1.95 (b, 1H), 1.75 (d, *J* = 0.7 Hz, 3H), 1.39 – 1.38 (m, 2H), 1.30 – 1.26 (m, 8H), 0.91 (s, 3H) ppm.

**$^{13}$C-NMR** (151 MHz, $CDCl_3$): δ 136.4, 135.0, 126.6, 125.7, 81.0, 75.4, 70.8, 33.2, 32.0, 29.5, 29.34, 29.32, 26.1, 22.8, 14.2, 12.4 ppm.

**HRMS (ESI$^+$):** calculated for $[M+Na]^+$ ($C_{16}H_{26}ONa^+$) required *m/z* 257.1881, found *m/z* 257.1874.

**FT-IR (neat) $\nu_{max}$:** 3312, 2923, 2853, 1462, 1378, 1252, 1016, 964, 630 cm$^{-1}$.

**$[a]_D^{20}$** = −1.7° (c = 0.33, $CHCl_3$).

### 4.9.5. (2*S*,3*R*)-3-((*tert*-Butyldimethylsilyl)oxy)-4-(4-((*R*,3*E*,5*E*)-2-hydroxy-3-methyltrideca-3,5-dien-1-yl)-1*H*-1,2,3-triazol-1-yl)-2-methyl-*N*-((*E*)-4-(4,4,5,5-tetramethyl-1,3,2-dioxaborolan-2-yl)but-3-en-1-yl)butanamide (17)

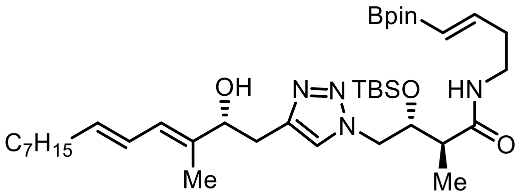


To a solution of FR core azide **16** (103 mg, 0.192 mmol, 1.00 equiv.) in THF (0.15 M) at 20 °C was added alkyne **15** (45.0 mg, 0.192 mmol, 1.00 equiv.), followed by addition of DIPEA (66.9 µL, 0.384 mmol, 2.00 equiv.) and CuI (36.8 mg, 0.192 mmol, 1.00 equiv.). The resulting cloudy reaction mixture was stirred at 20 °C for 22 h. Afterwards, the solvents were removed *in vacuo* and the resulting crude product was purified by flash column chromatography ($SiO_2$, 10 mL/0.1 mmol $C_v$, MeOH in $CH_2Cl_2$ gradient, 0% (1 $C_v$) → 1% (1 $C_v$) → 2% (2 $C_v$) → 4% (2 $C_v$) → 7% (2 $C_v$) → 10% (2 $C_v$)) to afford **21** (104 mg, 0.151 mmol, 79%) as a white foam.

**$^{1}$H-NMR** (600 MHz, $CDCl_3$): δ 7.53 (s, 1H), 6.55 (dt, *J* = 18.0, 6.5 Hz, 1H), 6.23 (dd, *J* = 15.0, 10.9 Hz, 1H), 6.07 (d, *J* = 10.7 Hz, 1H), 5.95 (t, *J* = 5.3 Hz, 1H), 5.72 – 5.64 (m, 1H), 5.52 (d, *J* = 18.0 Hz, 1H, **$H_{32}$**), 4.51 (dd, *J* = 14.0, 4.9 Hz, 1H), 4.38 (d, *J* = 8.1 Hz, 1H), 4.32 (dd, *J* = 14.0, 5.7 Hz, 1H), 4.24 (dd, *J* = 10.4, 5.2 Hz, 1H), 3.48 (dt, *J* = 13.5, 6.7 Hz, 1H), 3.24 (td, *J* = 12.1, 6.5 Hz, 1H), 2.97 (dd, *J* = 15.0, 3.8 Hz, 1H), 2.94 – 2.87 (m, 2H), 2.42 – 2.29 (m, 2H), 2.09 (q, *J* = 7.1 Hz, 3H), 1.81 (s, 3H), 1.41 – 1.36 (m, 2H), 1.31 – 1.24 (m, 20H), 1.18 (d, *J* = 7.1 Hz, 3H), 0.90 (s, 12H), 0.08 (s, 3H), 0.01 (s, 3H) ppm.

**$^{13}$C-NMR** (151 MHz, $CDCl_3$): δ 173.4, 150.4, 145.0, 136.1, 135.6, 126.0, 125.8, 123.9, 121.9 (*deduced from HSQC*), 83.4 (2C), 76.1, 73.0, 53.5, 44.9, 38.0, 35.8, 33.2, 32.0, 31.9, 29.6, 29.4, 29.3, 26.0, 25.0 (2C), 24.92, 24.91, 22.8, 18.1, 14.9, 14.3 (3C), 12.9, −4.64, −4.83 ppm.

**HRMS (ESI$^+$):** calculated for $[M+Na]^+$ ($C_{37}H_{67}N_4O_5BSiNa^+$) required *m/z* 709.4871, found *m/z* 709.4870.

**FT-IR (neat)** $\nu_{max}$: 2978, 2956, 2927, 2855, 1657, 1640, 1552, 1545, 1468, 1460, 1431, 1400, 1359, 1320, 1256, 1216, 1144, 1111, 1085, 1050, 1006, 992, 967, 941, 848, 834, 811, 779, 759 cm$^{-1}$.

$[a]_D^{20}$ = +10.7° (c = 0.45, $CHCl_3$).

### 4.9.6. (*R*,3*E*,5*E*)-1-(1-((2*R*,3*S*)-2-((*tert*-Butyldimethylsilyl)oxy)-3-methyl-4-oxo-4-(((*E*)-4-(4,4,5,5-tetramethyl-1,3,2-dioxaborolan-2-yl)but-3-en-1-yl)amino)butyl)-1*H*-1,2,3-triazol-4-yl)-3-methyltrideca-3,5-dien-2-yl (1*R*,4*R*)-4-chlorocyclobut-2-ene-1-carboxylate (S-89)

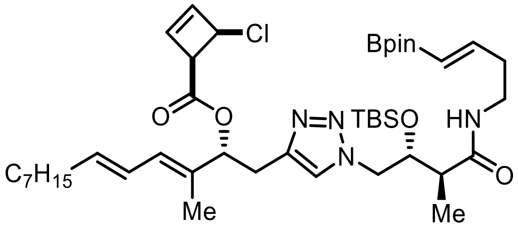


Synthesized according to **General Procedure M** on 0.141 mmol scale to yield **S-89** (65.2 mg, 92.0 µmol, 65%) as a colourless foam.

**$^1$H-NMR** (700 MHz, $CDCl_3$): δ 7.60 (s, 1H), 6.54 (dt, *J* = 17.9, 6.5 Hz, 1H), 6.31 (t, *J* = 5.5 Hz, 1H), 6.23 (d, *J* = 2.3 Hz, 1H), 6.18 – 6.16 (m, 2H), 6.09 (d, *J* = 10.8 Hz, 1H), 5.75 – 5.69 (m, 1H), 5.52 (dd, *J* = 9.1, 4.4 Hz, 1H), 5.49 (d, *J* = 18.0 Hz, 1H), 5.05 (d, *J* = 4.3 Hz, 1H), 4.61 (dd, *J* = 14.2, 3.8 Hz, 1H), 4.23 (dd, *J* = 14.2, 4.6 Hz, 1H), 4.17 (dt, *J* = 8.2, 4.2 Hz, 1H), 4.07 (d, *J* = 4.2 Hz, 1H), 3.47 (dd, *J* = 13.6, 6.5 Hz, 1H), 3.22 (dd, *J* = 14.9, 9.3 Hz, 1H), 3.14 (dt, *J* = 13.2, 7.0 Hz, 1H), 3.04 (dd, *J* = 14.9, 4.4 Hz, 1H), 2.34 (ddd, *J* = 7.5, 3.5, 1.6 Hz, 2H), 2.09 (dd, *J* = 13.7, 6.5 Hz, 2H), 1.83 (m, 4H), 1.39 – 1.35 (m, 2H), 1.28 – 1.24 (m, 20H), 1.11 (d, *J* = 7.0 Hz, 3H), 0.92 (s, 9H), 0.88 (t, *J* = 7.1 Hz, 3H), 0.10 (s, 3H), 0.08 (s, 3H) ppm.

**$^{13}$C-NMR** (176 MHz, $CDCl_3$): δ 173.6, 150.5, 143.6, 140.7, 137.0, 136.6, 131.5, 128.8, 126.0, 123.9, 121.8 (*deduced from HSQCs*), 83.3 (2C), 79.5, 73.0, 56.2, 54.1, 53.5, 44.1, 38.2, 35.7, 33.2, 32.0, 30.2, 29.47, 29.37, 29.31, 26.0 (4C), 24.9 (2C), 22.8, 18.1, 14.8, 14.3 (3C), 12.9, −4.4, −5.0 ppm.

**HRMS (ESI$^+$):** calculated for $[M+Na]^+$ ($C_{42}H_{70}N_4O_6BSiClNa^+$) required *m/z* 823.4744, found *m/z* 823.4742.

**FT-IR (neat)** $\nu_{max}$: 2954, 2927, 2855, 1733, 1658, 1640, 1359, 1322, 1287, 1259, 1232, 1168, 1144, 1083, 1049, 992, 967, 941, 835 cm$^{-1}$.

### 4.9.7. (14*Z*,3*R*,6*E*,8*E*,10*E*,16*S*,17*R*)-17-((*tert*-Butyldimethylsilyl)oxy)-3-((2*E*,4*E*)-dodeca-2,4-dien-2-yl)-16-methyl-11*H*-4-oxa-14-aza-1(4,1)-triazolacyclooctadecaphane-6,8,10-triene-5,15-dione (S-90)

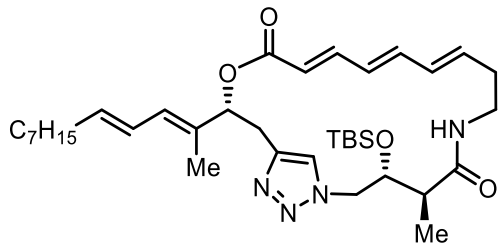


Synthesized according to **General Procedure N** on 50.0 µmol scale to yield **S-90** (21.2 mg, 33.2 µmol, 66%) as a colourless foam.

**$^{1}$H-NMR** (600 MHz, $CDCl_3$): δ 7.24 (s, 1H), 6.69 (dd, *J* = 15.3, 11.3 Hz, 1H), 6.41 (dd, *J* = 14.9, 10.6 Hz, 1H), 6.26 (dd, *J* = 14.9, 10.8 Hz, 1H), 6.16 (d, *J* = 10.8 Hz, 1H), 6.14 – 6.08 (m, 1H), 6.04 (dd, *J* = 15.0, 11.5 Hz, 1H), 6.03 – 5.97 (m, 1H), 5.83 (dd, *J* = 8.6, 4.1 Hz, 1H), 5.78 – 5.71 (m, 1H), 5.51 (dd, *J* = 9.1, 5.3 Hz, 1H), 5.49 (d, *J* = 15.5 Hz, 1H), 4.63 (ddd, *J* = 9.6, 3.7, 2.8 Hz, 1H), 4.04 – 3.88 (m, 4H), 3.14 – 3.08 (m, 2H), 2.61 (qd, *J* = 7.2, 4.0 Hz, 1H); 2.54 (bd, *J* = 14.2 Hz, 1H), 2.28 – 2.20 (m, 1H), 2.14 – 2.08 (m, 2H), 1.87 (s, 3H), 1.41 – 1.35 (m, 2H), 1.31 – 1.25 (m, 8H), 1.21 (d, *J* = 7.3 Hz, 3H), 0.92 (t, *J* = 7.4 Hz, 3H), 0.78 (s, 9H), −0.07 (s, 3H), −0.41 (s, 3H) ppm.

**$^{13}$C-NMR** (151 MHz, $CDCl_3$): δ 172.5, 166.2, 145.6, 144.0, 141.8, 138.6, 136.4, 132.8, 131.4, 127.5, 126.6, 125.8, 123.5, 120.0, 78.1, 76.4, 71.8, 53.2, 45.8, 37.8, 34.5, 33.18, 33.15, 32.0, 29.5, 29.3, 25.8, 25.0, 14.3 (3C), 13.3 (2C), 10.0, −5.1, −5.5 ppm.

**HRMS (ESI$^+$):** calculated for $[M+Na]^+$ ($C_{36}H_{58}N_4O_4SiNa^+$) required *m/z* 661.4125, found *m/z* 661.4119.

**FT-IR (neat)** $\nu_{max}$: 2956, 2925, 2854, 1710, 1654, 1640, 1616, 1533, 1528, 1467, 1462, 1432, 1387, 1360, 1298, 1249, 1234, 1216, 1144, 1111, 1080, 1051, 1007, 965, 939, 886, 837, 830, 809 cm$^{-1}$.

**[a]$_D^{20}$** = −13.9° (c = 0.054, $CHCl_3$).

### 4.9.8. Fs-FR9

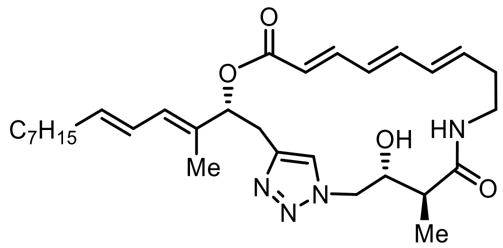

Synthesized according to **General Procedure O** on 24.0 µmol scale to yield **Fs-FR9** (10.1 mg, 19.2 µmol, 80%) as a white solid.

**$^1$H-NMR** (700 MHz, $CDCl_3$): δ 7.54 (s, 1H), 6.99 (dd, *J* = 15.3, 11.3 Hz, 1H), 6.43 (dd, 15.0, 10.7 Hz, 1H), 6.25 (dd, *J* = 14.9, 10.9 Hz, 1H), 6.17 (d, *J* = 10.8 Hz, 1H,), 6.15 (dd, *J* = 15.0, 11.3 Hz, 1H), 6.09 (dd, *J* = 15.1, 10.7 Hz, 1H), 5.87 (dt, *J* = 15.0, 7.5 Hz, 1H), 5.75 (dd, *J* = 14.6, 7.2 Hz, 1H), 5,71 (d, *J* = 15.3 Hz, 1H), 5.66 (t, *J* = 6.5 Hz, 1H), 5.48 (dd, *J* = 10.8, 3.2 Hz, 1H), 4.61 (d, *J* = 13.5 Hz, 1H), 3.99 (dd, *J* = 13.7, 10.5 Hz, 1H), 3.95 – 3.89 (m, 1H), 3.59 – 3.52 (m, 1H), 3.47 (dtd, *J* = 11.6, 6.7, 5.0 Hz, 1H), 3.41 (d, *J* = 3.3 Hz, 1H), 3.12 (dd, *J* = 14.8, 3.5 Hz, 1H), 3.04 (dd, *J* = 14.9, 10.9 Hz, 1H), 2.40 – 2.29 (m, 3H), 2.11 (q, *J* = 7.2 Hz, 2H), 1.85 (s, 3H), 1.41 – 1.36 (m, 2H), 1.33 – 1.26 (m, 8H), 1.22 (d, *J* = 7.0 Hz, 3H), 0.88 (t, *J* = 7.0 Hz, 3H) ppm.

**$^{13}$C-NMR** (176 MHz, $CDCl_3$): δ 174.3, 166.0, 144.5, 143.7, 140.4, 136.6, 136.5, 132.6, 132.0, 128.8, 126.9, 125.7, 124.7, 120.9, 77.6, 72.4, 54.5, 45.5, 38.1, 34.4, 33.2, 32.0, 30.7, 29.9, 29.5, 29.3, 22.8, 14.26, 14.24, 13.3 ppm.

**HRMS (ESI$^+$):** calculated for $[M+Na]^+$ ($C_{30}H_{44}N_4O_4Na^+$) required *m/z* 547.3260, found *m/z* 547.3265.

**FT-IR (neat) $ν_{max}$:** 3348, 2959, 2921, 2851, 1710, 1685, 1658, 1616, 1588, 1519, 1466, 1430, 1356, 1332, 1297, 1271, 1261, 1237, 1217, 1196, 1172, 1136, 1100, 1053, 1021, 998, 960, 878, 839, 795, 759, 720, 671, 616 $cm^{-1}$.

**[a]**$_D^{20}$ = −1.08° (c = 0.012, $CHCl_3$).

## 4.10. Fs-FR10

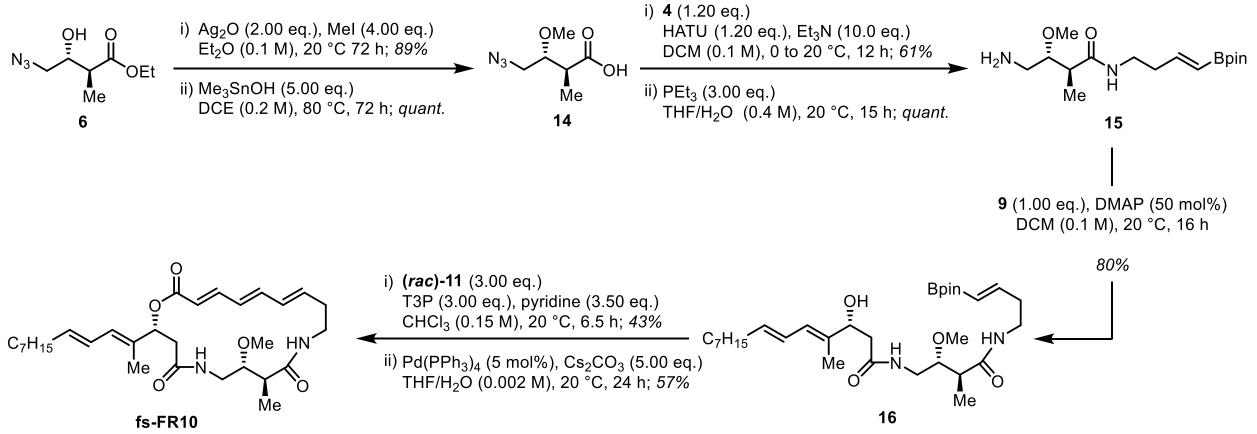


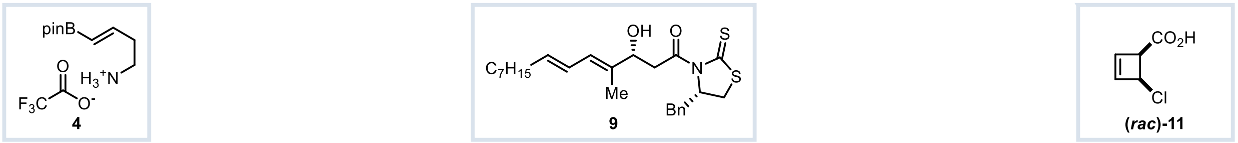


### 4.10.1. Ethyl (2*S*,3*R*)-4-azido-3-methoxy-2-methylbutanoate (S-91a)

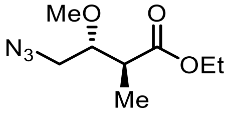

To a flame-dried Schlenk tube containing 4 Å molecular sieves was added dry $Et_2O$ and alcohol **S-39** (2.81 g, 15.0 mmol, 1.00 equiv.) under argon atmosphere, followed by $Ag_2O$ (6.95 g, 30.0 mmol, 2.00 equiv.) and MeI (3.74 mL, 60.0 mmol, 4.00 equiv.). The resulting reaction mixture was stirred in the dark for 3 days before being filtered over a short pad of celite (wash: 10% EtOAc in heptanes). The solvents were evaporated to afford **S-91a** (2.67 g, 2.50 mmol, 89%; d.r. 19:1) as a clear oil.

**$^1$H-NMR** (600 MHz, $CDCl_3$): δ 4.17 (q, *J* = 7.2 Hz, 2H), 3.58 (ddd, *J* = 7.3, 6.0, 3.2 Hz, 1H), 3.49 – 3.41 (m, 4H), 3.26 (dd, *J* = 13.2, 5.9 Hz, 1H), 2.79 (p, *J* = 7.2 Hz, 1H), 1.27 (t, *J* = 7.1 Hz, 3H), 1.12 (d, *J* = 7.2 Hz, 3H) ppm.

**$^{13}$C-NMR** (151 MHz, $CDCl_3$): δ 174.3, 81.9, 60.8, 58.4, 50.9, 42.0, 14.3, 12.8 ppm.

**HRMS (ESI$^+$):** calculated for $[M+Na]^+$ ($C_8H_{15}N_3O_3Na^+$) required *m/z* 224.1006, found *m/z* 224.1004.

**FT-IR (neat) $\nu_{max}$:** 2982, 2938, 2832, 2096, 1776, 1730, 1462, 1377, 1344, 1286, 1261, 1178, 1099, 1065, 1028, 916, 862, 759, 658, 558 $cm^{-1}$.

### 4.10.2. (2*S*,3*R*)-4-Azido-3-methoxy-2-methylbutanoic acid (S-91)

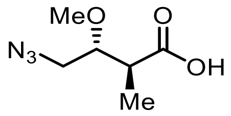

To a solution of ethyl (2*S*,3*R*)-4-azido-3-methoxy-2-methylbutanoate (**S-91a**) (1.06 g, 5.00 mmol, 1.00 equiv.) in DCE (0.2 M) was added $Me_3SnOH$ (4.52 g, 25.0 mmol, 5.00 equiv.) and the resulting mixture was heated to 80 °C for 72 h. Afterwards, the reaction was allowed to cool to room temperature and the solvents were removed under reduced pressure. The concentrated crude material was dissolved in EtOAc (50.0 mL) and was washed with 5% HCl (3 x 50.0 mL), water (1 x 50.0 mL) and brine (1 x 50.0 mL). The organic layer was dried over $Na_2SO_4$, the solids were filtered off and the solvents were removed *in vacuo* to yield carboxylic acid **S-91** amenable for use without further purification.

**$^1$H NMR** (400 MHz, $CDCl_3$): δ 3.58 (ddd, *J* = 7.2, 5.6, 3.4 Hz, 1H), 3.54 – 3.40 (m, 4H), 3.30 (dd, *J* = 13.2, 5.6 Hz, 1H), 2.89 – 2.81 (m, 1H), 1.18 (d, *J* = 7.2 Hz, 3H) ppm.

**HRMS (ESI$^+$):** calculated for $[M+Na]^+$ ($C_6H_{11}N_3O_3Na^+$) requires *m/z* 106.0693, found *m/z* 106.0690.

### 4.10.3. (2*S*,3*R*)-4-Azido-3-methoxy-2-methyl-N-((*E*)-4-(4,4,5,5-tetramethyl-1,3,2-dioxaborolan-2-yl)but-3-en-1-yl)butanamide (S-92a)

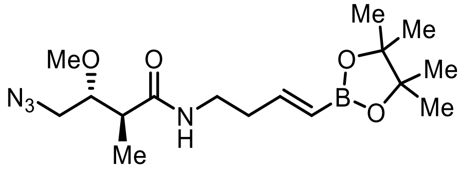

Synthesized according to **General Procedure I** on 1.00 mmol scale to yield **S-92a** (214 mg, 0.608 mmol, 61%) as a colourless foam.

**$^1$H-NMR** (600 MHz, $CDCl_3$): δ 6.56 (dt, *J* = 18.0, 6.6 Hz, 1H), 5.91 (s, 1H), 5.52 (d, *J* = 18.0 Hz, 1H), 3.53 (dd, *J* = 13.2, 3.2 Hz, 1H), 3.48 – 3.44 (m, 1H), 3.44 (s, 3H), 3.41 – 3.35 (m, 2H), 3.23 (dd, *J* = 13.2, 5.0 Hz, 1H), 2.43 (p, *J* = 7.1 Hz, 1H), 2.37 (qd, *J* = 6.6, 1.2 Hz, 2H), 1.26 (s, 12H), 1.11 (d, *J* = 7.1 Hz, 3H) ppm.

**$^{13}$C-NMR** (151 MHz, $CDCl_3$): δ 174.2, 150.4, 122.3 (*deduced from HSQC*), 83.4 (2C), 82.3, 58.9, 51.0, 44.1, 38.1, 35.7, 24.9 (4C), 14.4 ppm.

**HRMS (ESI$^+$):** calculated for $[M+Na]^+$ ($C_{16}H_{30}BN_4O_4Na^+$) required *m/z* 353.2355, found *m/z* 353.2355.

**FT-IR (neat) $\nu_{max}$:** 3308, 2978, 2934, 2097, 1639, 1543, 1445, 1357, 1319, 1267, 1233, 1142, 1101, 996, 969, 896, 848, 738, 640, 578 cm$^{-1}$.

**[a]** $_D^{20}$ = +14.4° (c = 0.85, $CHCl_3$).

### 4.10.4. (2*S*,3*R*)-4-Amino-3-methoxy-2-methyl-*N*-((*E*)-4-(4,4,5,5-tetramethyl-1,3,2-dioxaborolan-2-yl)but-3-en-1-yl)butanamide (S-92)

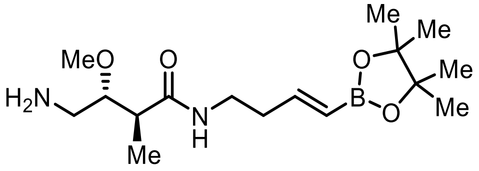


To a solution of azide **S-92a** (211 mg, 0.600 mmol, 1.00 equiv.) in THF/$H_2O$ (0.4 M, 7:1 v/v), a solution of $PEt_3$ (2.65 mL, 1.80 mmol, 3.00 equiv., 1 M in THF) was added dropwise. The reaction mixture was stirred for 15 h at room temperature. Then, the volatiles were removed under reduced pressure to quantitatively afford the desired primary amine **S-92** as a viscous colorless oil, which was used at the next step without any further purification.

**$^1$H NMR** (400 MHz, $CDCl_3$): δ 6.56 (dt, *J* = 17.9, 6.6 Hz, 1H), 6.02 (s, 1H), 5.51 (d, *J* = 18.0 Hz, 1H), 3.41 – 3.32 (m, 4H), 3.29 – 3.22 (m, 1H), 2.92 (dd, *J* = 13.5, 4.1 Hz, 1H), 2.72 (dd, *J* = 13.5, 5.1 Hz, 1H), 2.45 (p, *J* = 7.1 Hz, 1H), 2.36 (q, *J* = 6.6 Hz, 2H), 1.12 (d, *J* = 7.0 Hz, 3H) ppm.
**HRMS (ESI$^+$):** calculated for $[M+H]^+$ ($C_{16}H_{32}BN_2O_4^+$) requires *m/z* 327.2450, found *m/z* 327.2452.

### 4.10.5. (*R*,4*E*,6*E*)-3-Hydroxy-*N*-((2*R*,3*S*)-2-methoxy-3-methyl-4-oxo-4-(((*E*)-4-(4,4,5,5-tetramethyl-1,3,2-dioxaborolan-2-yl)but-3-en-1-yl)amino)butyl)-4-methyltetradeca-4,6-dienamide (S-93)

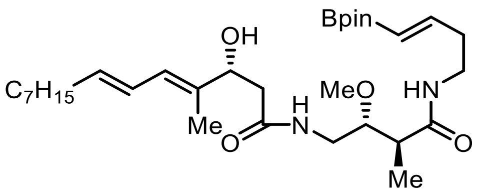


Synthesized according to **General Procedure L** on 0.500 mmol scale to yield **S-93** (226 mg, 0.402 mmol, 80%) as a pale yellow foam.
**$^1$H-NMR** (600 MHz, $CDCl_3$): δ 6.55 (dt, *J* = 18.0, 6.6 Hz, 1H), 6.48 – 6.41 (m, 1H), 6.21 (dd, *J* = 15.0, 10.9 Hz, 1H), 6.07 (d, *J* = 10.8 Hz, 1H), 6.03 (t, *J* = 5.4 Hz, 1H), 5.73 – 5.66 (m, 1H), 5.51 (d, *J* = 18.0 Hz, 1H), 4.43 (dd, *J* = 8.7, 2.8 Hz, 1H), 3.63 – 3.53 (m, 2H), 3.43 – 3.39 (m, 4H), 3.37 – 3.32 (m, 2H), 3.28 (dt, *J* = 14.0, 5.2 Hz,

1H), 2.45 – 2.33 (m, 5H), 2.09 (dd, *J* = 14.3, 7.1 Hz, 2H), 1.74 (s, 3H), 1.42 – 1.34 (m, 2H), 1.29 – 1.24 (m, 20H), 1.13 (d, *J* = 7.1 Hz, 3H), 0.87 (t, *J* = 7.0 Hz, 3H) ppm.

**$^{13}$C-NMR** (151 MHz, $CDCl_3$): δ 174.4, 172.3, 150.6, 136.0, 135.5, 125.8, 125.7, 121.9 (*deduced from HSQC*), 83.4 (2C), 81.1, 73.8, 58.8, 43.9, 41.7, 39.0, 38.0, 35.8, 33.1, 32.0, 29.5, 29.3, 29.3, 24.9 (4C), 22.8, 14.7, 14.2, 12.8 ppm.

**HRMS (ESI$^+$):** calculated for $[M+Na]^+$ ($C_{31}H_{55}BN_2O_6Na^+$) required *m/z* 585.4046, found *m/z* 585.4050.

**FT-IR (neat) $ν_{max}$:** 3304, 2926, 2855, 1640, 1545, 1456, 1359, 1321, 1269, 1235, 1144, 1099, 999, 968, 849, 759 cm$^{-1}$.

**[a]$_D^{20}$** = +27.1° (c = 1.00, $CHCl_3$).

### 4.10.6. (*R*,4*E*,6*E*)-1-(((2*R*,3*S*)-2-Methoxy-3-methyl-4-oxo-4-(((*E*)-4-(4,4,5,5-tetramethyl-1,3,2-dioxaborolan-2-yl)but-3-en-1-yl)amino)butyl)amino)-4-methyl-1-oxotetradeca-4,6-dien-3-yl (1*R*,4*R*)-4-chlorocyclobut-2-ene-1-carboxylate (S-93a)

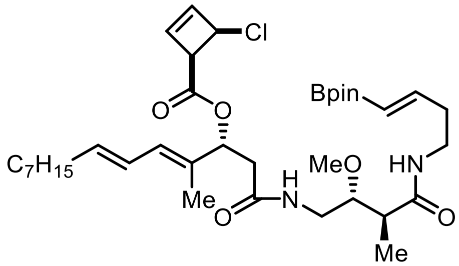


Synthesized according to **General Procedure M** on 0.183 mmol scale to yield **S-93a** (53.3 mg, 78.7 µmol, 43%, mixture of *cis*-diastereomers) as a colourless viscous oil.

**$^1$H-NMR** (600 MHz, $CDCl_3$): δ 6.55 (dt, *J* = 18.0, 6.6 Hz, 1H *for each isomer*), 6.29 – 6.22 (m, 2H *for each isomer*), 6.21 – 6.08 (m, 3H *for each isomer*), 6.05 (t, *J* = 5.5 Hz, 1H *for each isomer*), 5.75 – 5.68 (m, 1H *for each isomer*), 5.67 – 5.60 (m, 1H *for each isomer*), 5.51 (d, *J* = 18.0 Hz, 1H *for each isomer*), 5.10 (t, *J* = 4.1 Hz, 1H *for first isomer*), 5.08 (d, *J* = 4.3 Hz, 1H *for second isomer*), 4.07 (app dd, *J* = 7.6, 2.4 Hz, 1H *for each isomer*), 3.72 – 3.65 (m, 1H *for each isomer*), 3.41 – 3.33 (m, 6H *for each isomer*), 3.10 (ddd, *J* = 13.3, 10.8, 6.5 Hz, 1H *for each isomer*), 2.72 – 2.66 (m, 1H *for each isomer*), 2.53 (ddd, *J* = 14.6, 10.6, 5.5 Hz, 1H *for each isomer*), 2.33 (ddt, *J* = 21.0, 9.9, 6.8 Hz, 3H *for each isomer*), 2.08 (dd, *J* = 8.8, 4.1 Hz, 2H *for each isomer*), 1.77 (s, 3H *for each isomer*), 1.38 – 1.32 (m, 2H *for each isomer*), 1.30 – 1.24 (m, 20H *for each isomer*), 1.10 (app dd, *J* = 7.1, 1.2 Hz, 3H *for each isomer*), 0.87 (t, *J* = 7.0 Hz, 3H *for each isomer*) ppm.

**$^{13}$C-NMR** (151 MHz, $CDCl_3$): δ 174.6 (*for both isomers*), 169.4 (*for first isomer*), 169.3 (*for second isomer*), 168.9 (*for first isomer*), 168.8 (*for second isomer*), 150.4 (*for first isomer*), 150.4 (*for second isomer*), 140.6 (*for first isomer*), 140.5 (*for second isomer*), 137.3 (*for first isomer*), 137.3 (*for second isomer*), 136.6 (*for both isomers*), 131.1 (*for first isomer*), 130.8 (*for second isomer*), 129.0 (*for first isomer*), 128.6 (*for second isomer*), 125.4 (*for both isomers*), 121.7 (*for both isomers, deduced with HSQC*), 83.4 (*for both isomers*), 81.1 (*for first isomer*), 81.0 (*for second isomer*), 77.0 (*for first isomer*), 76.9 (*for second isomer*), 58.6 (*for both isomers*), 56.5 (*for first isomer*), 56.4 (*for second isomer*), 54.0 (*for first isomer*), 54.0 (*for second isomer*), 43.7 (*for first isomer*), 43.6 (*for second isomer*), 40.9 (*for both isomers*), 38.8 (*for first isomer*), 38.7 (*for second isomer*), 38.1 (*for first isomer*), 38.0 (*for second isomer*), 35.8 (*for both isomers*), 33.2 (*for both isomers*), 31.9 (*for both isomers*), 29.4 (1C *for first isomer*), 29.4 (1C *for second isomer*), 29.3 (1C *for each isomer*), 24.9 (*for first isomer*), 24.9 (*for second isomer*), 22.8 (1C *for each isomer*), 14.8 (*for first isomer*), 14.8 (*for second isomer*), 14.2 (*for both isomers*), 12.8 (*for first isomer*), 12.8 (*for second isomer*) ppm.
**HRMS (ESI$^+$):** calculated for $[M+Na]^+$ ($C_{36}H_{58}BClN_2O_7Na^+$) required *m/z* 699.3918, found *m/z* 699.3922.
**FT-IR (neat) $\nu_{max}$:** 2956, 2926, 2854, 1719, 1652, 1622, 1556, 1546, 1460, 1438, 1368, 1289, 1228, 1176, 1170, 1106, 1071, 1036, 991, 966, 920 $cm^{-1}$.

### 4.10.7. Fs-FR10

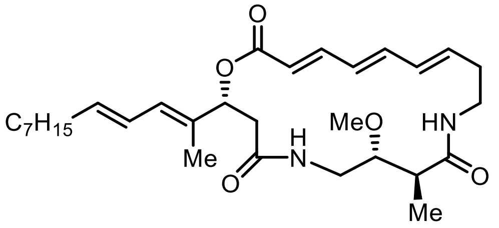


Synthesized according to **General Procedure N** on 30.0 µmol scale to yield **Fs-FR10** (11.0 mg, 17.1 µmol, 57%) as a colourless foam.
**$^1$H NMR** (600 MHz, $CDCl_3$): δ 7.26 (s, 1H), 6.49 (dd, *J* = 14.9, 10.8 Hz, 1H), 6.26 – 6.10 (m, 4H), 5.86 (ddd, *J* = 15.4, 11.1, 4.6 Hz, 1H), 5.78 – 5.71 (m, 3H), 5.66 (br. s, 1H), 5.61 (dd, *J* = 10.8, 2.9 Hz, 1H), 3.86 – 3.78 (m, 1H), 3.48 (ddd, *J* = 9.2, 5.2, 4.0 Hz, 1H), 3.37 – 3.31 (m, 4H), 3.18 – 3.11 (m, 1H), 2.92 (dt, *J* = 14.0, 4,5 Hz, 1H), 2.61 (dt, *J* = 13.8, 5.2 Hz, 2H), 2.53 – 2.43 (m, 2H), 2.33 – 2.22 (m, 1H), 2.13 – 2.06 (m, 2H), 1.80 (s, 3H), 1.41 – 1.35 (m, 2H), 1.31 – 1.26 (m, 8H), 1.07 (d, *J* = 7.5 Hz, 3H), 0.88 (t, *J* = 7.1 Hz, 3H) ppm.
**$^{13}$C NMR** (151 MHz, $CDCl_3$): δ 173.6, 169.8, 166.0, 146.3, 142.6, 138.2, 136.7, 132.0, 127.8, 126.6, 125.6, 119.8, 80.5, 76.7, 56.7, 42.2, 42.1, 40.5, 37.7, 33.6, 33.2, 32.0, 29.5, 29.3, 29.3, 22.8, 14.3, 13.5, 12.3 ppm.

**IR (neat) $\nu_{max}$:** 2956, 2924, 2853, 1740, 1702, 1646, 1643, 1614, 1556, 1541, 1527, 1464, 1436, 1377, 1350, 1294, 1271, 1259, 1235, 1172, 1101, 1031, 1008, 967, 911 $cm^{-1}$.

**HRMS ($ESI^+$):** calculated for $[M+Na]^+$ ($C_{30}H_{46}N_2O_5Na^+$) requires *m/z* 537.3299, found *m/z* 537.3284.

**[a]**$_D^{20}$ = –99° (c = 2.1, $CHCl_3$)

## 4.11. Fs-FR12

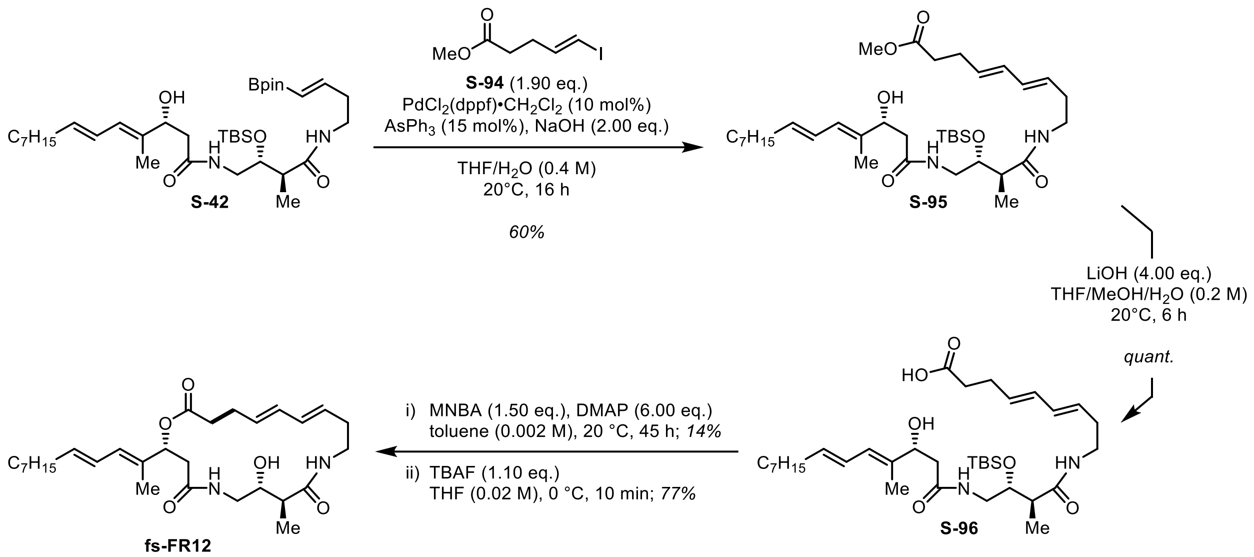

### 4.11.1. Methyl (*E*)-5-iodopent-4-enoate (S-94)

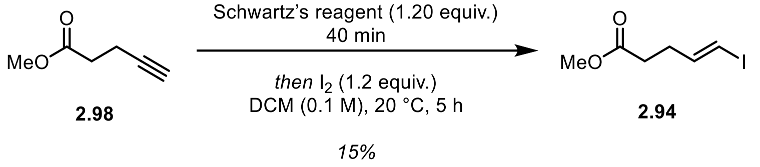

<u>*Representative procedure*</u>: To a flame-dried Schlenk flask were added alkyne **S-98** (1.36 g, 12.1 mmol, 1.00 equiv.) and $CH_2Cl_2$ (0.2 M). The solution was cooled to 0 °C before Schwartz's reagent (zirconocene chloride hydride, 3.75 g, 14.5 mmol, 1.20 equiv.) was added under argon flow. The reaction mixture (white suspension) was stirred under argon for 40 min before turning to intense yellow. Then, a solution of iodine (3.69 g, 14.5 mmol, 1.20 equiv.) in $CH_2Cl_2$ (0.1 M) was added dropwise with an addition funnel at room temperature over the course of 15 min and under argon atmosphere (balloon). After 2.5 h of stirring at room temperature, the brown reaction mixture was transferred to a separatory funnel containing a sat. aq. $Na_2S_2O_3$ solution. The aqueous and organic layers were partitioned, and the aqueous layer was subsequently extracted with $Et_2O$ (twice). The combined organic layers were washed with brine, dried over anhydrous magnesium sulfate, filtered, and concentrated under reduced pressure. The crude product was purified by column chromatography (silica gel, 10% to 25% EtOAc in heptanes) to afford the desired vinyl iodide **S-94** (447 mg, 1.86 mmol, 15.4%) as an orange oil.

**$^{1}$H NMR** (600 MHz, $CDCl_3$): δ 6.52 (dt, *J* = 13.6, 6.7 Hz, 1H), 6.11 (d, *J* = 14.4 Hz, 1H), 3.68 (s, 3H), 2.44 – 2.34 (m, 4H) ppm.

**$^{13}$C NMR** (151 MHz, $CDCl_3$): δ 172.9, 144.3, 76.4, 51.9, 32.8, 31.3 ppm.

**IR (neat) $\nu_{max}$:** 1732, 1435, 1364, 1294, 1257, 1237, 1195, 1171, 1141, 1085, 1063, 1028, 993, 942, 884, 834, 795 $cm^{-1}$.

**HRMS (ESI$^{+}$):** calculated for $[M+Na]^{+}$ ($C_6H_9IO_2Na^{+}$) requires *m/z* 262.9540, found *m/z* 262.9540.

### 4.11.2. Methyl (4*E*,6*E*)-9-((2*S*,3*R*)-3-((*tert*-butyldimethylsilyl)oxy)-4-((*R*,4*E*,6*E*)-3-hydroxy-4-methyltetradeca-4,6-dienamido)-2-methylbutanamido)nona-4,6-dienoate (S-95)

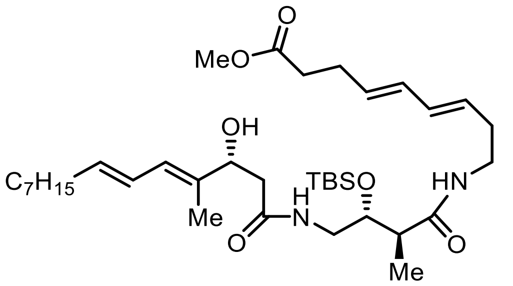


To a solution of boronic acid pinacol ester **10** (441 mg, 0.665 mmol, 1.00 equiv.) and vinyl iodide **S-94** (303 mg, 1.26 mmol, 1.90 equiv.)[6] in THF (0.4 M), $PdCl_2$(dppf)•$CH_2Cl_2$ (55.3 mg, 66.4 µmol, 10 mol%), triphenylarsine (30.5 mg, 99.6 µmol,15 mol%) and a 3 M aqueous solution of sodium hydroxide (0.44 ml, 1.33 mmol, 2.00 equiv.) were added at room temperature. After stirring for 16 h, the reaction was stopped by addition of a saturated aqueous solution of ammonium chloride. The aqueous layer was extracted with EtOAc and the combined organic layers were washed with brine, dried over anhydrous magnesium sulfate, filtered, and concentrated under reduced pressure. Purification by column chromatography (silica gel, 35% EtOAc in heptanes; 5% to 15% isopropanol in heptanes) delivered the desired product **S-95** (260 mg, 0.339 mmol, 60%) as a colorless viscous oil.

**$^{1}$H NMR** (600 MHz, $CDCl_3$): δ 6.39 (s, 1H), 6.22 (dd, *J* = 15.0, 11.0 Hz, 2H), 6.04 (ddd, *J* = 19.6, 17.4, 10.6 Hz, 3H), 5.73 – 5.67 (m, 1H), 5.62 – 5.55 (m, 1H), 5.54 – 5.47 (m, 1H), 4.43 (dd, *J* = 8.9, 3.0 Hz, 1H), 3.90 – 3.85 (m, 1H), 3.72 – 3.68 (m, 1H), 3.67 (s, 3H), 3.38 (td, *J* = 13.3, 6.7 Hz, 1H), 3.23 (dt, *J* = 19.9, 6.6 Hz, 1H), 2.97 – 2.91 (m, 1H), 2.47 – 2.36 (m, 8H), 2.28 – 2.24 (m, 2H), 2.10 (app q, *J* = 7.2 Hz, 2H), 1.74 (s, 3H), 1.39 – 1.35 (m, 2H), 1.31 – 1.24 (m, 8H), 1.14 (d, *J* = 7.2 Hz, 3H), 0.91 (s, 9H), 0.88 (t, *J* = 7.0 Hz, 3H), 0.16 (s, 3H), 0.09 (s, 3H) ppm.

**$^{13}$C NMR** (151 MHz, $CDCl_3$): δ 174.6, 173.6, 172.3, 136.1, 135.5, 132.7, 131.1, 131.1, 129.1, 125.8, 125.7, 73.8, 72.1, 51.8, 44.8, 43.0, 41.7, 38.8, 33.8, 33.2, 32.9, 32.0, 29.6, 29.3, 29.3, 27.9, 26.0 (3C), 22.8, 18.1, 15.5, 14.2, 12.8, −4.4, −4.8 ppm.

**IR (neat) $ν_{max}$:** 2957, 2926, 2854, 1740, 1713, 1649, 1221, 989, 754 $cm^{-1}$.

**HRMS (ESI$^+$):** calculated for $[M+Na]^+$ ($C_{36}H_{64}N_2O_6SiNa^+$) requires *m/z* 671.4426, found *m/z* 671.4401.

**$[a]_D^{20}$** = +2.6°(c = 1.00, $CHCl_3$).

### 4.11.3. (4*E*,6*E*)-9-((2*S*,3*R*)-3-((*tert*-Butyldimethylsilyl)oxy)-4-((*R*,4*E*,6*E*)-3-hydroxy-4-methyltetradeca-4,6-dienamido)-2-methylbutanamido)nona-4,6-dienoic acid (S-96)

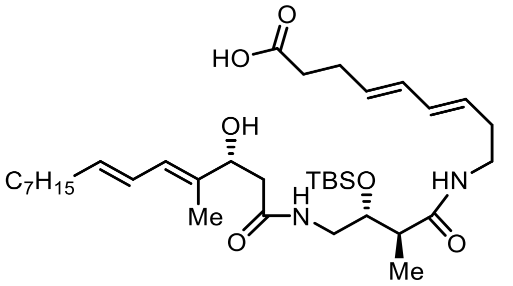

Synthesized according to **General Procedure H** on 0.103 mmol scale to yield **S-96** which was used in the next step without further purification.

### 4.11.4. (2*R*,7*R*,8*S*,13*E*,15*E*)-7-((*tert*-Butyldimethylsilyl)oxy)-2-((2*E*,4*E*)-dodeca-2,4-dien-2-yl)-8-methyl-1-oxa-5,10-diazacyclononadeca-13,15-diene-4,9,19-trione (S-97)

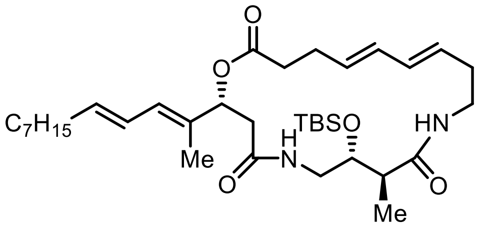

To a flame-dried Schlenk tube equipped with molecular sieves 4 Å, DMAP (113 mg, 0.924 mmol, 6.00 equiv.) and MNBA (81.2 mg, 0.231 mmol, 1.50 equiv.) were added under argon flow. Dry toluene (0.002 M) was then added, followed by the carboxylic acid **S-96** (97.8 mg, 0.154 mmol, 1.00 equiv.) dissolved in dry toluene via a syringe, and the mixture was stirred at ambient temperature for 45 h. Then, the reaction mixture was diluted with EtOAc and filtered to remove the molecular sieves. The organic phase was washed twice with a saturated aqueous solution of sodium bicarbonate and brine, dried over

anhydrous magnesium sulfate, filtered, and the solvents were evaporated under reduced pressure. The product was purified by column chromatography (silica gel, 10% → 15% → 60% EtOAc in heptanes) to yield **S-97** (13.7 mg, 22.2 mmol, 14%) as a colorless oil.

**$^1$H NMR** (600 MHz, $CDCl_3$): δ 6.90 – 6.84 (m, 1H), 6.17 (dd, *J* = 15.0, 10.9 Hz, 1H), 6.09 (dd, *J* = 15.2, 10.4 Hz, 1H), 6.03 (d, *J* = 10.9 Hz, 1H), 5.98 (dd, *J* = 14.8, 10.5 Hz, 1H), 5.92 – 5.86 (m, 1H), 5.77 – 5.70 (m, 1H), 5.61 – 5.54 (m, 3H), 3.91 – 3.81 (m, 2H), 3.44 – 3.38 (m, 1H), 3.11 – 3.05 (m, 1H), 2.96 – 2.89 (m, 1H), 2.58 – 2.39 (m, 7H), 2.32 – 2.24 (m, 1H), 2.09 (dd, *J* = 14.1, 6.7 Hz, 2H), 1.72 (s, 3H), 1.41 – 1.35 (m, 2H), 1.32 – 1.23 (m, 8H), 1.19 (d, *J* = 7.3 Hz, 3H), 0.95 (s, 9H), 0.89 – 0.84 (m, 3H), 0.21 (s, 3H), 0.12 (s, 3H) ppm.

**$^{13}$C NMR** (151 MHz, $CDCl_3$): δ 174.7, 171.9, 170.2, 137.4, 132.7, 131.7, 131.7, 131.0, 130.5, 127.7, 125.4, 75.6, 72.6, 44.6, 44.4, 41.8, 37.8, 34.4, 33.7, 33.2, 32.0, 29.4, 29.3, 29.3, 26.7, 26.0 (3C), 22.8, 18.1, 16.6, 14.2, 12.9, −4.2, −4.6 ppm.

**IR (neat) $\nu_{max}$:** 3306, 2925, 2854, 1738, 1649, 1549, 1435, 1362, 1253, 1188, 1138, 1093, 1029, 988, 964, 909, 837, 777, 736, 702 $cm^{-1}$.

**HRMS (ESI$^+$):** calculated for $[M+Na]^+$ ($C_{35}H_{60}N_2O_5SiNa^+$) requires *m/z* 639.4164, found *m/z* 639.4150.

**[a]$_D^{20}$** = +69.7°(c = 1.00, $CHCl_3$).

### 4.11.5. Fs-FR12

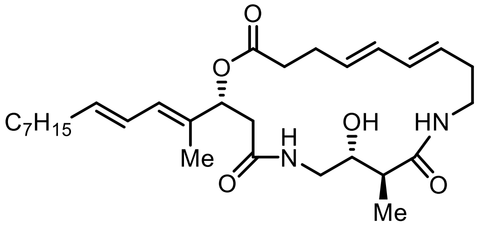


Synthesized according to **General Procedure O** on 17.8 µmol scale to yield **Fs-FR12** (6.90 mg, 13.7 µmol, 77%) as a white solid.

**$^1$H-NMR** (600 MHz, $CDCl_3$): δ 7.29 – 7.22 (m, 1H), 6.39 – 6.30 (m, 1H), 6.25 – 6.13 (m, 2H), 6.12 – 6.03 (m, 2H), 5.99 (dd, *J* = 15.0, 10.4 Hz, 1H), 5.78 – 5.70 (m, 1H), 5.63 (dd, *J* = 11.2, 1.8 Hz, 1H), 5.54 (ddd, *J* = 22.6, 14.6, 7.0 Hz, 2H), 4.31 (s, 1H), 3.61 – 3.50 (m, 2H), 3.48 – 3.40 (m, 1H), 3.28 – 3.20 (m, 1H), 3.03 – 2.94 (m, 1H), 2.64 (dd, *J* = 15.4, 11.4 Hz, 1H), 2.52 (dd, *J* = 15.4, 2.3 Hz, 1H), 2.43 (dddd, *J* = 32.2, 29.6, 16.8, 9.1 Hz, 5H), 2.31 – 2.20 (m, 3H), 2.10 (dd, *J* = 14.2, 7.1 Hz, 2H), 1.73 (s, 3H), 1.40 – 1.36 (m, 2H), 1.32 – 1.24 (m, 11H), 0.88 (t, *J* = 7.0 Hz, 3H) ppm.

**$^{13}$C-NMR** (151 MHz, $CDCl_3$) δ 175.2, 172.3, 170.6, 137.5, 133.0, 131.5, 131.3, 130.8, 129.7, 128.1, 125.3, 74.9, 74.2, 45.8, 44.4, 41.2, 38.3, 33.9, 33.2, 33.1, 32.0, 29.4, 29.4, 29.3, 27.2, 22.8, 15.9, 14.2, 12.8 ppm.

**HRMS (ESI$^+$):** calculated for $[M+Na]^+$ ($C_{29}H_{46}N_2O_5Na^+$) required *m/z* 525.3299, found *m/z* 525,3306.

**FT-IR (neat) $\nu_{max}$:** 3301, 2955, 2922, 2853, 1731, 1639, 1550, 1254,1243, 989 $cm^{-1}$.

**[a]$_D^{25}$** = 19.6° (c = 0.031, $CHCl_3$).

## 4.12. Fs-FR13

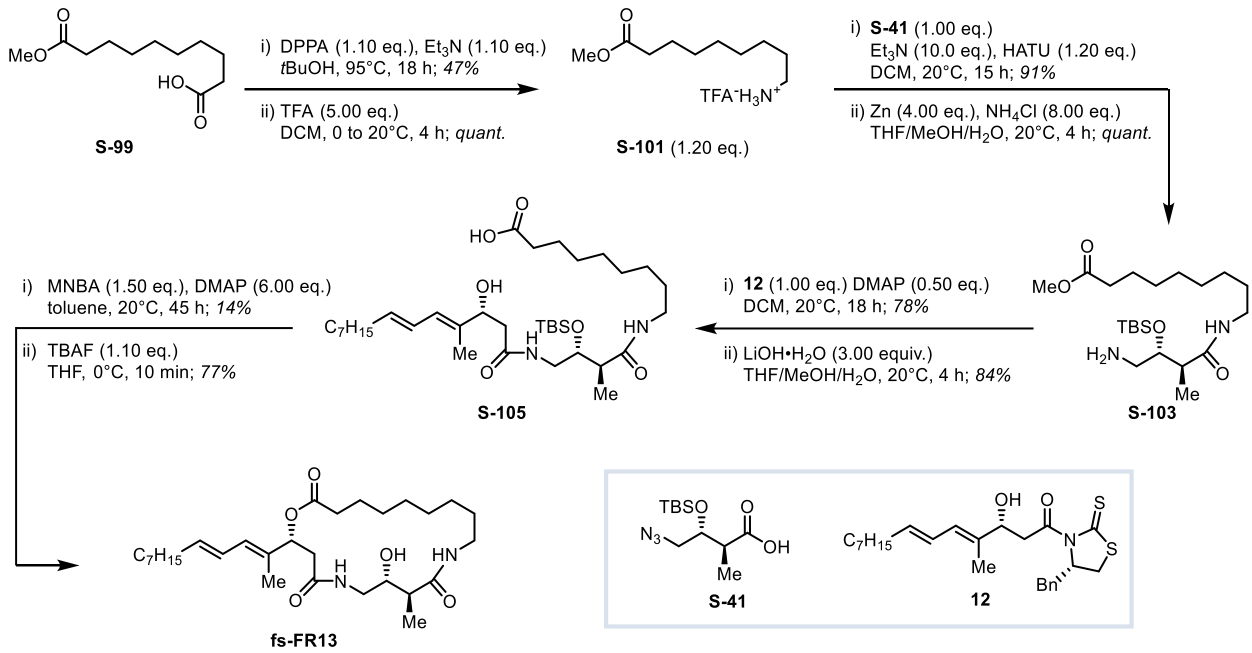


## 4.12.1. Methyl 9-((tert-butoxycarbonyl)amino)nonanoate (S-100)

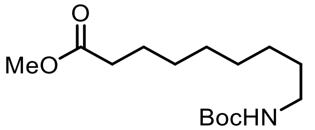


Synthesized according to **General Procedure B** on 16.1 mmol scale to yield **S-100**(2.18 g, 7.58 mmol, 47%) as a colorless oil.

**$^{1}$H NMR** (700 MHz, $CDCl_3$): δ 4.49 (s, 1H), 3.66 (s, 3H), 3.10 (m, 2H), 2.29 (t, *J* = 7.5 Hz, 2H), 1.63 – 1.59 (m, 2H), 1.44 (s, 10H), 1.29 (s, 9H) ppm.

**$^{13}$C NMR** (176 MHz, $CDCl_3$): δ 174.4, 156.1, 51.6, 40.7, 34.2, 30.2, 29.9, 29.3, 29.22, 29.18, 26.86, 28.58 (3C), 25.0 ppm.

**IR (neat) $\nu_{max}$:** 3391, 3369, 2976, 2929, 2856, 1736, 1702, 1517, 1456, 1438, 1391, 1365, 1266, 1248, 1171, 1110, 1042, 1011, 909, 869, 779, 732, 648 $cm^{-1}$.

**HRMS (ESI$^{+}$):** calculated for $[M+Na]^{+}$ ($C_{15}H_{29}NO_4Na^{+}$) requires *m/z* 310.1989, found *m/z* 310.1994.

### 4.12.2. 9-Methoxy-9-oxononan-1-aminium 2,2,2-trifluoroacetate (S-101)

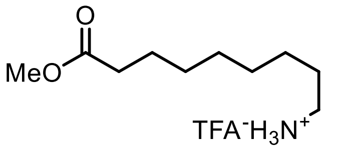


Synthesized according to **General Procedure D** on 7.14 mmol scale to yield **S-101** as a colorless oil which was used without further purification.

**$^1$H NMR** (400 MHz, $CDCl_3$): δ 3.68 (s, 3H), 3.13 – 3.03 (m, 2H), 2.33 (t, *J* = 7.4 Hz, 2H), 1.74 – 1.66 (m, 2H), 1.65 – 1.56 (m, 2H), 1.40 – 1.28 (m, 8H) ppm.

### 4.12.3. Methyl 9-((2*S*,3*R*)-4-azido-3-((*tert*-butyldimethylsilyl)oxy)-2-methylbutan-amido)nonanoate (S-102)

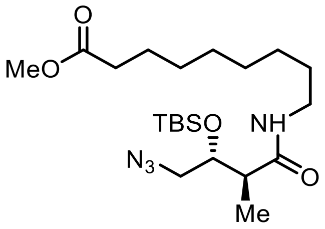


Synthesized according to **General Procedure I** on 7.14 mmol scale to yield **S-102** (2.88 g, 6.50 mmol, 91%) as a colorless oil.

**$^1$H-NMR** (600 MHz, $CDCl_3$): δ 5.96 (s, 1H), 3.91 (dd, *J* = 10.1, 4.6 Hz, 1H), 3.67 (s, 3H), 3.46 (dd, *J* = 12.9, 4.0 Hz, 1H), 3.32 (td, *J* = 13.4, 7.2 Hz, 1H), 3.24 (dd, *J* = 12.9, 4.8 Hz, 1H), 3.11 (qd, *J* = 7.1, 5.2 Hz, 1H), 2.49 – 2.42 (m, 1H), 2.30 (t, *J* = 7.5 Hz, 2H), 1.61 (dd, *J* = 14.6, 7.3 Hz, 2H), 1.52 – 1.44 (m, 2H), 1.30 (br s, 8H), 1.14 (d, *J* = 7.1 Hz, 3H), 0.91 (s, 9H), 0.14 (s, 3H), 0.09 (s, 3H) ppm.

**$^{13}$C-NMR** (151 MHz, $CDCl_3$): δ 174.43, 174.00, 73.7, 54.9, 51.6, 45.6, 39.5, 34.2, 29.7, 29.3, 29.2, 29.18, 27.1, 26.0 (3C), 25.0, 15.3, −4.3, −4.9 ppm.

**HRMS (ESI$^+$):** calculated for $[M+Na]^+$ ($C_{21}H_{42}N_4O_4SiNa^+$) requires *m/z* 465.2868, found *m/z* 465.2866.

**FT-IR (neat) $\nu_{max}$:** 2954, 2929, 2857, 2101, 1741, 1723, 1646, 1552, 1545, 1469, 1438, 1375, 1361, 1320, 1283, 1255, 1216, 1173, 1102, 1059, 1007, 974, 839, 827, 809, 779, 754 cm$^{-1}$.

**$[a]_D^{20}$** = +7.6° (c = 1.00, $CHCl_3$).

### 4.12.4. Methyl 9-((2S,3R)-4-amino-3-((tert-butyldimethylsilyl)oxy)-2-methylbutan-amido)nonanoate (S-103)

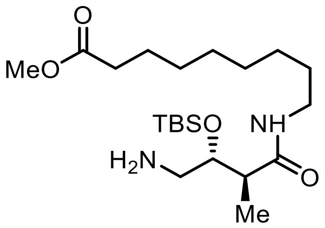


Synthesized according to **General Procedure J** on 6.50 mmol scale to yield **S-103** as a colorless oil which was used in the next step without further purification.

### 4.12.5. Methyl 9-((2*S*,3R)-3-((*tert*-butyldimethylsilyl)oxy)-4-((*R*,4*E*,6*E*)-3-hydroxy-4-methyl-tetradeca-4,6-dienamido)-2-methylbutanamido)nonanoate (S-104)

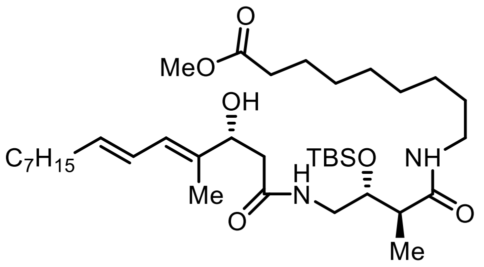


Synthesized according to **General Procedure L** on 1.10 mmol scale to yield **S-104** (560 mg, 0.857 mmol, 78%) as a yellow oil.

**$^1$H-NMR** (500 MHz, $CDCl_3$): δ 6.39 – 6.32 (m, 1H), 6.22 (dd, *J* = 14.9, 10.9 Hz, 1H), 6.08 (d, *J* = 11.4 Hz, 2H), 5.76 – 5.66 (m, 1H), 4.43 (d, *J* = 8.5 Hz, 1H), 3.88 (dt, *J* = 8.3, 4.4 Hz, 1H), 3.78 – 3.71 (m, 1H), 3.66 (s, 3H), 3.41 (d, *J* = 2.3 Hz, 1H), 3.34 – 3.26 (m, 1H), 3.17 – 3.10 (m, 1H), 2.96 – 2.88 (m, 1H), 2.46 – 2.38 (m, 3H), 2.30 (t, *J* = 7.5 Hz, 2H), 2.10 (dd, *J* = 14.4, 7.1 Hz, 2H), 1.74 (s, 3H), 1.62 (d, *J* = 6.9 Hz, 2H), 1.53 – 1.44 (m, 2H), 1.42 – 1.36 (m, 2H), 1.35 – 1.24 (m, 16H), 1.16 (d, *J* = 7.1 Hz, 3H), 0.93 – 0.85 (d, *J* = 7.6 Hz, 12H), 0.17 (s, 3H), 0.11 (s, 3H) ppm.

**$^{13}$C-NMR** (125 MHz, $CDCl_3$): δ 174.4 (2C, *deduced from HMBC*), 172.3, 136.1, 135.5, 125.8, 125.8, 73.8, 72.1, 51.6, 44.9, 43.1, 41.7, 39.5, 34.2, 33.2, 32.0, 29.7, 29.6, 29.3, 29.3, 29.3, 29.2 (2C), 27.1, 26.0 (3C), 25.0, 22.8, 18.1, 15.4, 14.2, 12.8, −4.4, −4.8 ppm.

**HRMS (ESI$^+$):** calculated for $[M+Na]^+$ ($C_{36}H_{68}N_2O_6SiNa^+$) requires *m/z* 675.4739, found *m/z* 675.4774.

**FT-IR (neat) $\nu_{max}$:** 3308, 2926, 2855, 1740, 1645, 1540, 1462, 1436, 1362, 1319, 1251, 1198, 1171, 1142, 1100, 1025, 1005, 965, 940, 835, 777, 754, 723, 702 $cm^{-1}$.

**[a]**$_D^{20}$ = +27.8° (c = 1.00, $CHCl_3$).

### 4.12.6. 9-((2*S*,3*R*)-3-((*tert*-Butyldimethylsilyl)oxy)-4-((*R*,4*E*,6*E*)-3-hydroxy-4-methyltetradeca-4,6-dienamido)-2-methylbutanamido)nonanoic acid (S-105)

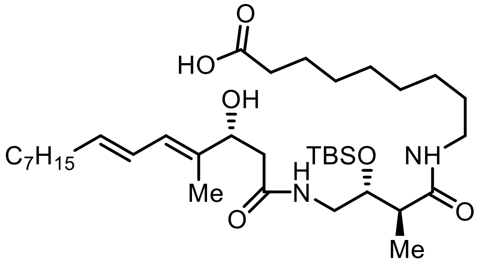


Synthesized according to **General Procedure H** on 0.855 mmol scale to yield **S-105** (459 mg, 0.718 mmol, 84%) as an orange oil which was used without further purification.

**$^1$H-NMR** (600 MHz, $CDCl_3$): δ 6.73 (s, 1H), 6.39 (s, 1H), 6.28 – 6.18 (m, 1H), 6.08 (d*, J* = 10.7 Hz, 1H), 5.76 – 5.66 (m, 1H), 4.44 (d*, J* = 8.9 Hz, 1H), 3.91 – 3.83 (m, 1H), 3.80 – 3.71 (m, 1H), 3.43 (dd*, J* = 13.1, 6.7 Hz, 1H), 3.30 – 3.20 (m, 1H), 3.11 – 3.01 (m, 1H), 2.92 – 2.83 (m, 1H), 2.54 – 2.49 (m, 1H), 2.45 – 2.40 (m, 1H), 2.39 – 2.30 (m, 3H), 2.14 – 2.06 (m, 2H), 1.74 (s, 3H), 1.63 (s, 2H), 1.48 (d*, J* = 5.9 Hz, 2H), 1.37 (d*, J* = 6.5 Hz, 2H), 1.31 (d*, J* = 9.8 Hz, 16H), 1.18 (d*, J* = 7.0 Hz, 3H), 0.92 (s, 9H), 0.88 (t*, J* = 6.7 Hz, 3H), 0.19 (s, 3H), 0.12 (s, 3H) ppm.

**HRMS (ESI$^+$):** calculated for $[M+Na]^+$ ($C_{35}H_{66}N_2O_6SiNa^+$) requires *m/z* 661.4582, found *m/z* 661.4548.

### 4.12.7. (2*R*,7*R*,8*S*)-7-((*tert*-Butyldimethylsilyl)oxy)-2-((2*E*,4*E*)-dodeca-2,4-dien-2-yl)-8-methyl-1-oxa-5,10-diazacyclononadecane-4,9,19-trione (S-106)

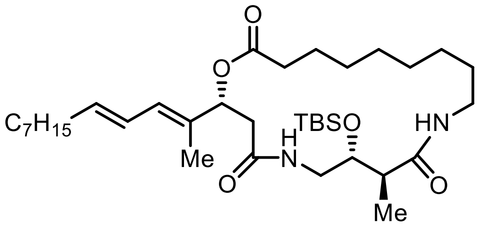


To a flame-dried Schlenk tube equipped with molecular sieves 4 Å, DMAP (147 mg, 1.20 mmol, 6.00 equiv.) and MNBA (103 mg, 0.300 mmol, 1.50 equiv.) were added under argon flow. Dry toluene (0.002 M) was then added, followed by the carboxylic acid **S-105** (128 mg, 0.200 mmol, 1.00 equiv.) dissolved in dry toluene via a syringe, and the mixture was stirred at ambient temperature for 72 h. Then, the reaction mixture was diluted with EtOAc and filtered to remove the molecular sieves. The organic phase was washed

twice with a saturated aqueous solution of sodium bicarbonate and brine, dried over anhydrous magnesium sulfate, filtered, and the solvents were evaporated under reduced pressure. The product was purified by column chromatography (silica gel, 10% → 15% → 60% EtOAc in heptanes) to yield **S-106** (43.6 mg, 70.2 µmol, 35%) as a colorless oil.

**$^1$H NMR** (700 MHz, $CDCl_3$): δ 6.32 (d, *J* = 5.7 Hz, 1H), 6.27 (dd, *J* = 7.4, 4.4 Hz, 1H), 6.17 (dd, *J* = 15.0, 10.8 Hz, 1H), 6.05 (d, *J* = 10.8 Hz, 1H), 5.76 – 5.69 (m, 1H), 5.52 (dd, *J* = 10.7, 2.0 Hz, 1H), 3.85 – 3.81 (m, 1H), 3.81 – 3.77 (m, 1H), 3.77 – 3.71 (m, 1H), 2.94 – 2.84 (m, 2H), 2.57 (dd, *J* = 14.2, 10.8 Hz, 1H), 2.53 – 2.49 (m, 1H), 2.43 (dd, *J* = 14.3, 2.4 Hz, 1H), 2.39 – 2.32 (m, 1H), 2.32 – 2.27 (m, 1H), 2.09 (q, *J* = 7.1 Hz, 2H), 1.73 (s, 3H), 1.69 – 1.63 (m, 2H), 1.62 – 1.55 (m, 2H), 1.50 – 1.44 (m, 2H), 1.42 – 1.36 (m, 4H), 1.34 – 1.31 (m, 2H), 1.30 – 1.25 (m, 10H), 1.16 (d, *J* = 7.2 Hz, 3H), 0.93 (s, 9H), 0.89 – 0.86 (m, 3H), 0.17 (s, 3H), 0.11 (s, 3H) ppm.

**$^{13}$C NMR** (176 MHz, $CDCl_3$): δ 174.5, 172.9, 170.0, 137.2, 131.8, 127.6, 125.4, 75.9, 72.3, 44.9, 43.3, 41.9, 37.9, 34.4, 33.2, 32.0, 29.4, 29.3, 29.3, 28.9, 28.4, 27.8, 27.3, 26.0 (3C), 25.3, 24.6, 22.8, 18.1, 15.2, 14.2, 12.8, −4.3, −4.8 ppm.

**IR (neat) $\nu_{max}$:** 3293, 2927, 2856, 1732, 1649, 1542, 1461, 1442, 1362, 1321, 1251, 1188, 1143, 1123, 1098, 1050, 1026, 1005, 964, 836 $cm^{-1}$.

**HRMS (ESI$^+$):** calculated for $[M+Na]^+$ ($C_{35}H_{64}N_2O_5SiNa^+$) requires *m/z* 643.4477, found *m/z* 643.4464.

**$[a]_D^{20}$** = +25.6°(c = 1.00, $CHCl_3$).

### 4.12.8. Fs-FR13

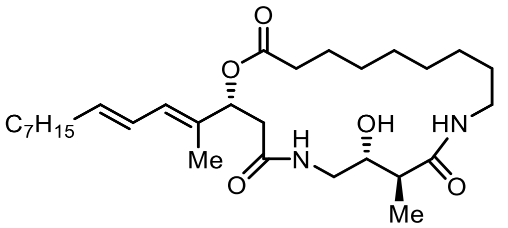


Synthesized according to **General Procedure O** on 19.3 µmol scale to yield **Fs-FR13** (7.50 mg, 14.8 µmol, 77%) as a white solid.

**$^1$H-NMR** (600 MHz, $CDCl_3$): δ 6.43 (s, 1H), 6.24 – 6.15 (m, 2H), 6.07 (d, *J* = 10.8 Hz, 1H), 5.78 – 5.70 (m, 1H), 5.61 (dd, *J* = 11.1, 2.4 Hz, 1H), 4.57 (s, 1H), 3.68 – 3.61 (m, 1H), 3.51 (ddd, *J* = 13.9, 7.3, 3.8 Hz, 1H), 3.42 – 3.34 (m, 1H), 3.32 – 3.25 (m, 1H), 3.21 – 3.14 (m, 1H), 2.63 (dd, *J* = 14.8, 11.2 Hz, 1H), 2.51 (dd,

$J$ = 14.8, 2.6 Hz, 1H), 2.36 – 2.28 (m, 3H), 2.10 (q, $J$ = 7.1 Hz, 2H), 1.74 (s, 3H), 1.69 – 1.60 (m, 2H), 1.57 – 1.48 (m, 4H), 1.42 – 1.23 (m, 19H), 0.88 (t, $J$ = 7.0 Hz, 3H) ppm.

**$^{13}$C-NMR** (151 MHz, $CDCl_3$): δ 175.3, 173.2, 171.0, 137.4, 131.5, 127.9, 125.4, 75.1, 74.1, 45.4, 44.0, 41.3, 38.7, 34.2, 33.2, 32.0, 29.4, 29.4, 29.3, 29.1, 28.9, 28.6, 28.0, 25.7, 24.7, 22.8, 15.7, 14.2, 12.8 ppm.

**HRMS (ESI$^+$):** calculated for $[M+Na]^+$ ($C_{29}H_{50}N_2O_5Na^+$) requires *m/z* 529.3612, found *m/z* 529.3612.

**FT-IR (neat) $\nu_{max}$:** 3294, 3092, 2924, 2854, 1734, 1637, 1547, 1457, 1438, 1374, 1294, 1258, 1217, 1196, 1175, 1153, 1106, 1057, 1029, 754, 723 cm$^{-1}$.

**$[a]_D^{20}$** = +18.2° (c = 0.28, $CHCl_3$).

## 4.13. Fs-FR14

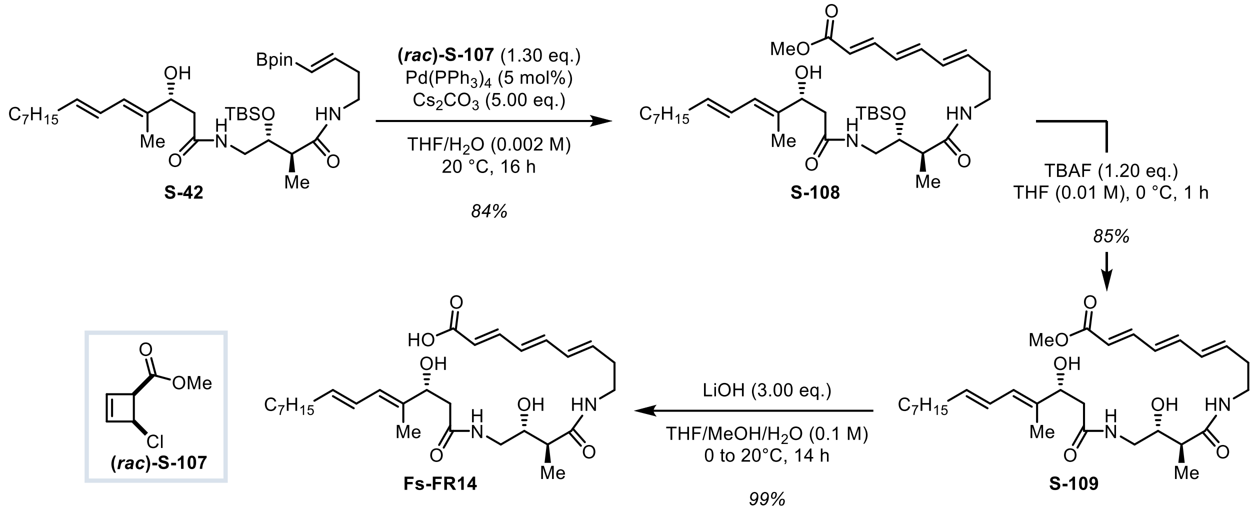


### 4.13.1. (±)-*cis*-Methyl-4-chlorocyclobut-2-ene-1-carboxylate ((*rac*)-S-107)

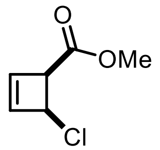

To a flame dried round bottom flask under argon was added (±)-*cis*-4-chlorocyclobut-2-ene-carboxylic acid **(*rac*)-11** (300 mg, 2.26 mmol, 1.00 equiv.), which was then dissolved in dry $CH_2Cl_2$ (0.1 M) and cooled to 0 °C. DMF (35.0 µL, 0.450 mmol, 0.200 equiv.) and oxalyl chloride (287 µL, 3.40 mmol, 1.50 equiv.) were added subsequently and the reaction mixture was stirred at 0 °C for 2 h. Next, dry Methanol (1.83 mL, 45.3 mmol, 20.0 equiv.) was added dropwise at 0 °C and the reaction mixture was allowed to warm to 20 °C. After stirring for 4 h at 20 °C, all volatiles were carefully removed under reduced pressure (product is volatile) at 20 °C. The crude product obtained was purified by column chromatography ($SiO_2$, pentane/$Et_2O$ 95:5 to 90:10) to yield **(*rac*)-S-107** (315 mg, 2.15 mmol, 95%) as a colourless oil.

**$^1$H-NMR** (600 MHz, $CDCl_3$): δ 6.28 (m, 2H), 5.09 (d, *J* = 4.3 Hz, 1H), 4.09 (d, *J* = 4.2 Hz, 1H), 3.77 (s, 3H) ppm.

**$^{13}$C-NMR** (151 MHz, $CDCl_3$): δ 170.4, 140.5, 136.6, 56.4, 54.1, 52.2 ppm.

**HRMS (ESI$^+$):** calculated for $[M+Na]^+$ $(C_6H_7ClO_2Na)^+$ requires m/z 169.0027, found m/z 169.0026.

**FT-IR (neat) $\nu_{max}$:** 2952; 1733, 1622, 1566, 1435, 1409, 1338, 1287, 1233, 1194, 1173, 1140, 1117, 1032, 999, 942, 901, 872, 840, 772, 739 $cm^{-1}$.

### 4.13.2. Methyl-(2E,4E,6E)-9-((2S,3R)-3-((tert-butyldimethylsilyl)oxy)-4-((R,4E,6E)-3-hydroxy-4-methyltetradeca-4,6-dienamido)-2-methylbutanamido)nona-2,4,6-trienoate (S-108)

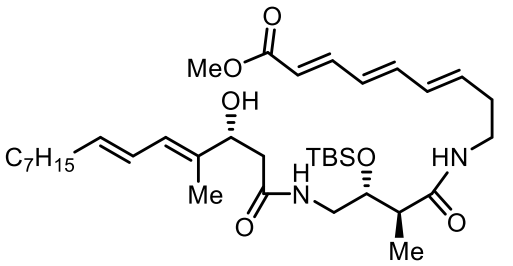

Synthesized according to **General Procedure N** with (±)-*cis*-Methyl-4-chlorocyclobut-2-ene-1-carboxylate **(*rac*)-S-107** (1.30 equiv.) on 0.170 mmol scale to yield **S-108** (89.8 mg, 0.145 mmol, 85%) as a yellow oil.

$^1$**H-NMR** (600 MHz, $CDCl_3$): δ 7.29 (dd, *J* = 15.4, 11.5 Hz, 1H), 6.50 (dd, *J* = 14.9, 10.8 Hz, 1H), 6.31 – 6.27 (m, 1H), 6.07 (d, *J* = 11.0 Hz, 1H), 4.43 (d, *J* = 8.9 Hz, 1H), 3.90 – 3.85 (m, 1H), 3.75 (s, 3H), 3.72 – 3.66 (m, 1H), 3.41 (dq, *J* = 13.2, 6.7 Hz, 1H), 3.34 (s, 1H), 3.29 (td, *J* = 12.7, 6.7 Hz, 1H), 3.01 – 2.92 (m, 1H), 2.10 (q, *J* = 7.2 Hz, 2H), 1.74 (s, 3H), 1.42 – 1.34 (m, 2H), 1.33 – 1.23 (m, 11H), 1.15 (d, *J* = 7.2 Hz, 3H), 0.90 (s, 9H), 0.88 (t, *J* = 7.0 Hz, 3H), 0.15 (s, 3H), 0.09 (s, 3H) ppm.

$^{13}$**C-NMR** (151 MHz, $CDCl_3$): δ 174.6, 172.3, 167.7, 144.7, 140.4, 136.2, 136.0, 135.4, 132.2, 129.1, 125.8, 120.6, 77.4, 77.2, 77.0, 73.8, 72.1, 51.7, 44.8, 43.0, 41.8, 38.8, 33.3, 33.2, 32.0, 29.6, 29.3, 26.0, 22.8, 18.1, 15.5, 14.2, 12.8, 1.2, –4.4, –4.8 ppm.

**HRMS (ESI$^+$):** calculated for $[M+Na]^+$ $(C_{36}H_{62}N_2O_6SiNa)^+$ requires m/z 669.4269, found m/z 669.4272.

**FT-IR (neat) $\nu_{max}$:** 2954, 2925, 2854, 1719, 1699, 1646, 1642, 1617, 1552, 1537, 1527, 1468, 1434, 1361, 1302, 1256, 1231, 1174, 1135, 110, 1040, 1003, 964, 938, 886, 835, 805, 777, 753, 723, 682, 665, 529, 499, 426 cm$^{-1}$.

**$[a]_D^{20}$** = −57° (c = 0.17, $CHCl_3$).

### 4.13.3. Methyl-(2E,4E,6E)-9-((2S,3R)-3-hydroxy-4-((R,4E,6E)-3-hydroxy-4-methyltetradeca-4,6-dienamido)-2-methylbutanamido)nona-2,4,6-trienoate (S-109)

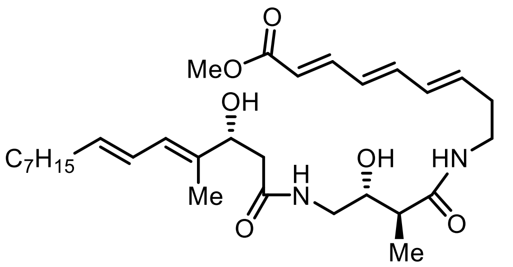

Synthesized according to **General Procedure O** on 7.73 µmol scale to yield **S-109** (3.50 mg, 6.57 µmol, 85%) as a white solid.

All spectroscopic data in accordance with the literature.[7]

**$^1$H-NMR** (400 MHz, $CDCl_3$): δ 7.29 (dd, *J* = 15.2, 11.2 Hz, 1H), 6.51 (dd, *J* = 14.8, 10.7 Hz, 1H), 6.37 – 6.14 (m, 4H), 6.06 (d, *J* = 10.8 Hz, 1H), 5.92 – 5.80 (m, 2H), 5.77 – 5.66 (m, 1H), 4.42 (dd, *J* = 8.8, 3.2 Hz, 2H), 3.74 (s, 3H), 3.66 (bs, 1H), 3.61 – 3.52 (m, 1H), 3.36 (dd, *J* = 12.8, 6.6 Hz, 2H), 3.16 – 3.07 (m, 1H), 2.48 – 2.26 (m, 5H), 2.10 (q, *J* = 7.0 Hz, 2H), 1.73 (s, 3H), 1.64 (bs, 1H), 1.42 – 1.33 (m, 2H), 1.33 – 1.32 (m, 12H), 0.88 (t, *J* = 6.8 Hz, 3H) ppm.

**HRMS (ESI$^+$):** calculated for $[M+Na]^+$ $(C_{30}H_{48}N_2O_6Na)^+$ requires m/z 555.3405, found m/z 555.3408.

### 4.13.4. Fs-FR14

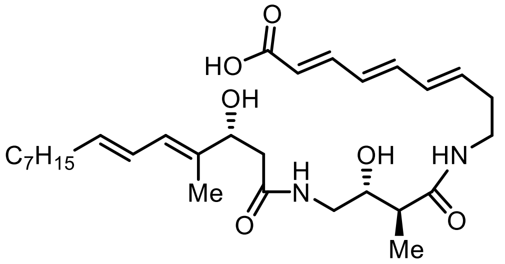


To a flame dried 5 mL round bottom flask under argon was added methyl ester **S-109** (17.1 mg, 32.1 µmol, 1.00 equiv.) was dissolved in 4 mL of a 2:2:1 mixture of THF:MeOH:$H_2O$. After cooling to 0 °C, LiOH (15.4 mg, 64.2 µmol, 20.0 equiv.) was added in one portion and the reaction mixture was allowed to warm to 20 °C over night. Next, THF and methanol were removed under reduced pressure. After diluting with 5 mL EtOAc, the reaction mixture was acidified with 5 mL of a sat. aq. $NaH_2PO_4$ solution. The phases were separated, and the aqueous phase was extracted 3 x 5 mL EtOAc. The combined organic extracts were washed with 5 mL brine and subsequently dried over $MgSO_4$. After filtration from the drying agent, all volatiles were removed under reduced pressure to yield **Fs-FR14** (16.1 mg, 31.8 µmol, 99%) as a white solid.

All spectroscopic data in accordance with the literature.[7]

**$^1$H-NMR** (600 MHz, MeOH-$d_4$): δ 7.27 (dd, *J* = 15.1, 11.3 Hz, 1H), 6.59 (dd, *J* = 14.9, 10.7 Hz, 1H), 6.32 (dd, *J* = 14.9, 11.3 Hz, 1H), 6.26 (m, 2H), 6.04 (d, *J* = 10.8 Hz, 1H), 5.98 – 5.92 (m, 1H), 5.85 (d, *J* = 15.2 Hz, 1H), 5.71 – 5.65 (m, 1H), 4.42 (dd, *J* = 8.6, 4.7 Hz, 1H), 3.72 – 3.68 (m, 1H), 3.42 (dd, *J* = 13.8, 4.0 Hz, 1H), 3.28 (t, *J* = 6.9 Hz, 2H), 3.15 (dd, *J* = 13.8, 7.0 Hz, 1H), 2.47 – 2.42 (m, 1H), 2.41 – 2.32 (m, 4H), 2.11 (q, *J* = 14.1,

7.0 Hz, 2H), 1.75 (s, 3H), 1.44 – 1.37 (m, 3H), 1.36 – 1.26 (m, 9H), 1.12 (d, *J* = 7.0 Hz, 3H), 0.90 (t, *J* = 7.1 Hz, 3H) ppm.

**HRMS (ESI$^+$):** calculated for $[M+Na]^+$ $(C_{29}H_{46}N_2O_6Na)^+$ requires m/z 541.3248, found m/z 541.3249.

# 5. Role of T3P in esterification with cyclobutene (*rac*)-6.

Esterification of cyclobutene carboxylic acid was proven a challenging step due to significant epimerization of the activated cyclobutene carboxylate which eventually afforded triene systems with (*Z,E,E*) configuration. Ghosez's reagent has previously been the coupling agent of choice for our protocol, outperforming EDCI, DCC, and other traditional coupling agents.[1] Nevertheless, the acidic proton at the putative acid chloride intermediate renders this species susceptible to epimerization, which kept being the major obstacle in this reaction. Furthermore, the expected *N*,*N*-dimethylisobutyramide byproduct coelutes with the desired esters, resulting in further challenging purifications and low isolated yields. T3P, on the other hand, results in a less acidic mixed anhydride, significantly decreasing the degree of undesired epimerization. Moreover, the phosphonate byproduct is scarcely soluble in organic solvents and allows for efficient extractions, eventually leading to facile separations and higher yields compared to when other coupling agents are deployed.

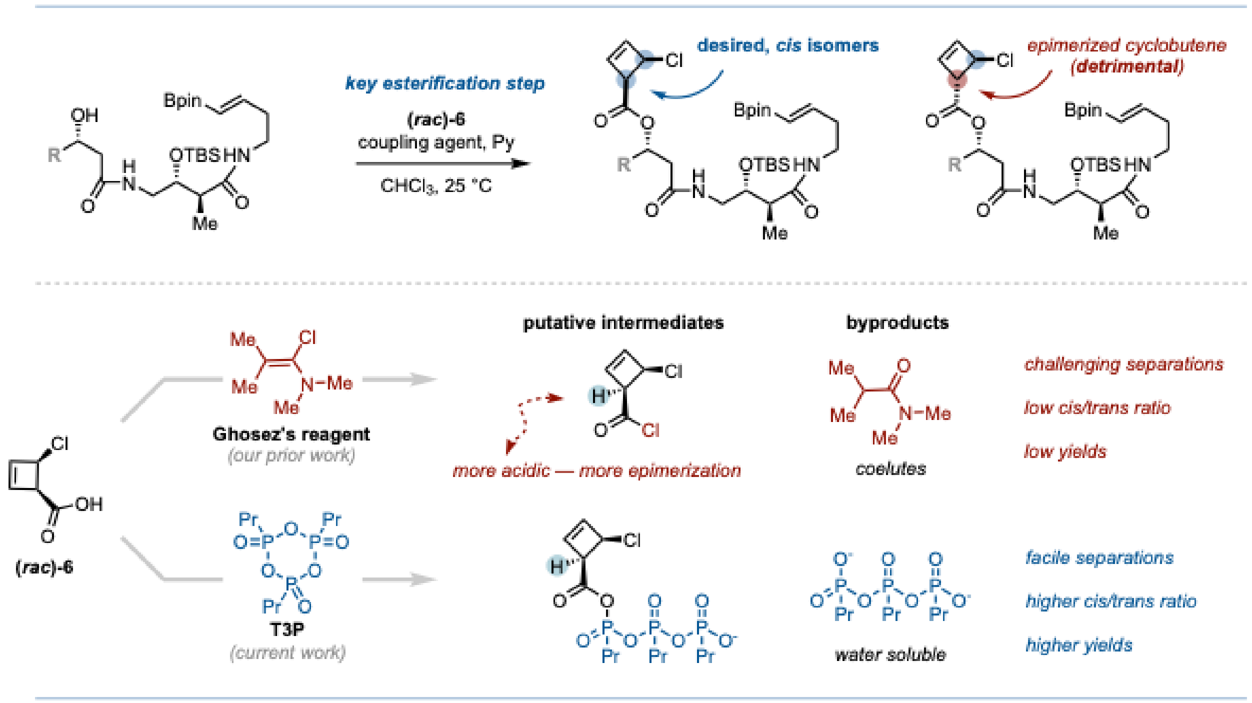
key esterification step
(rac)-6
coupling agent, Py
CHCl3, 25 °C
desired, cis isomers
epimerized cyclobutene
(detrimental)
Bpin
OTBS HN
Me
(rac)-6
Ghosez's reagent
(our prior work)
T3P
(current work)
putative intermediates
more acidic — more epimerization
byproducts
coelutes
water soluble
challenging separations
low cis/trans ratio
low yields
facile separations
higher cis/trans ratio
higher yields

# 6. NMR Spectra

## (*E*)-1,1-Dimethyl-2-((2*E*,4*E*)-2-methyldodeca-2,4-dien-1-ylidene)hydrazine (10a)

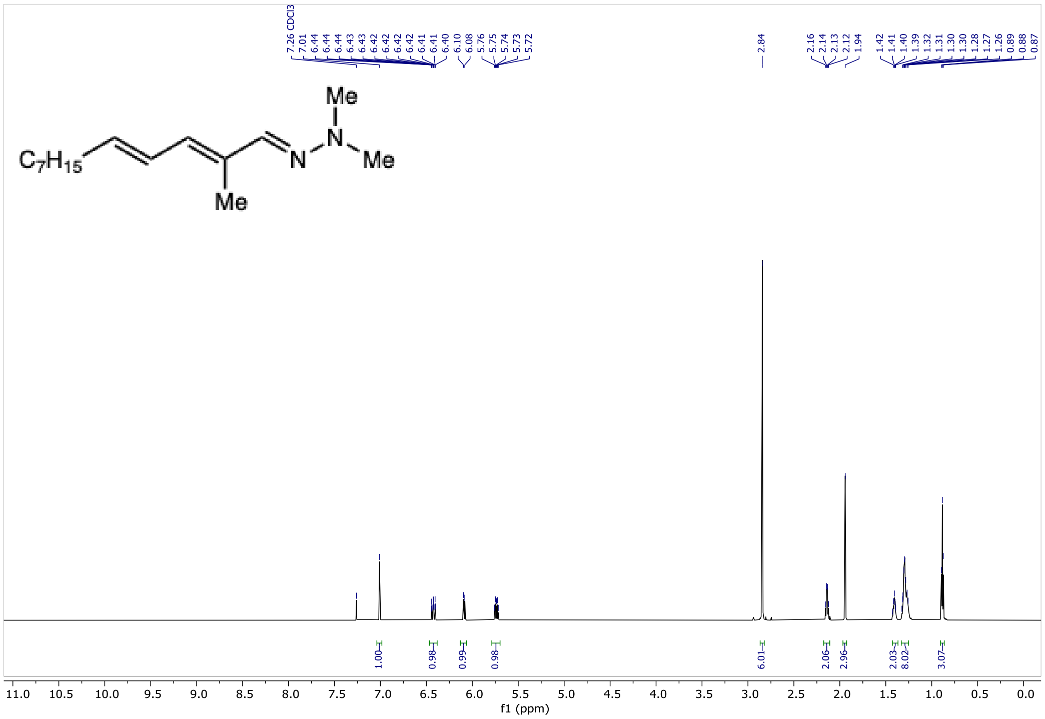


$^{1}H$
$CDCl_3$
600 MHz

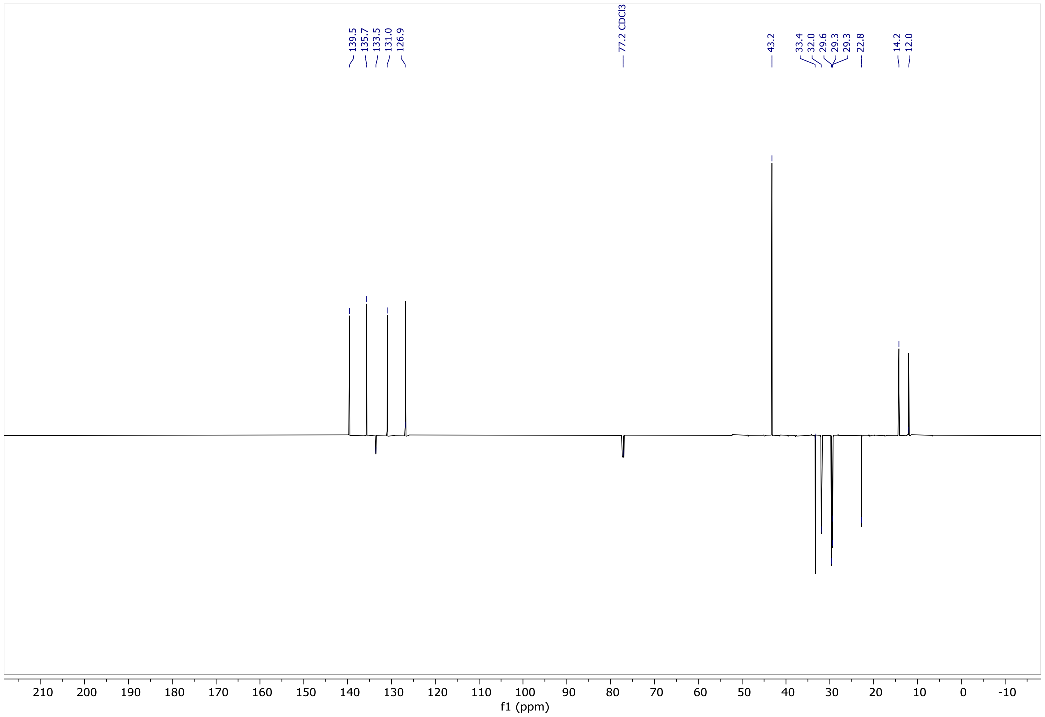


$^{13}C$
$CDCl_3$
151 MHz

## Ethyl (2*E*,4*E*)-2-methylhexa-2,4-dienoate (S-51)

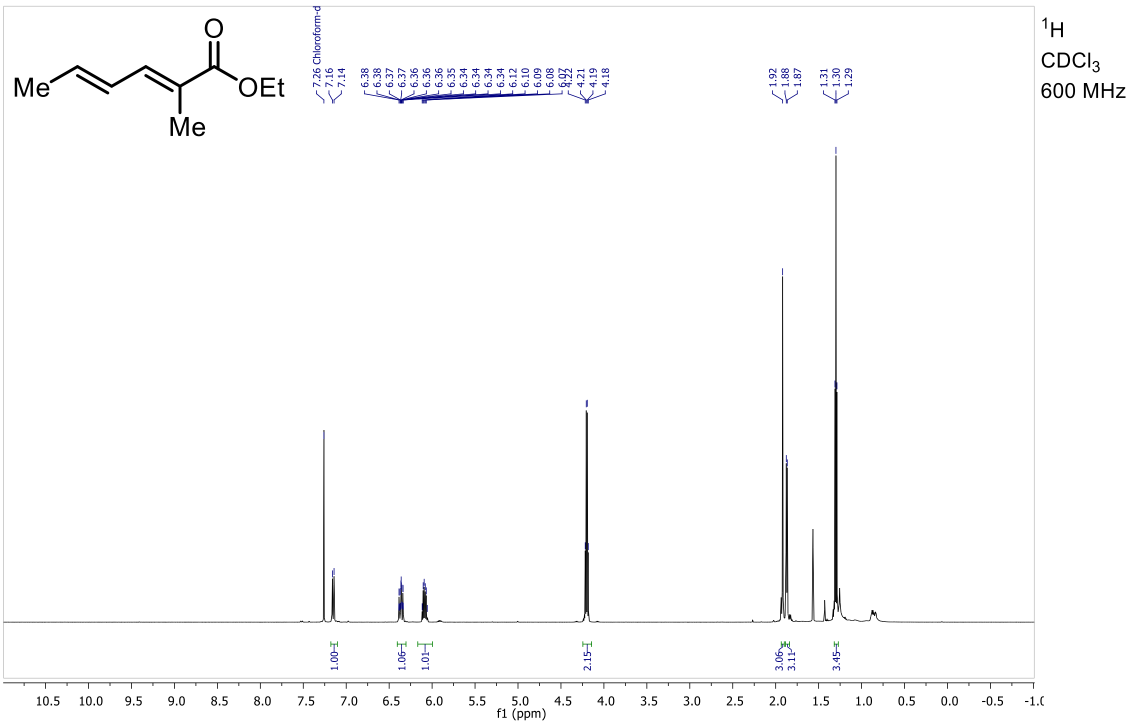


$^1H$
$CDCl_3$
600 MHz

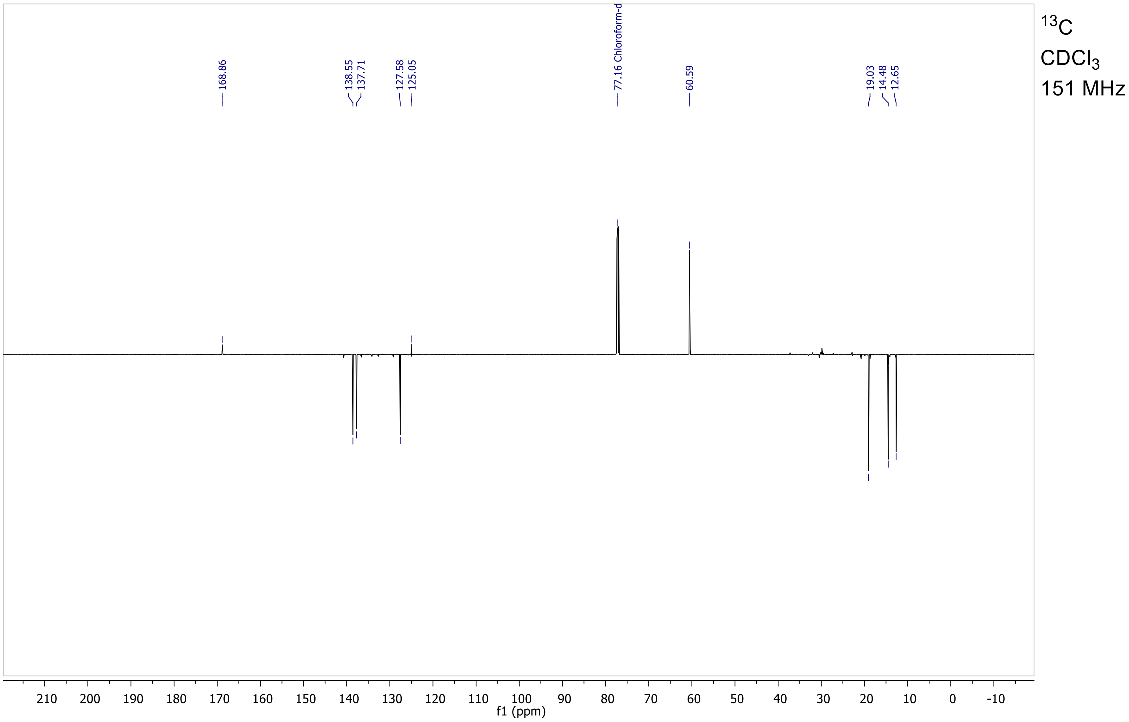


$^{13}C$
$CDCl_3$
151 MHz

((*R*,4*E*,6*E*)-1-((*S*)-4-benzyl-2-thioxothiazolidin-3-yl)-3-hydroxy-4-methylocta-4,6-dien-1-one (*S*-53)

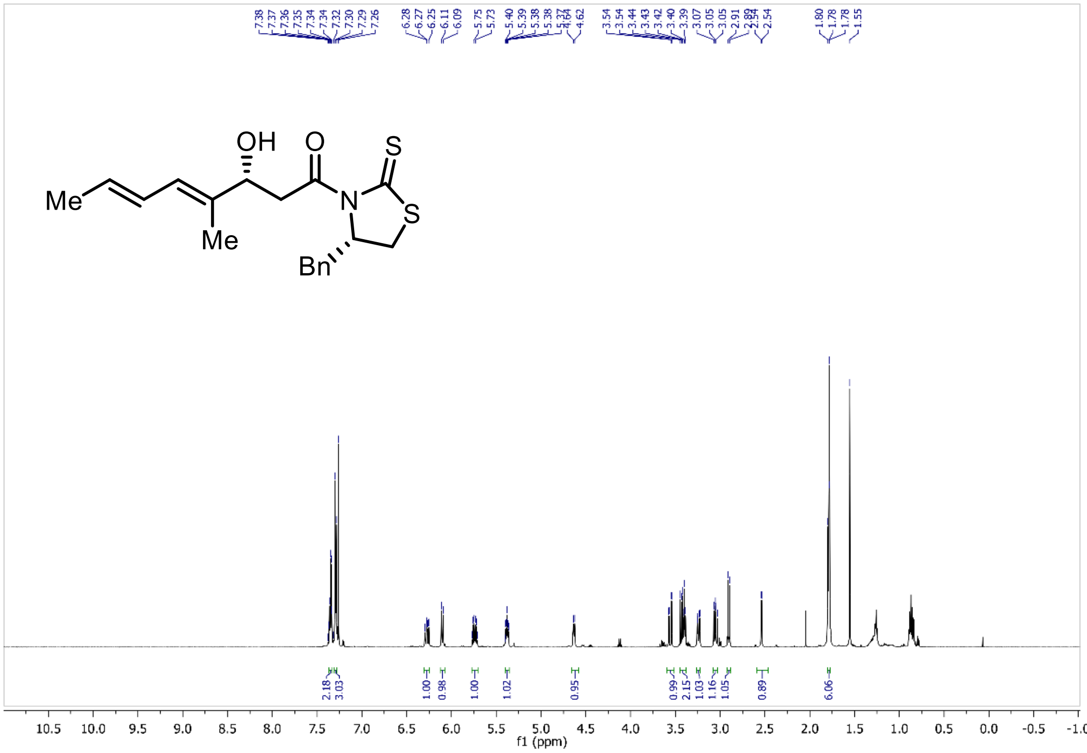


$^{1}H$
$CDCl_3$
600 MHz

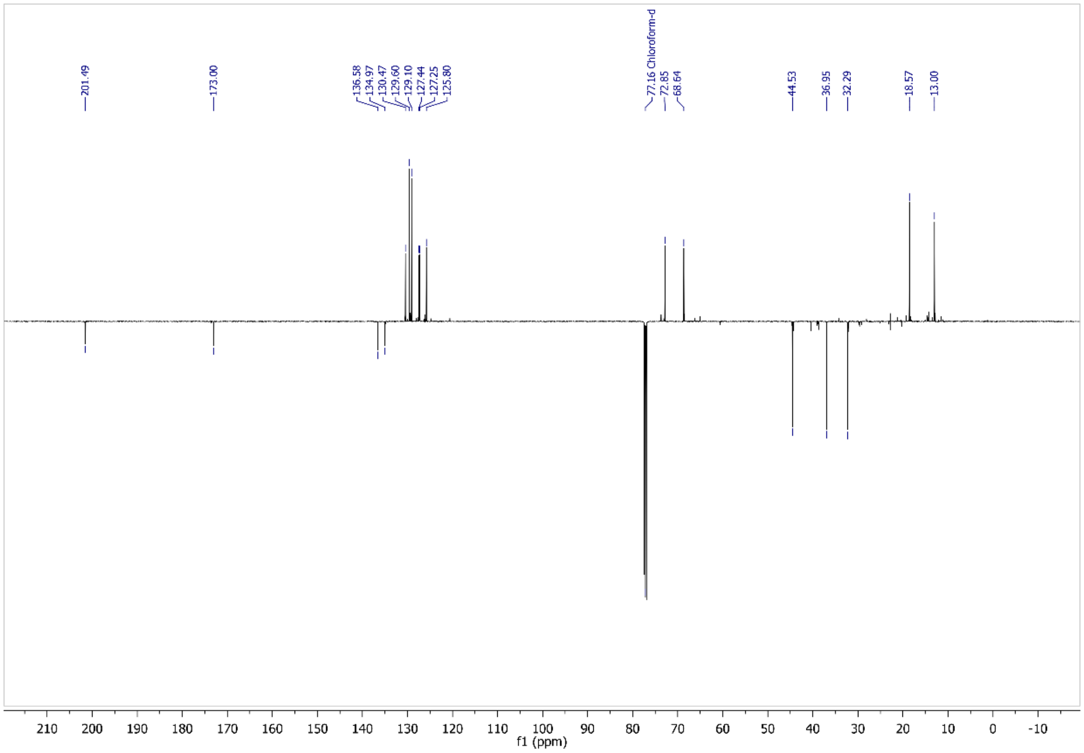


$^{13}C$
$CDCl_3$
151 MHz

(*R*,4*E*,6*E*)-*N*-((2*R*,3*S*)-2-((*tert*-butyldimethylsilyl)oxy)-3-methyl-4-oxo-4-(((*E*)-4-(4,4,5,5-tetramethyl-1,3,2-dioxaborolan-2-yl)but-3-en-1-yl)amino)butyl)-3-hydroxy-4-methylocta-4,6-dienamide (S-54)

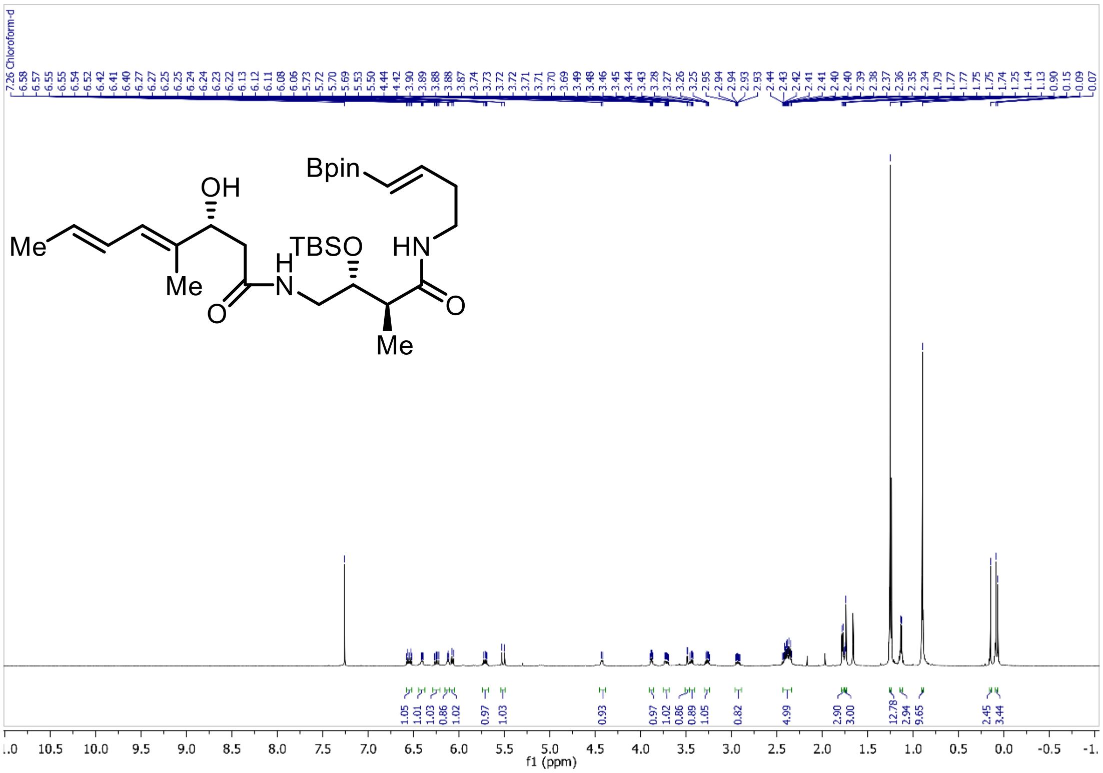


$^{1}H$
$CDCl_3$
600 MHz

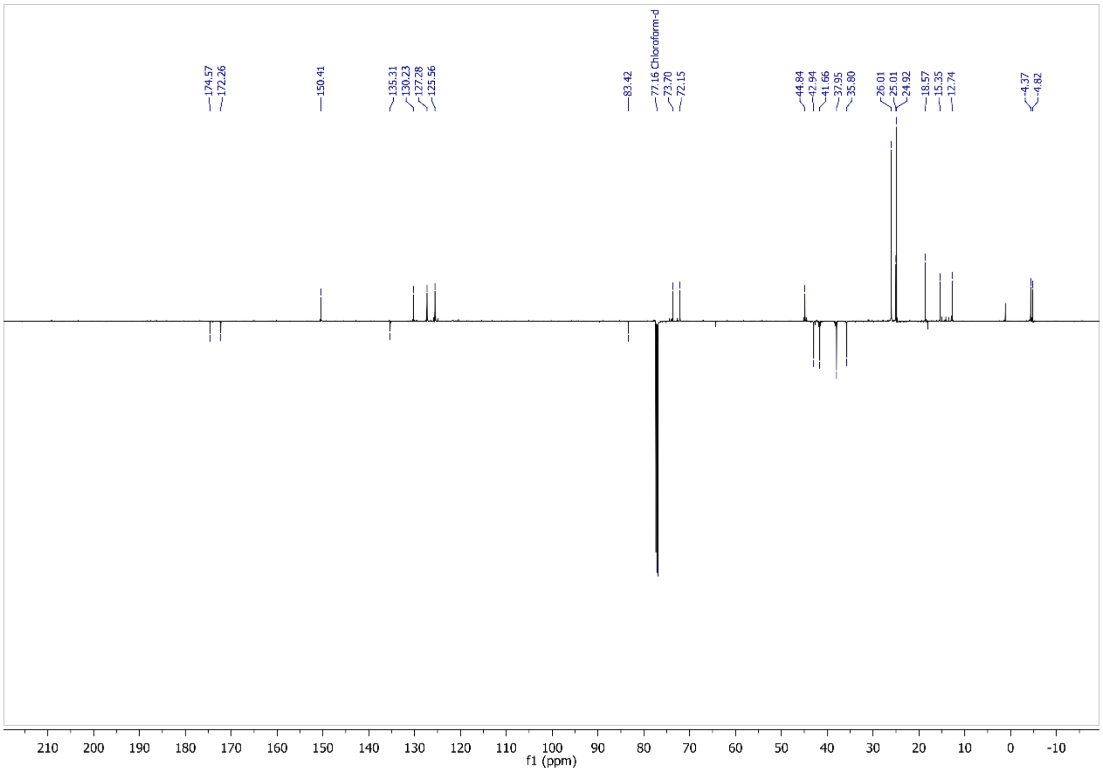


$^{13}C$
$CDCl_3$
151 MHz

(*R*,4*E*,6*E*)-1-(((2*R*,3*S*)-2-((*tert*-butyldimethylsilyl)oxy)-3-methyl-4-oxo-4-(((*E*)-4-(4,4,5,5-tetramethyl-1,3,2-dioxaborolan-2-yl)but-3-en-1-yl)amino)butyl)amino)-4-methyl-1-oxoocta-4,6-dien-3-yl (1*R*,4*R*)-4-chlorocyclobut-2-ene-1-carboxylate (S-55)

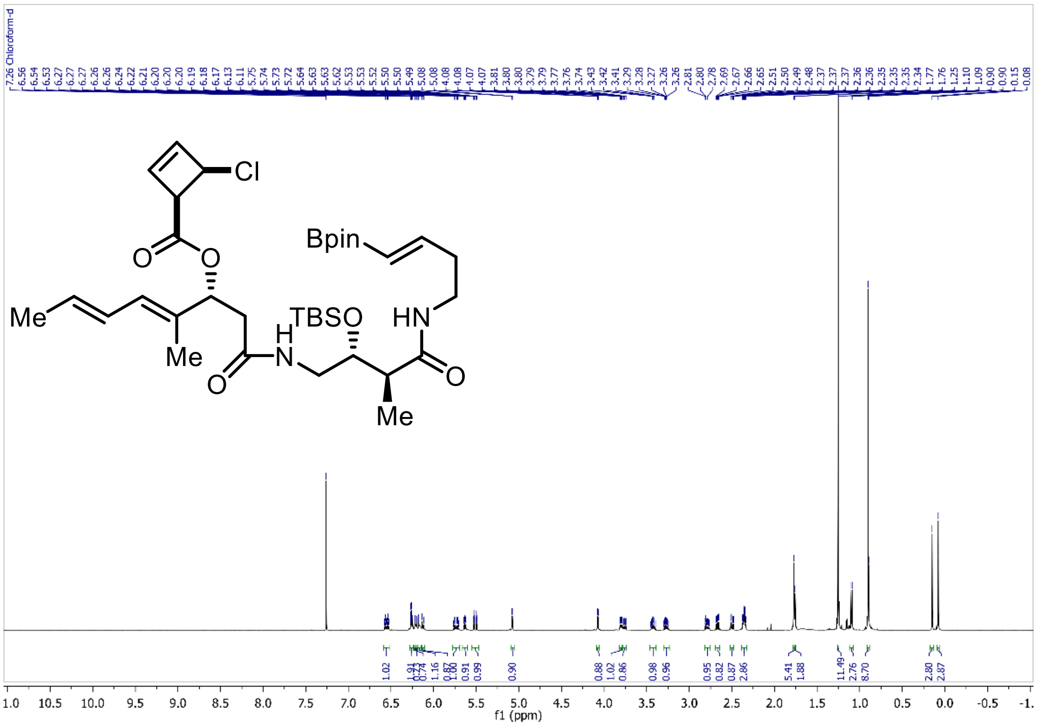


$^{1}H$
$CDCl_3$
600 MHz

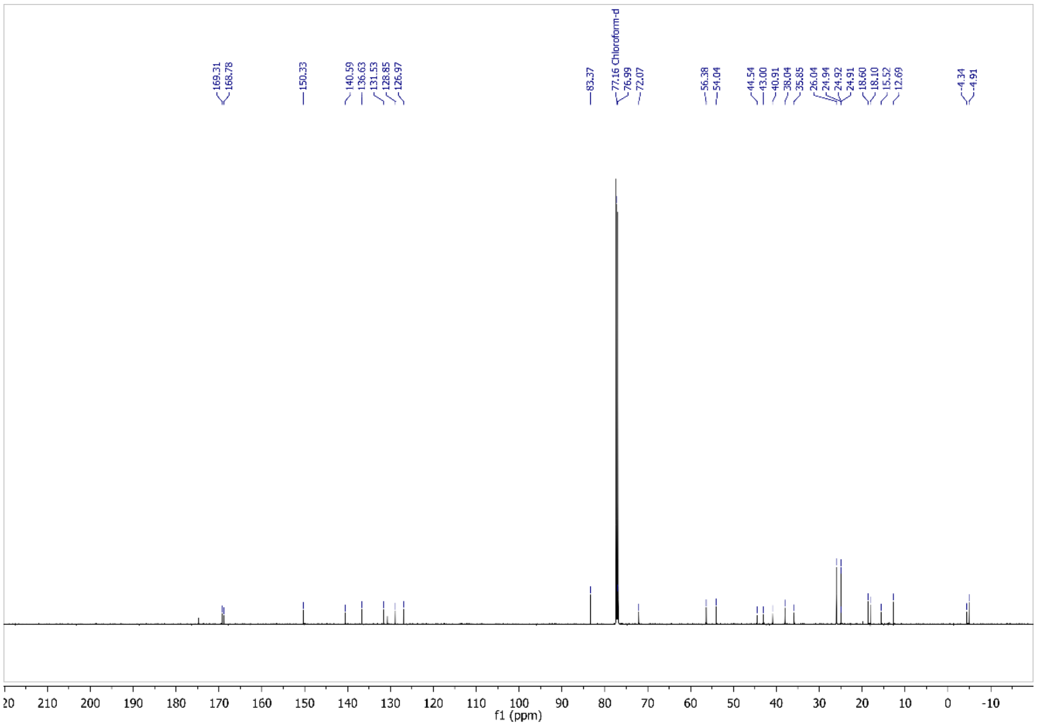


$^{13}C$
$CDCl_3$
151 MHz

(2*R*,7*R*,8*S*,13*E*,15*E*,17*E*)-7-((*tert*-butyldimethylsilyl)oxy)-2-((2*E*,4*E*)-hexa-2,4-dien-2-yl)-8-methyl-1-oxa-5,10-diazacyclononadeca-13,15,17-triene-4,9,19-trione (S-56)

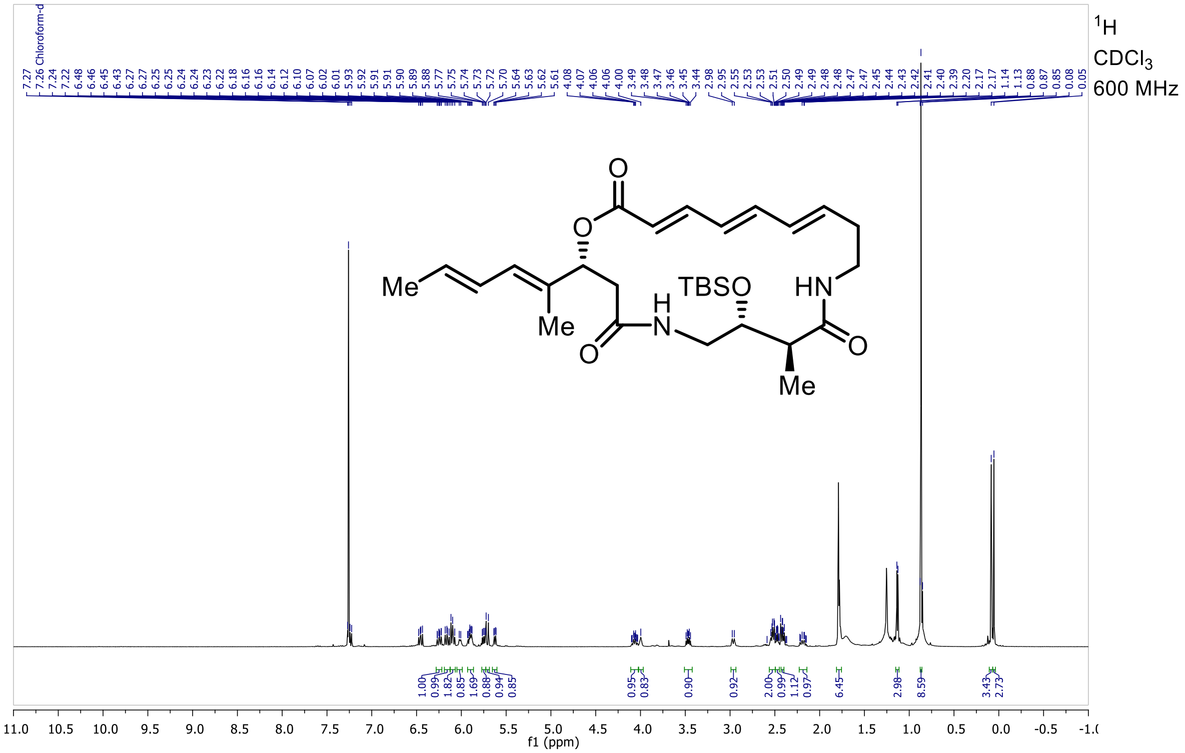


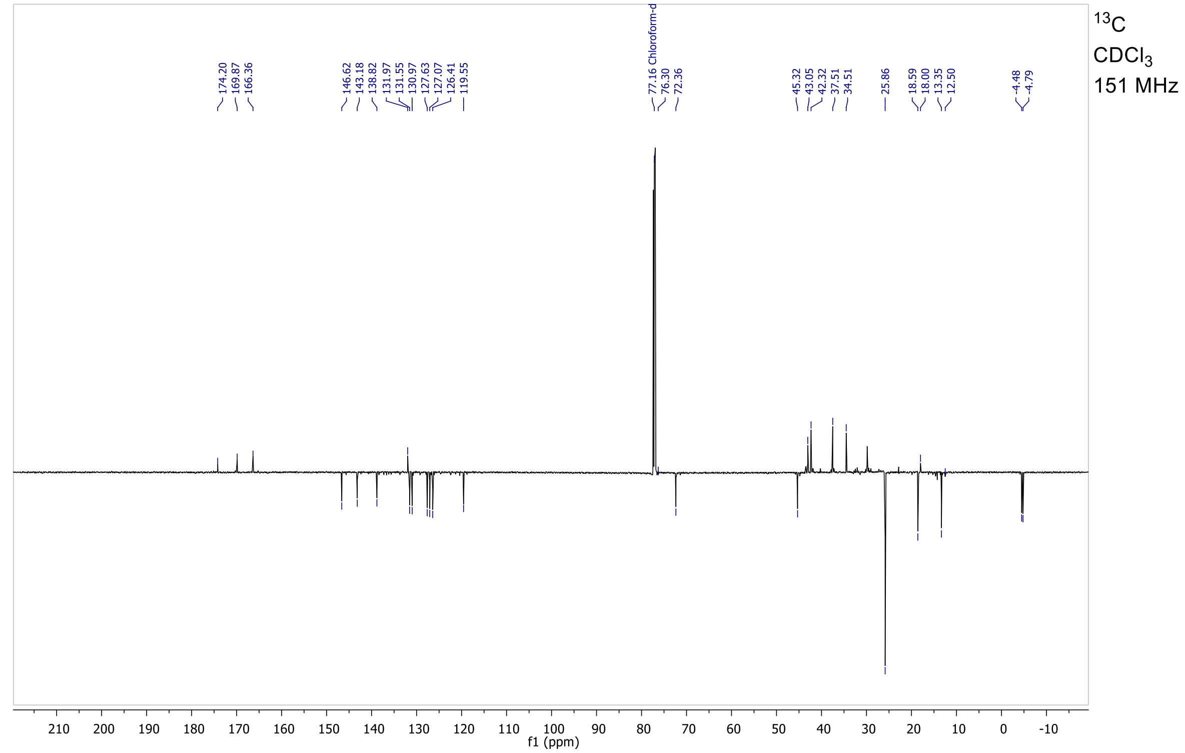

## Fs-FR2

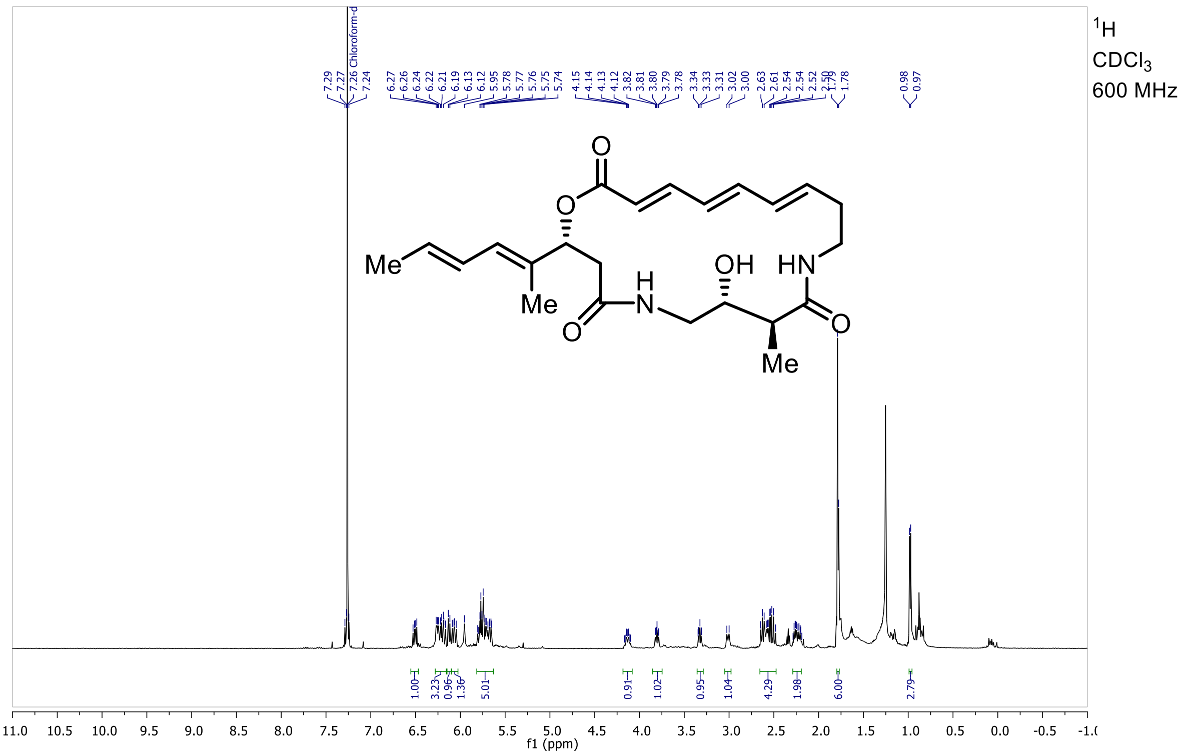


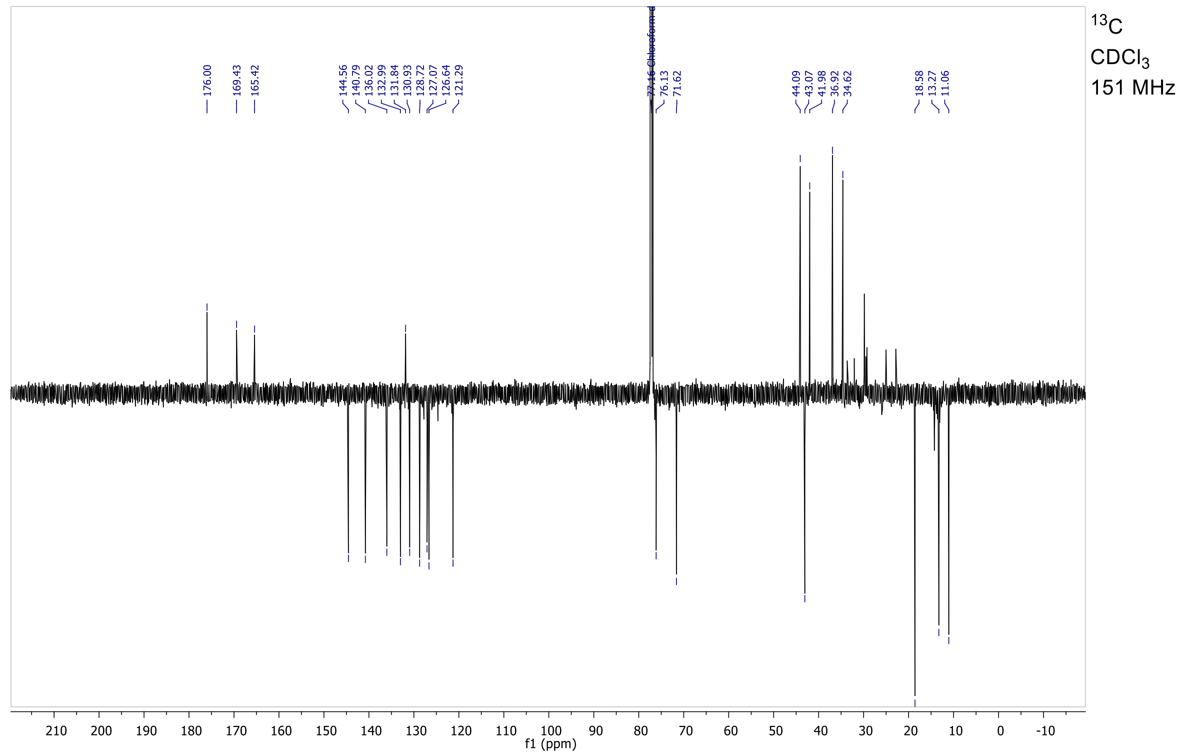

(*R*,4*E*,6*E*)-1-((*S*)-4-Benzyl-2-thioxothiazolidin-3-yl)-3-hydroxytetradeca-4,6-dien-1-one (S-58)

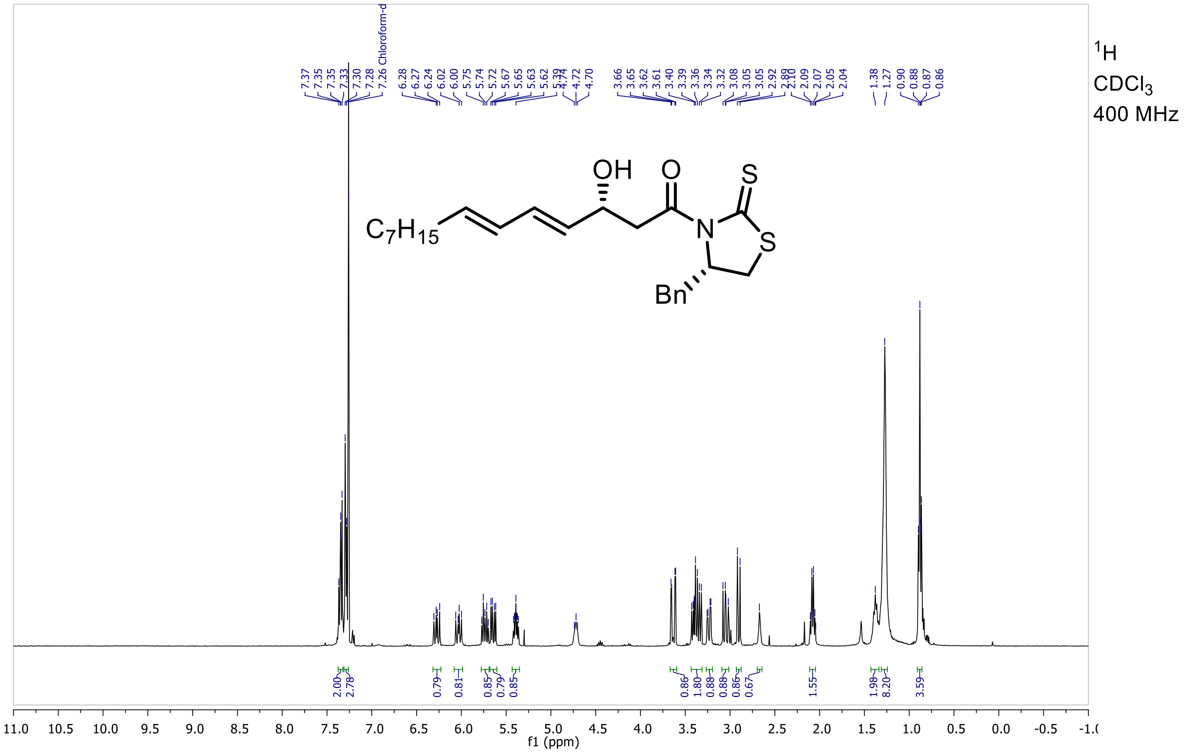


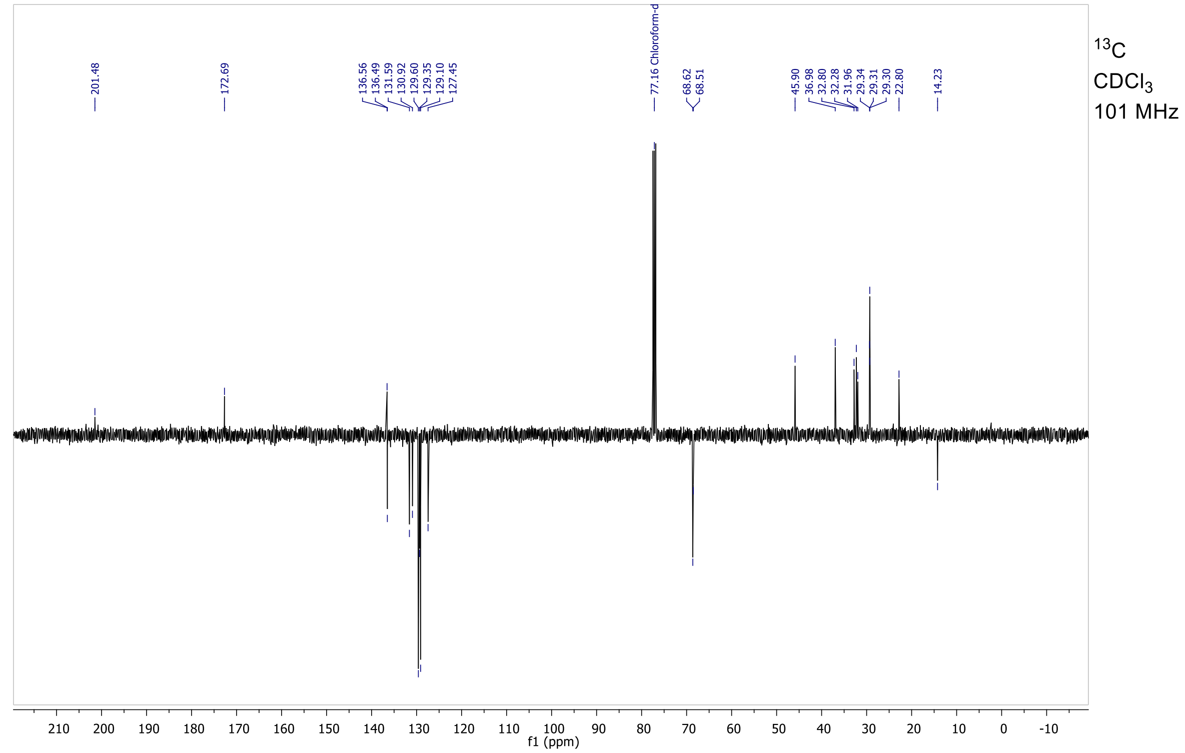

(*R*,4*E*,6*E*)-*N*-((2*R*,3*S*)-2-((*tert*-Butyldimethylsilyl)oxy)-3-methyl-4-oxo-4-(((*E*)-4-(4,4,5,5-tetramethyl-1,3,2-dioxaborolan-2-yl)but-3-en-1-yl)amino)butyl)-3-hydroxytetradeca-4,6-dienamide (S-59)

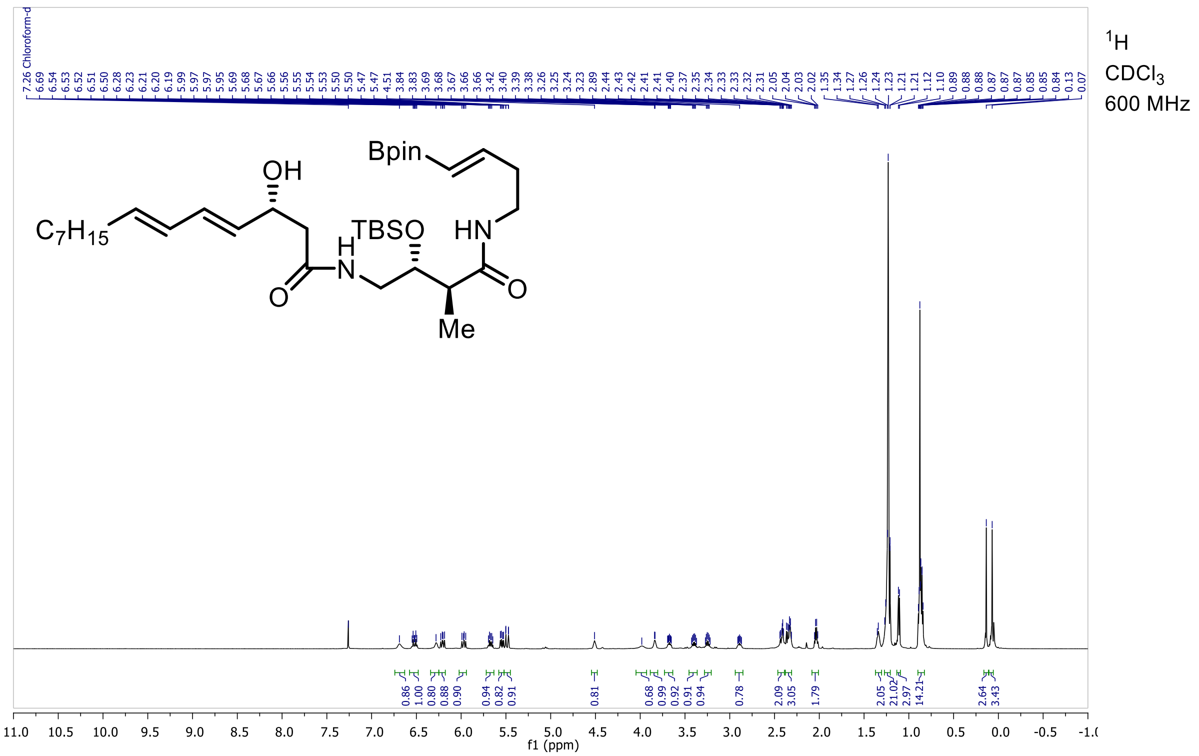


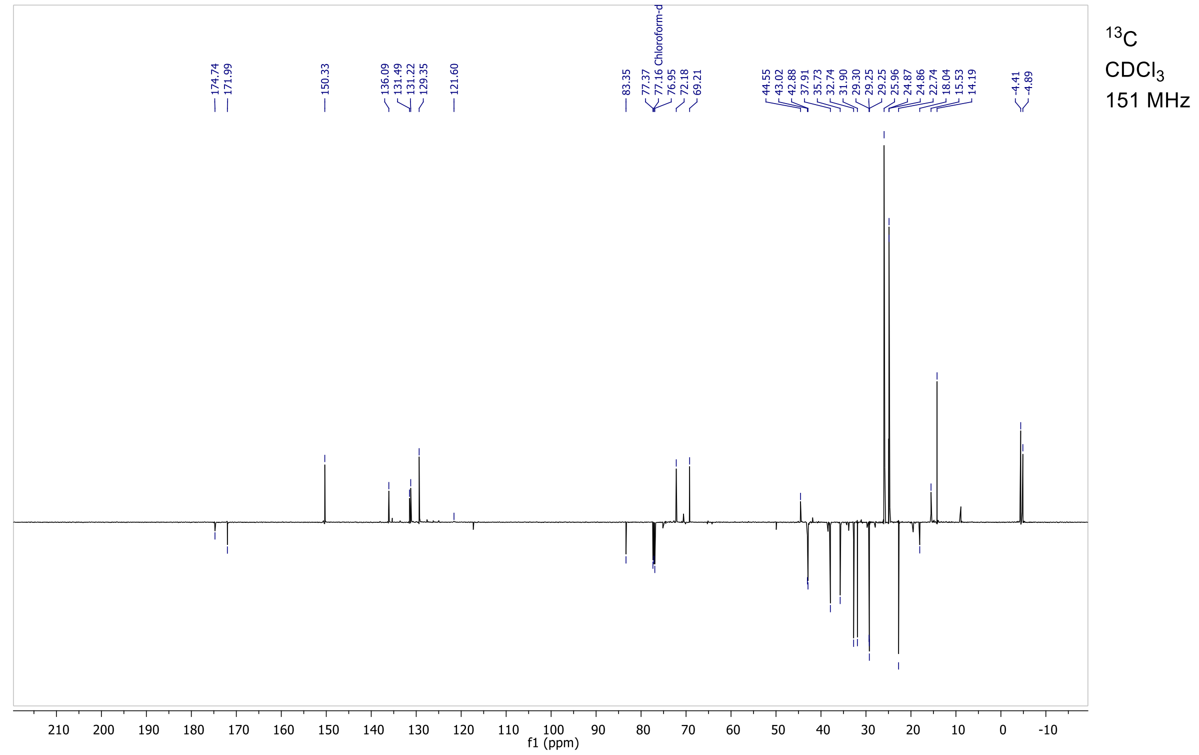

(*R*,4*E*,6*E*)-1-(((2*R*,3*S*)-2-((*tert*-Butyldimethylsilyl)oxy)-3-methyl-4-oxo-4-(((*E*)-4-(4,4,5,5-tetramethyl-1,3,2-dioxaborolan-2-yl)but-3-en-1-yl)amino)butyl)amino)-1-oxotetradeca-4,6-dien-3-yl (1*S*,4*R*)-4-chlorocyclobut-2-ene-1-carboxylate (S-60)

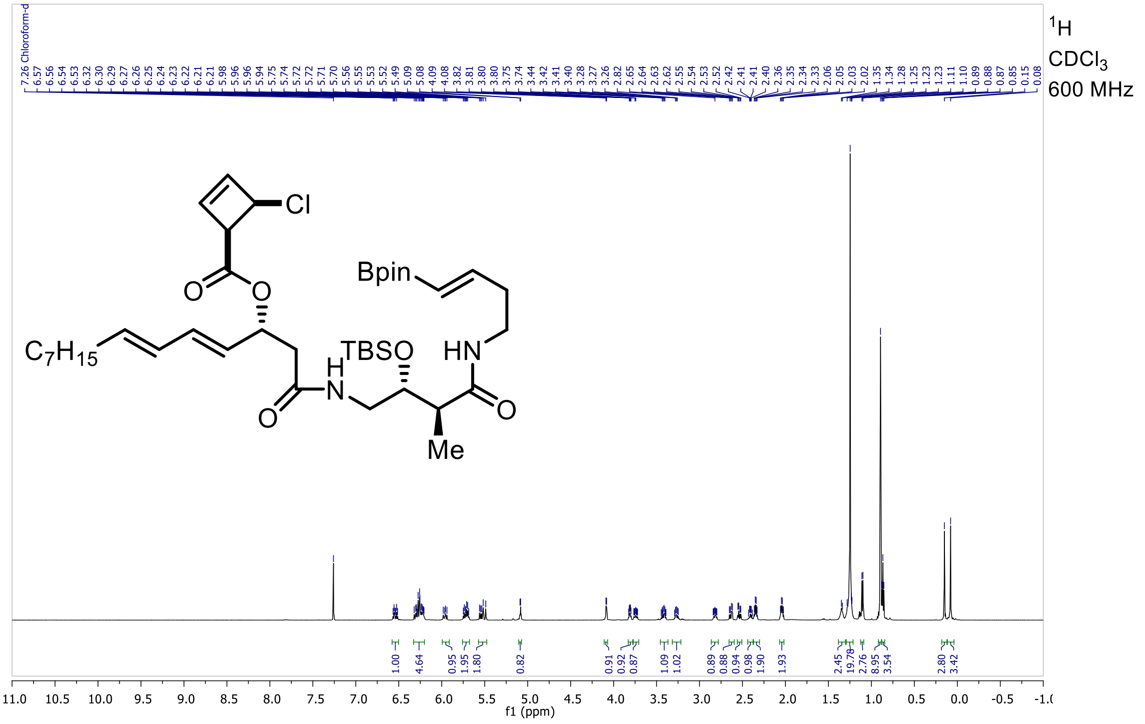


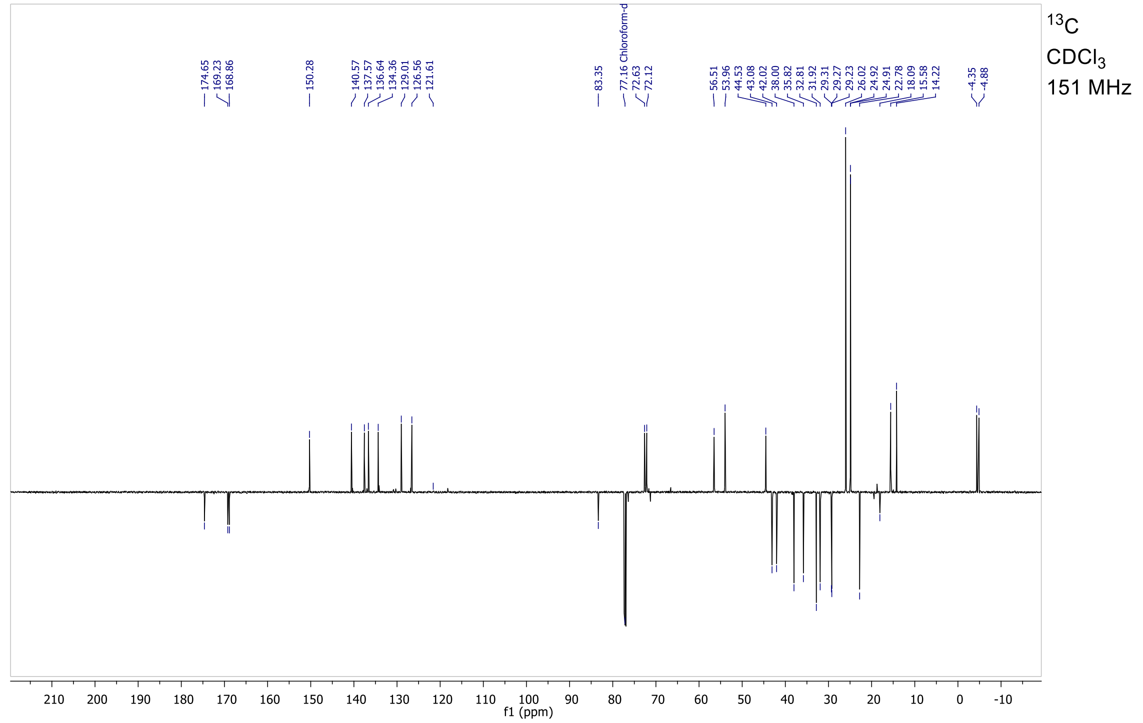

(2*R*,7*R*,8*S*,13*E*,15*E*,17*E*)-7-((*tert*-Butyldimethylsilyl)oxy)-8-methyl-2-((1*E*,3*E*)-undeca-1,3-dien-1-yl)-1-oxa-5,10-diazacyclononadeca-13,15,17-triene-4,9,19-trione (S-61)

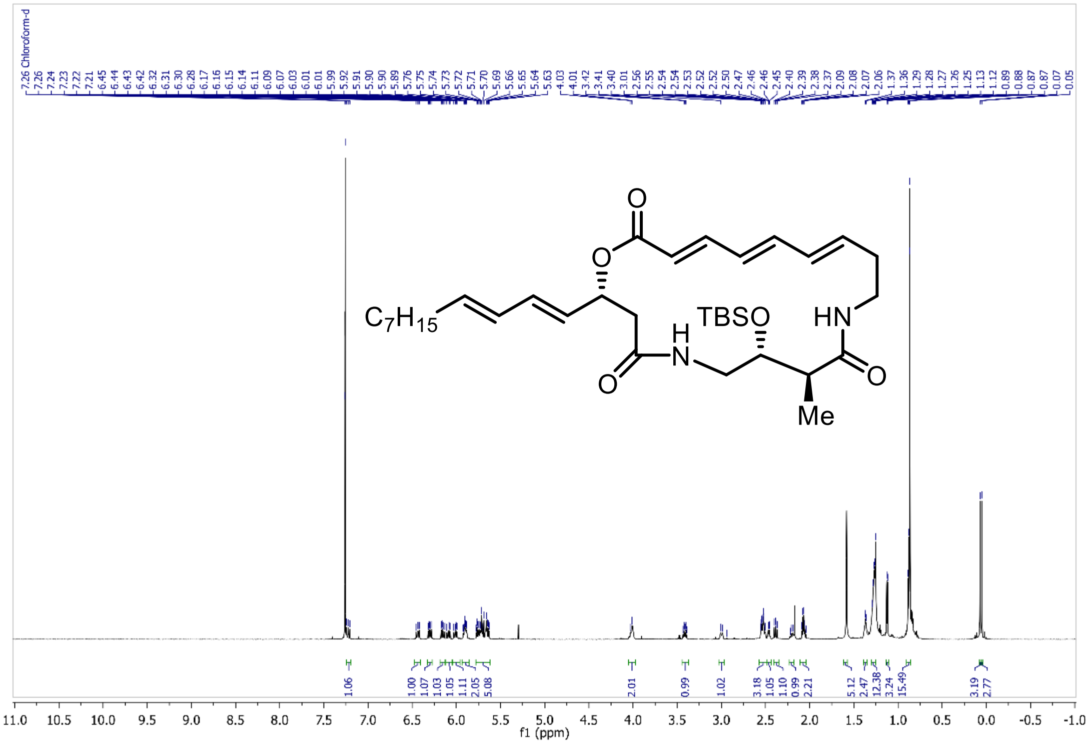


$^1H$
$CDCl_3$
700 MHz

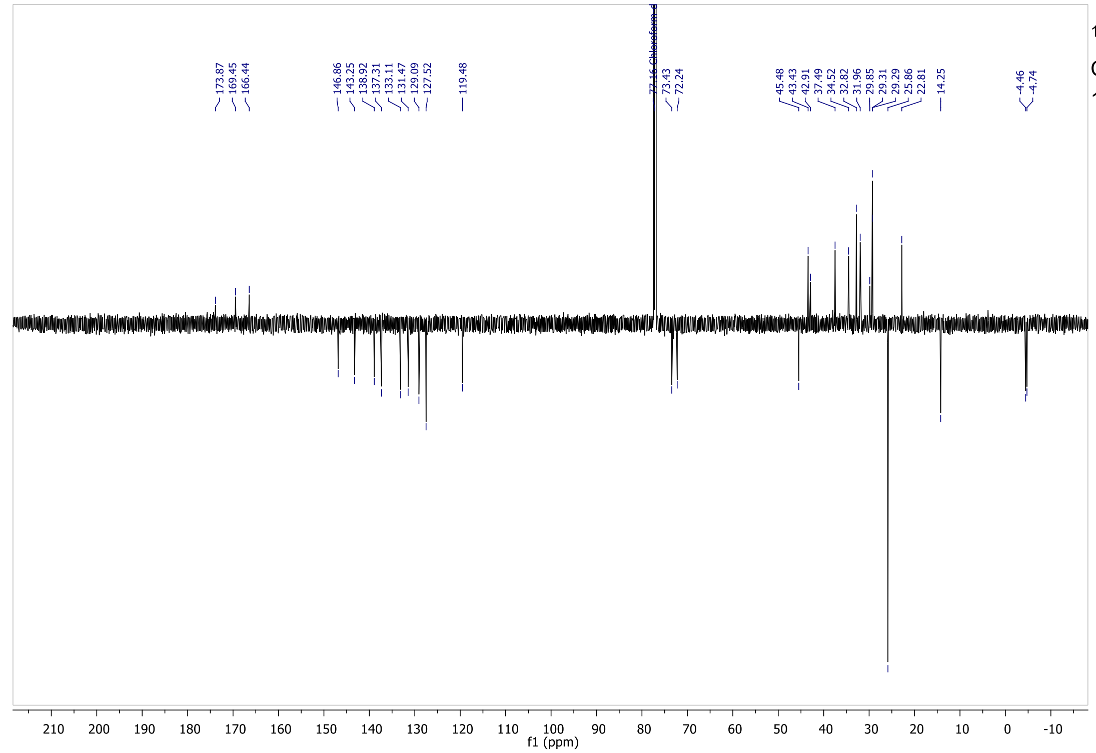


$^{13}C$
$CDCl_3$
176 MHz

## Fs-FR3

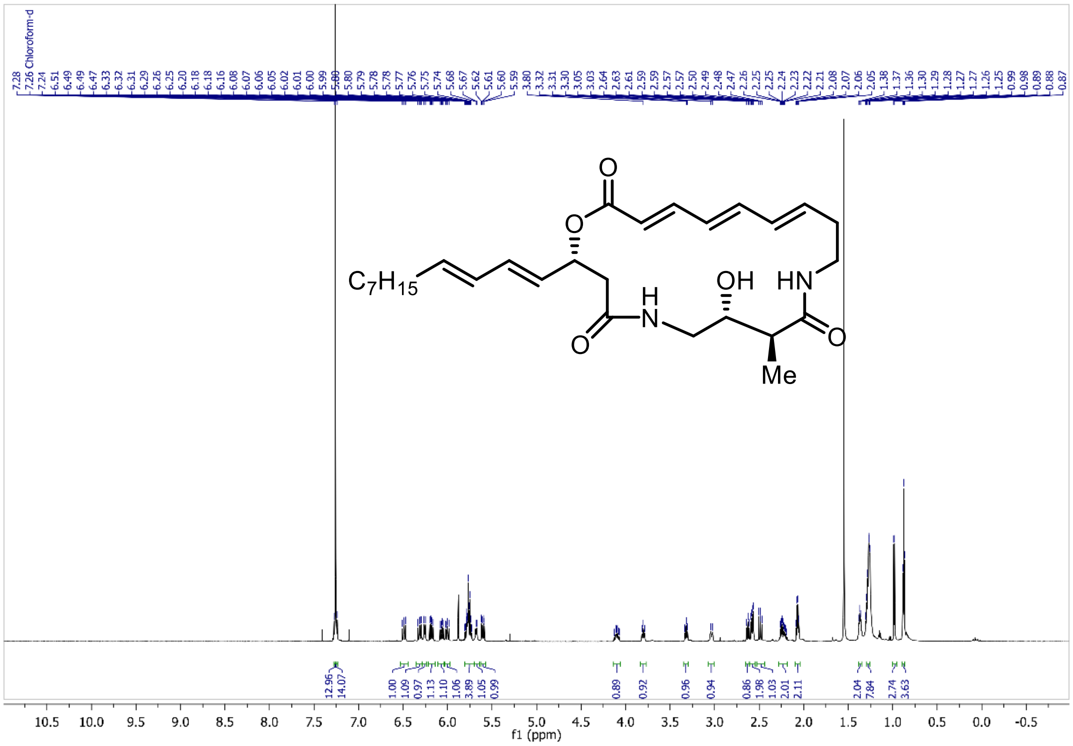


$^{1}H$
$CDCl_3$
700 MHz

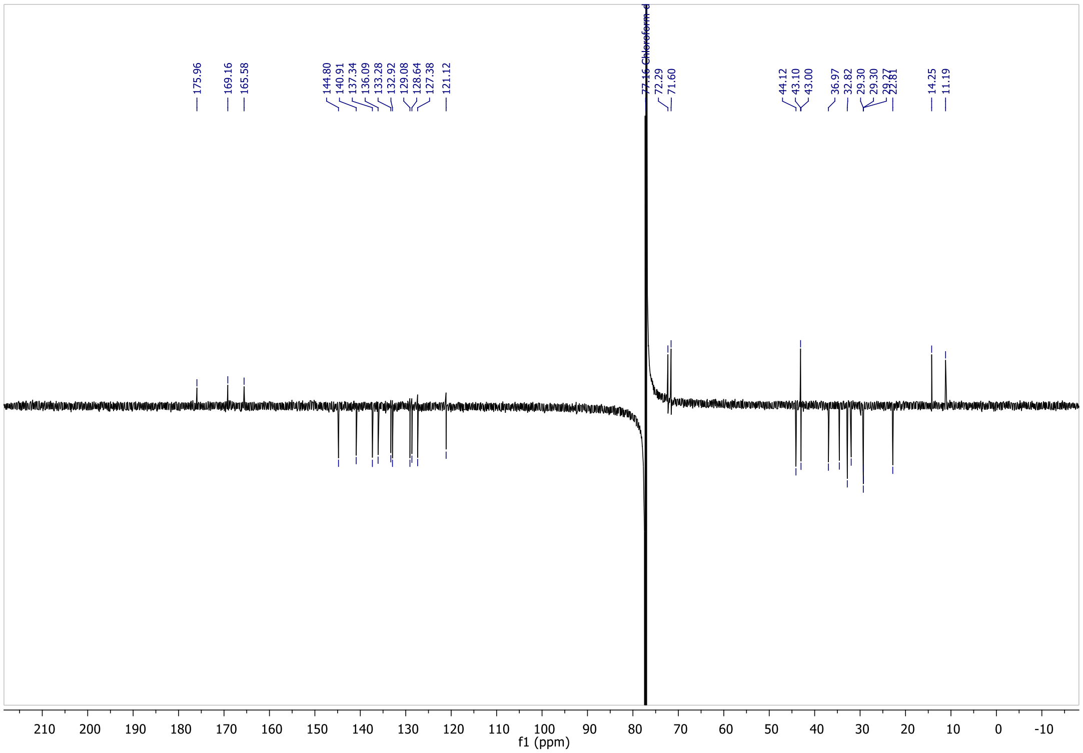


$^{13}C$
$CDCl_3$
176 MHz

Ethyl (2Z,4E)-2-fluorododeca-2,4-dienoate ((Z)-S-63) & Ethyl (2E,4E)-2-fluorododeca-2,4-dienoate ((*E*)-S-63)

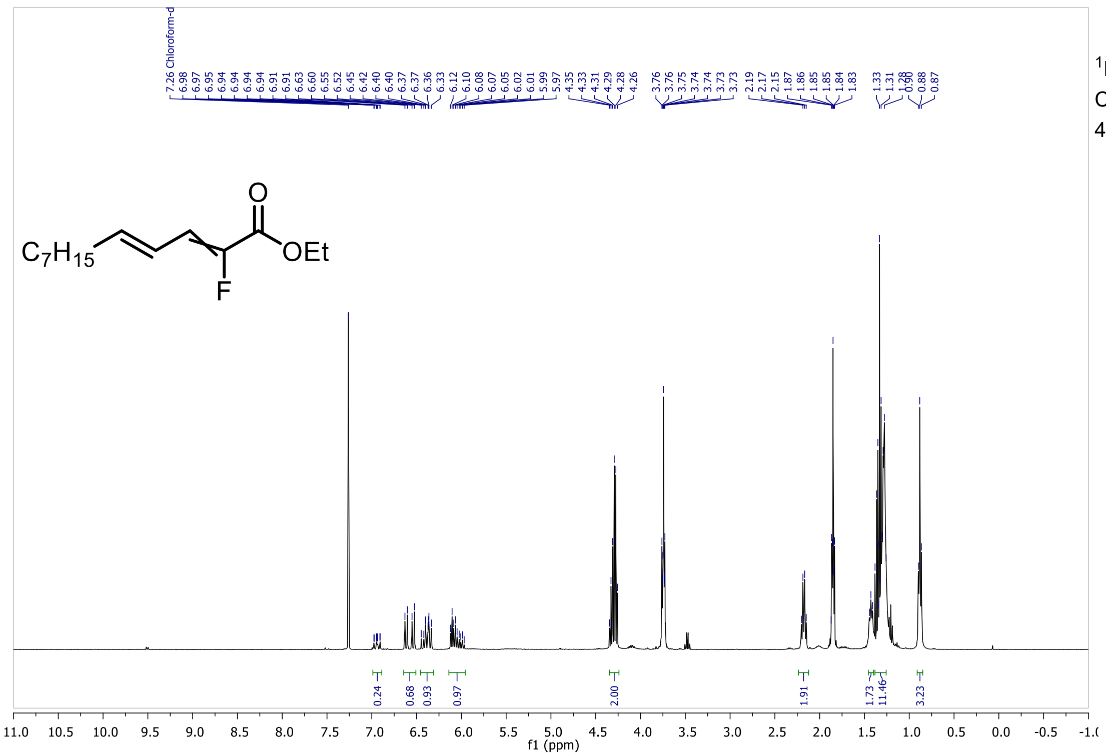


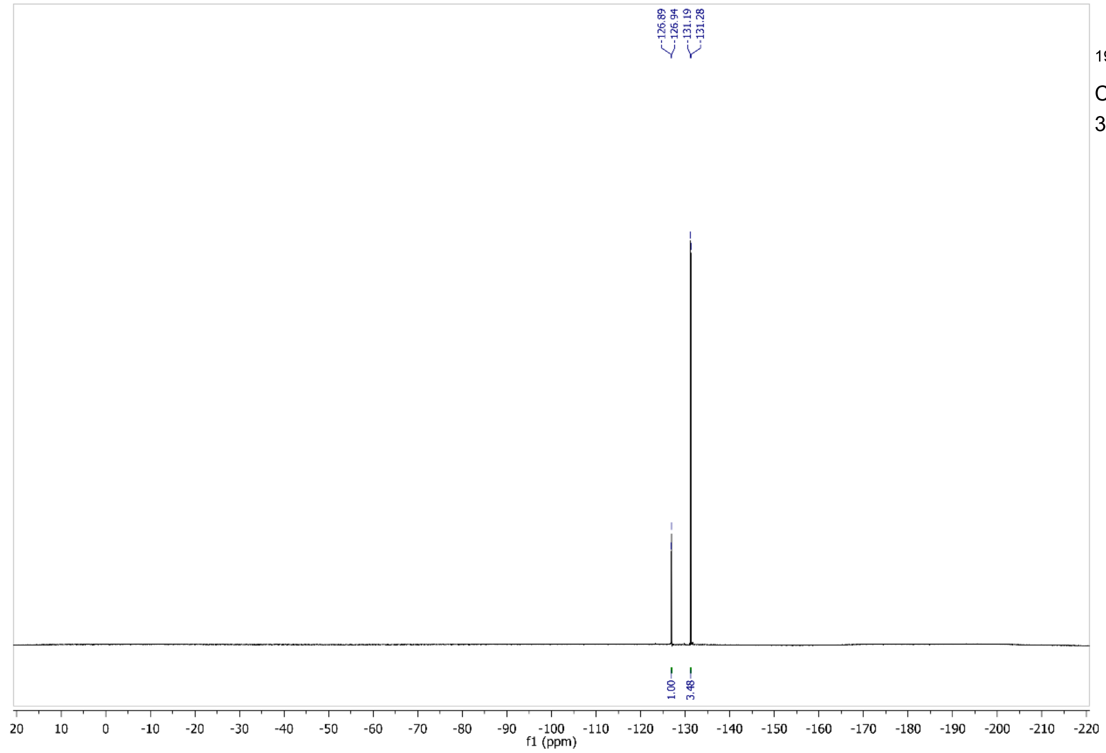

(2*Z*,4*E*)-2-fluorododeca-2,4-dien-1-ol (S-63a)

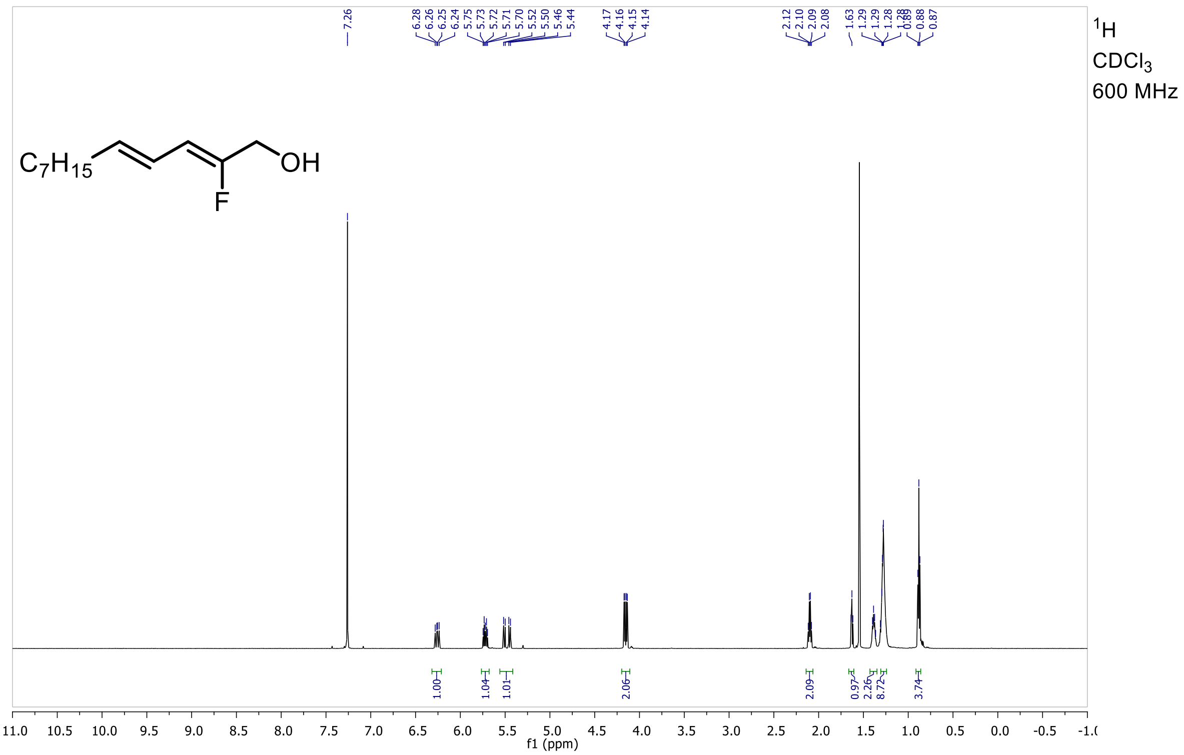


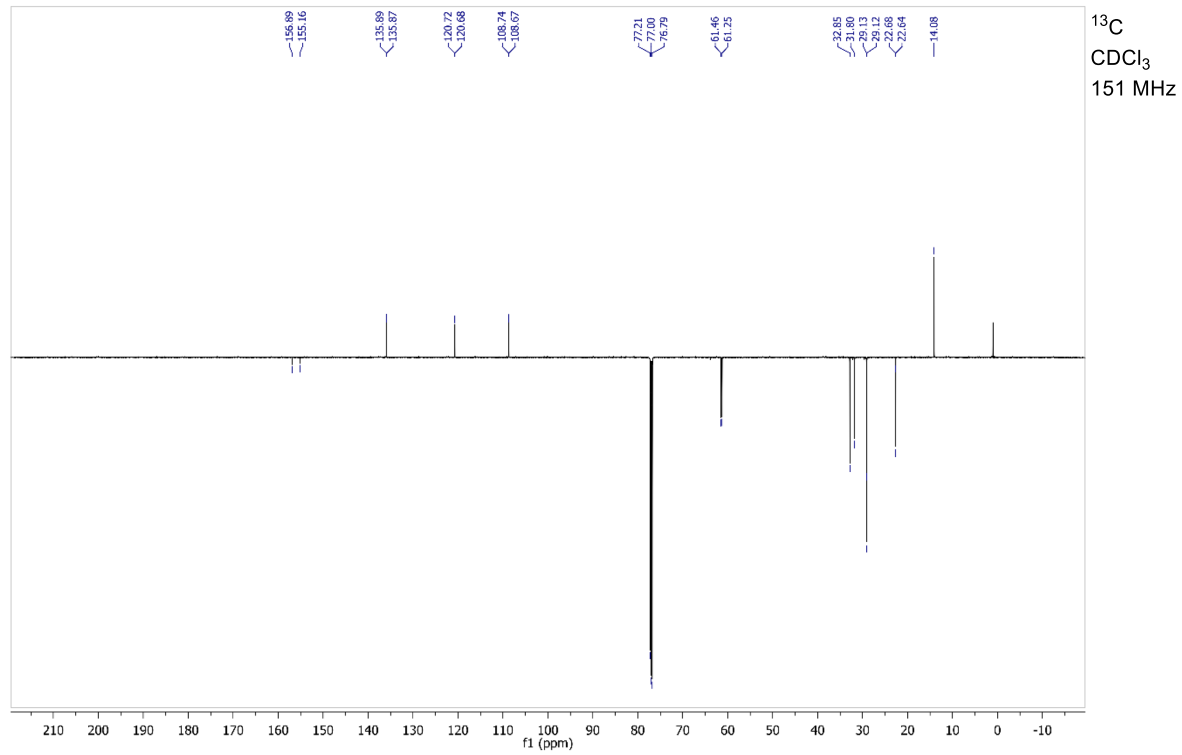

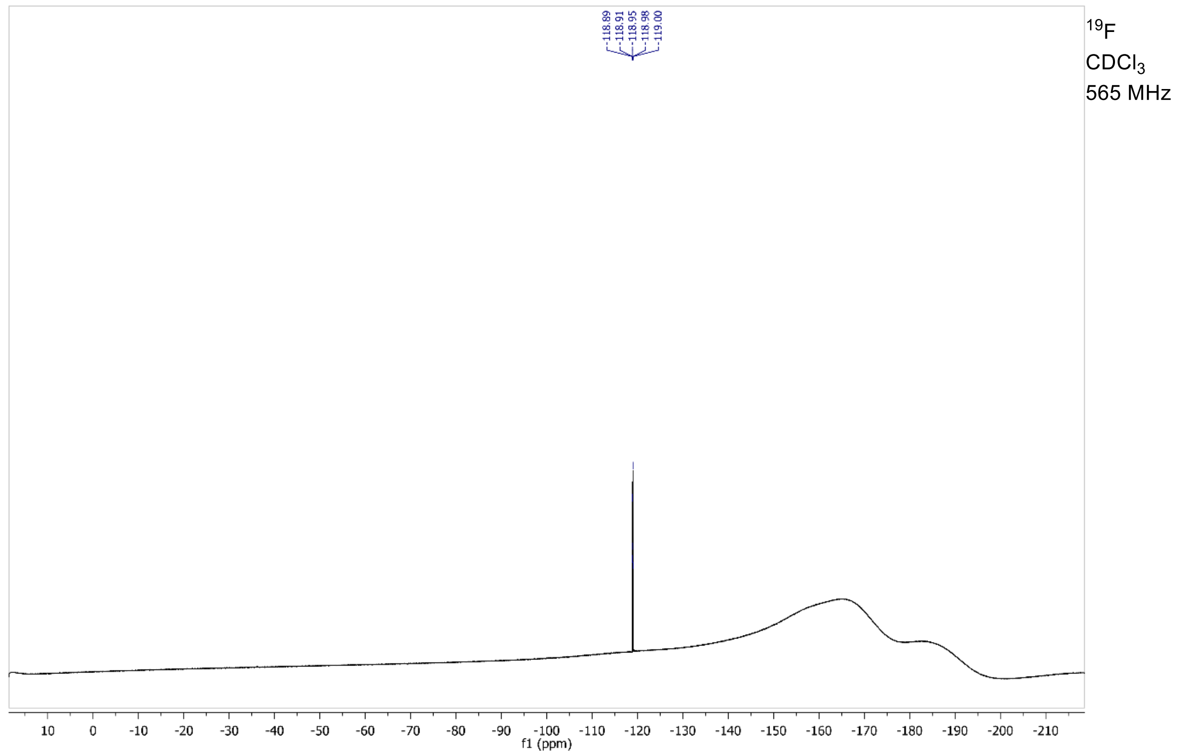


(2*Z*,4*E*)-2-fluorododeca-2,4-dienal (S-64)

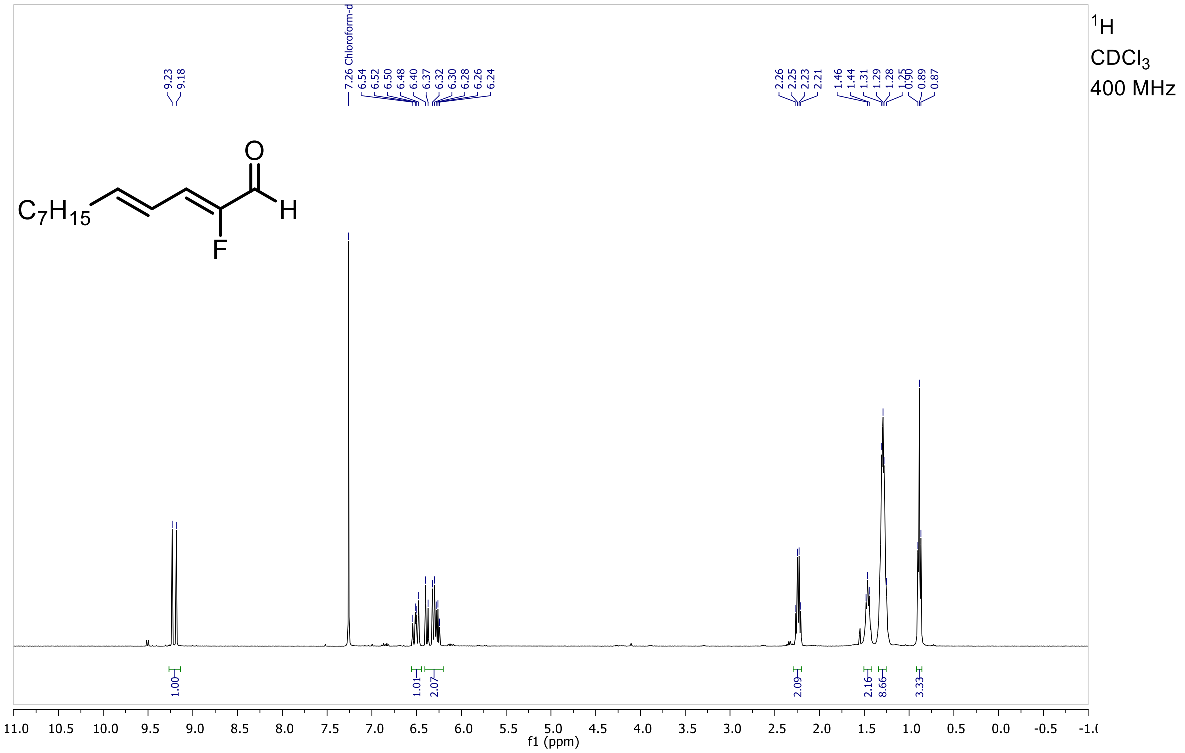

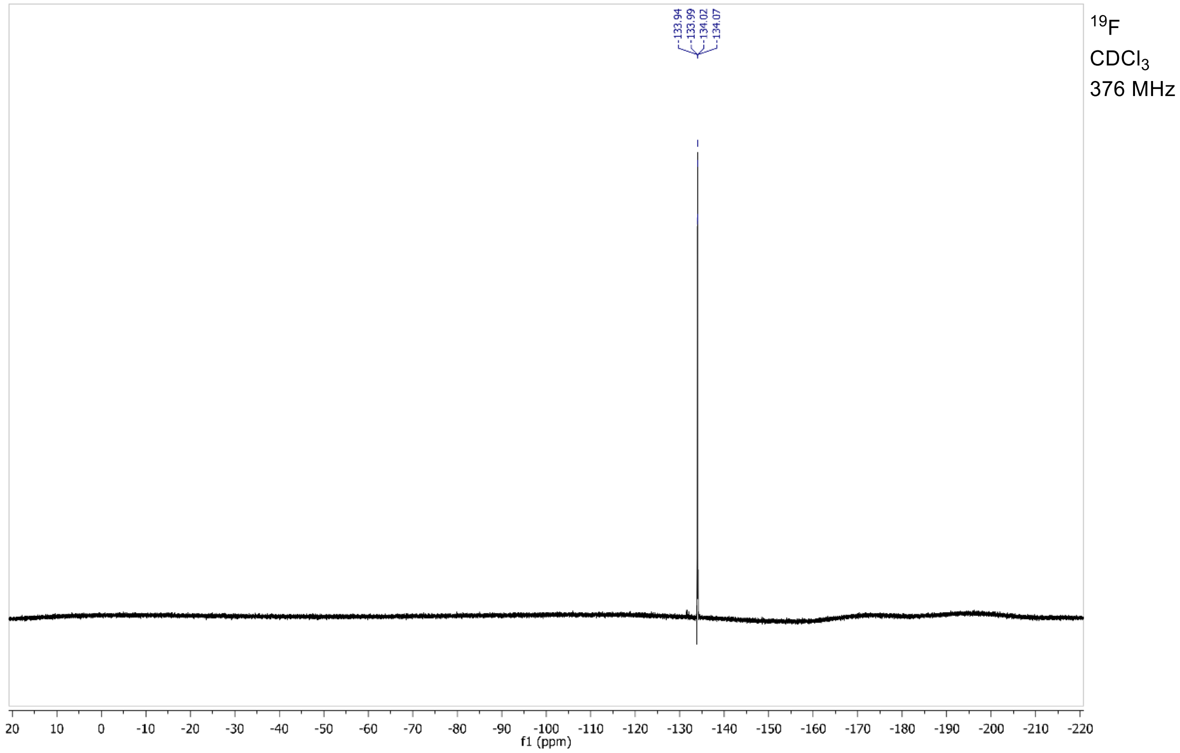
-133.94
-133.99
-134.02
-134.07
19F
CDCl3
376 MHz
20 10 0 -10 -20 -30 -40 -50 -60 -70 -80 -90 -100 -110 -120 -130 -140 -150 -160 -170 -180 -190 -200 -210 -220
f1 (ppm)

(*R*,4*Z*,6*E*)-1-((*S*)-4-benzyl-2-thioxothiazolidin-3-yl)-4-fluoro-3-hydroxytetradeca-4,6-dien-1-one (4)

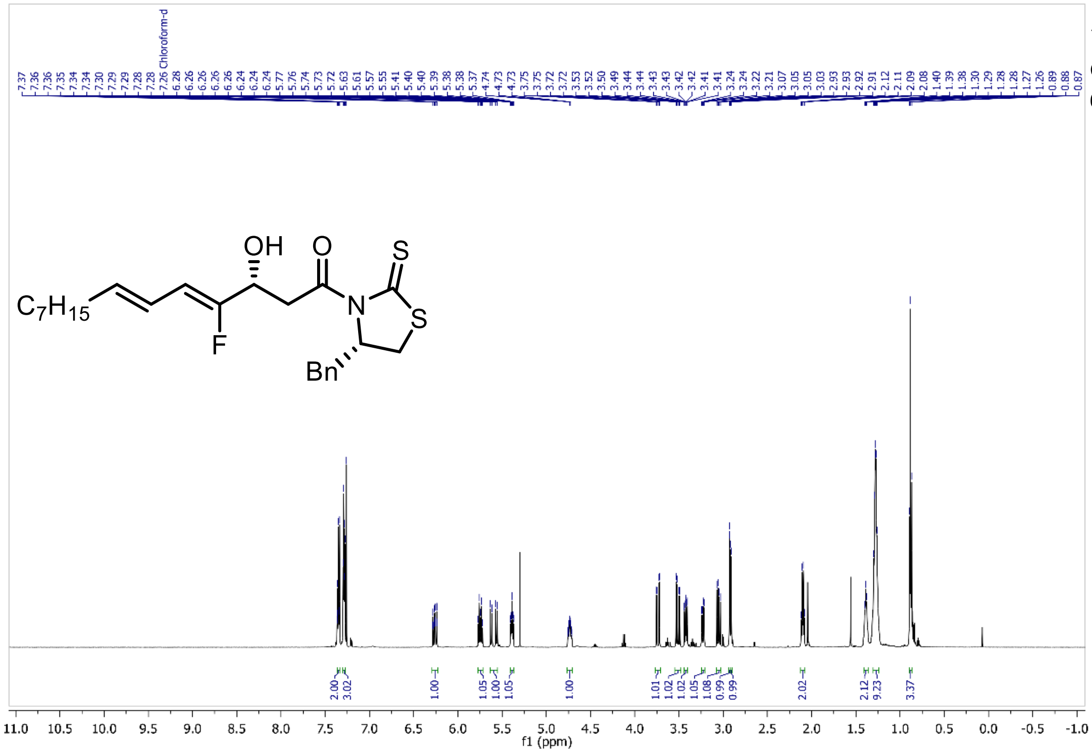


$^{1}H$
$CDCl_3$
600 MHz

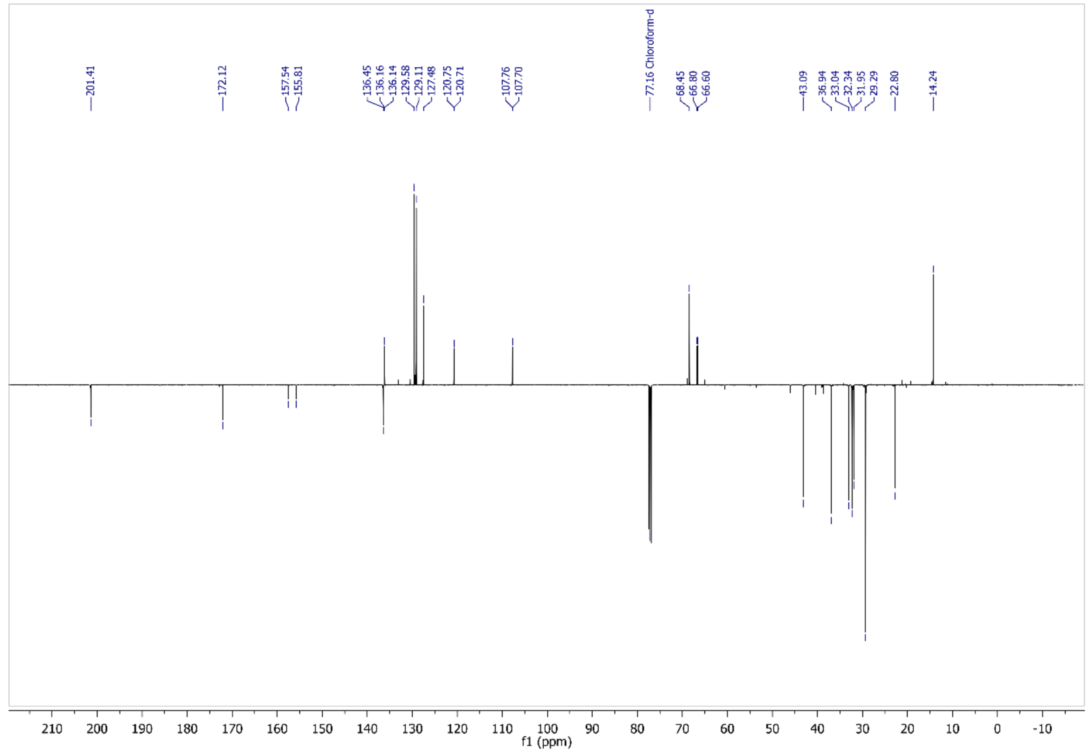


$^{13}C$
$CDCl_3$
151 MHz

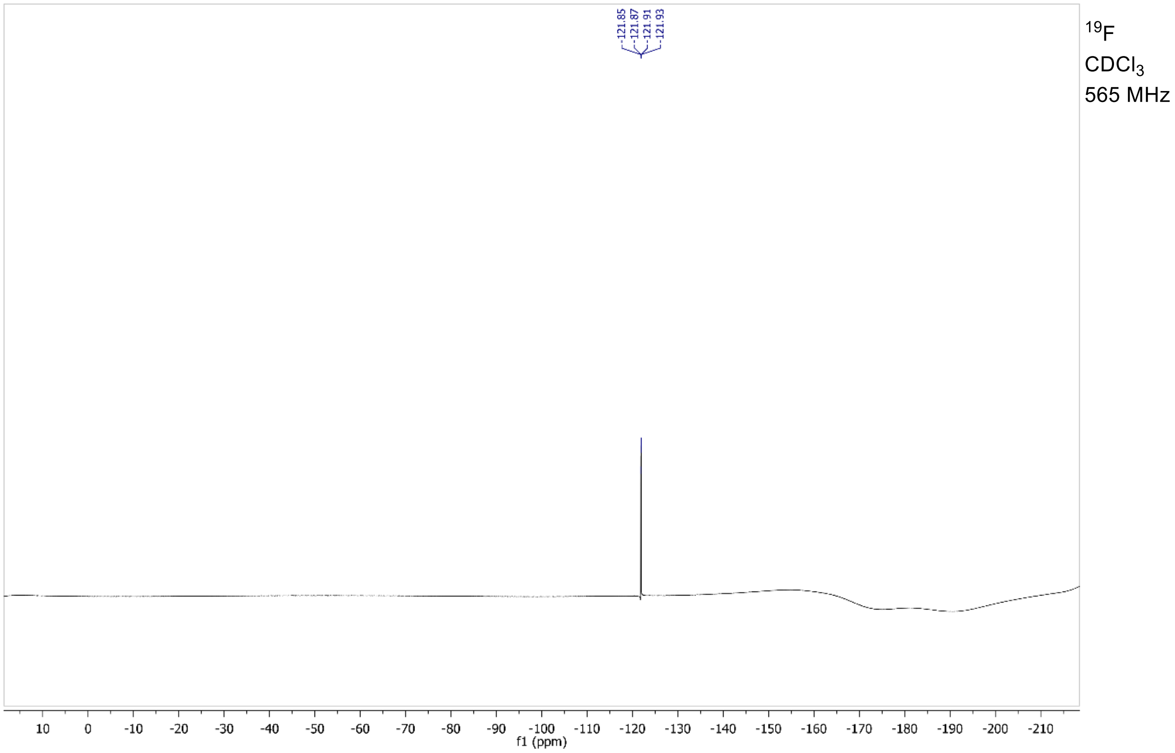


(*R*,4*Z*,6*E*)-*N*-((2*R*,3*S*)-2-((*tert*-butyldimethylsilyl)oxy)-3-methyl-4-oxo-4-(((*E*)-4-(4,4,5,5-tetramethyl-1,3,2-dioxaborolan-2-yl)but-3-en-1-yl)amino)butyl)-4-fluoro-3-hydroxytetradeca-4,6-dienamide (5)

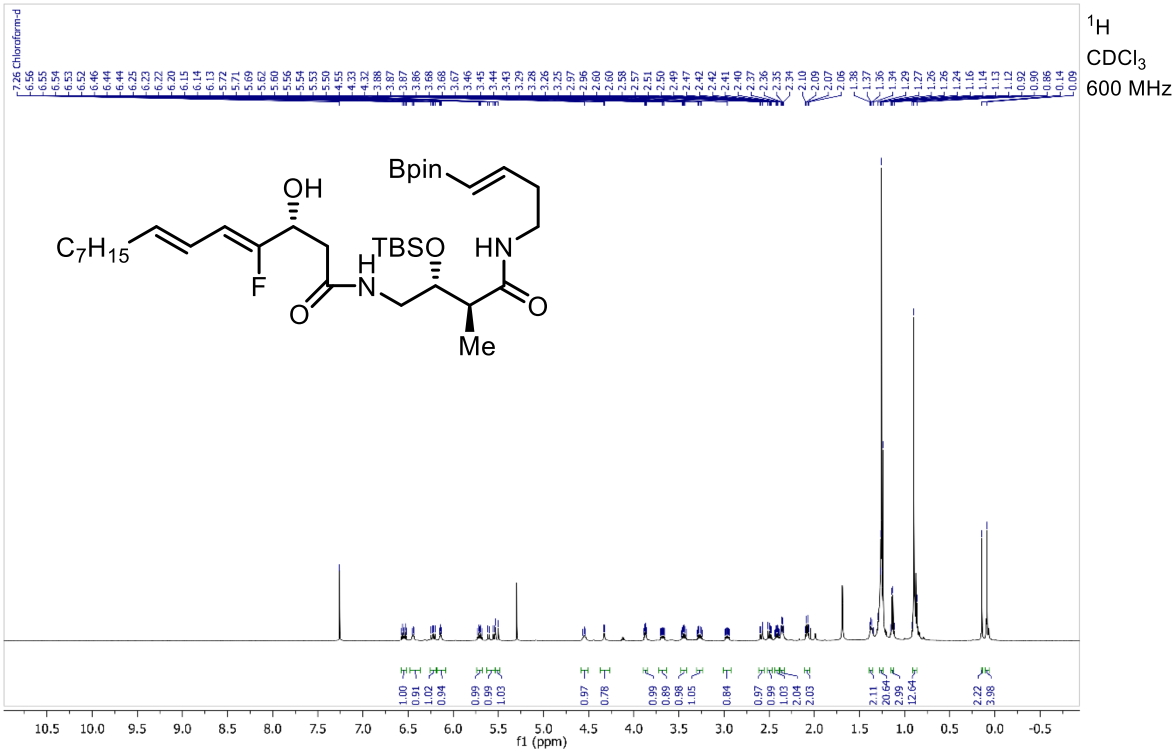

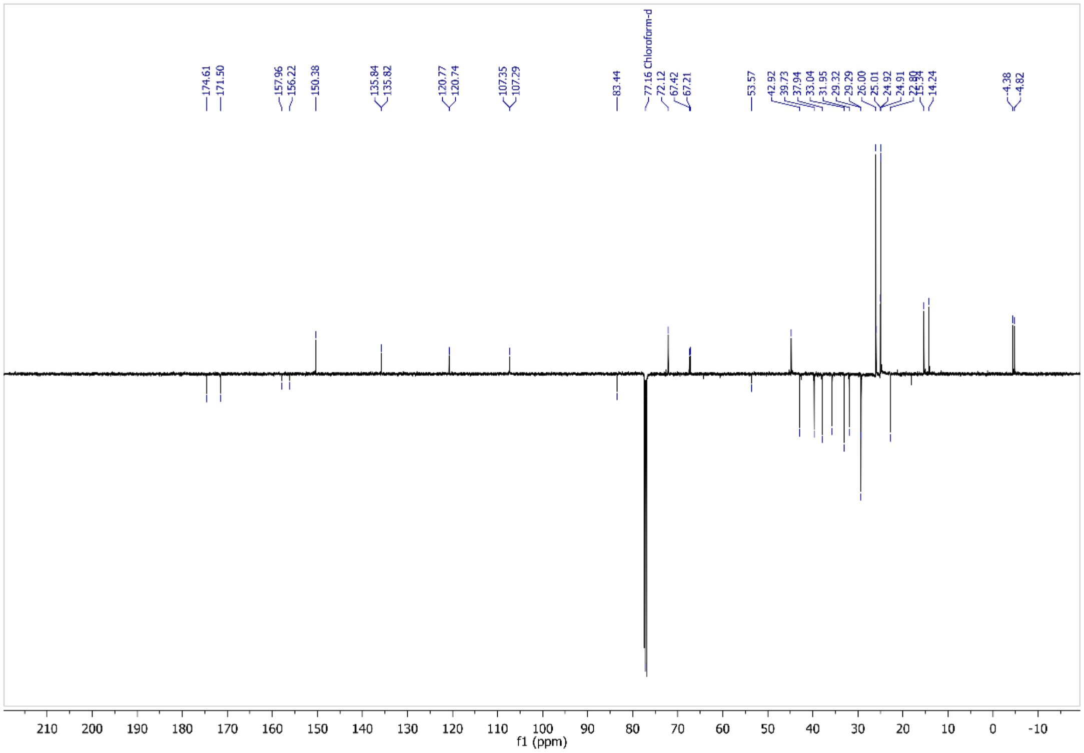


$^{13}C$
$CDCl_3$
151 MHz

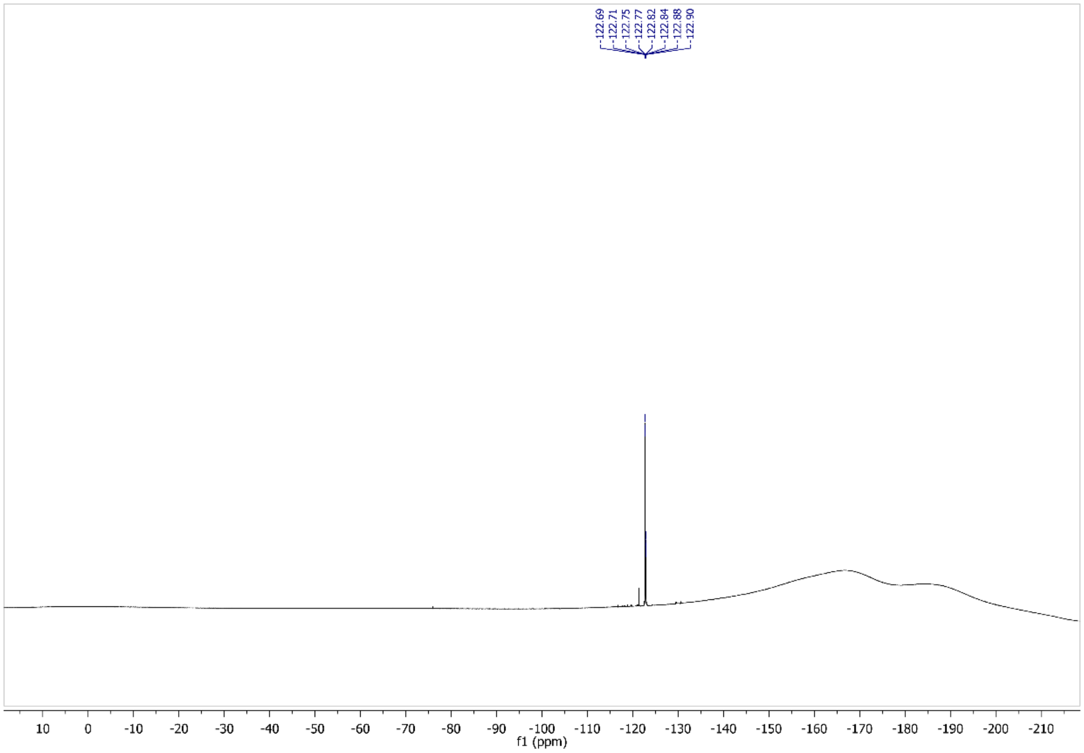


$^{19}F$
$CDCl_3$
565 MHz

(*R*,4*Z*,6*E*)-1-(((2*R*,3*S*)-2-((tert-butyldimethylsilyl)oxy)-3-methyl-4-oxo-4-(((*E*)-4-(4,4,5,5-tetramethyl-1,3,2-dioxaborolan-2-yl)but-3-en-1-yl)amino)butyl)amino)-4-fluoro-1-oxotetradeca-4,6-dien-3-yl (1*S*,4*S*)-4-chlorocyclobut-2-ene-1-carboxylate (7)

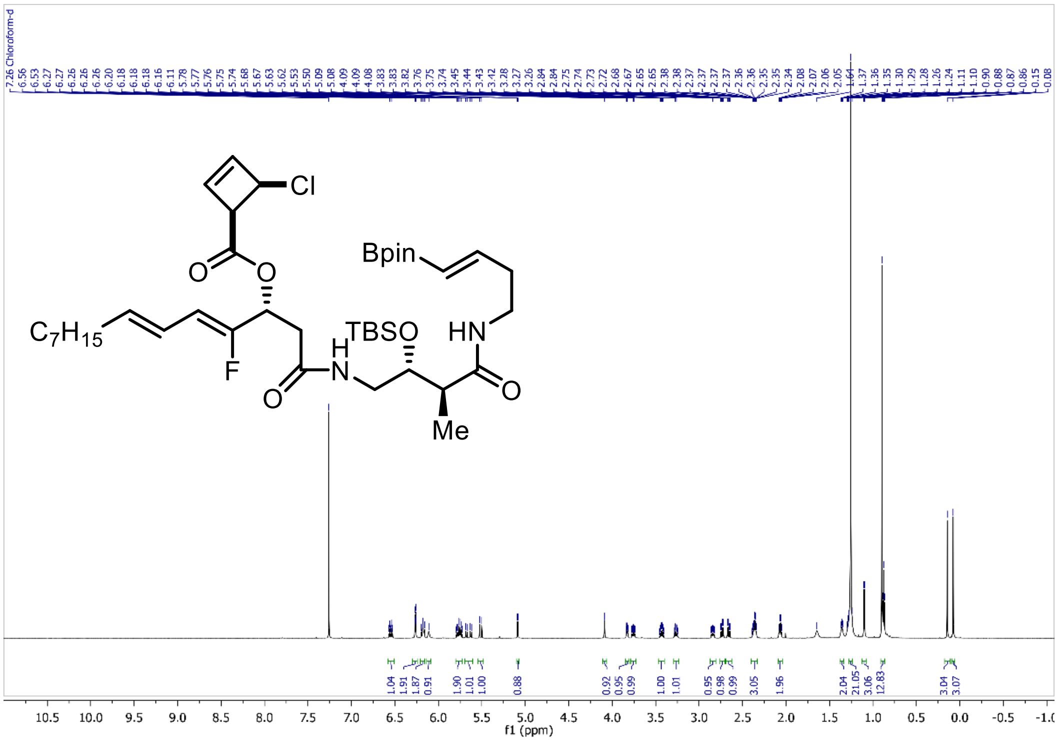


$^1H$
$CDCl_3$
600 MHz

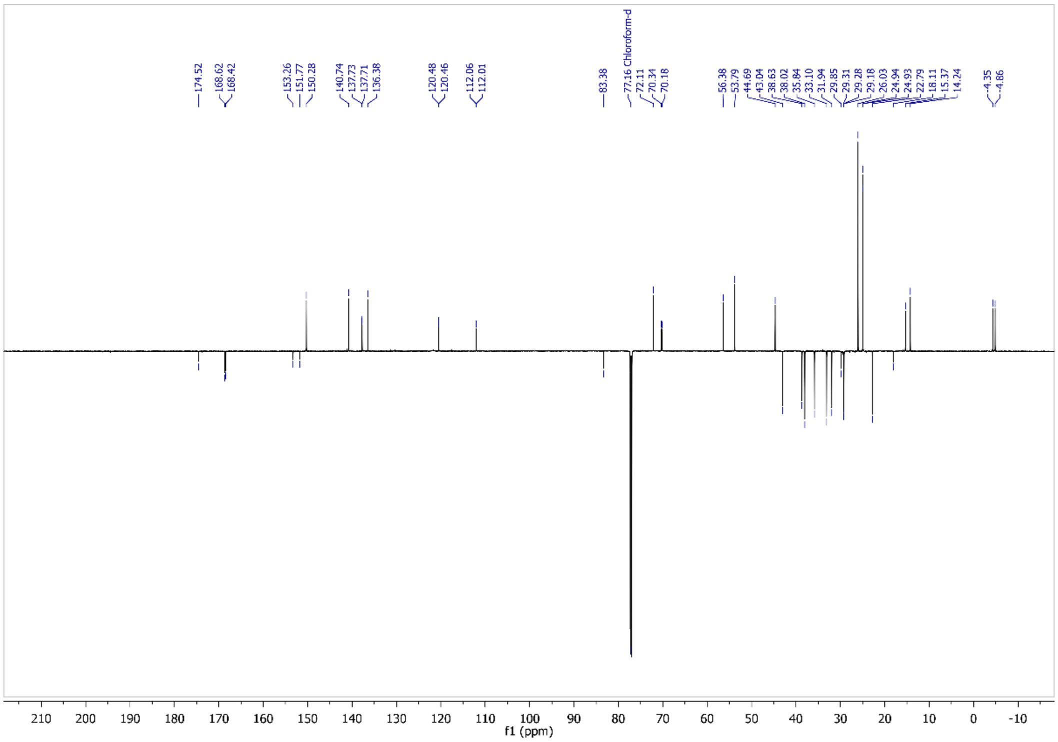


$^{13}C$
$CDCl_3$
151 MHz

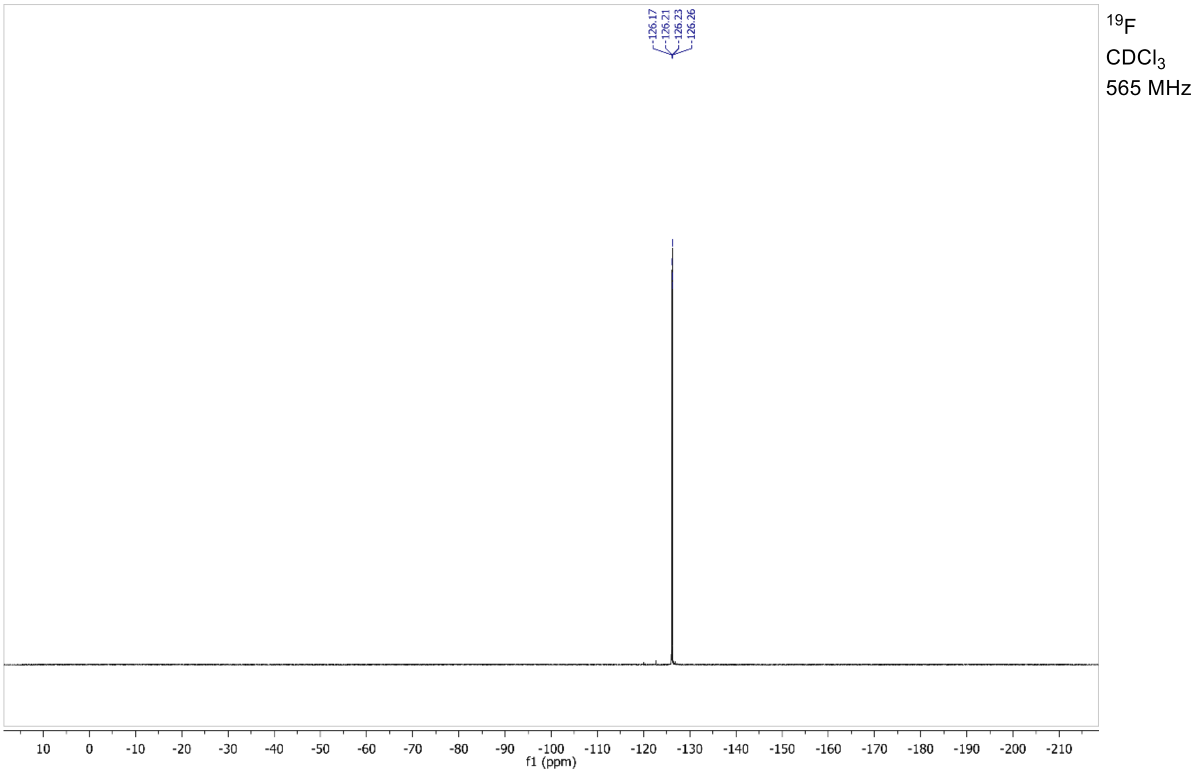


(2R,7R,8S,13E,15E,17E)-7-((tert-butyldimethylsilyl)oxy)-2-((1Z,3E)-1-fluoroundeca-1,3-dien-1-yl)-8-methyl-1-oxa-5,10-diazacyclononadeca-13,15,17-triene-4,9,19-trione (8)

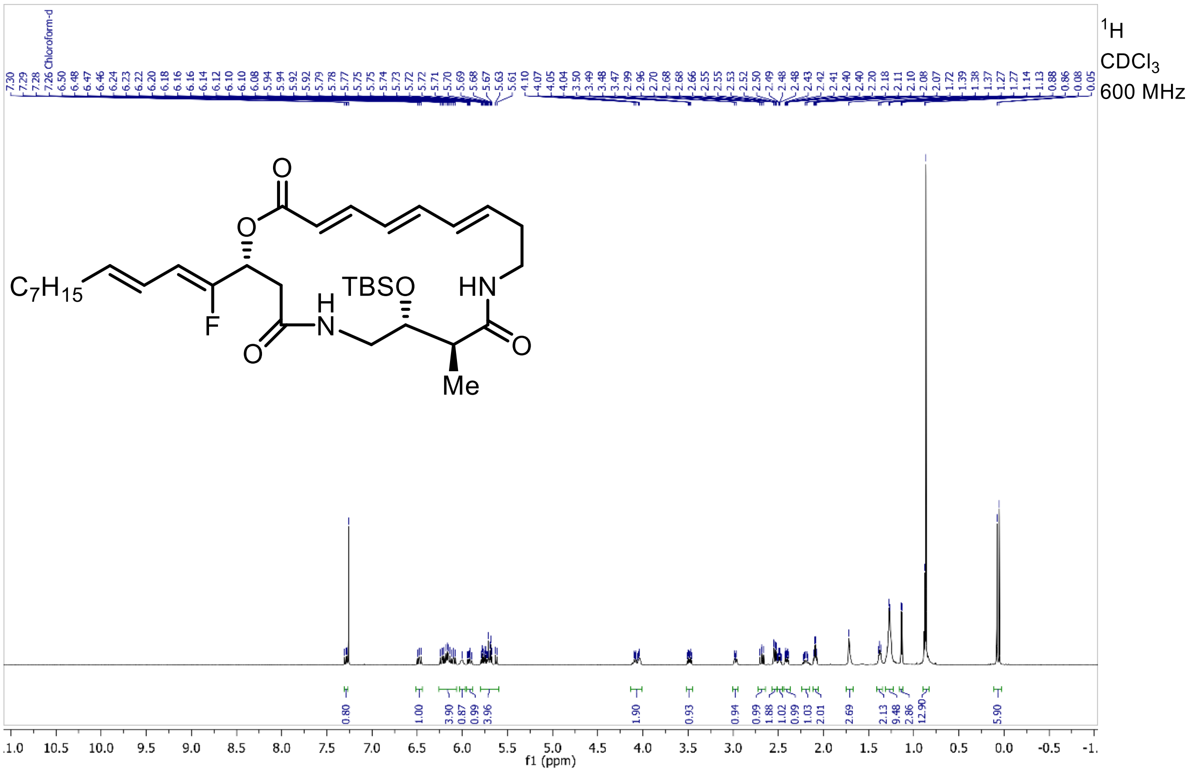

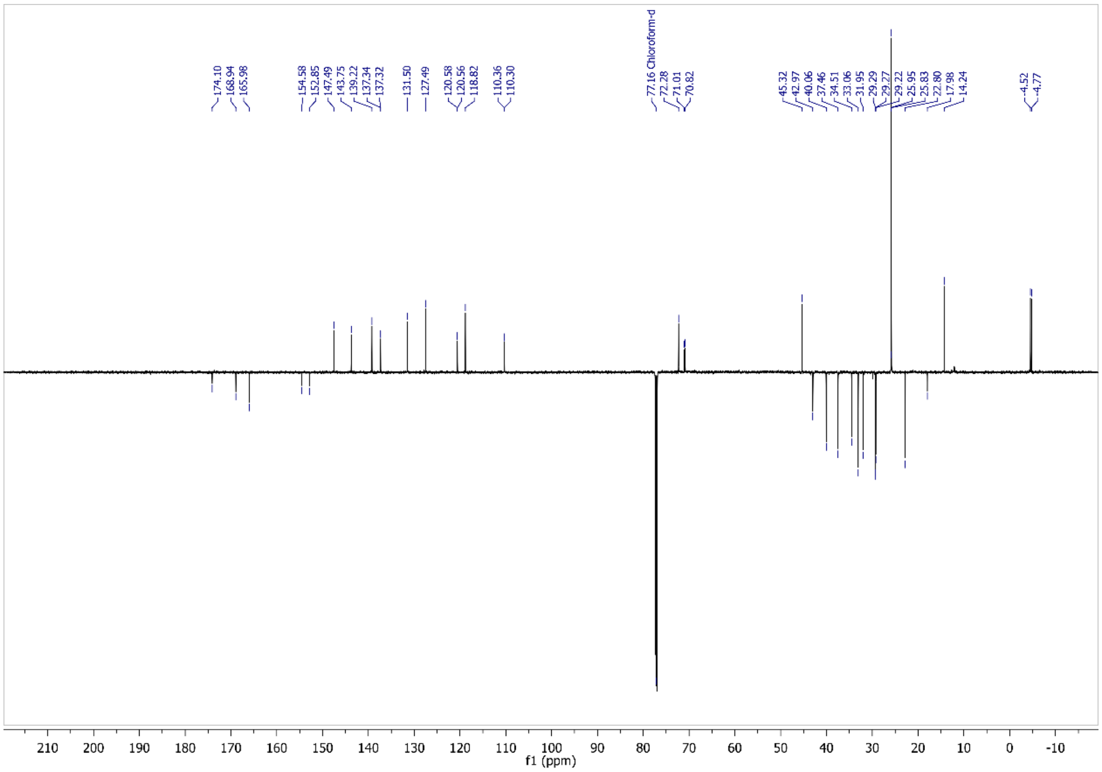


$^{13}C$
$CDCl_3$
151 MHz

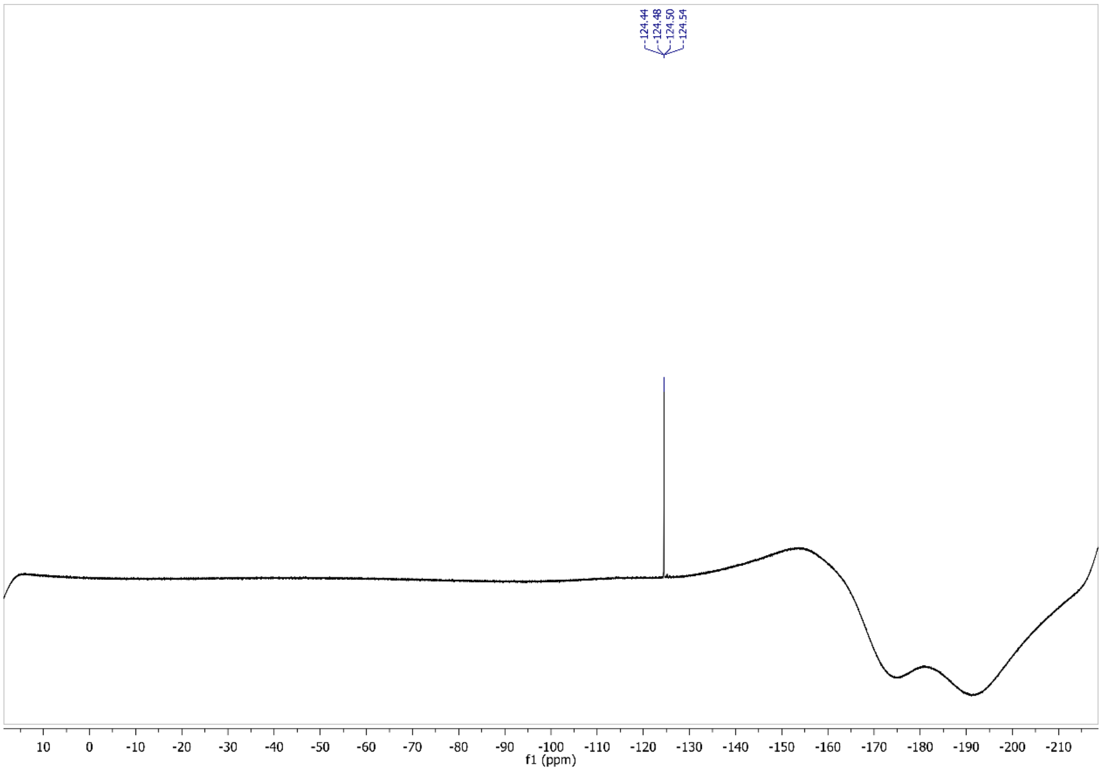


$^{19}F$
$CDCl_3$
565 MHz

fs-FR4

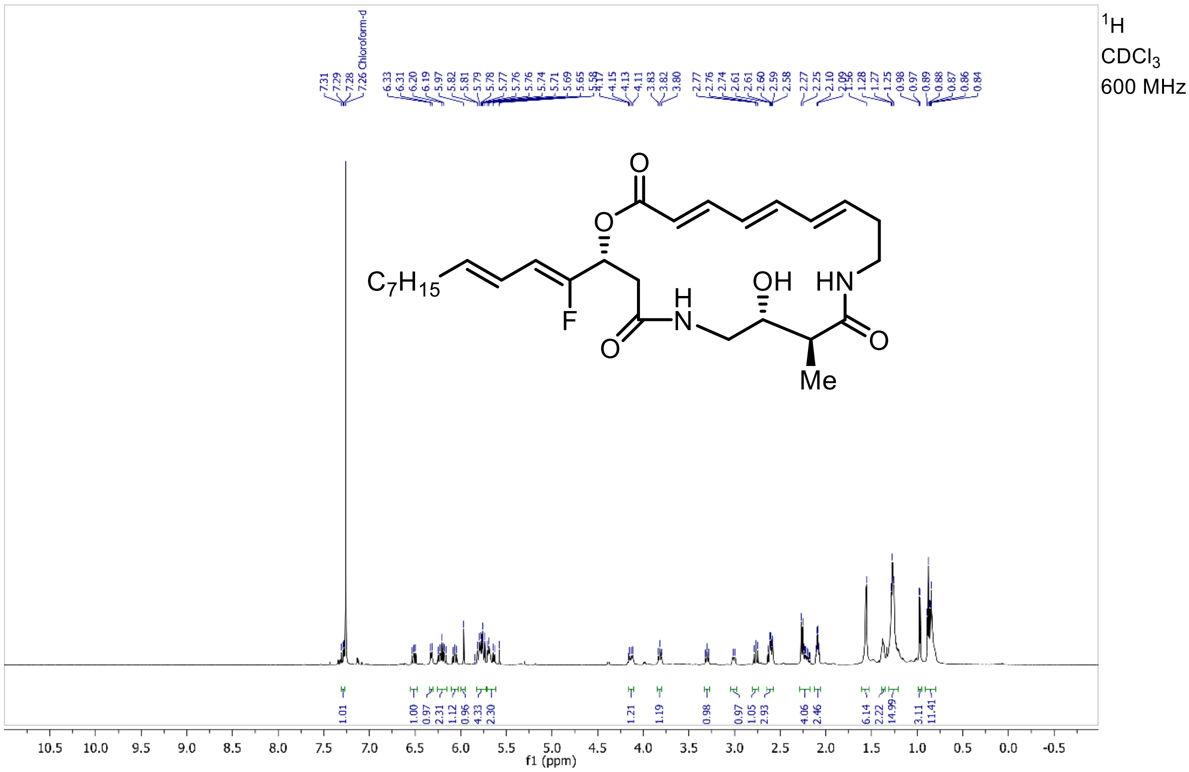


$^{1}H$
$CDCl_3$
600 MHz

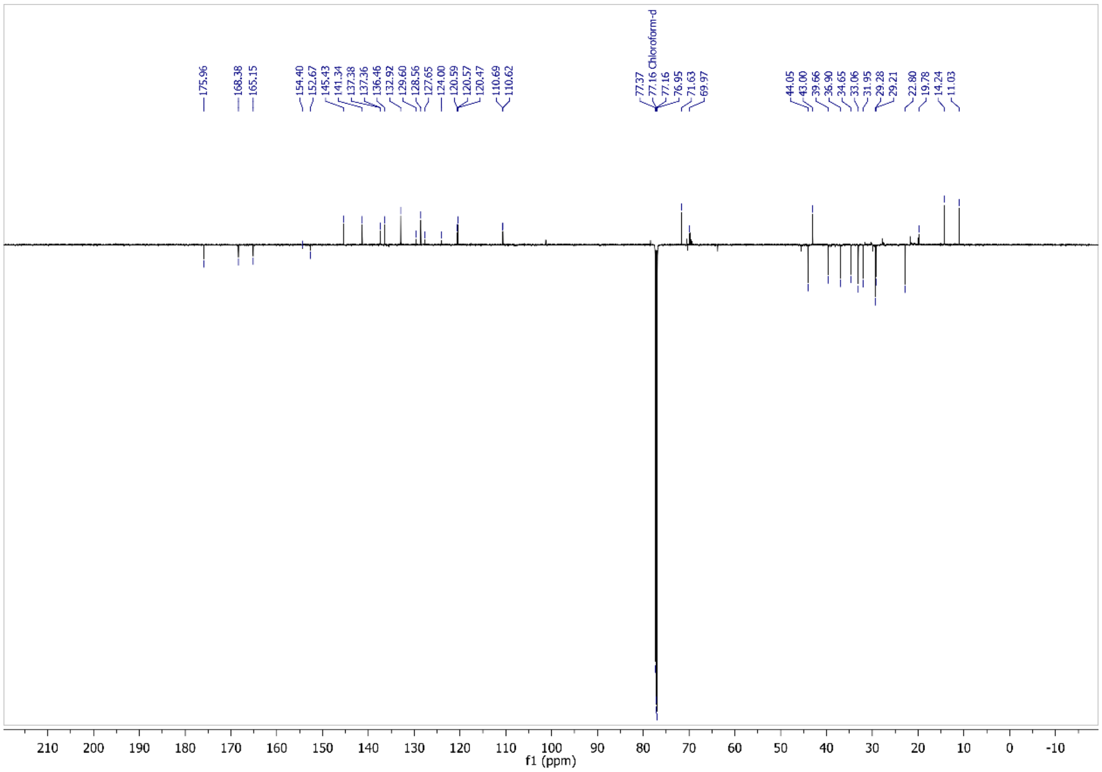


$^{13}C$
$CDCl_3$
151 MHz

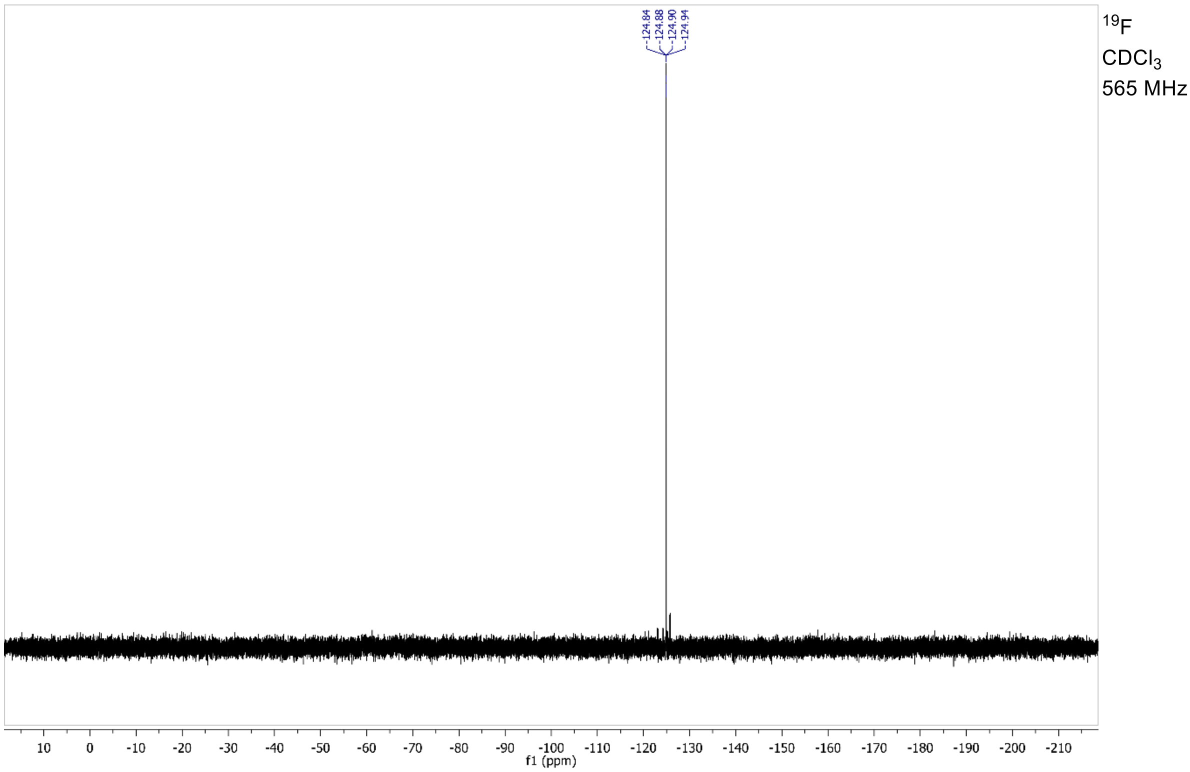


$^{19}F$
$CDCl_3$
565 MHz

ethyl (2Z,4E)-2-(trifluoromethyl)dodeca-2,4-dienoate (S-66)

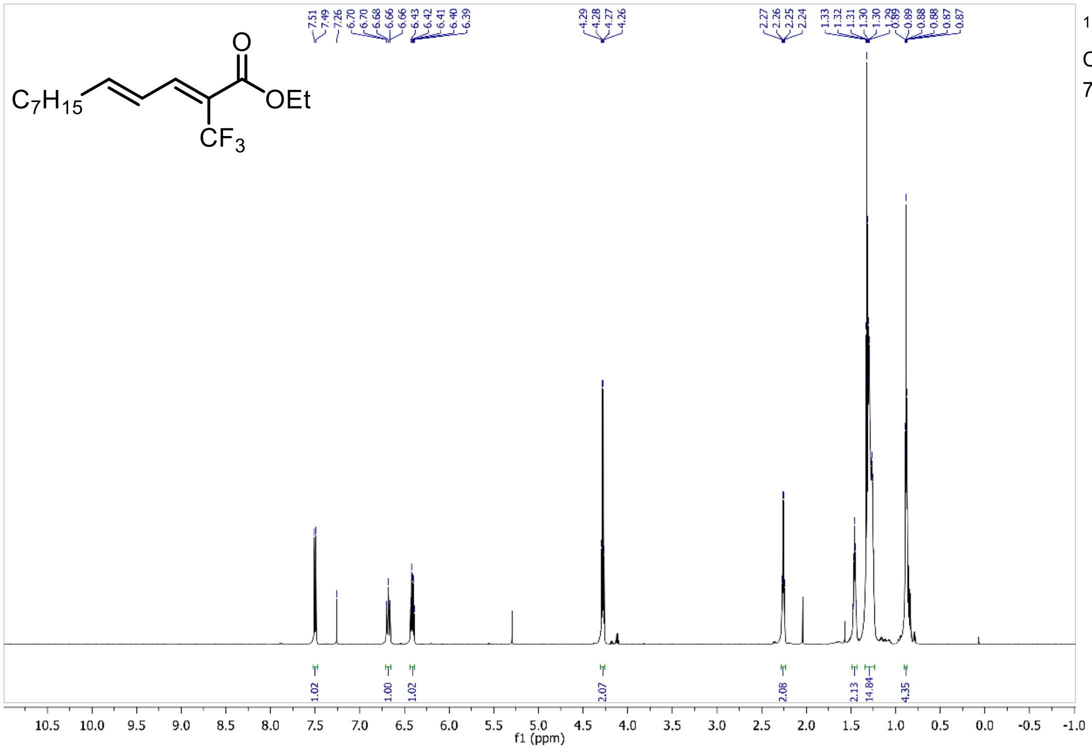


$^{1}H$
$CDCl_3$
700 MHz

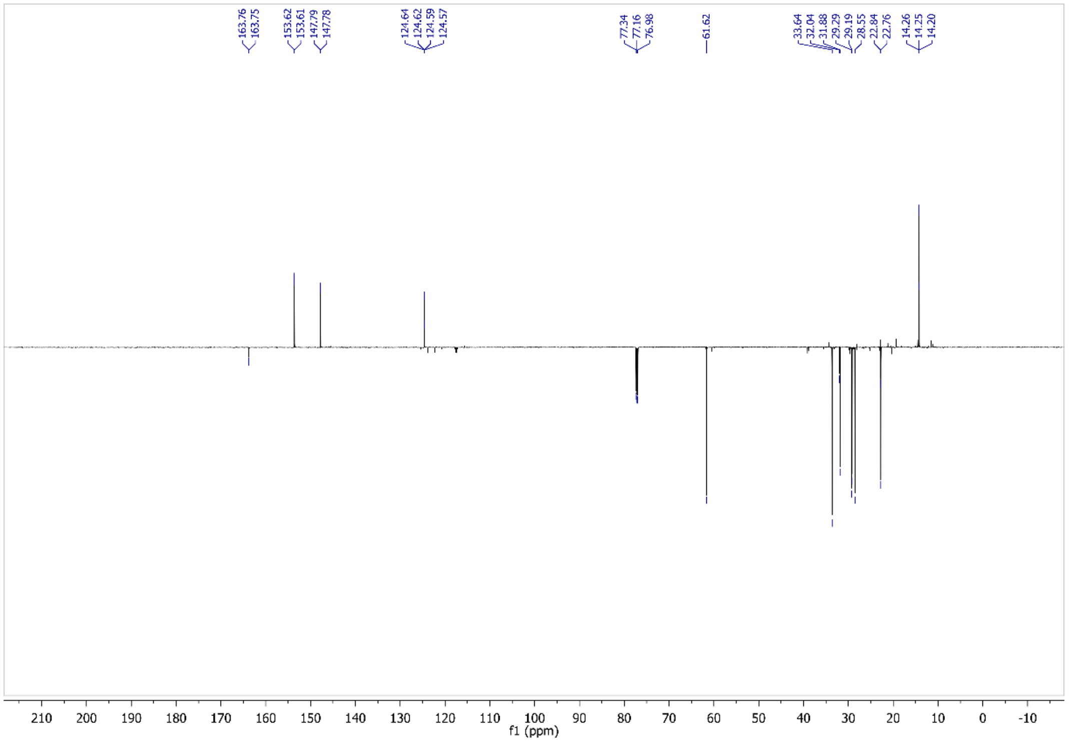


$^{13}C$
$CDCl_3$
176 MHz

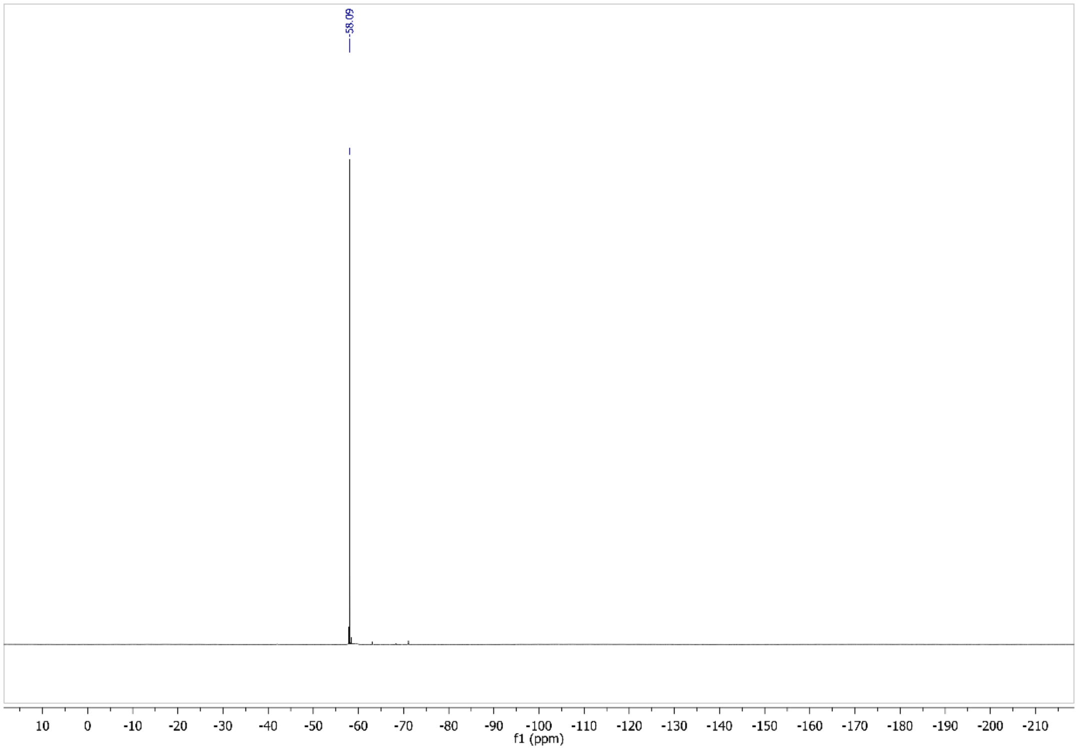


$^{19}F$
$CDCl_3$
658 MHz

## (2*Z*,4*E*)-2-(trifluoromethyl)dodeca-2,4-dienal (S-68)

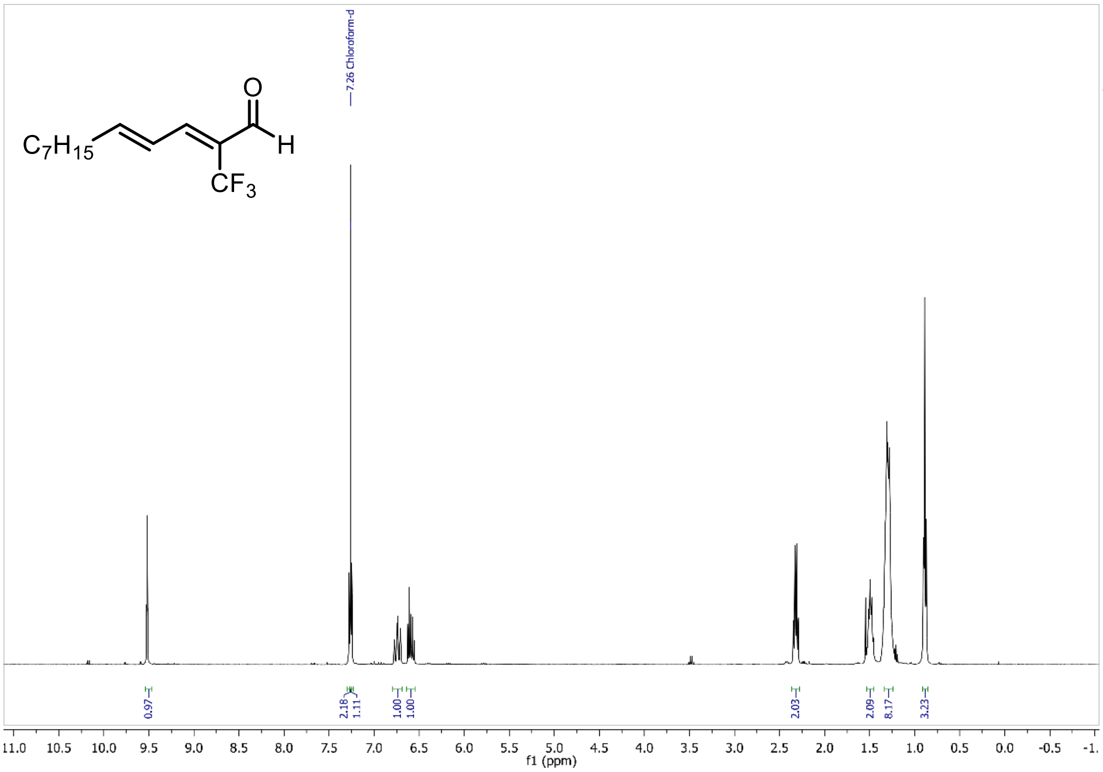


$^{1}H$
$CDCl_3$
400 MHz

## (*R*,4*Z*,6*E*)-1-((*S*)-4-benzyl-2-thioxothiazolidin-3-yl)-3-hydroxy-4-(trifluoromethyl)tetradeca-4,6-dien-1-one (S-69)

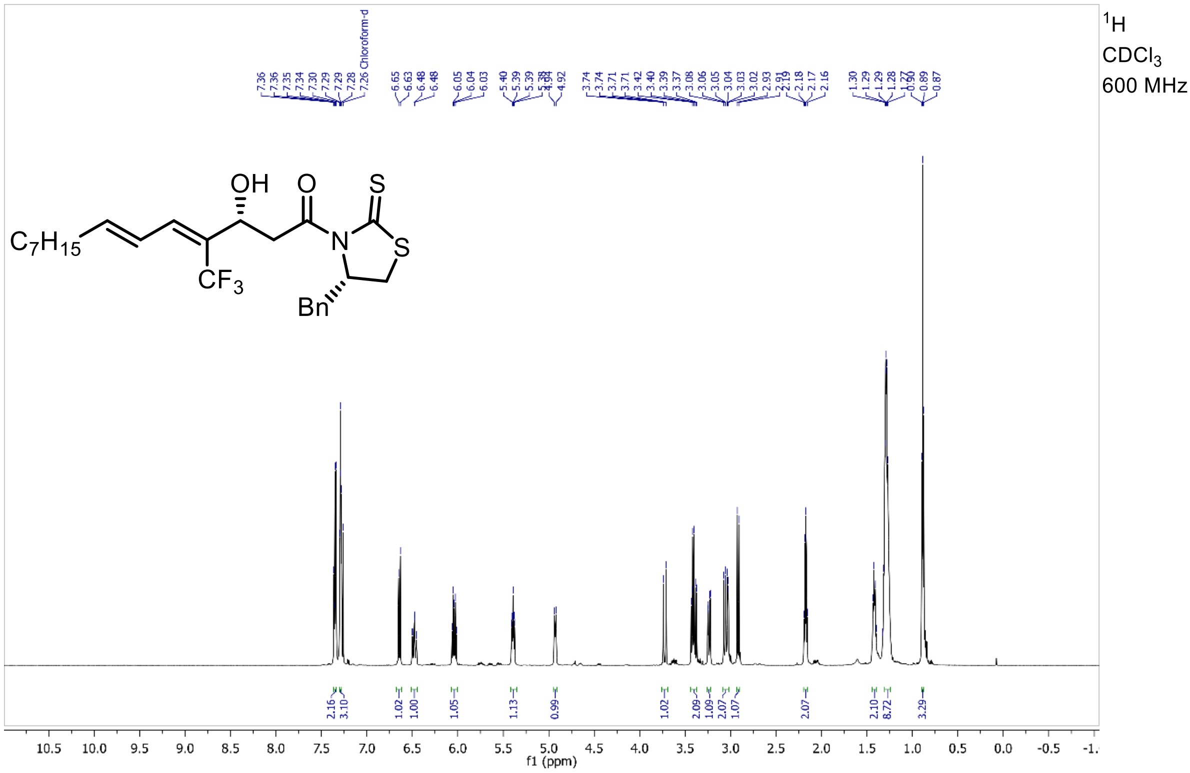


$^1H$
$CDCl_3$
600 MHz

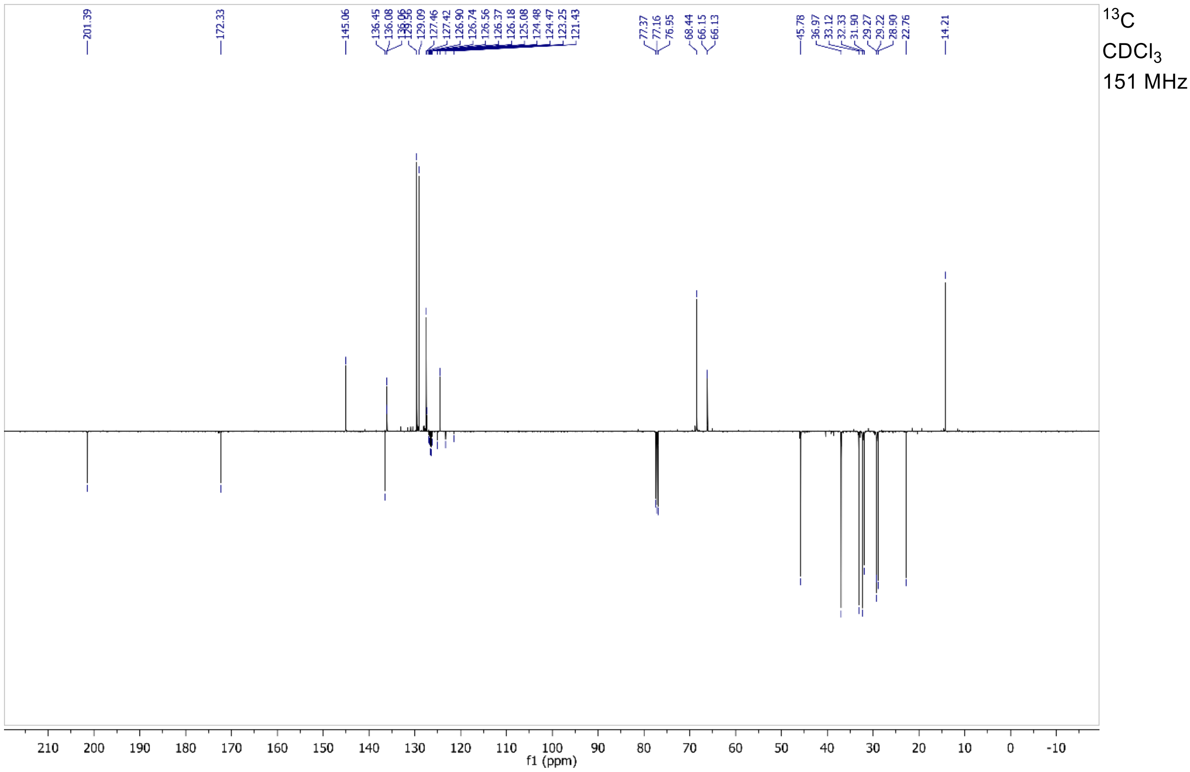


$^{13}C$
$CDCl_3$
151 MHz

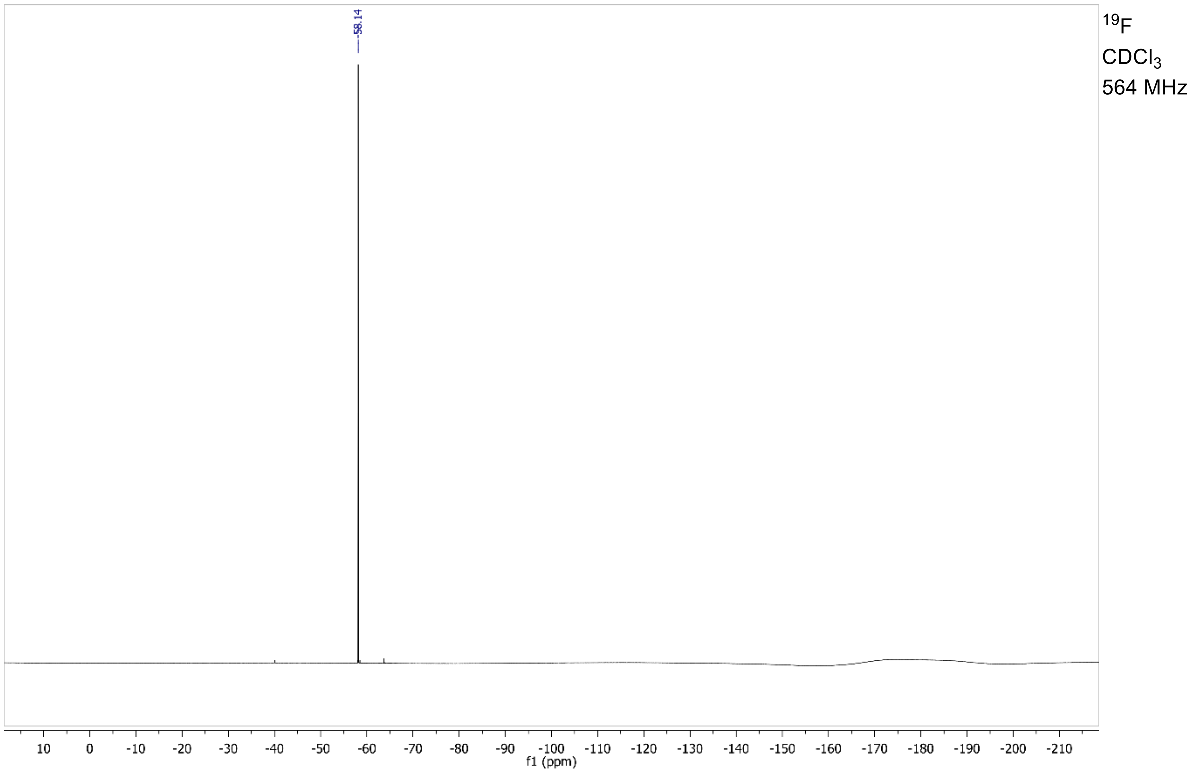


$^{19}F$
$CDCl_3$
564 MHz

(*R*,4*Z*,6*E*)-*N*-((2*R*,3*S*)-2-((*tert*-butyldimethylsilyl)oxy)-3-methyl-4-oxo-4-(((*E*)-4-(4,4,5,5-tetramethyl-1,3,2-dioxaborolan-2-yl)but-3-en-1-yl)amino)butyl)-3-hydroxy-4-(trifluoromethyl)tetradeca-4,6-dienamide (S-70)

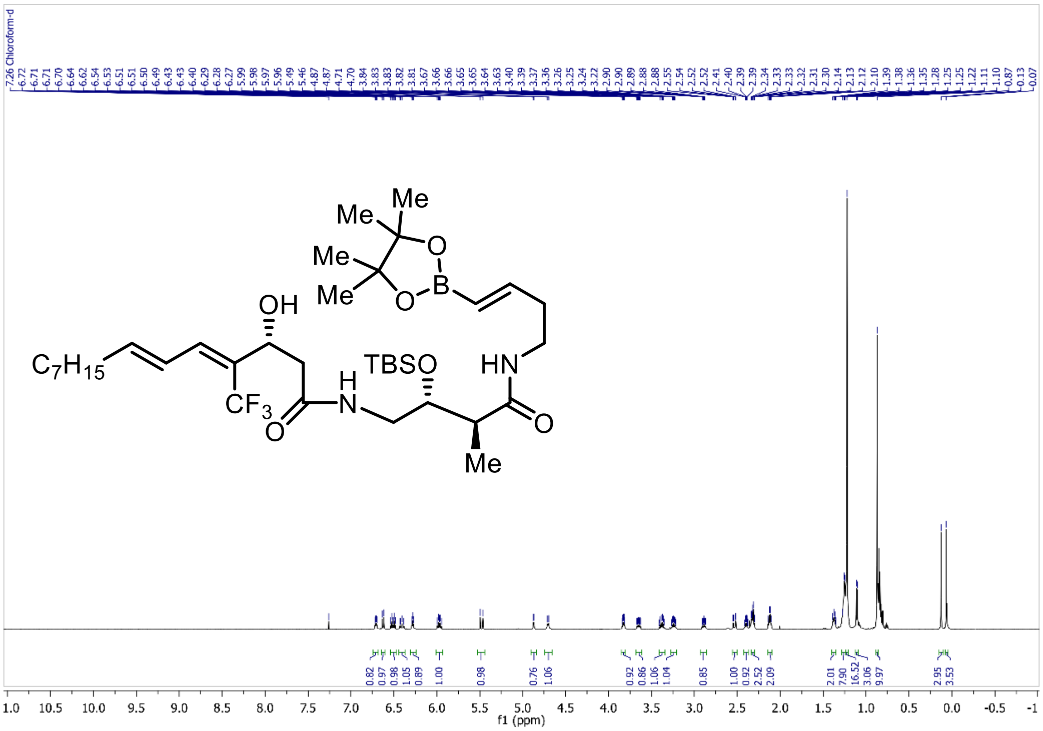


$^1H$
$CDCl_3$
600 MHz

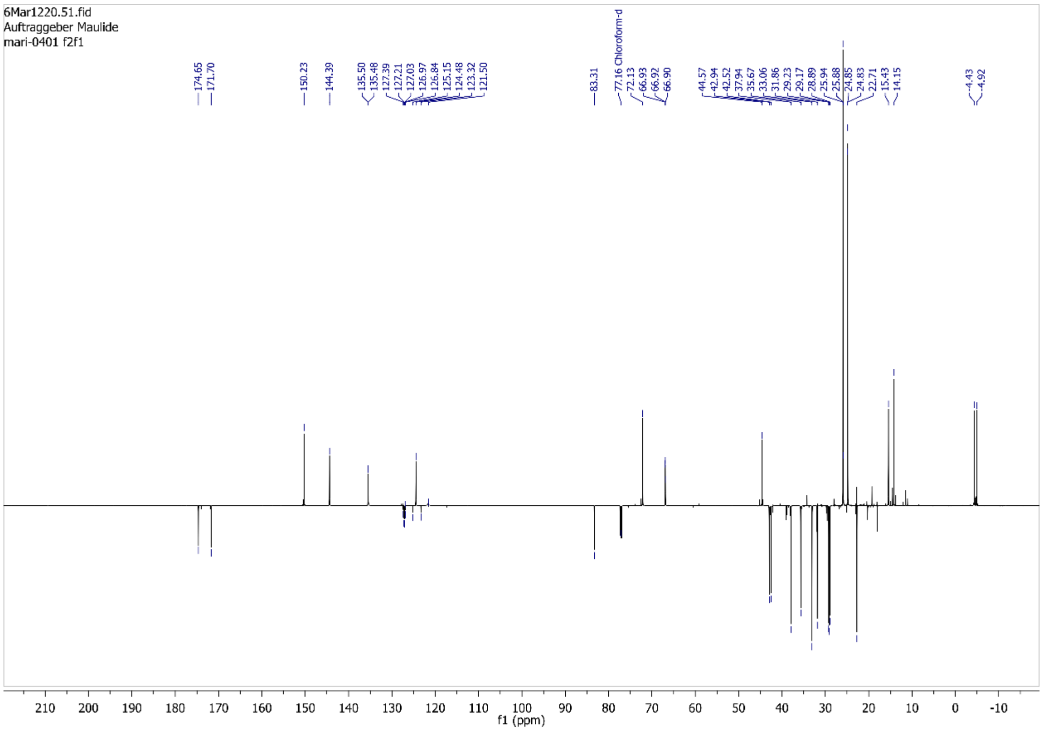


$^{13}C$
$CDCl_3$
151 MHz

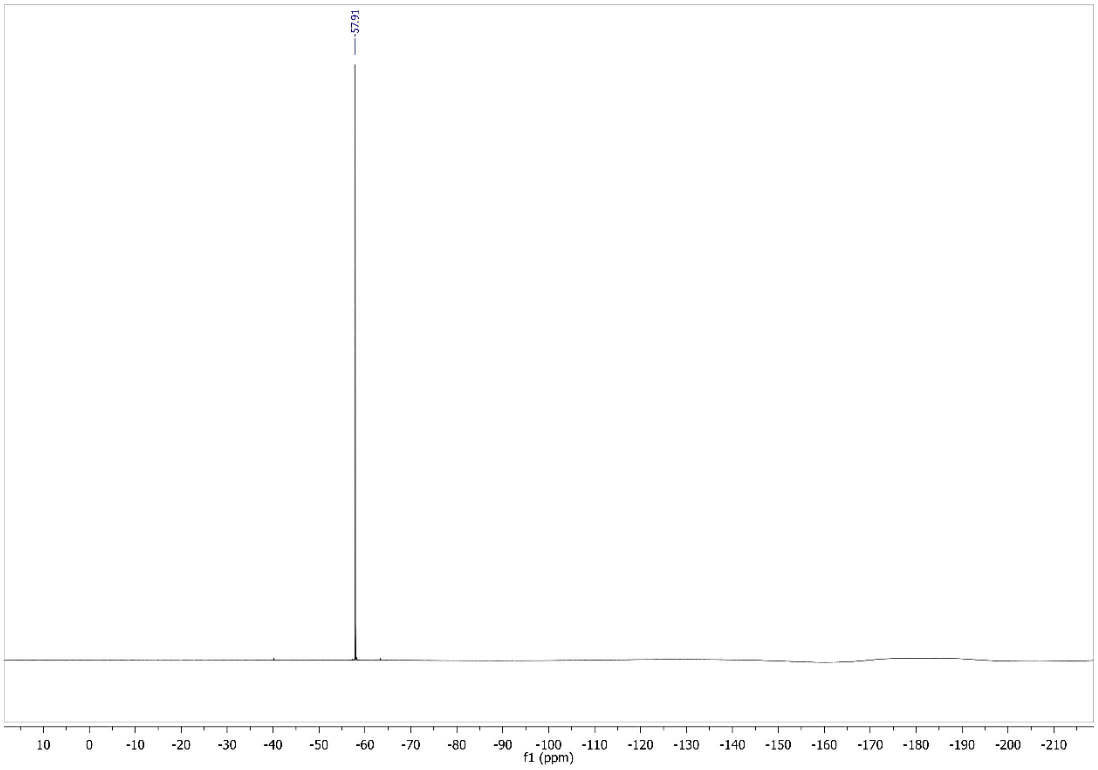


$^{19}F$
$CDCl_3$
565 MHz

(*R*,4*Z*,6*E*)-1-(((2*R*,3*S*)-2-((*tert*-butyldimethylsilyl)oxy)-3-methyl-4-oxo-4-(((*E*)-4-(4,4,5,5-tetramethyl-1,3,2-dioxaborolan-2-yl)but-3-en-1-yl)amino)butyl)amino)-1-oxo-4-(trifluoromethyl)tetradeca-4,6-dien-3-yl (1*R*,4*R*)-4-chlorocyclobut-2-ene-1-carboxylate (S-71)

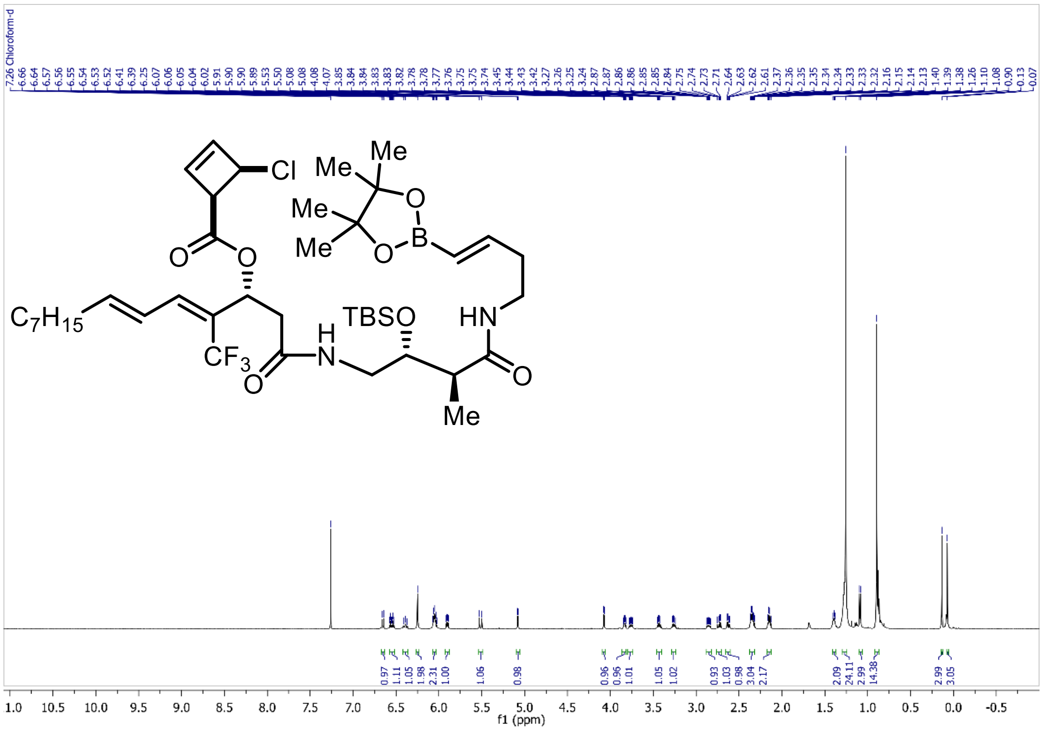


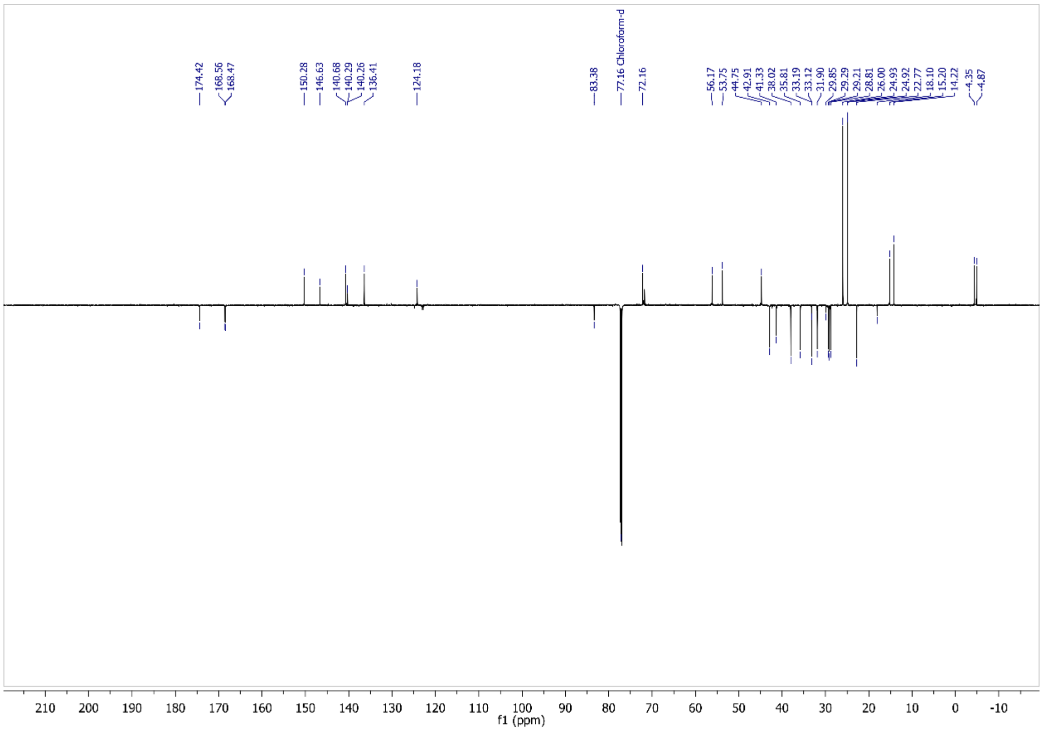

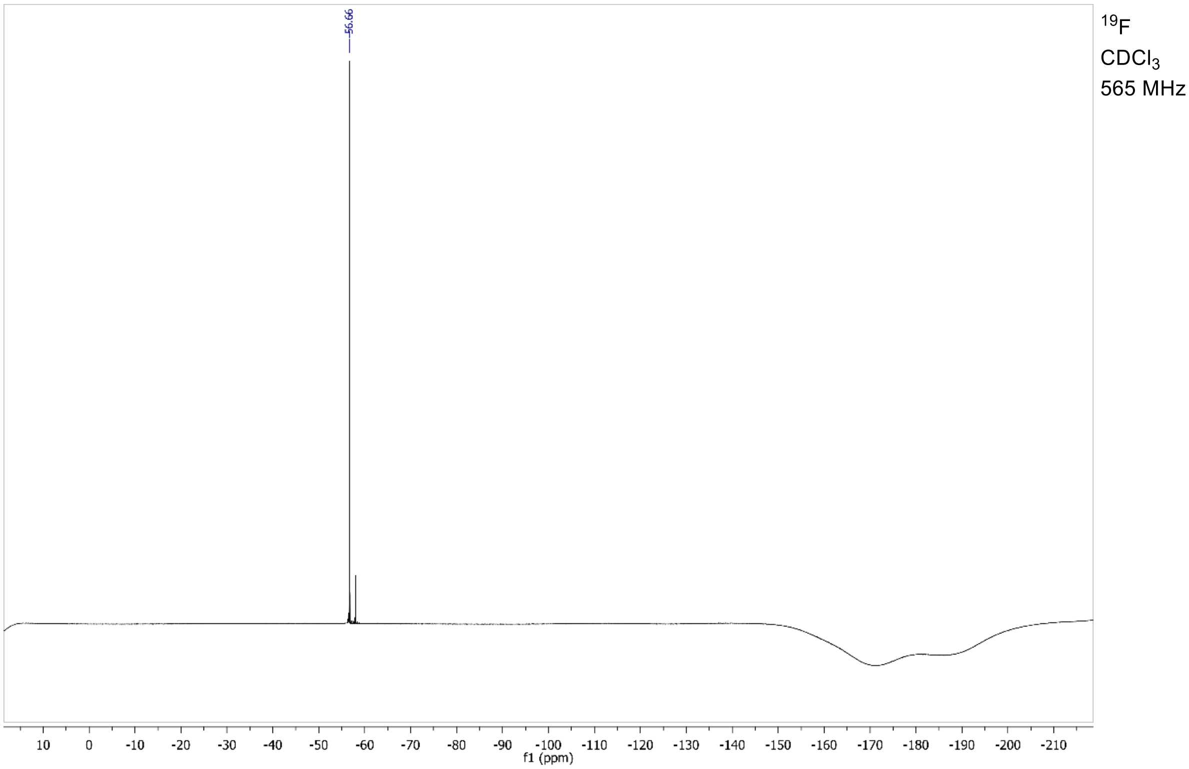


(2*R*,7*R*,8*S*,13*E*,15*E*,17*E*)-7-((*tert*-butyldimethylsilyl)oxy)-8-methyl-2-((2*Z*,4*E*)-1,1,1-trifluorododeca-2,4-dien-2-yl)-1-oxa-5,10-diazacyclononadeca-13,15,17-triene-4,9,19-trione (S-72)

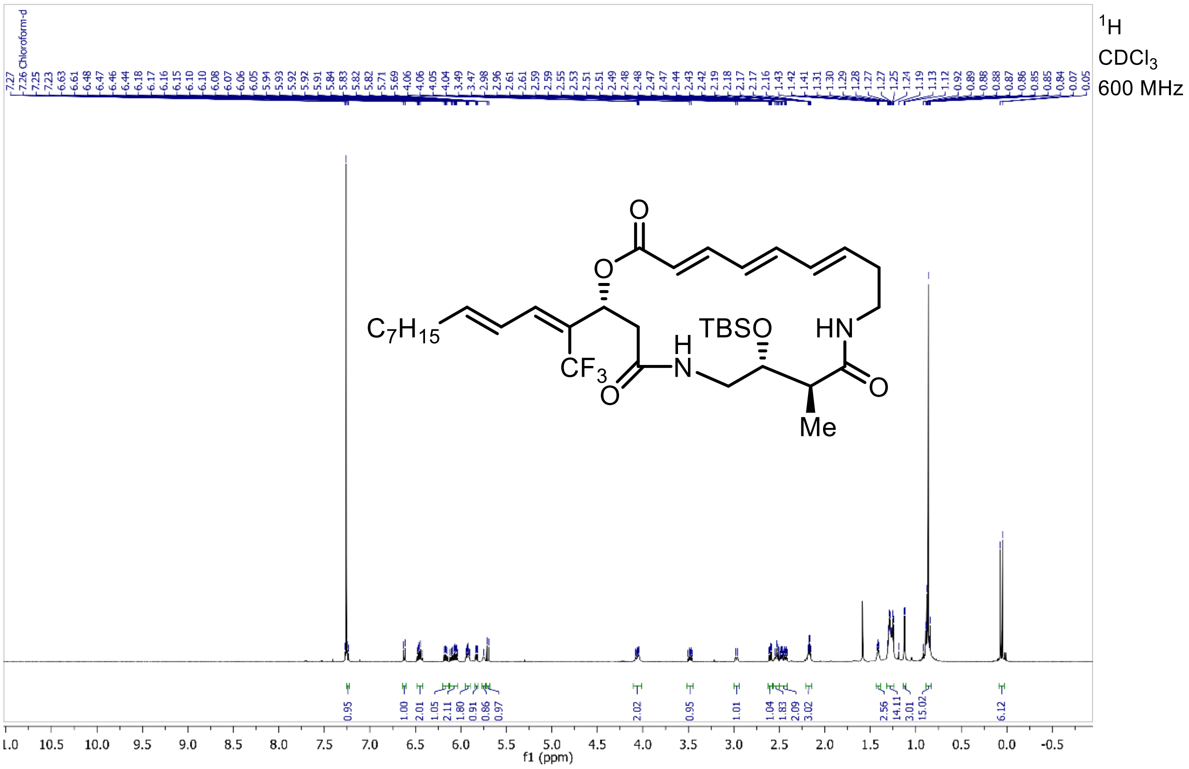

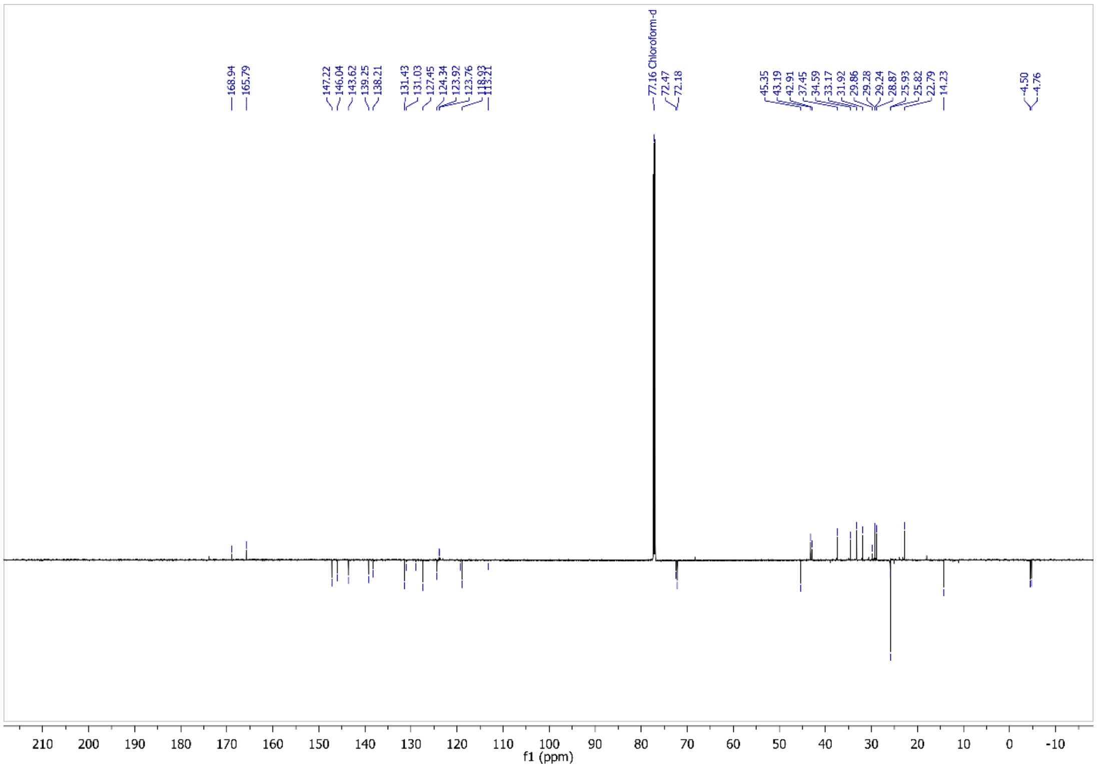


$^{13}C$
$CDCl_3$
151 MHz

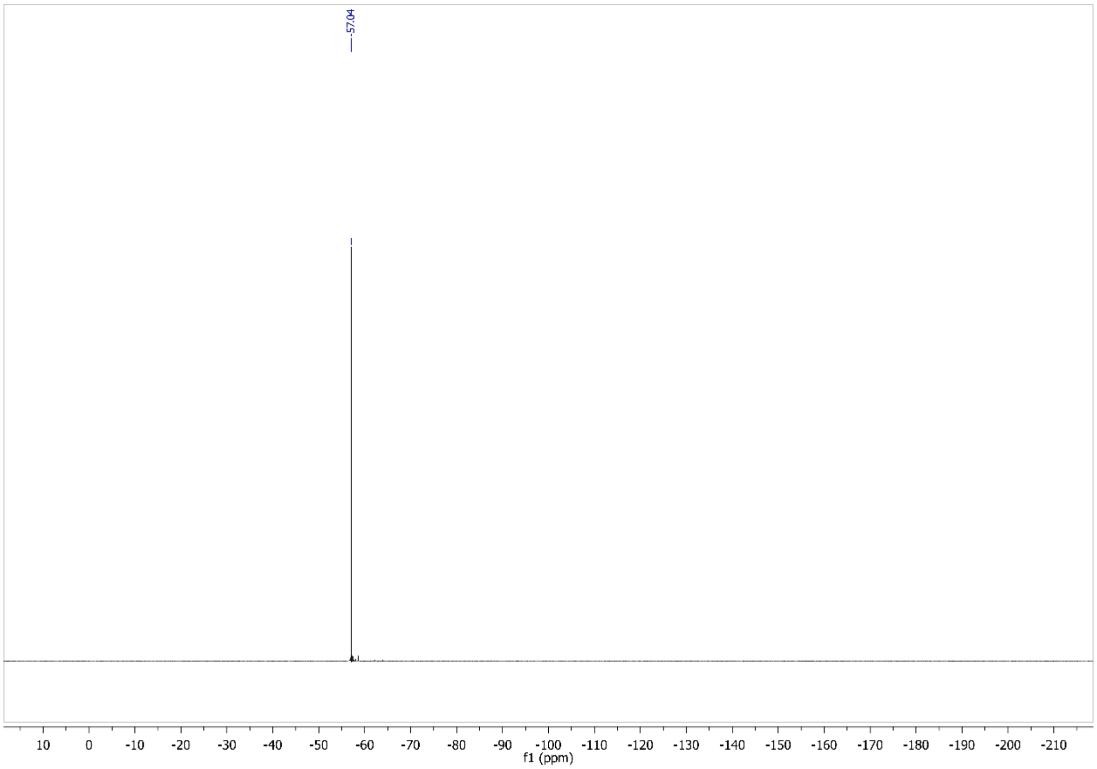


$^{19}F$
$CDCl_3$
565 MHz

## Fs-FR5

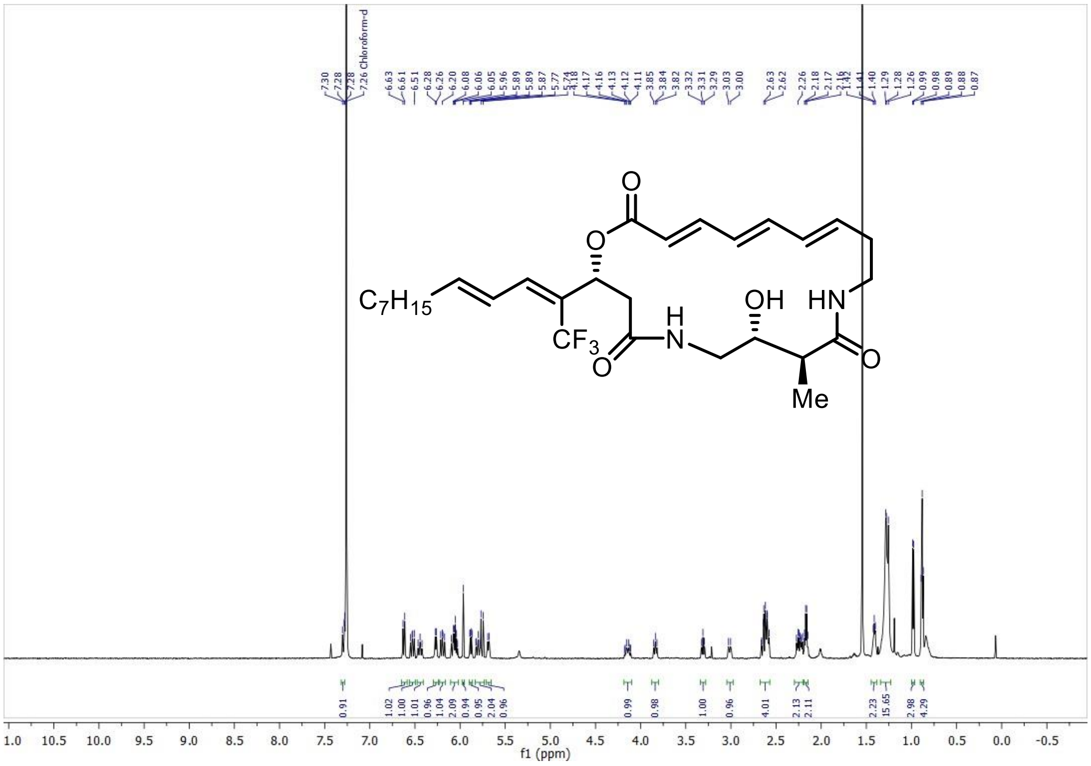


$^1H$
$CDCl_3$
600 MHz

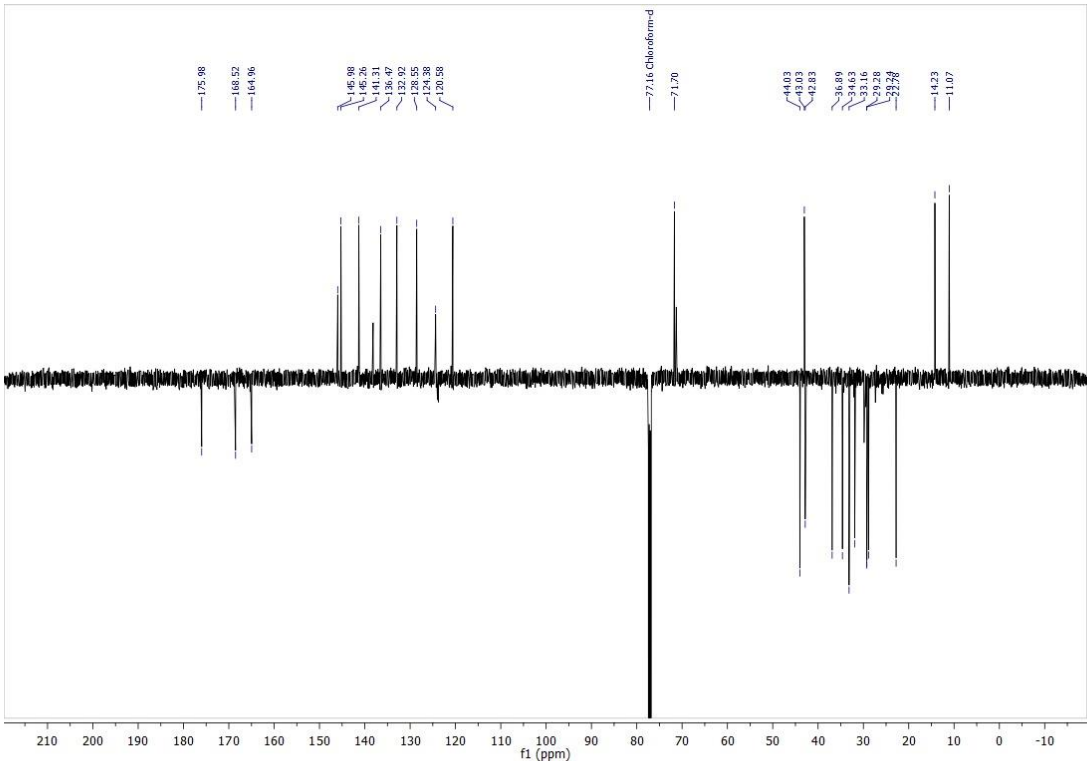


$^{13}C$
$CDCl_3$
151 MHz

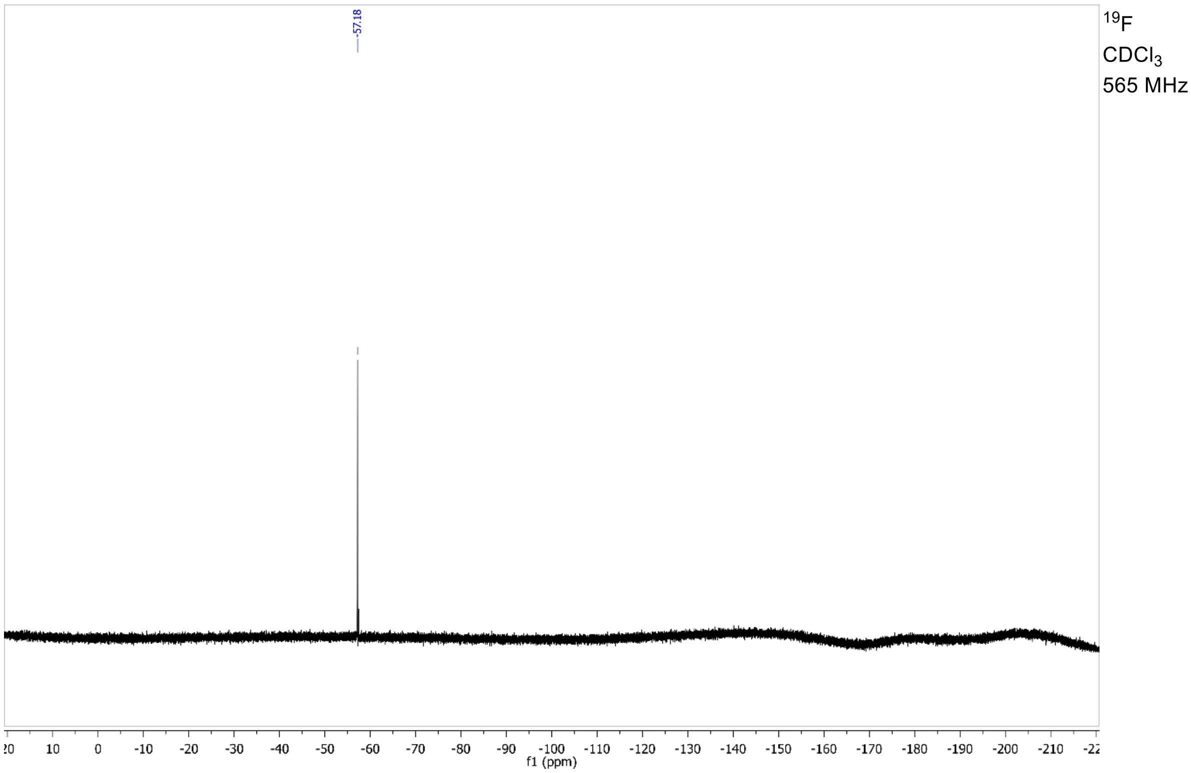


$^{19}F$
$CDCl_3$
565 MHz

## (2*E*,4*E*)-5-cyclohexyl-2-methylpenta-2,4-dien-1-ol (S-76)

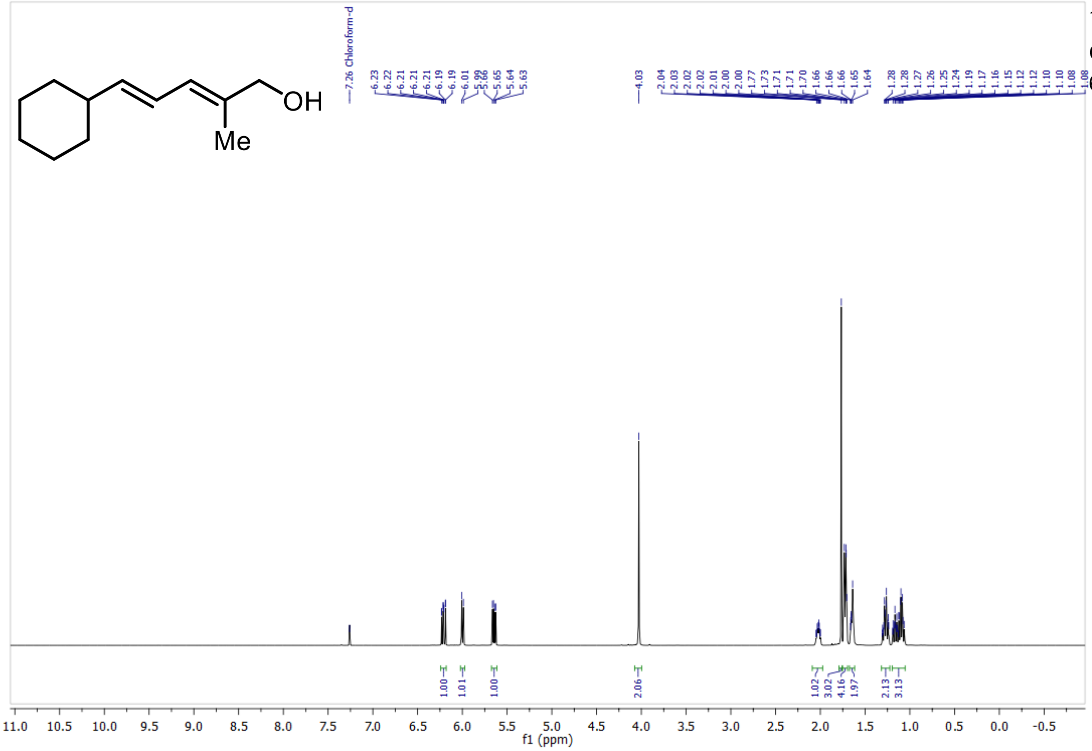


$^{1}H$
$CDCl_3$
600 MHz

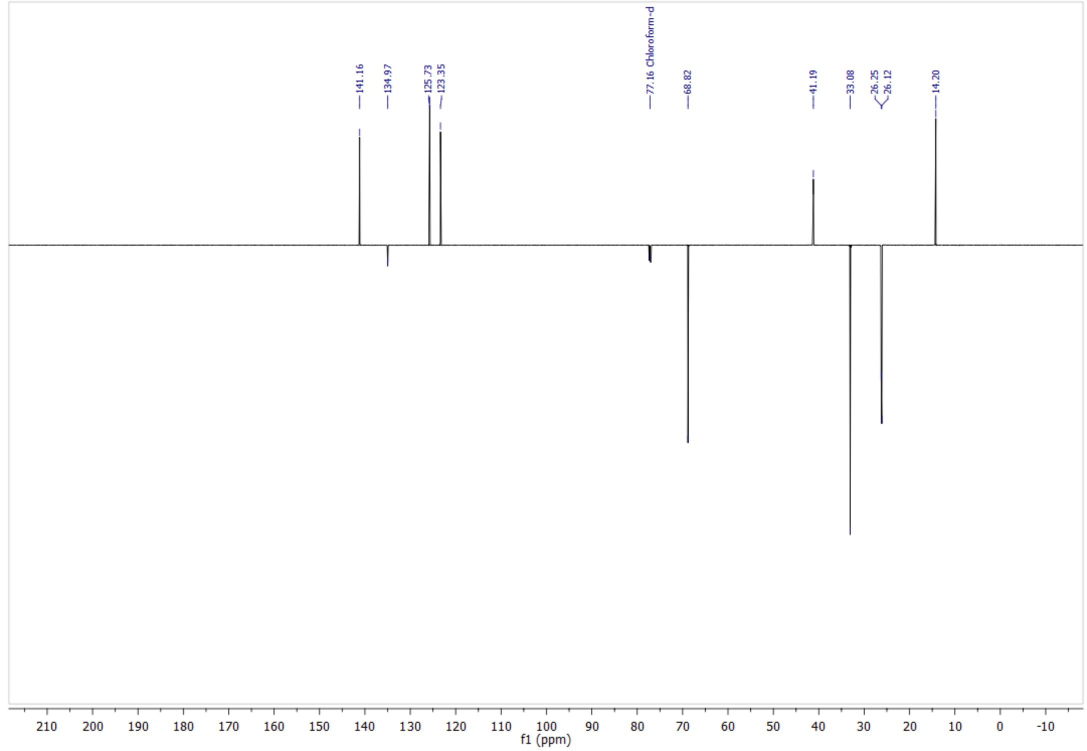


$^{13}C$
$CDCl_3$
151 MHz

## (2*E*,4*E*)-5-cyclohexyl-2-methylpenta-2,4-dienal (S-77)

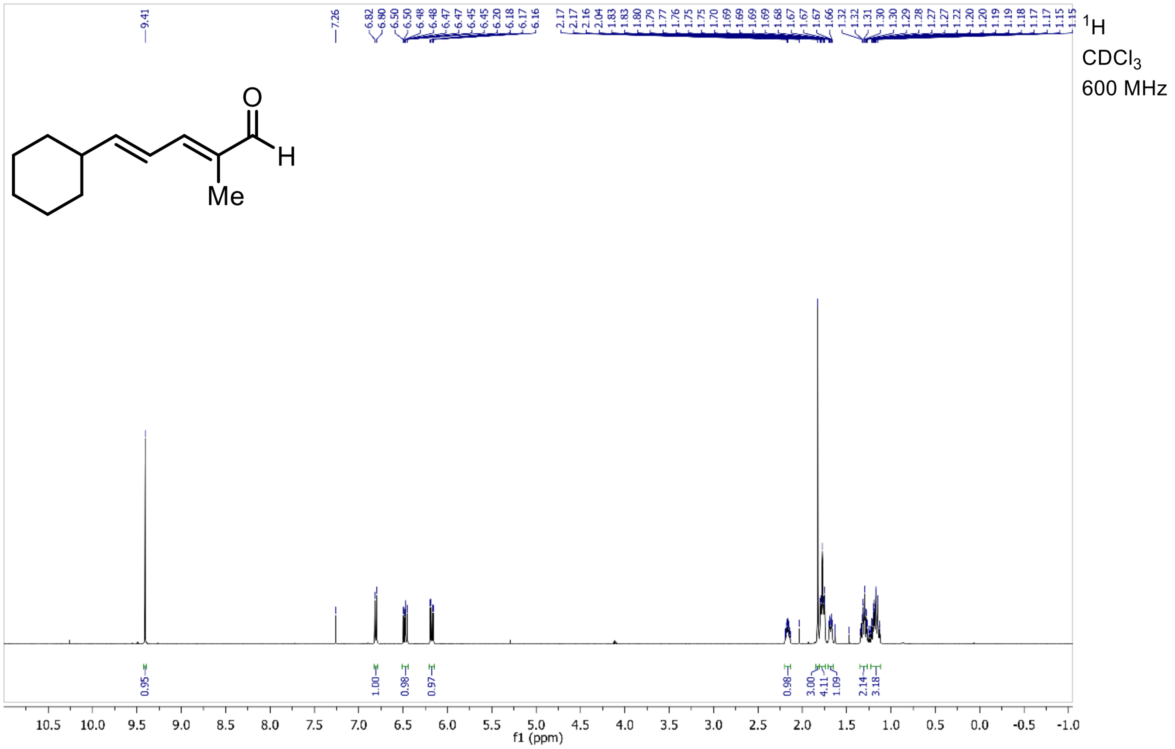


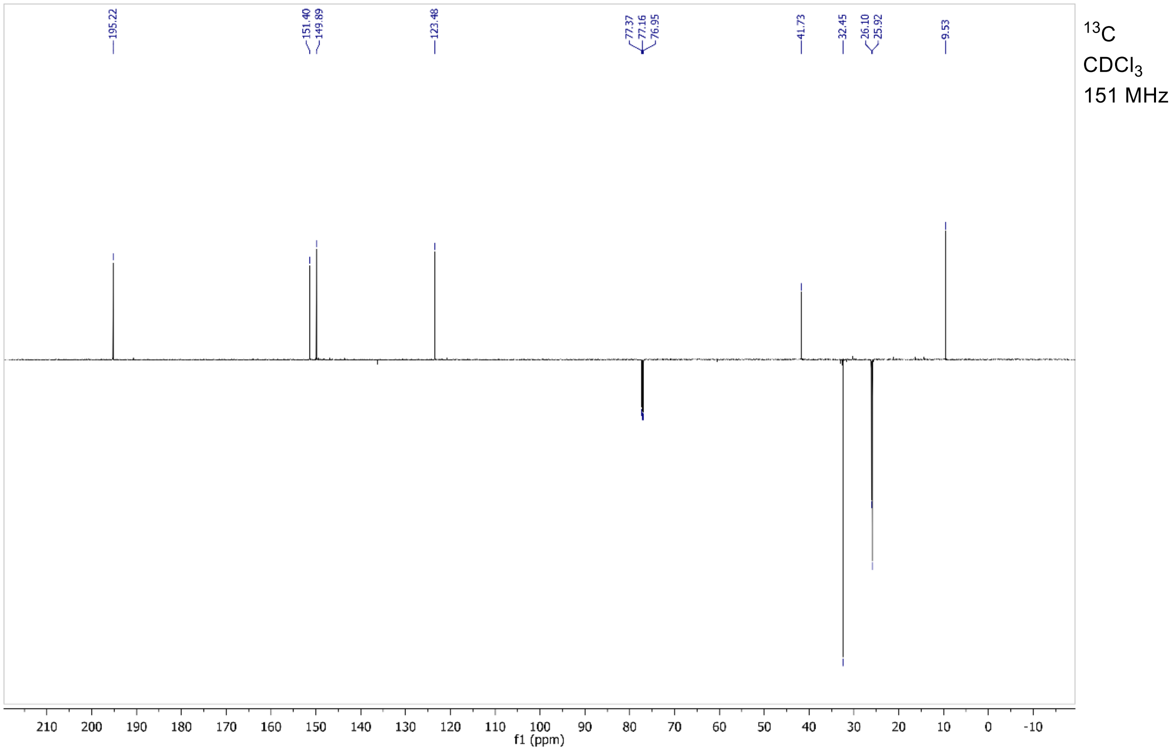

(*R*,4*E*,6*E*)-1-((*S*)-4-benzyl-2-thioxothiazolidin-3-yl)-7-cyclohexyl-3-hydroxy-4-methylhepta-4,6-dien-1-one (S-78)

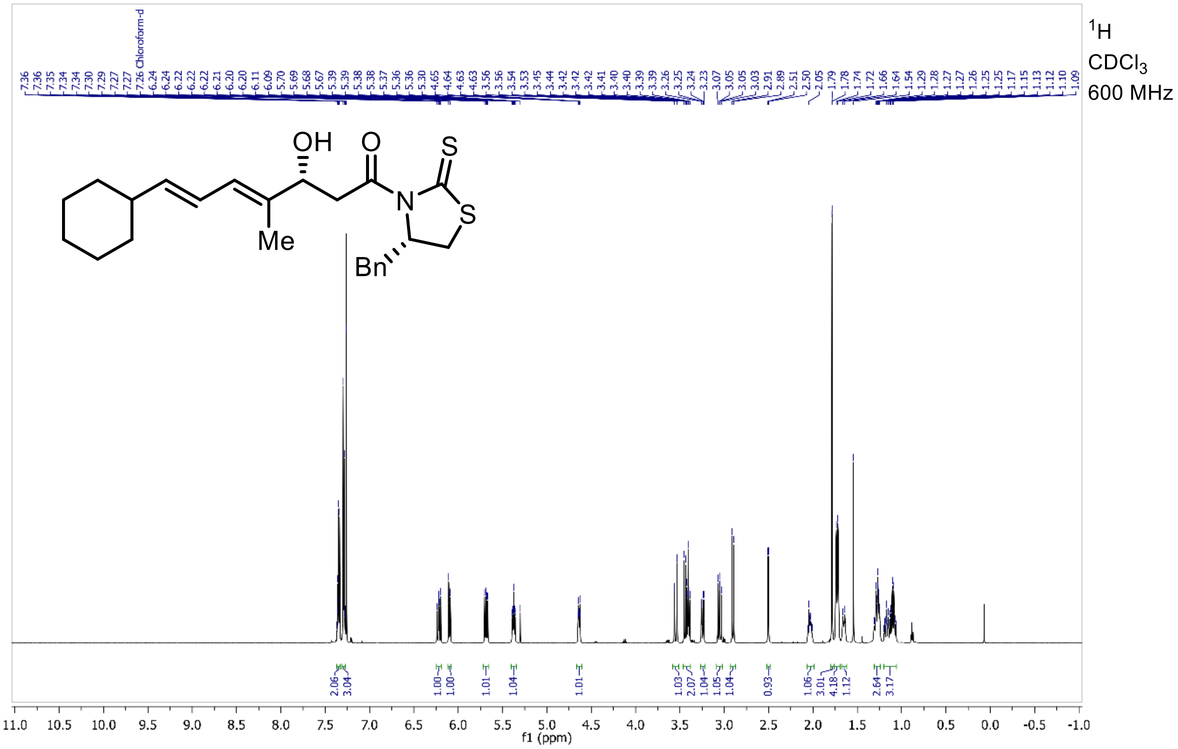


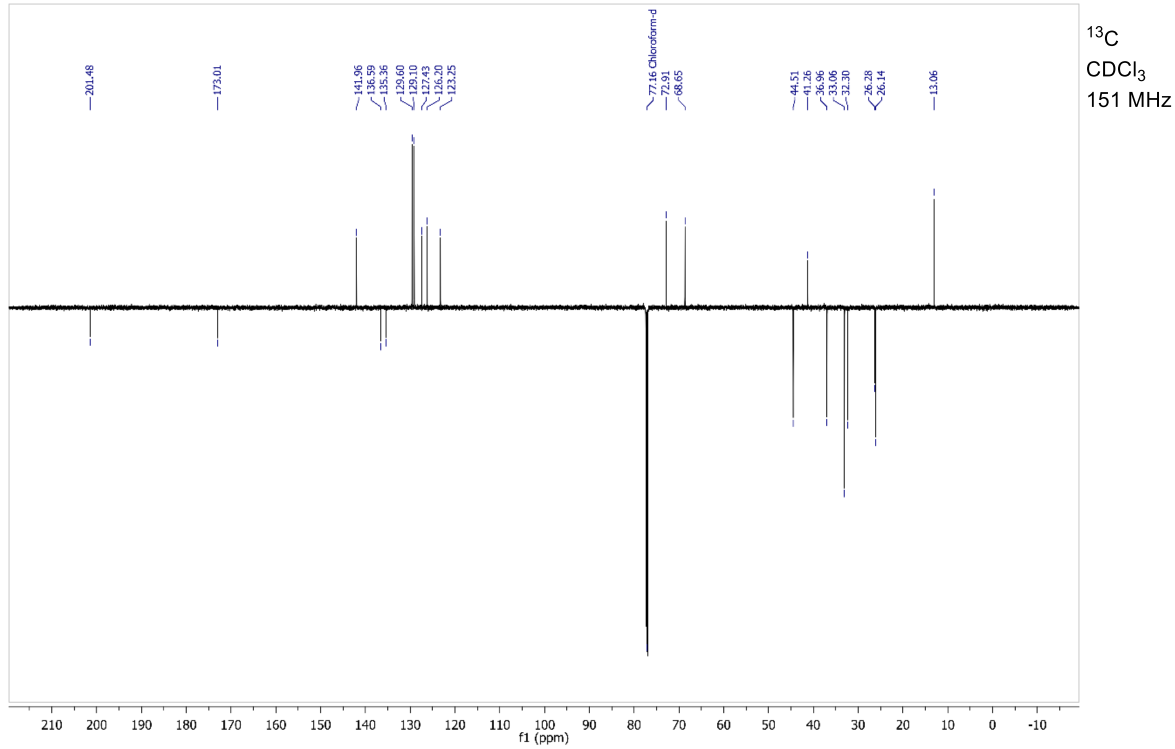

(*R*,4*E*,6*E*)-*N*-((2*R*,3*S*)-2-((*tert*-butyldimethylsilyl)oxy)-3-methyl-4-oxo-4-(((*E*)-4-(4,4,5,5-tetramethyl-1,3,2-dioxaborolan-2-yl)but-3-en-1-yl)amino)butyl)-7-cyclohexyl-3-hydroxy-4-methylhepta-4,6-dienamide (S-79)

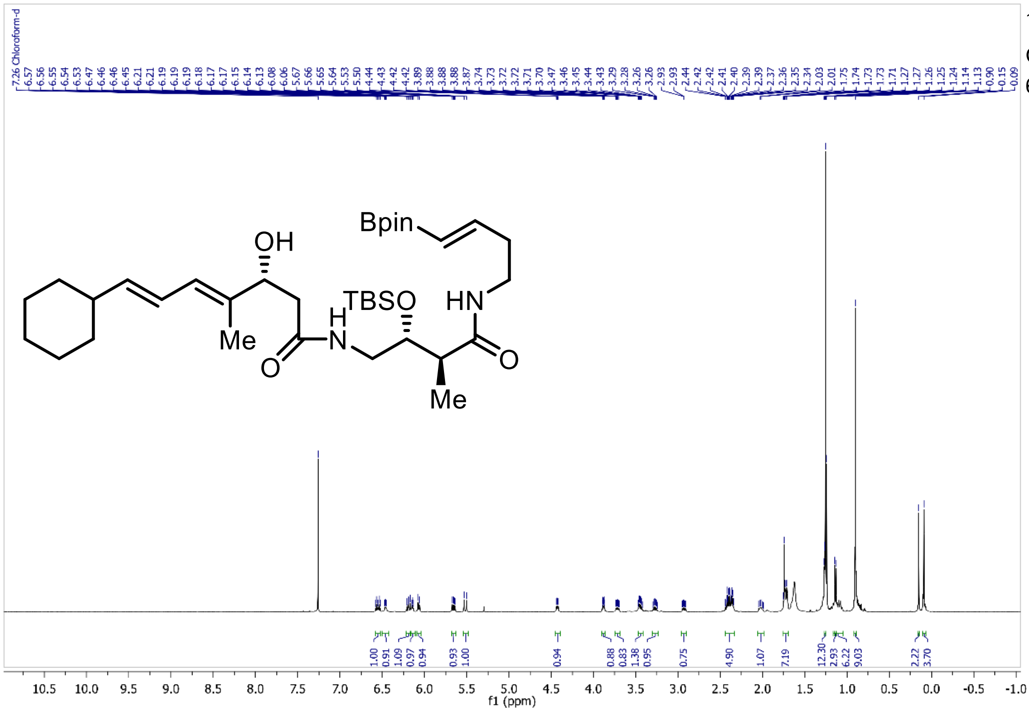


$^{1}H$
$CDCl_3$
600 MHz

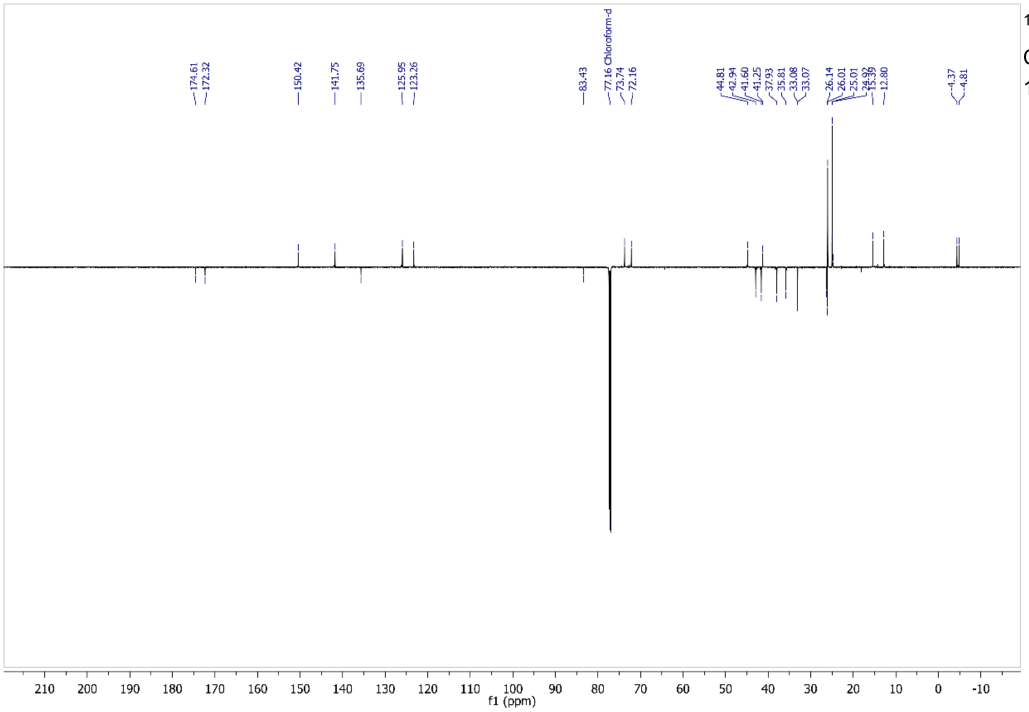


$^{13}C$
$CDCl_3$
151 MHz

(*R*,4*E*,6*E*)-1-(((2*R*,3*S*)-2-((*tert*-butyldimethylsilyl)oxy)-3-methyl-4-oxo-4-(((*E*)-4-(4,4,5,5-tetramethyl-1,3,2-dioxaborolan-2-yl)but-3-en-1-yl)amino)butyl)amino)-7-cyclohexyl-4-methyl-1-oxohepta-4,6-dien-3-yl (1*R*,4*R*)-4-chlorocyclobut-2-ene-1-carboxylate (*S*-80)

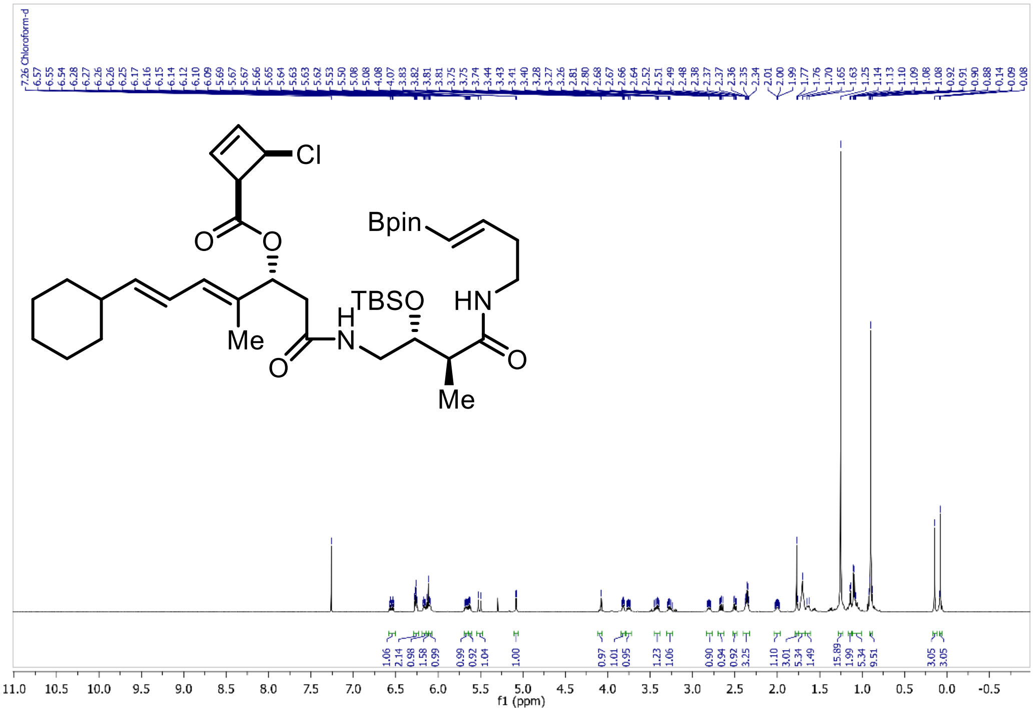


$^{1}H$
$CDCl_3$
600 MHz

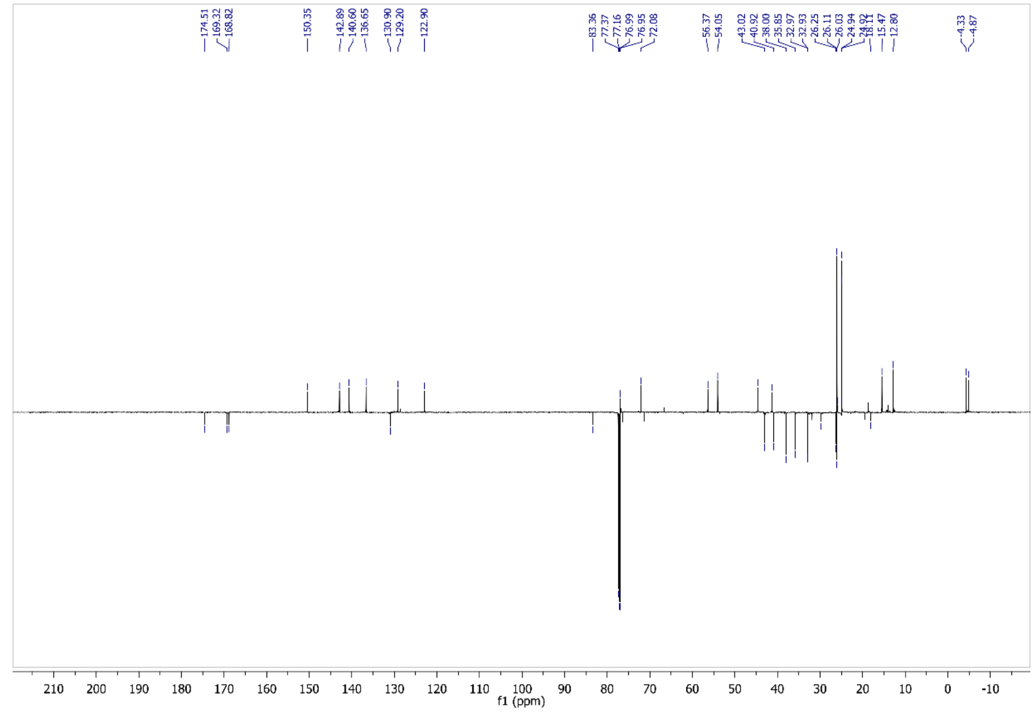


$^{13}C$
$CDCl_3$
151 MHz

(2*R*,7*R*,8*S*,13*E*,15*E*,17*E*)-7-((*tert*-butyldimethylsilyl)oxy)-2-((2*E*,4*E*)-5-cyclohexylpenta-2,4-dien-2-yl)-8-methyl-1-oxa-5,10-diazacyclononadeca-13,15,17-triene-4,9,19-trione (S-81)

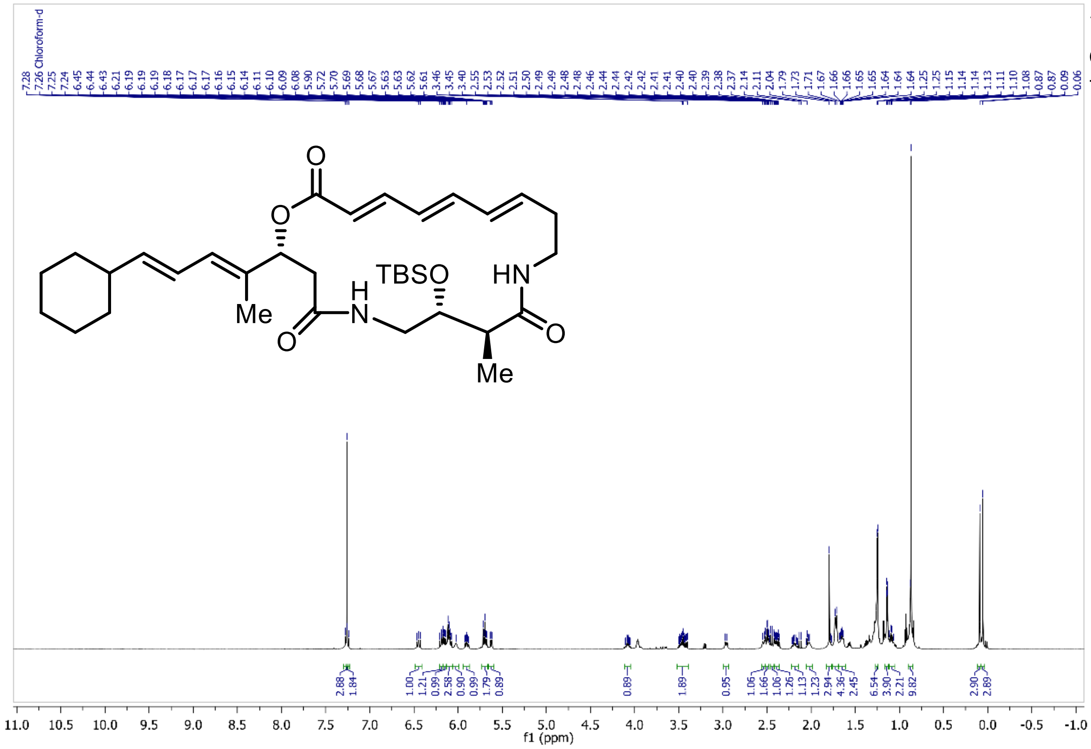


$^{1}H$
$CDCl_3$
700 MHz

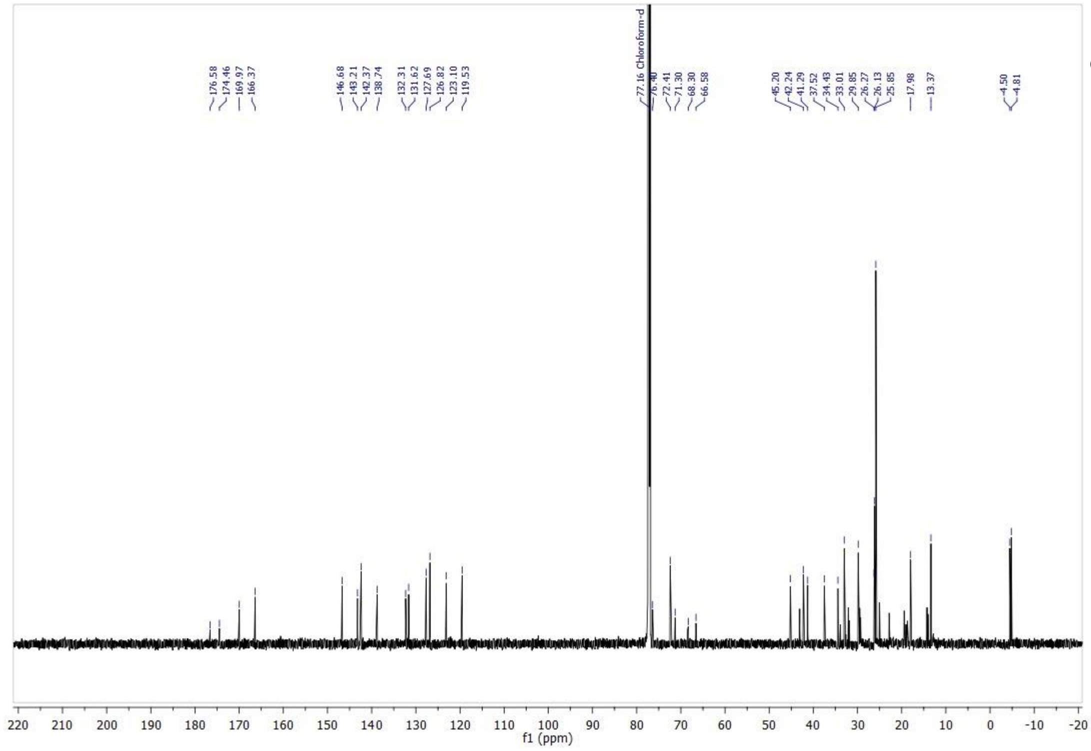


$^{13}C$
$CDCl_3$
176 MHz

## Fs-FR6

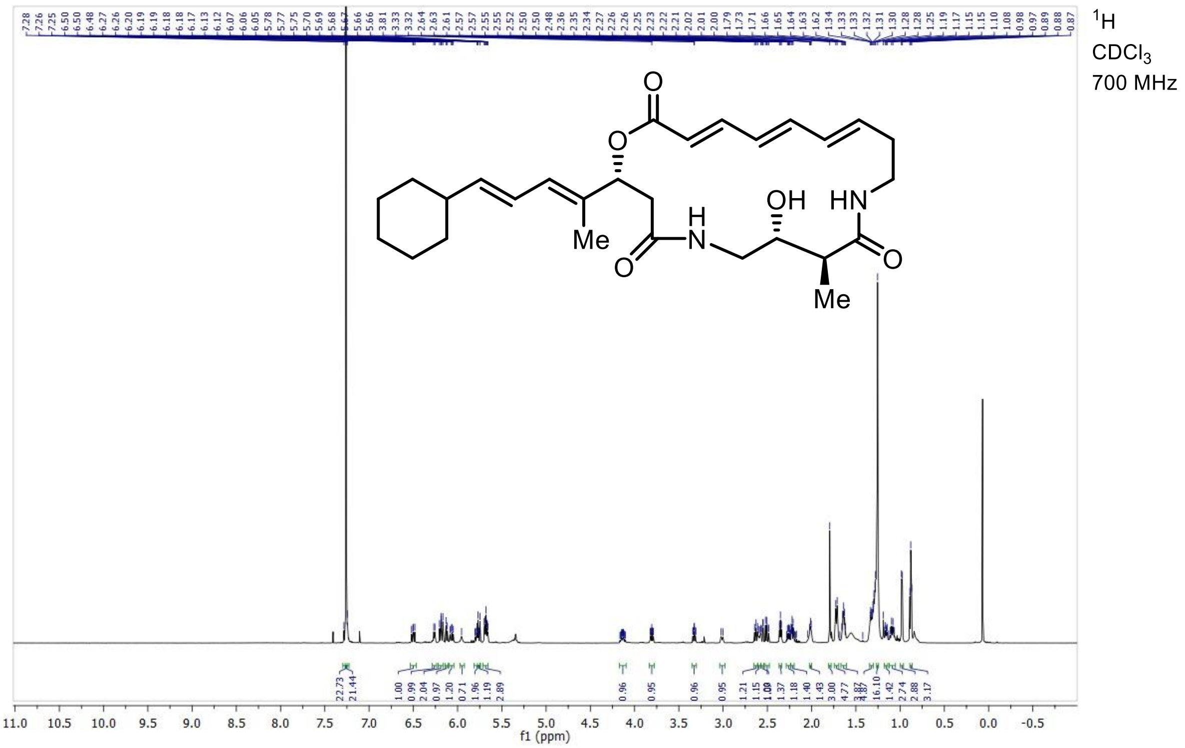

$^1$H
$CDCl_3$
700 MHz
Me
O
OH
HN
H
N
Me
f1 (ppm)


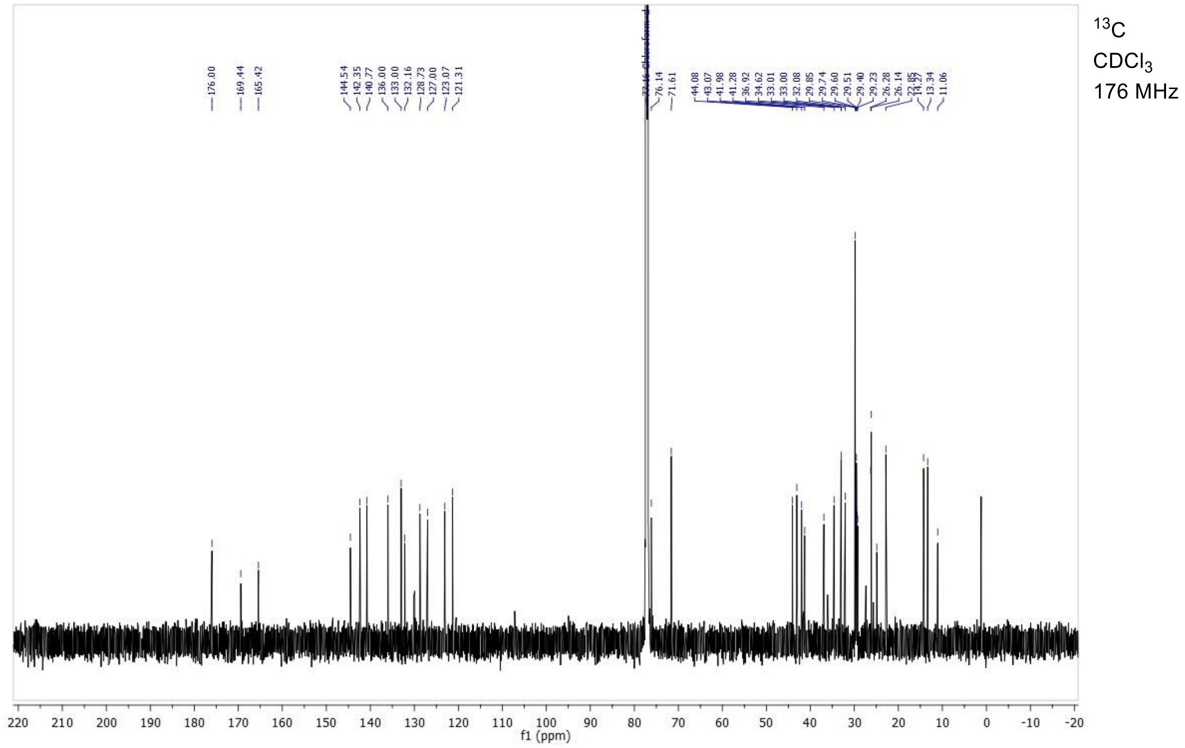

$^{13}$C
$CDCl_3$
176 MHz
176.00
169.44
165.42
144.54
142.35
140.77
136.00
133.00
132.16
128.73
127.00
123.07
121.31
76.14
71.61
44.08
43.07
41.98
41.28
36.92
34.62
33.01
33.00
32.08
29.85
29.74
29.60
29.51
29.40
29.23
26.28
26.14
13.34
11.06
f1 (ppm)

Fs-FR7

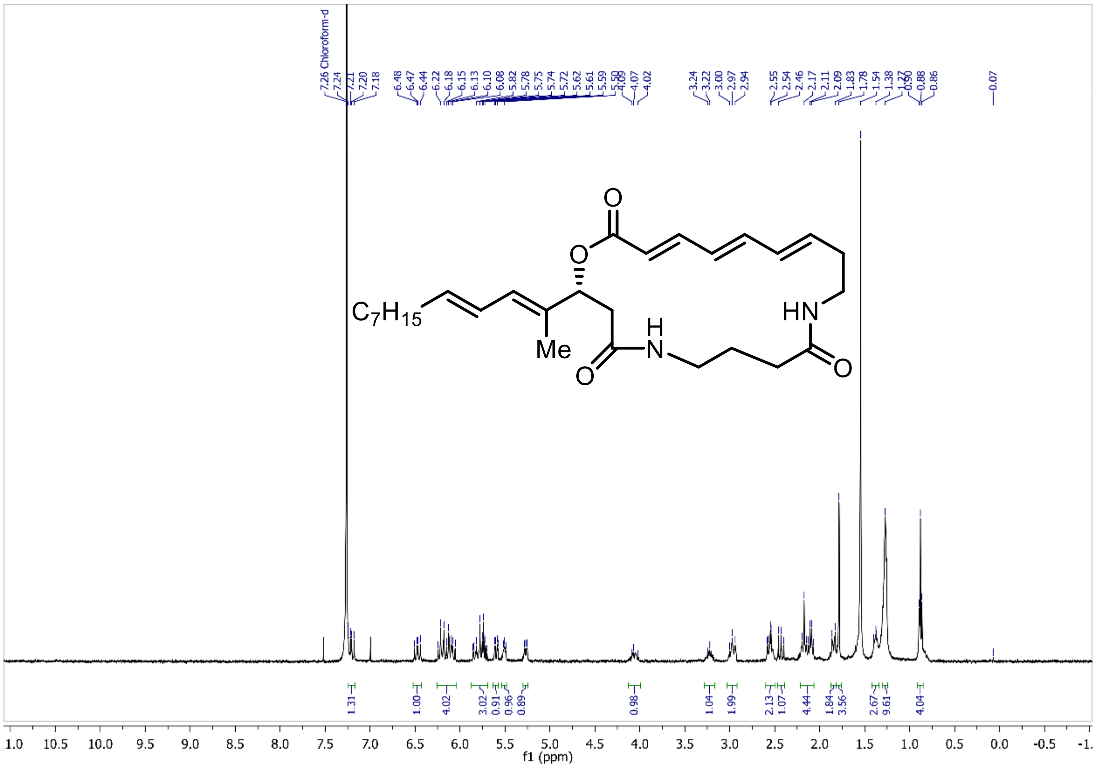


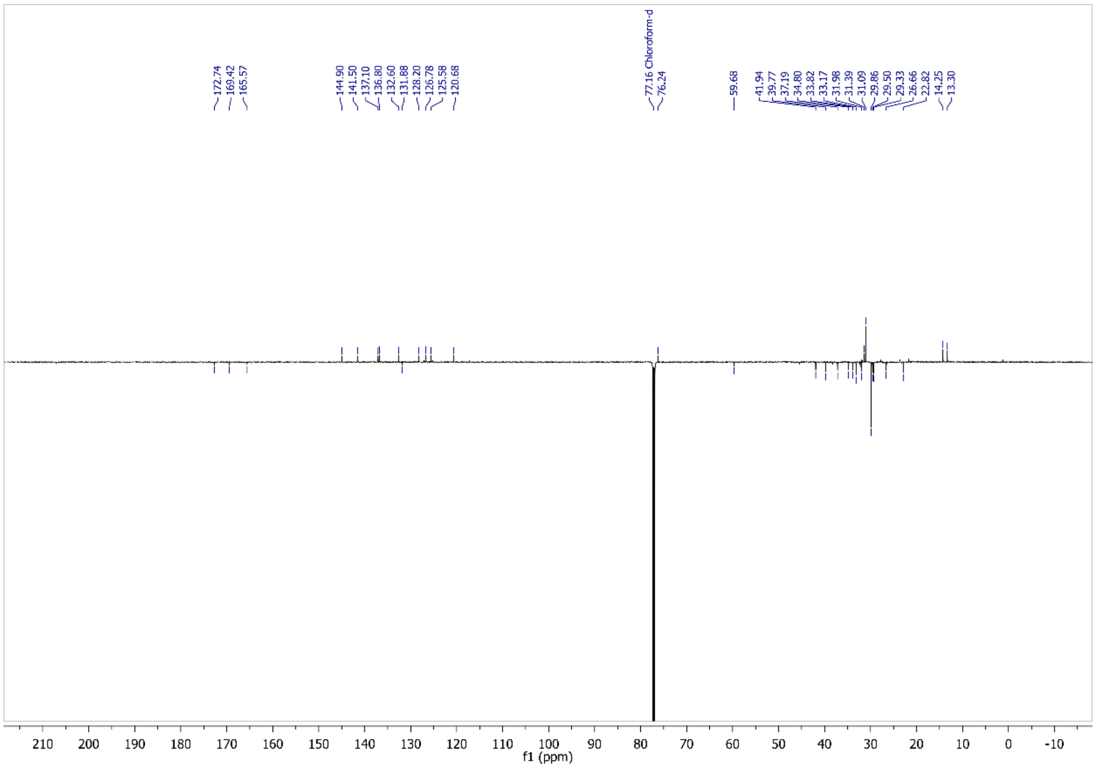

(*R*,4*E*,6*E*)-1-((*S*)-4-benzyl-2-thioxothiazolidin-3-yl)-3-((*tert*-butyldimethylsilyl)oxy)-4-methyltetradeca-4,6-dien-1-one (S-87)

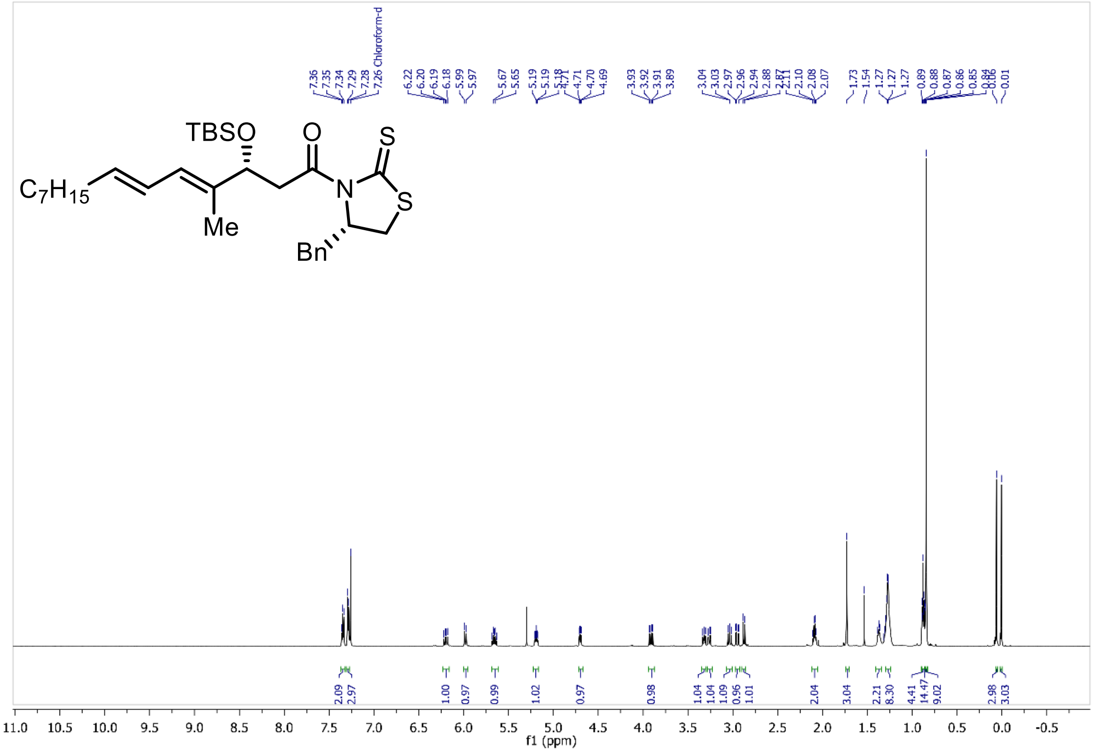


$^1H$
$CDCl_3$
600 MHz

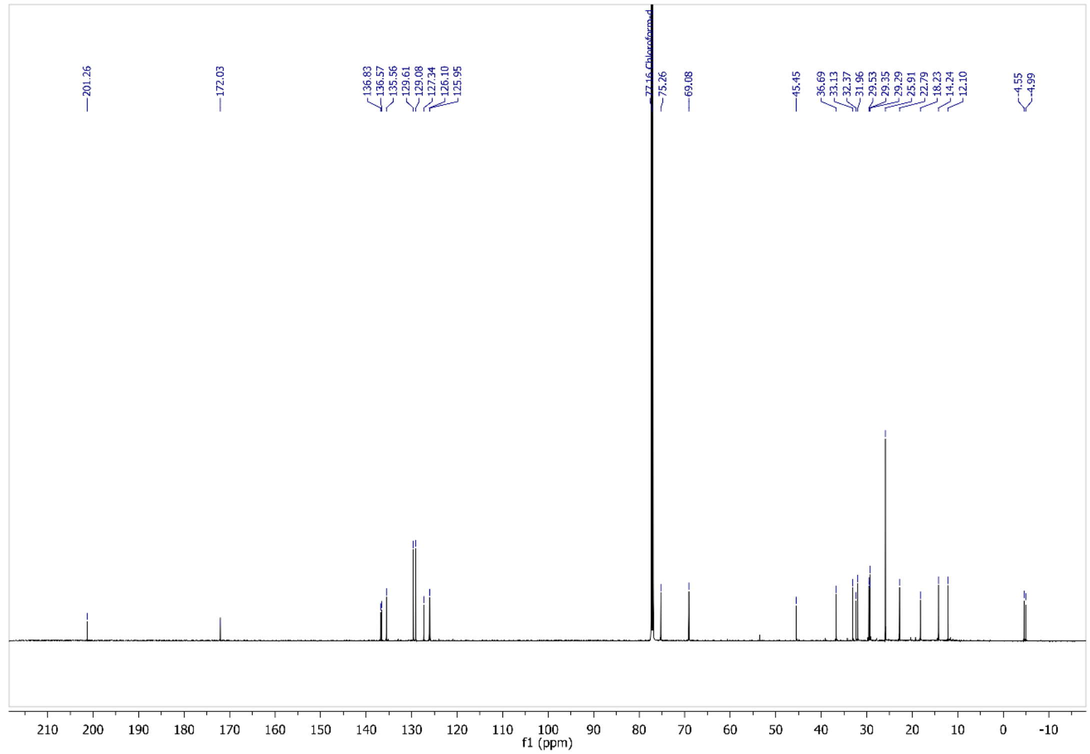


$^{13}C$
$CDCl_3$
151 MHz

## (*R*,4*E*,6*E*)-3-((*tert*-butyldimethylsilyl)oxy)-4-methyltetradeca-4,6-dienal (13)

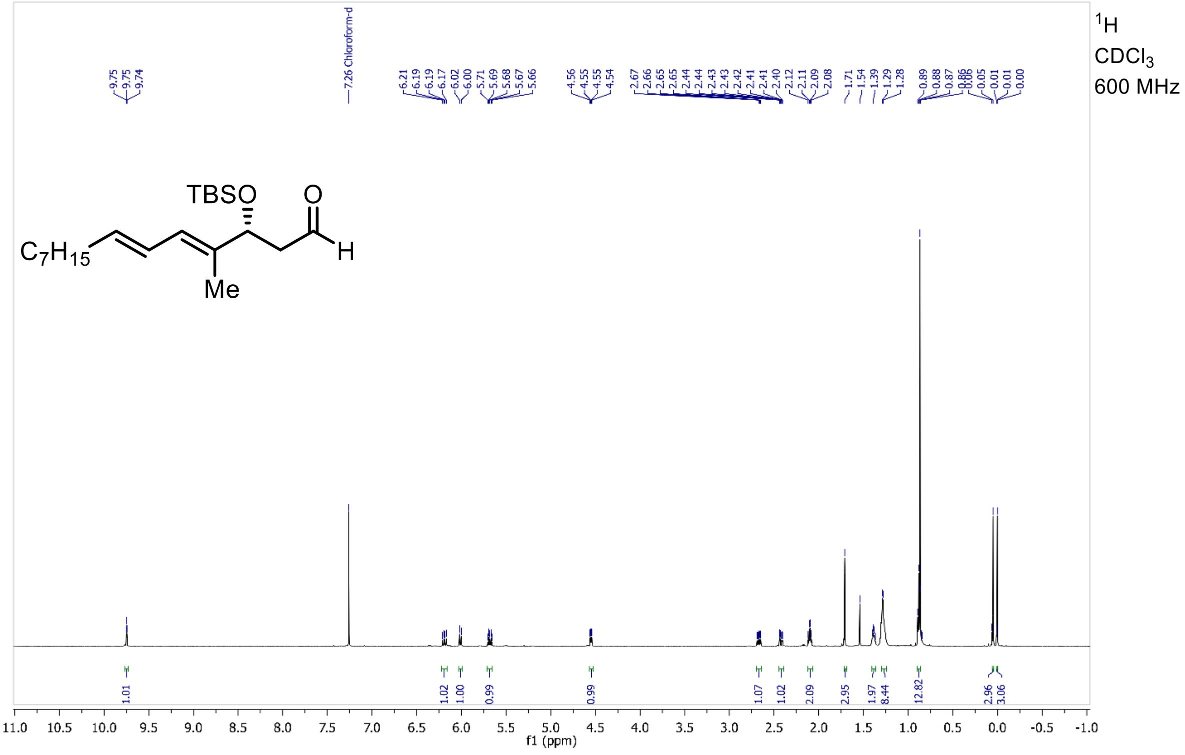


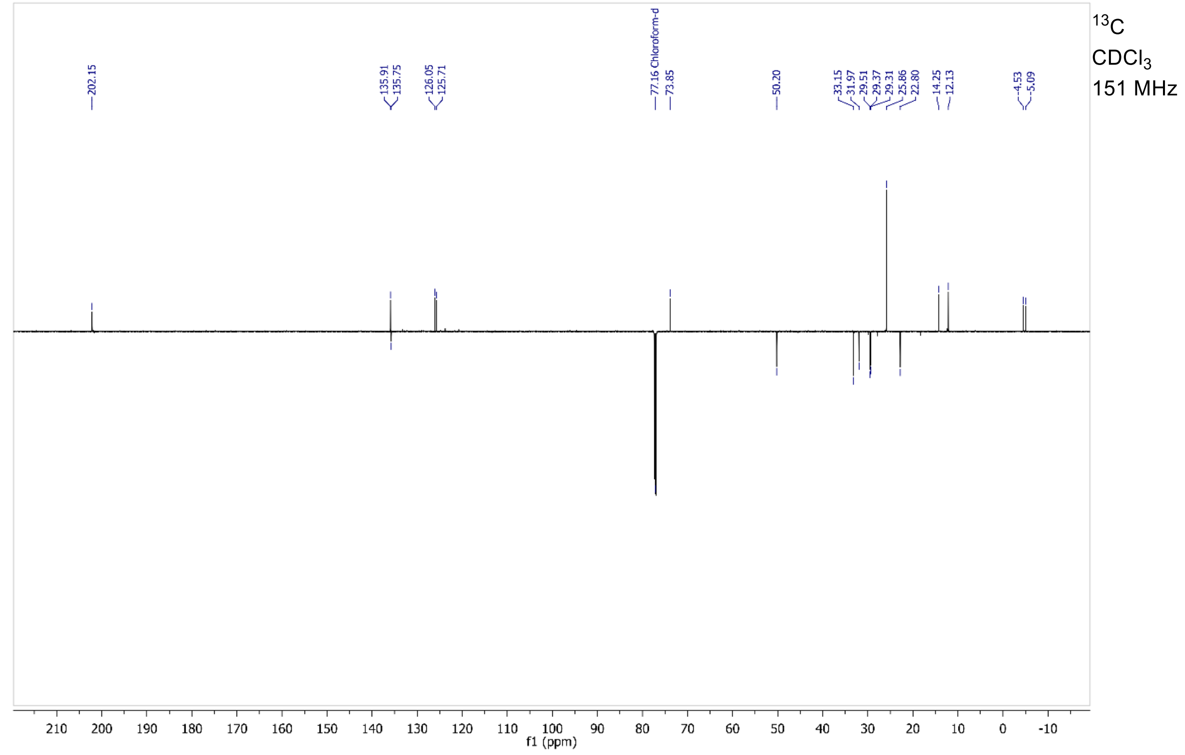

## *tert*-Butyldimethyl(((*R*,5*E*,7*E*)-5-methylpentadeca-5,7-dien-1-yn-4-yl)oxy)silane (S-88)

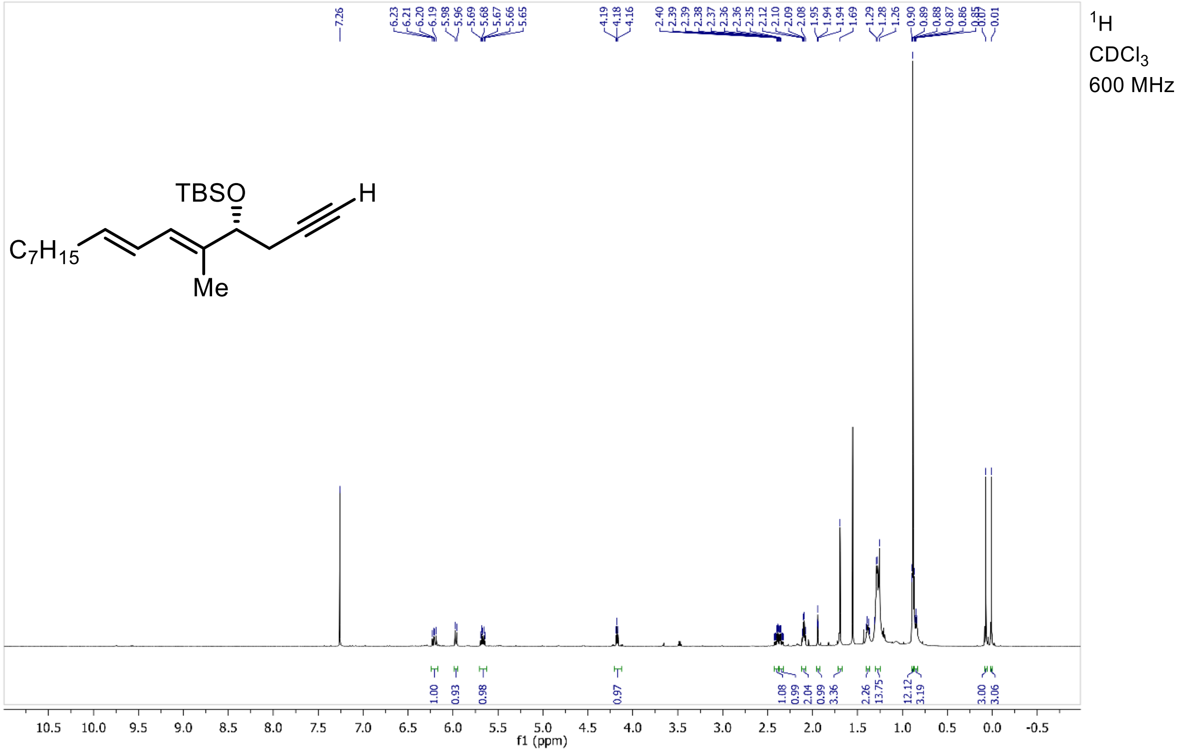


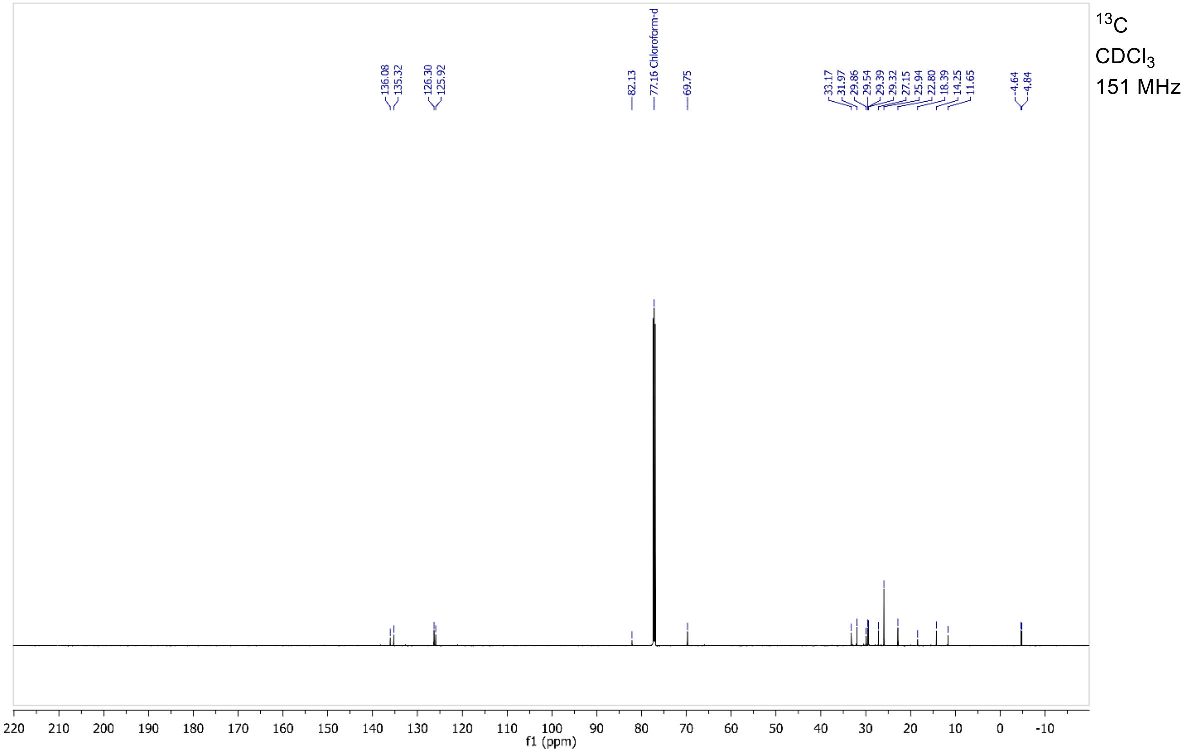

## (*R*,5*E*,7*E*)-5-methylpentadeca-5,7-dien-1-yn-4-ol (15)

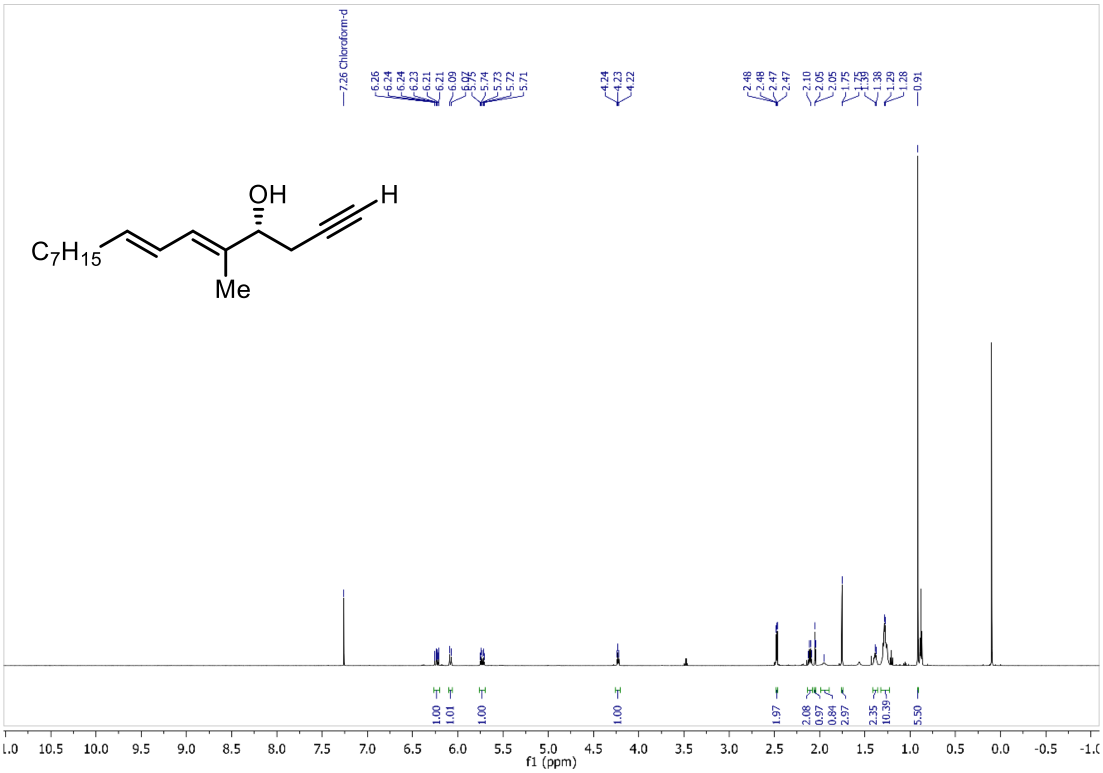


$^1$H
$CDCl_3$
600 MHz

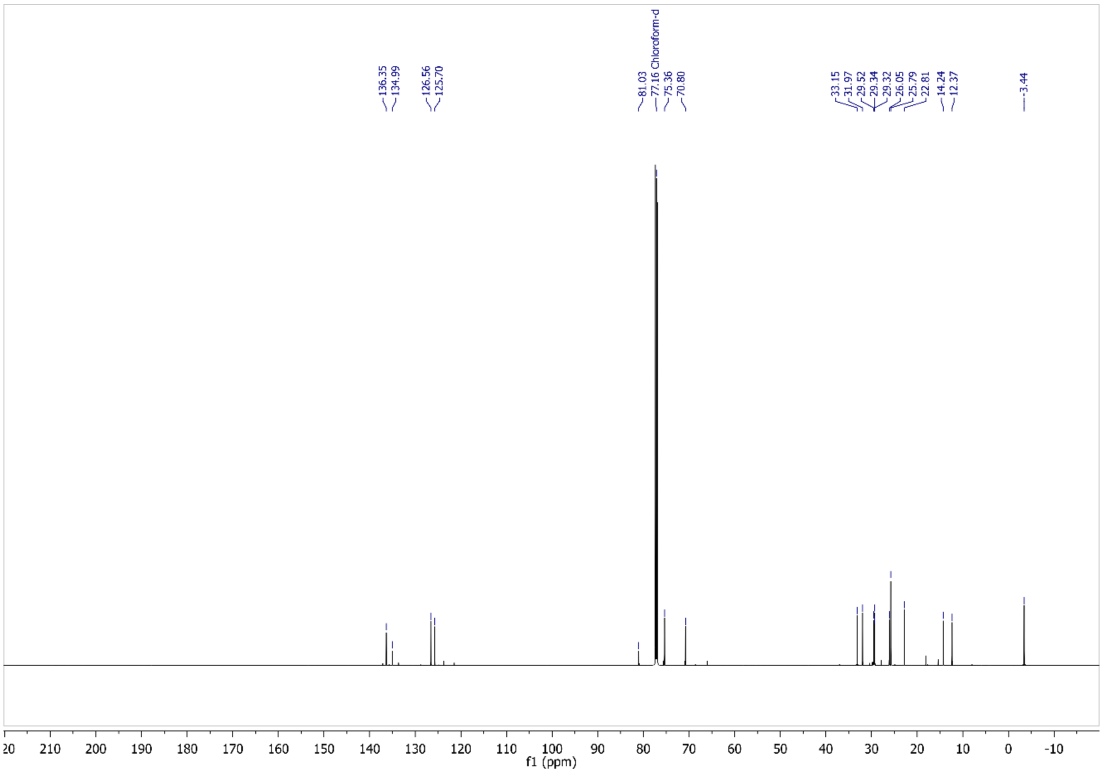


$^{13}$C
$CDCl_3$
151 MHz

(2*S*,3*R*)-3-((*tert*-butyldimethylsilyl)oxy)-4-(4-((*R*,3*E*,5*E*)-2-hydroxy-3-methyltrideca-3,5-dien-1-yl)-1*H*-1,2,3-triazol-1-yl)-2-methyl-*N*-((*E*)-4-(4,4,5,5-tetramethyl-1,3,2-dioxaborolan-2-yl)but-3-en-1-yl)butanamide (17)

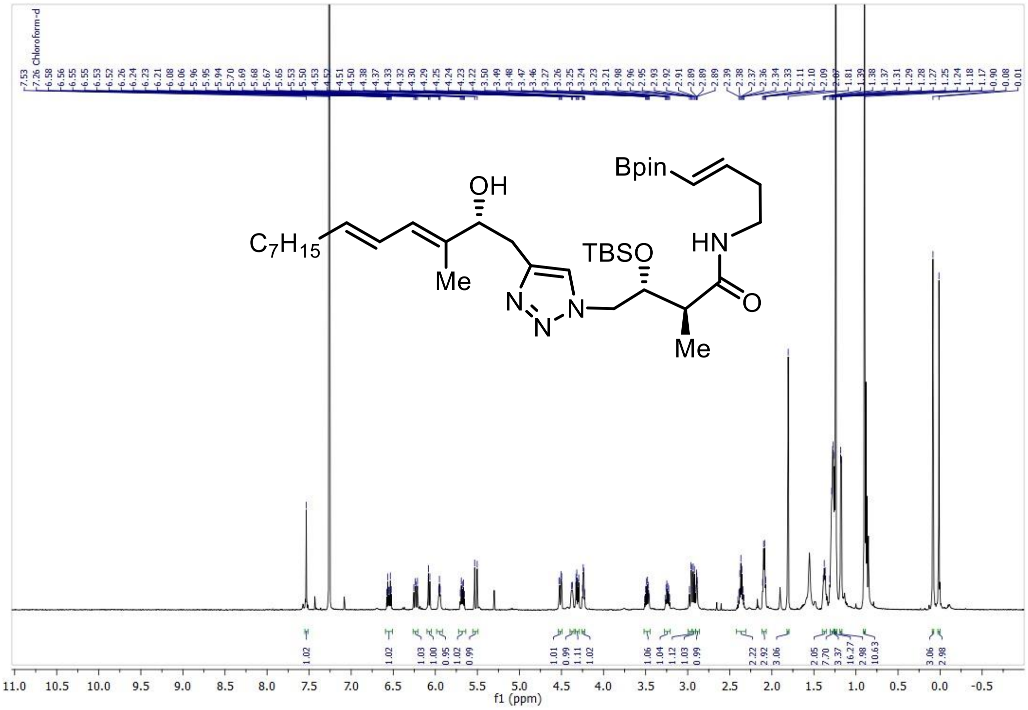


$^1H$
$CDCl_3$
600 MHz

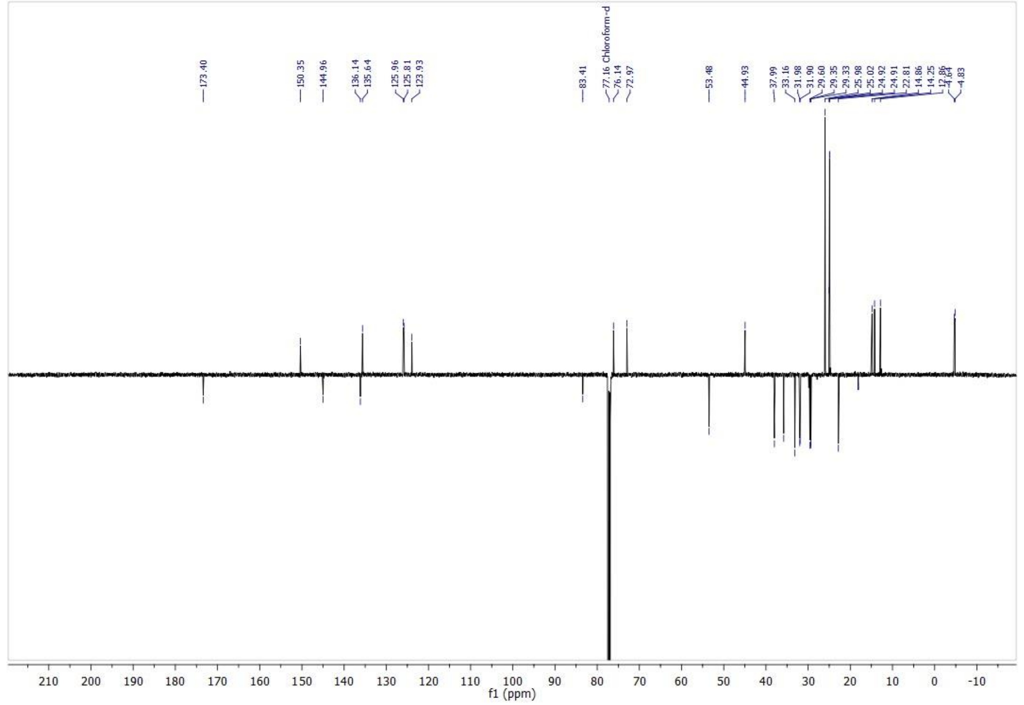


$^{13}C$
$CDCl_3$
151 MHz

(*R*,3*E*,5*E*)-1-(1-((2*R*,3*S*)-2-((*tert*-butyldimethylsilyl)oxy)-3-methyl-4-oxo-4-(((*E*)-4-(4,4,5,5-tetramethyl-1,3,2-dioxaborolan-2-yl)but-3-en-1-yl)amino)butyl)-1*H*-1,2,3-triazol-4-yl)-3-methyltrideca-3,5-dien-2-yl (1*R*,4*R*)-4-chlorocyclobut-2-ene-1-carboxylate (S-89)

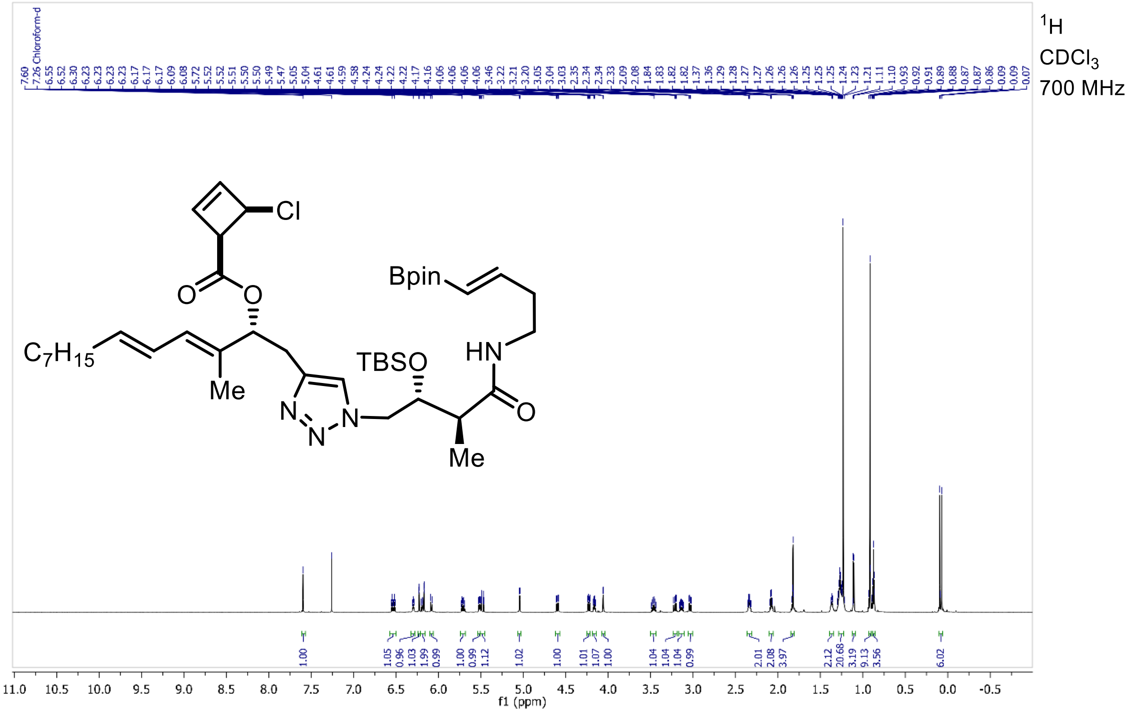


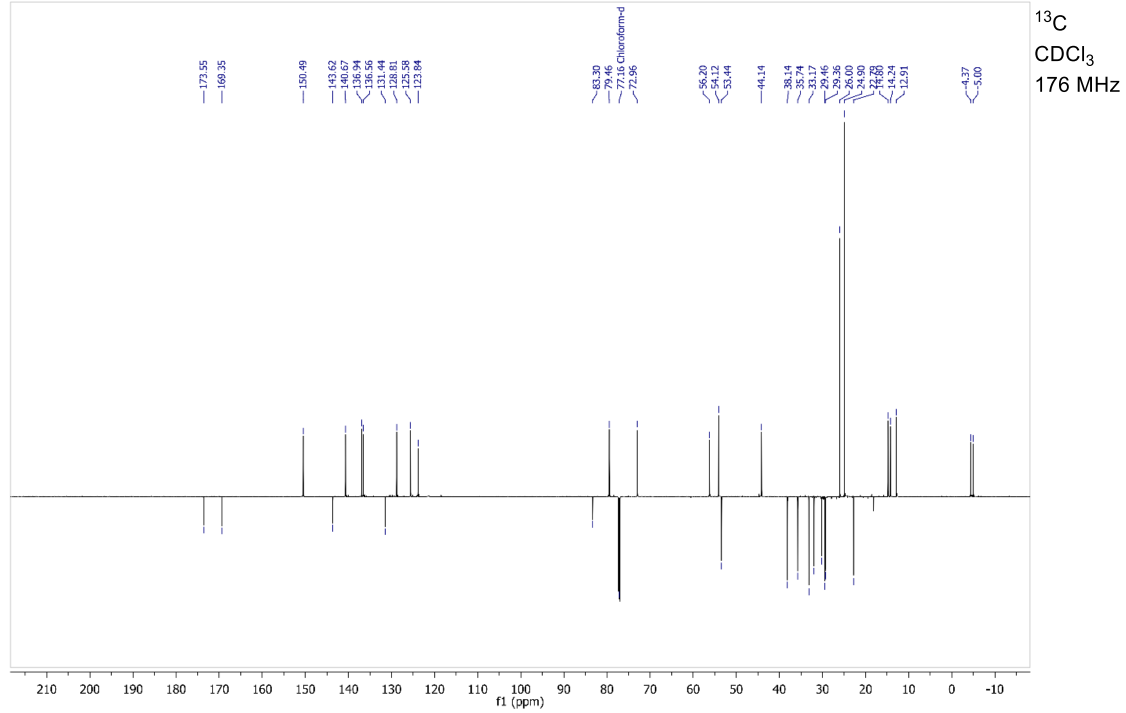

(14*Z*,3*R*,6*E*,8*E*,10*E*,16*S*,17*R*)-17-((*tert*-butyldimethylsilyl)oxy)-3-((2*E*,4*E*)-dodeca-2,4-dien-2-yl)-16-methyl-11*H*-4-oxa-14-aza-1(4,1)-triazolacyclooctadecaphane-6,8,10-triene-5,15-dione (S-90)

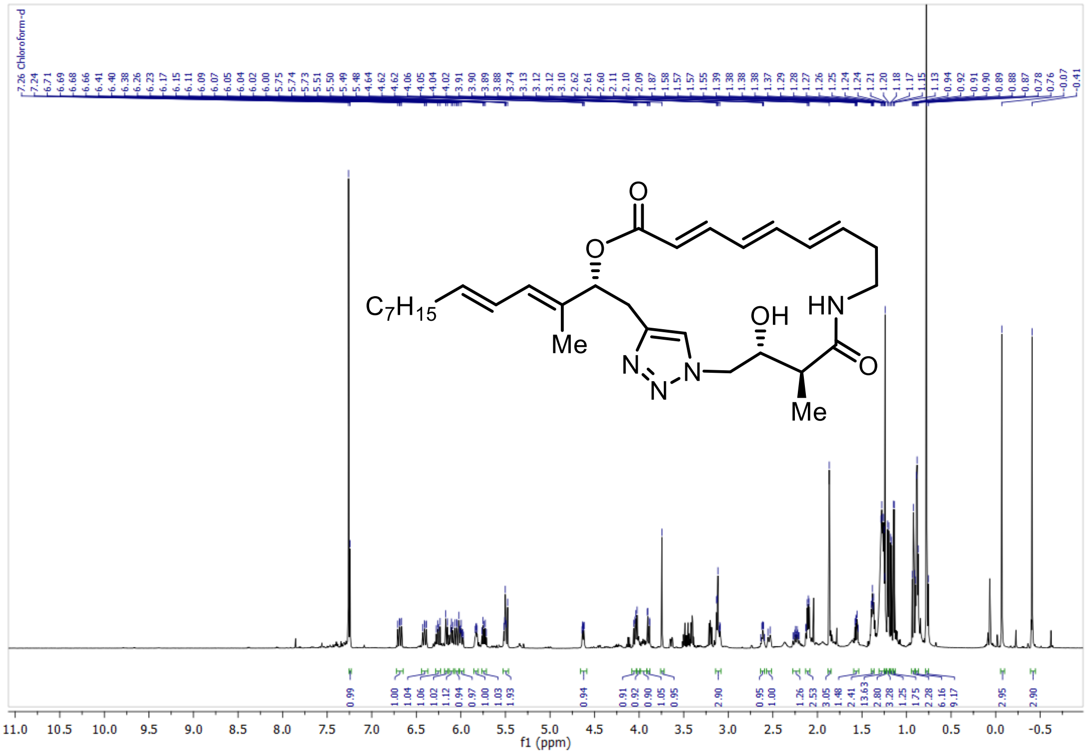


$^{1}H$
$CDCl_3$
600 MHz

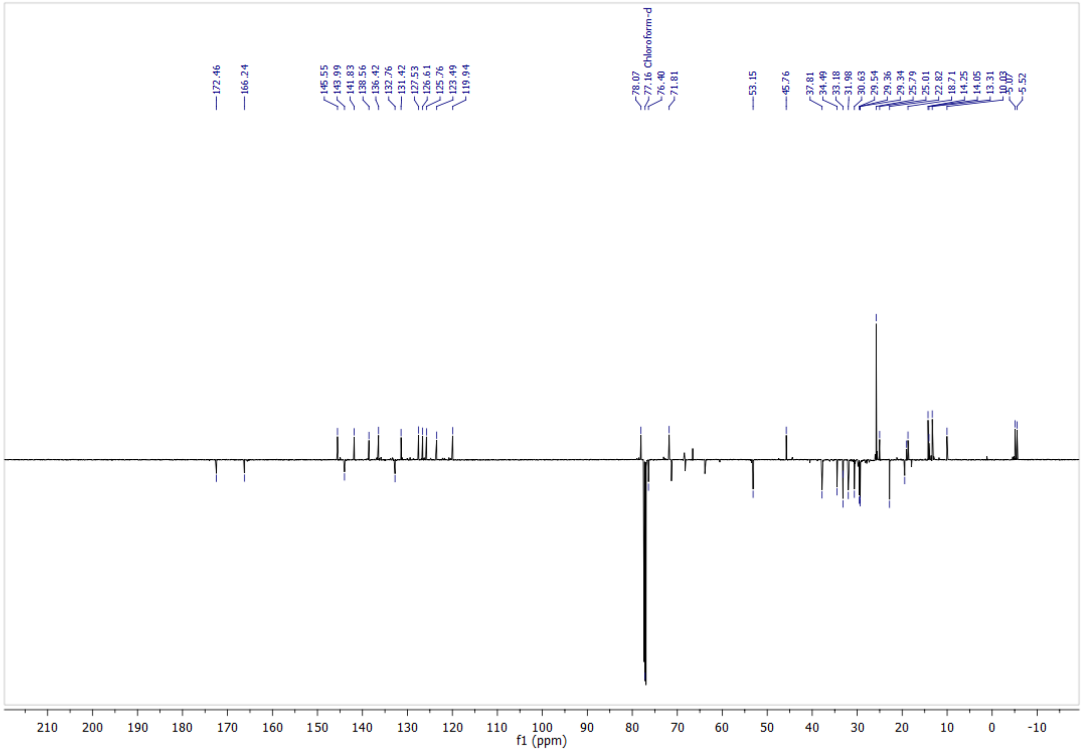


$^{13}C$
$CDCl_3$
151 MHz

## Fs-FR9

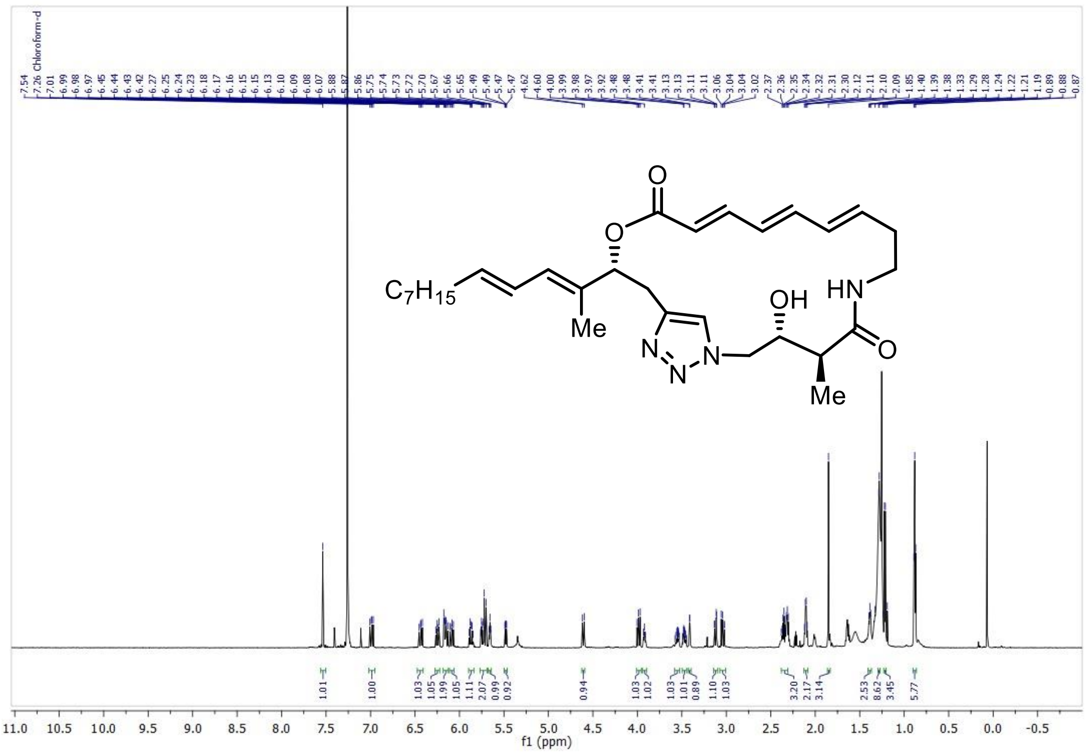


$^1H$
$CDCl_3$
700 MHz

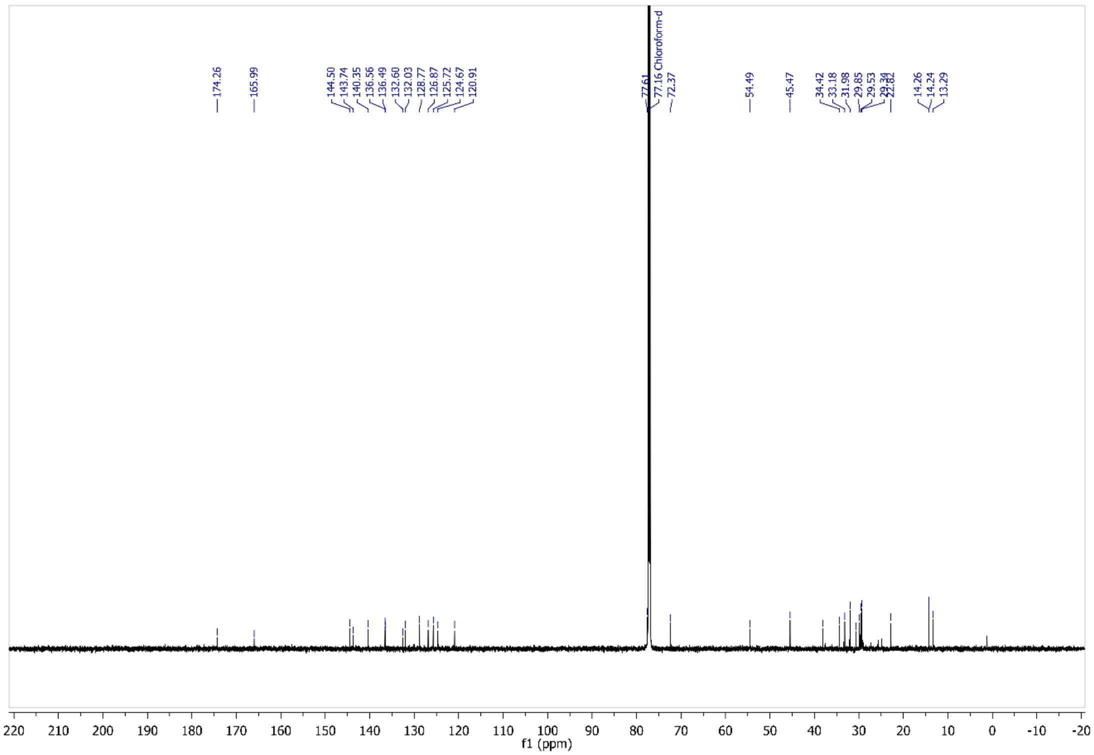


$^{13}C$
$CDCl_3$
176 MHz

## Ethyl (2*S*,3*R*)-4-azido-3-methoxy-2-methylbutanoate (*S*-91a)

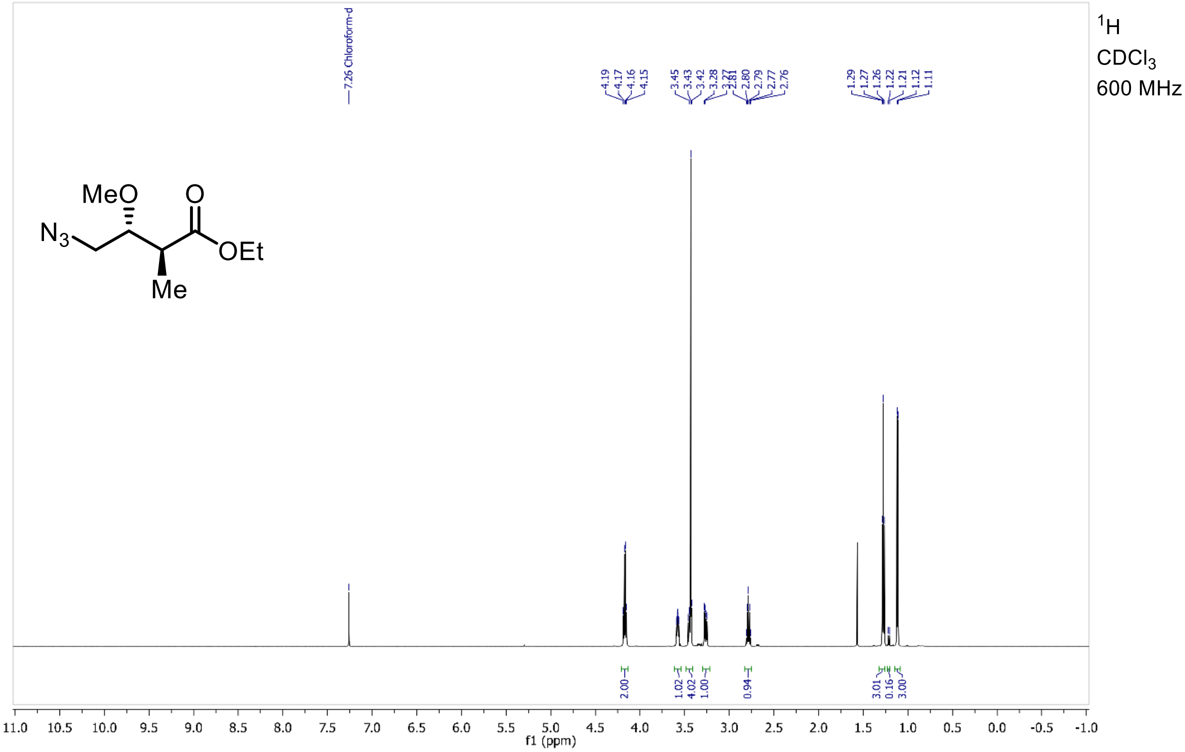


$^{1}H$
$CDCl_3$
600 MHz

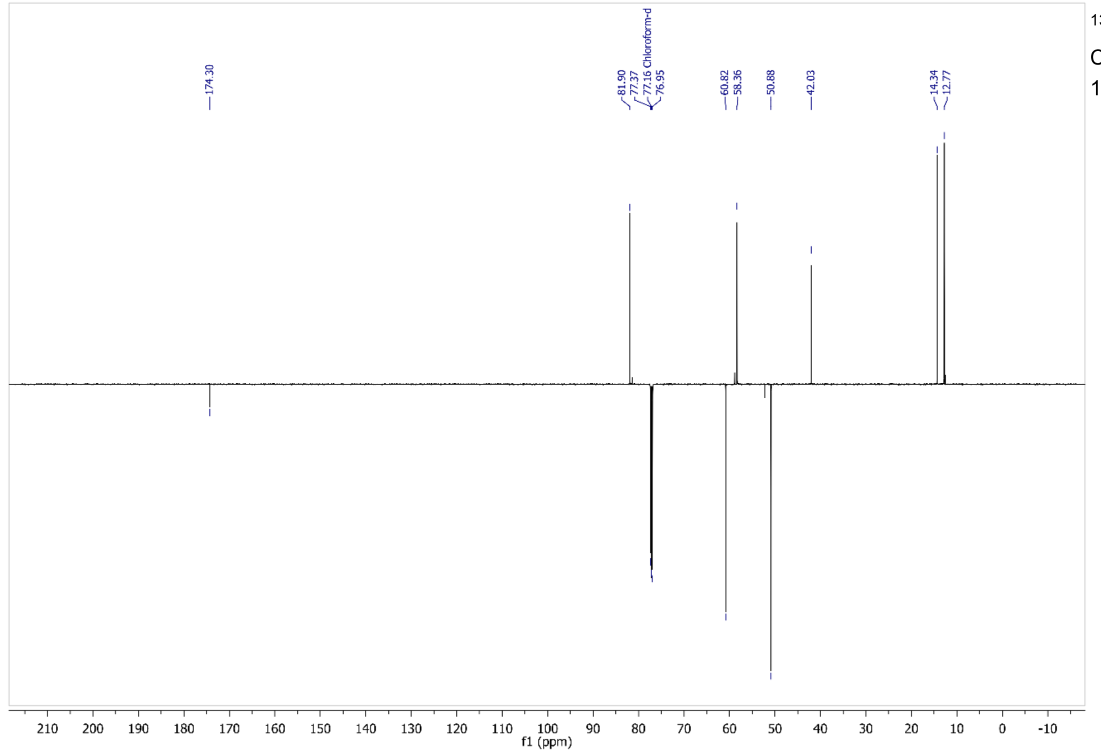


$^{13}C$
$CDCl_3$
151 MHz

(2*S*,3*R*)-4-Azido-3-methoxy-2-methyl-N-((*E*)-4-(4,4,5,5-tetramethyl-1,3,2-dioxaborolan-2-yl)but-3-en-1-yl)butanamide (S-92a)

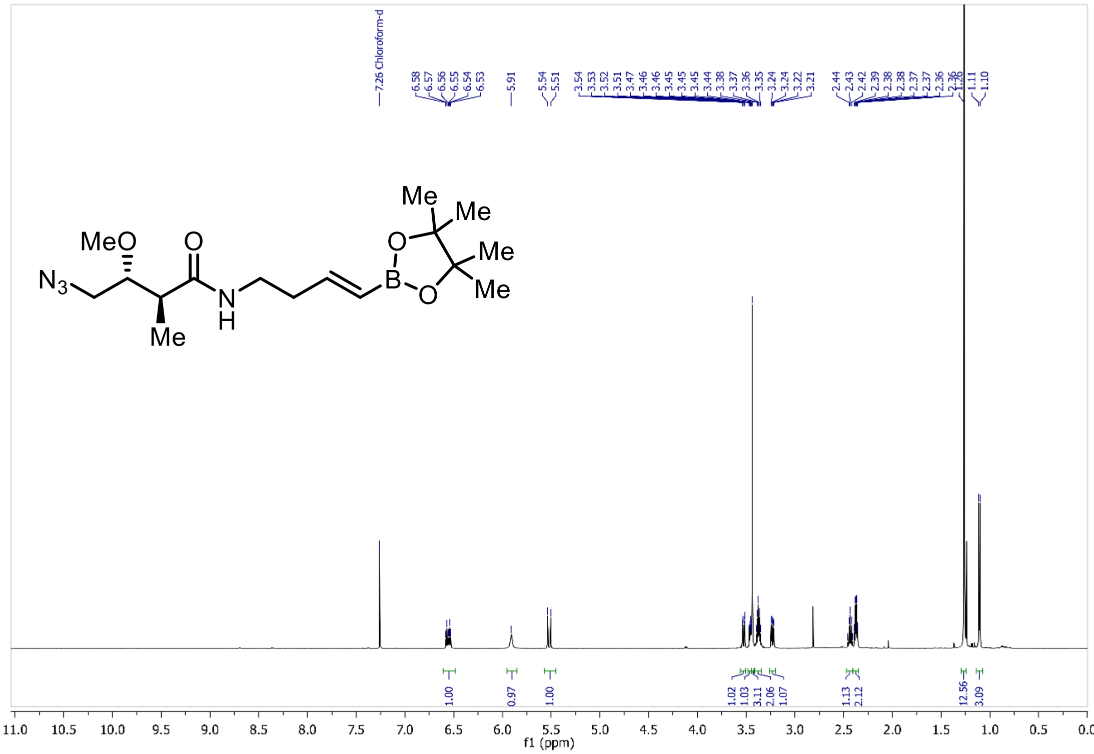


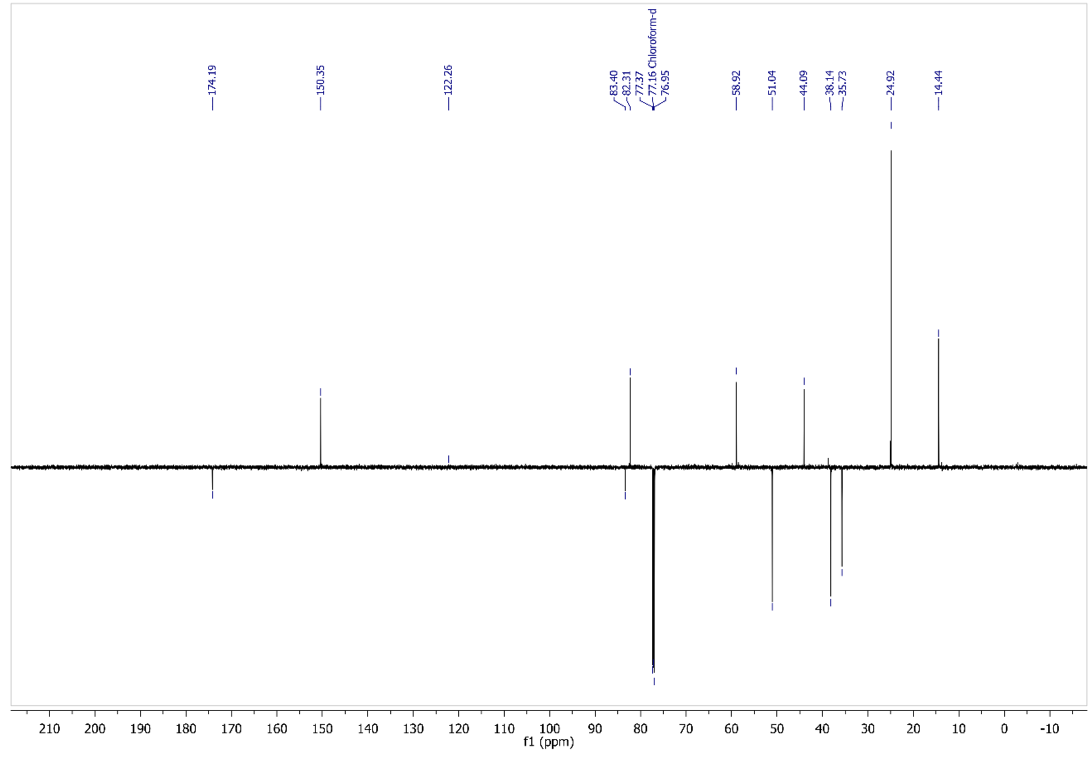

(2*S*,3*R*)-4-Amino-3-methoxy-2-methyl-*N*-((*E*)-4-(4,4,5,5-tetramethyl-1,3,2-dioxaborolan-2-yl)but-3-en-1-yl)butanamide (S-92)

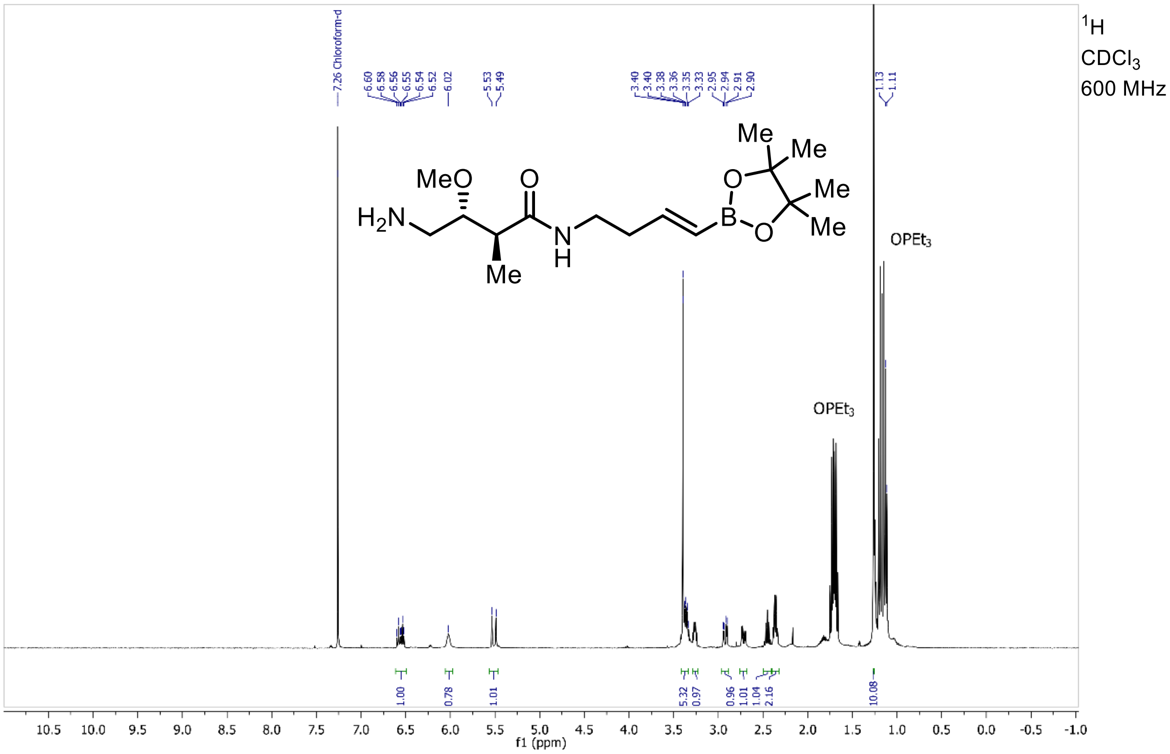

(*R*,4*E*,6*E*)-3-Hydroxy-*N*-((2*R*,3*S*)-2-methoxy-3-methyl-4-oxo-4-(((*E*)-4-(4,4,5,5-tetramethyl-1,3,2-dioxaborolan-2-yl)but-3-en-1-yl)amino)butyl)-4-methyltetradeca-4,6-dienamide (S-93)

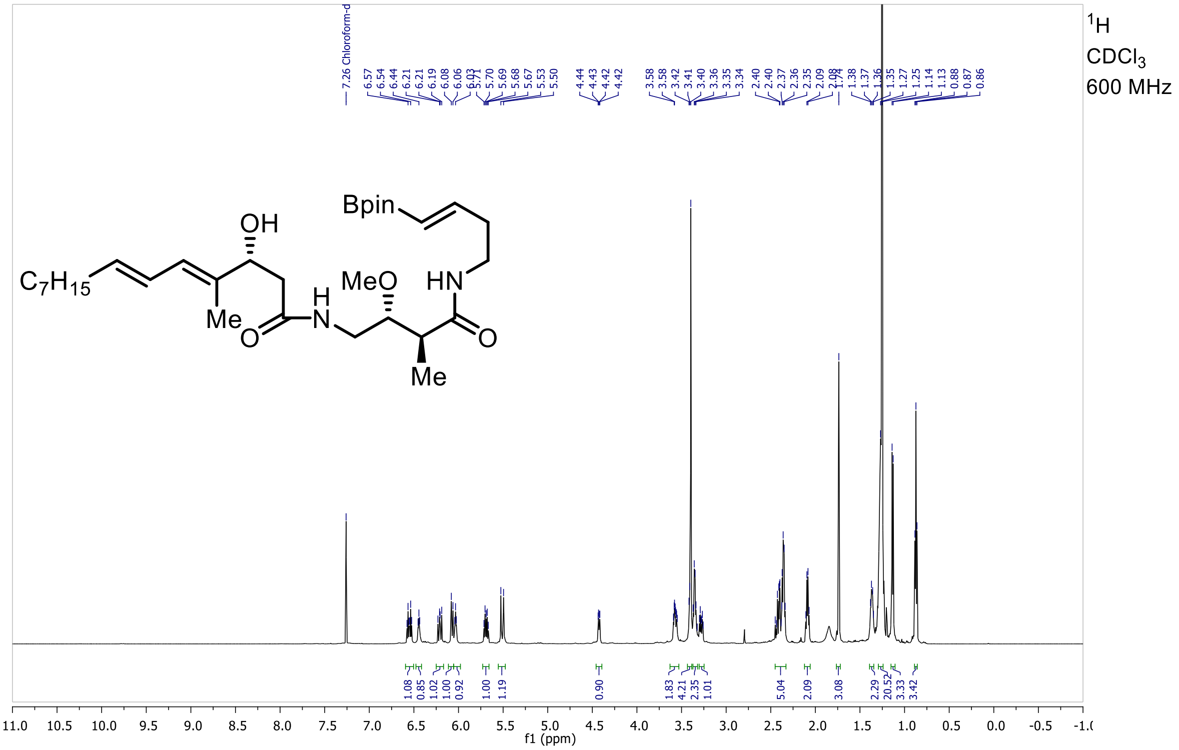


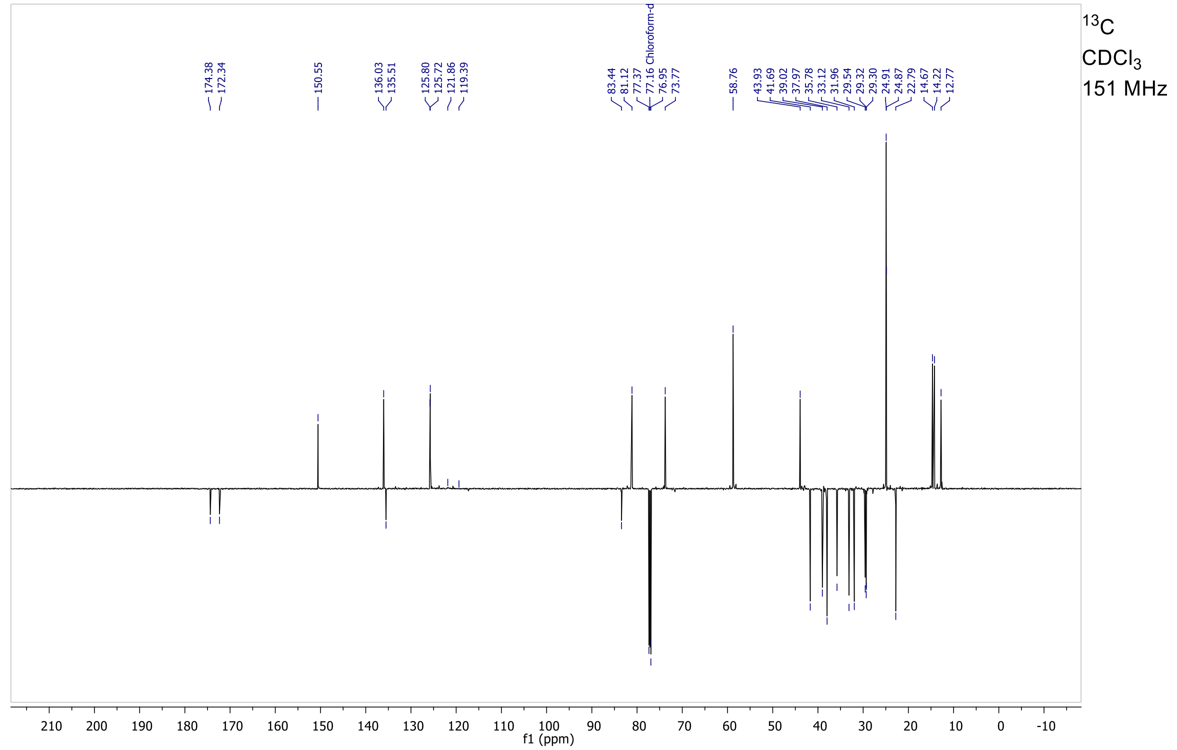

(*R*,4*E*,6*E*)-1-(((2*R*,3*S*)-2-Methoxy-3-methyl-4-oxo-4-(((*E*)-4-(4,4,5,5-tetramethyl-1,3,2-dioxaborolan-2-yl)but-3-en-1-yl)amino)butyl)amino)-4-methyl-1-oxotetradeca-4,6-dien-3-yl (1*R*,4*R*)-4-chlorocyclobut-2-ene-1-carboxylate (S-93a)

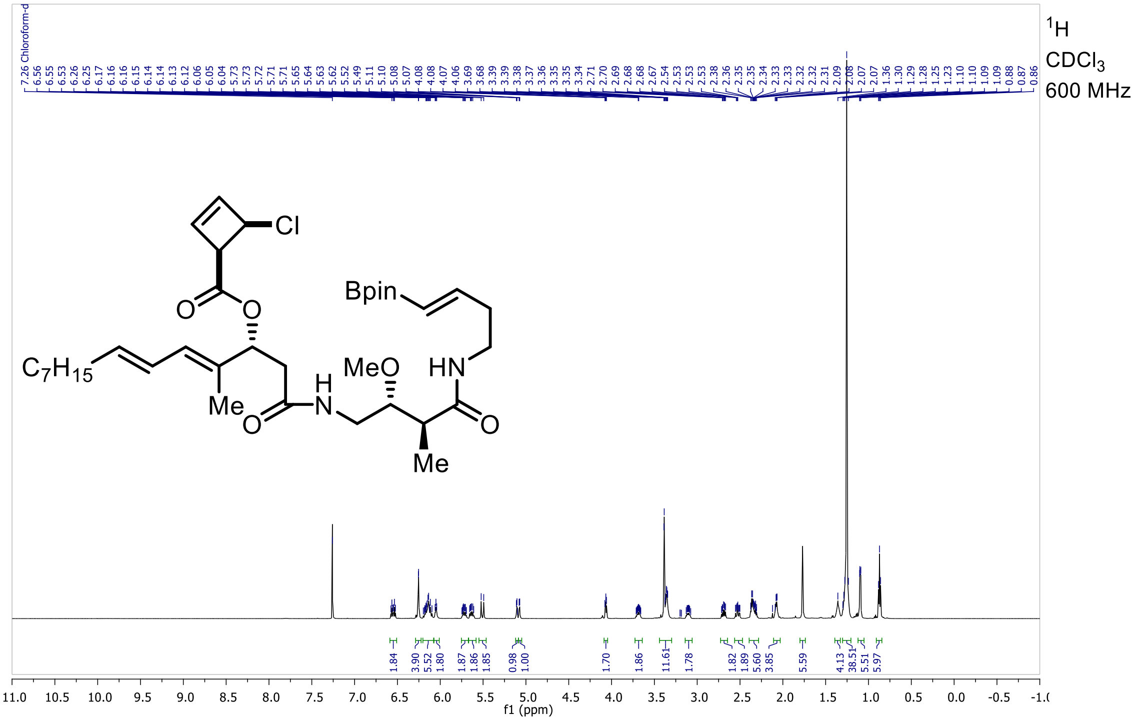


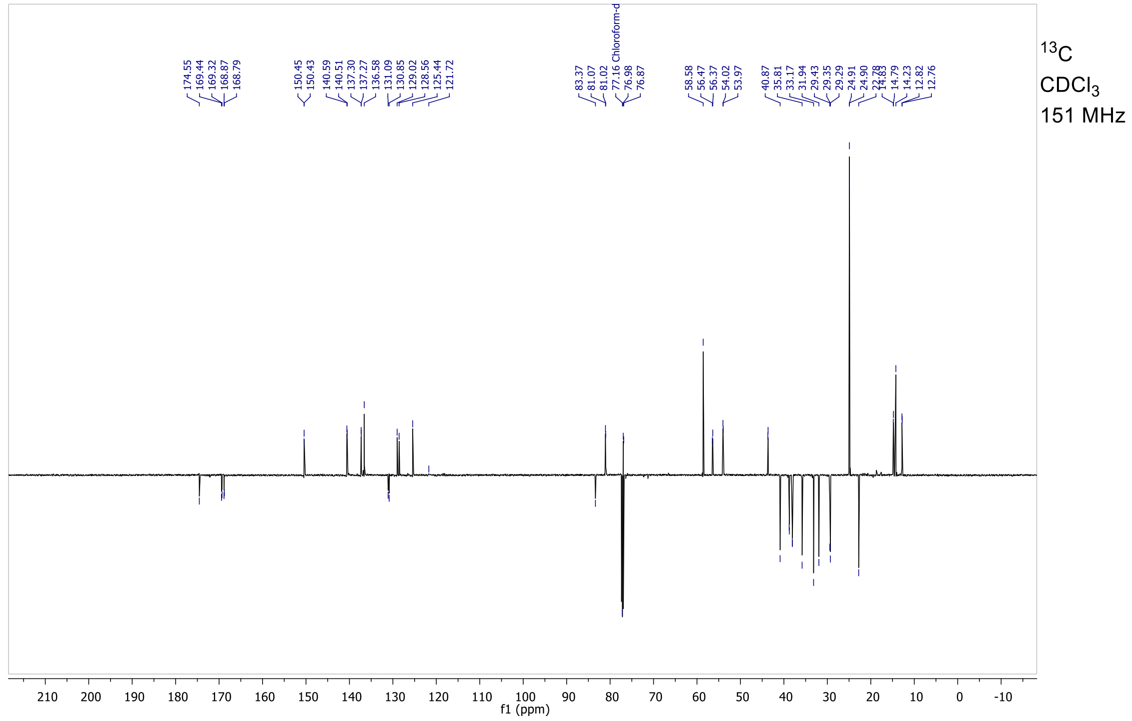

Fs-FR10

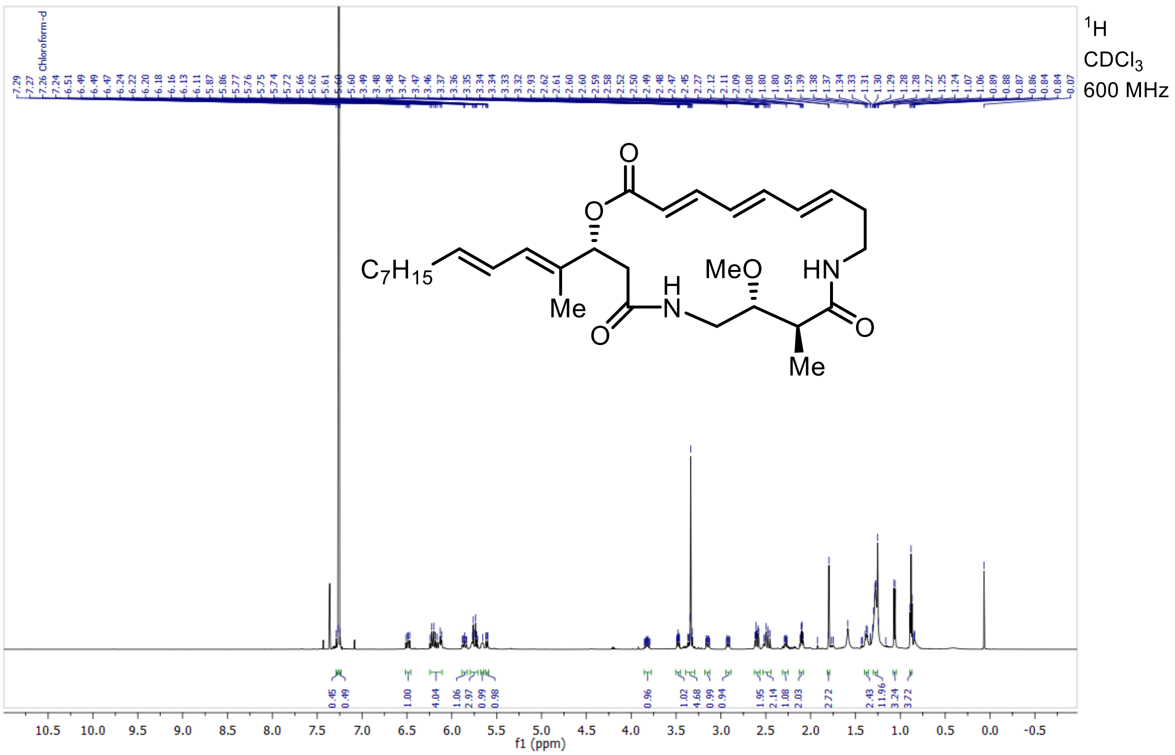


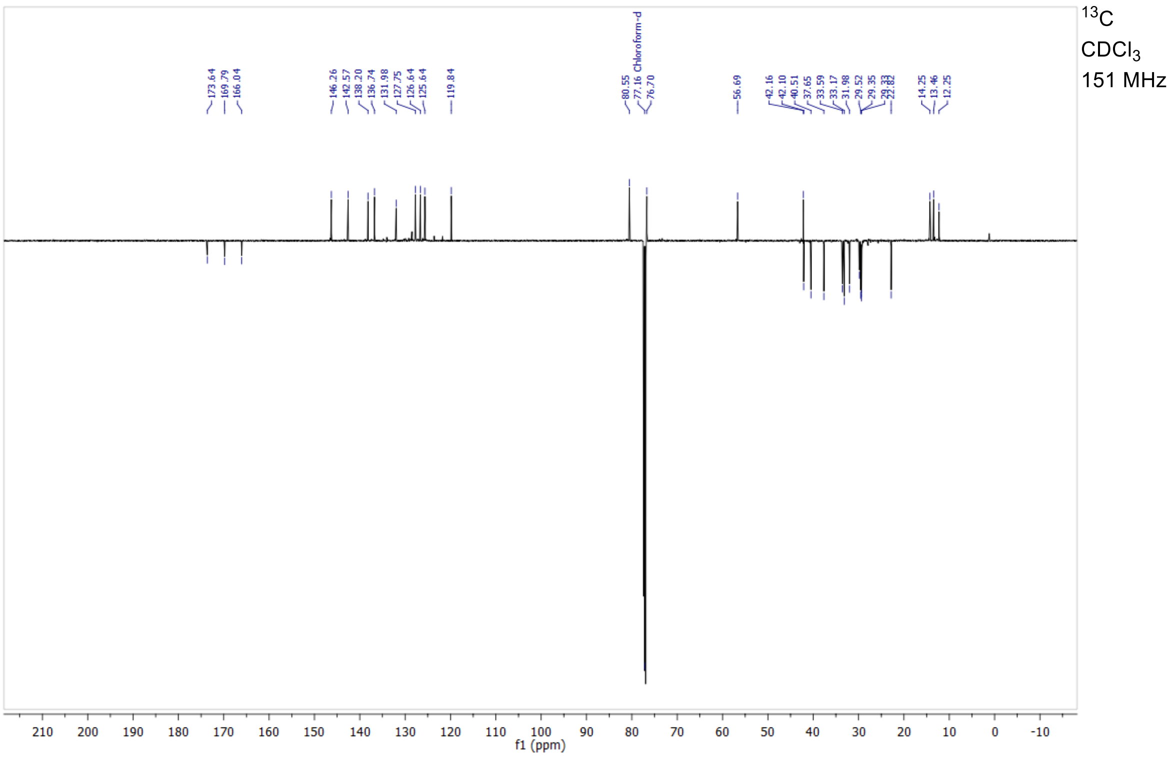

# Methyl (*E*)-5-iodopent-4-enoate (S-94)

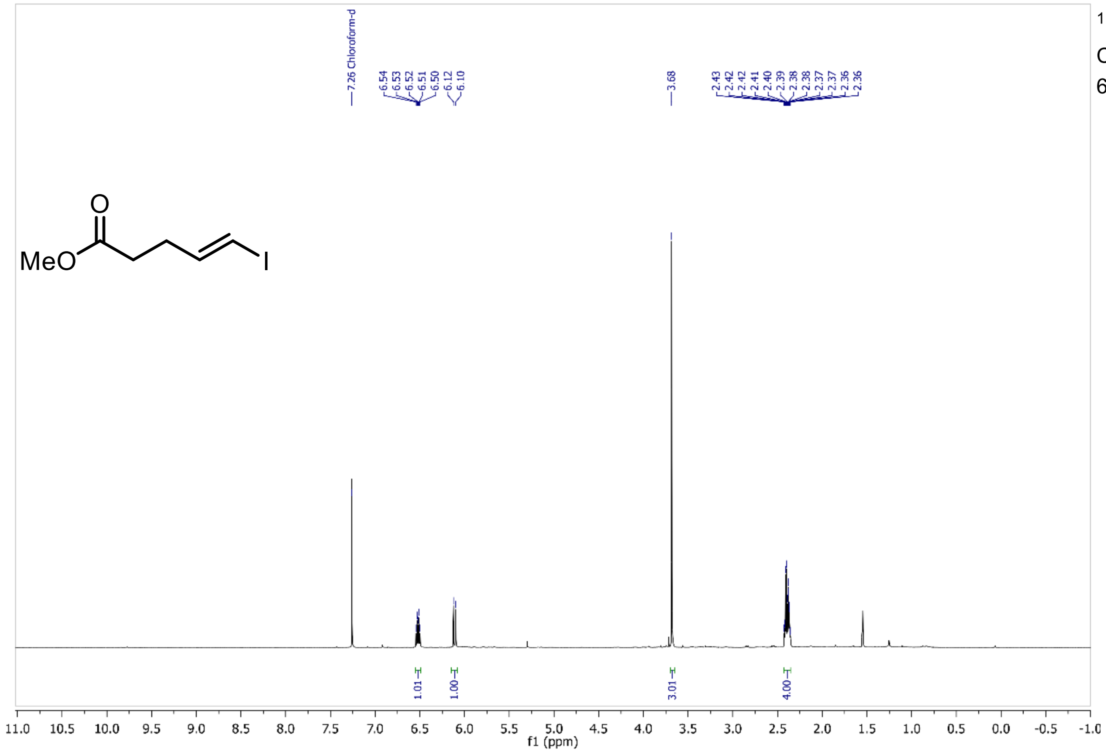


$^1$H
$CDCl_3$
600 MHz

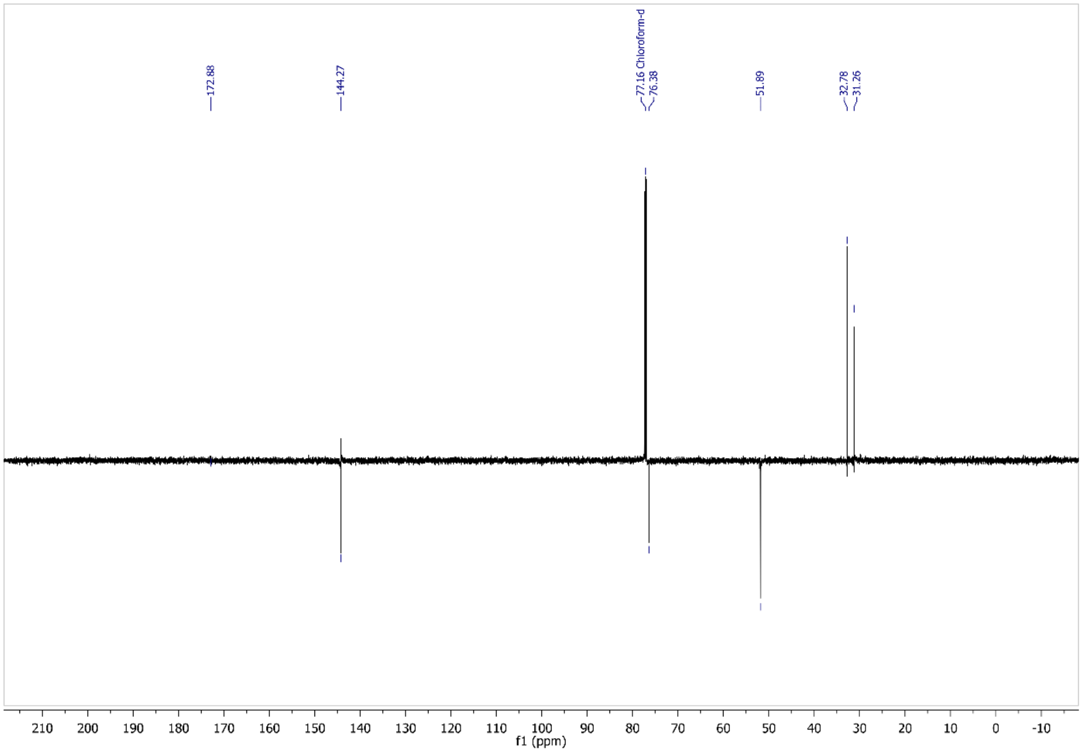


$^{13}$C
$CDCl_3$
151 MHz

Methyl (4*E*,6*E*)-9-((2*S*,3*R*)-3-((*tert*-butyldimethylsilyl)oxy)-4-((*R*,4*E*,6*E*)-3-hydroxy-4-methyl-tetradeca-4,6-dienamido)-2-methylbutanamido)nona-4,6-dienoate (S-95)

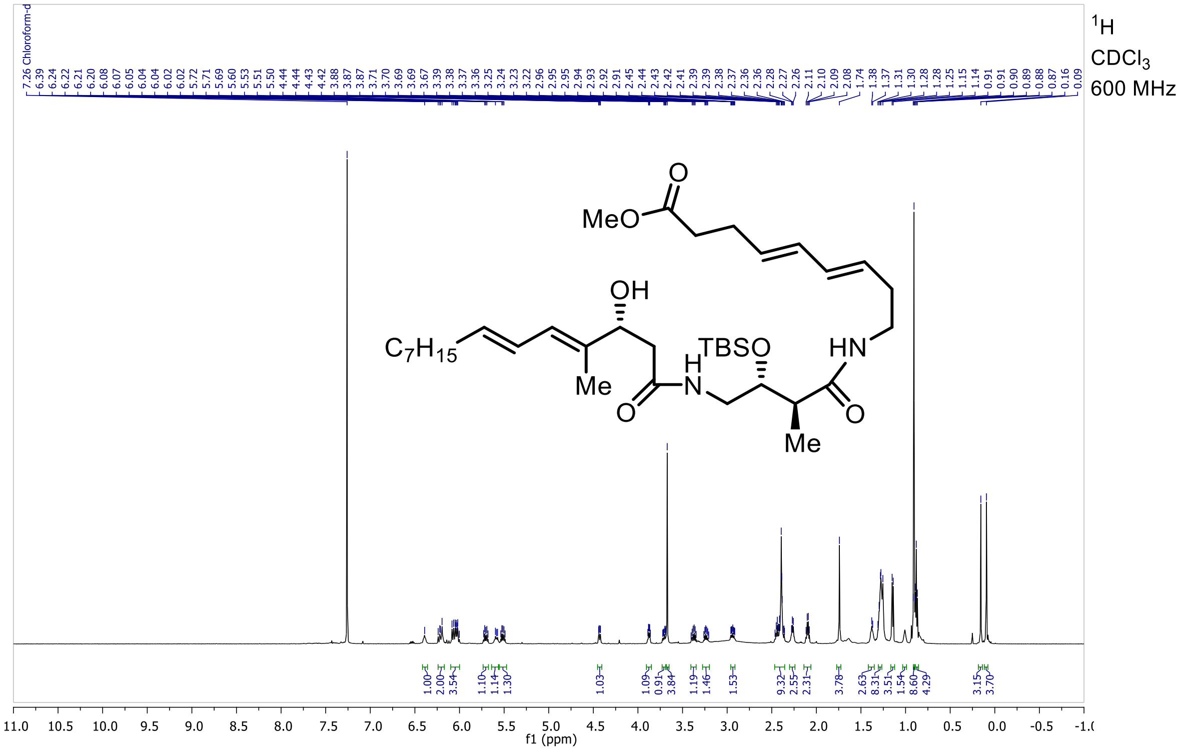


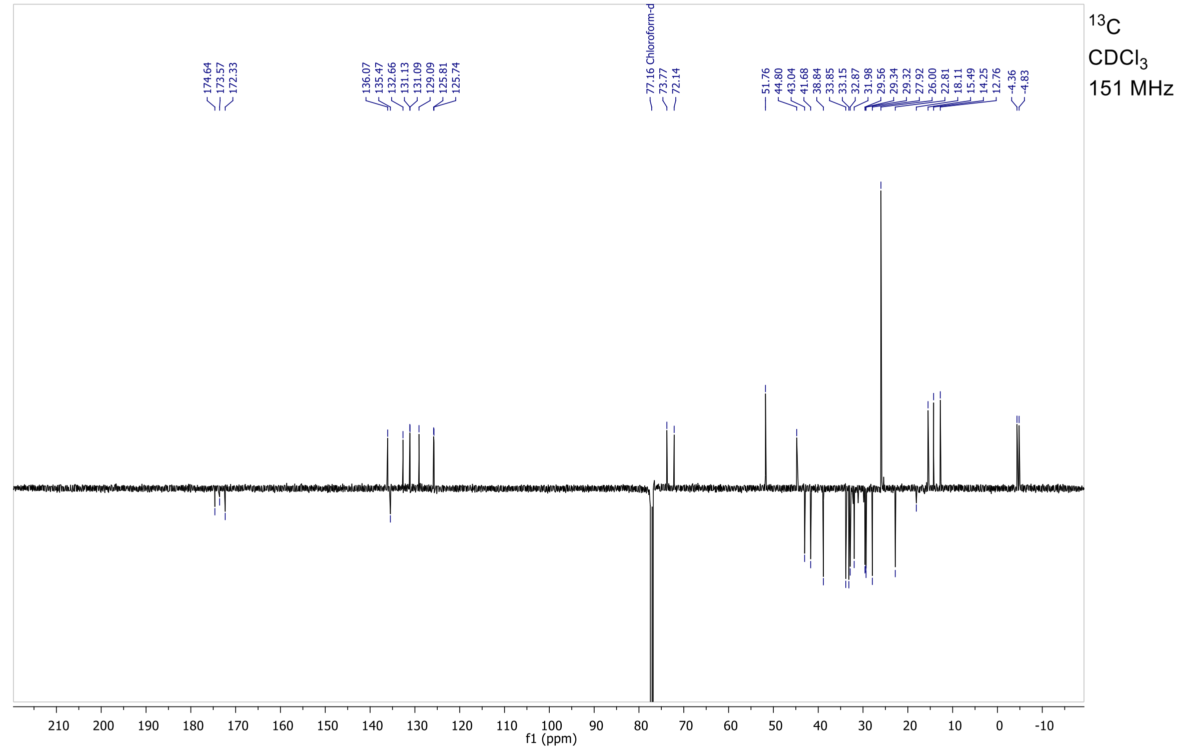

(2*R*,7*R*,8*S*,13*E*,15*E*)-7-((*tert*-Butyldimethylsilyl)oxy)-2-((2*E*,4*E*)-dodeca-2,4-dien-2-yl)-8-methyl-1-oxa-5,10-diazacyclononadeca-13,15-diene-4,9,19-trione (S-97)

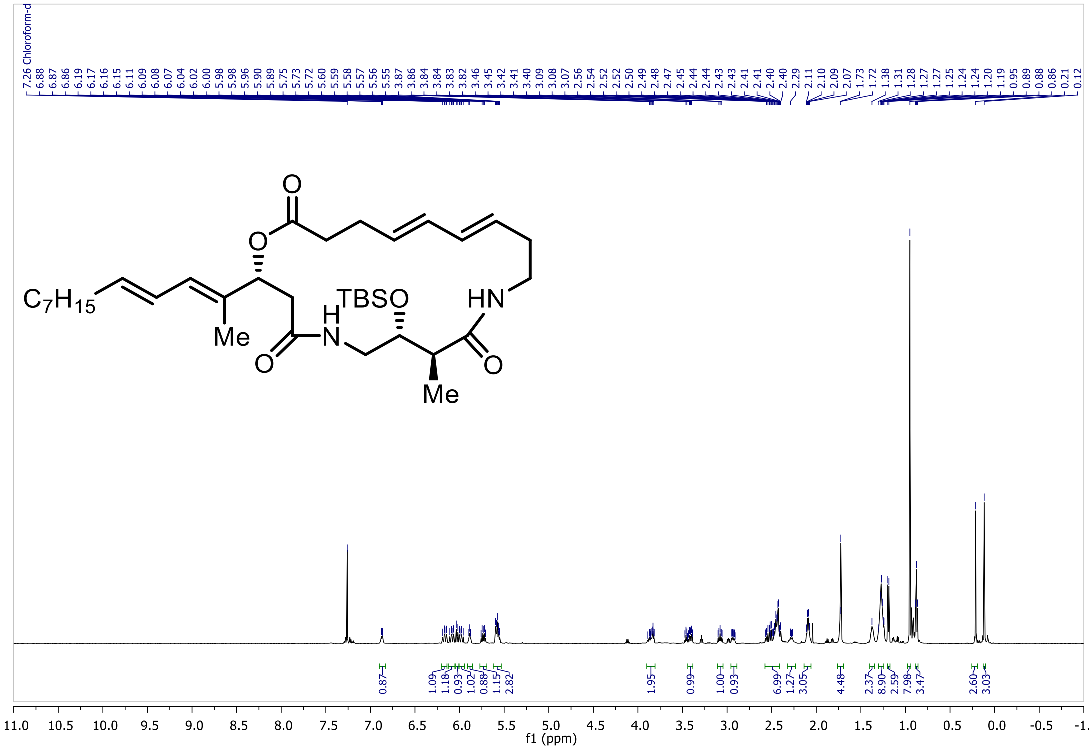


$^{1}H$
$CDCl_3$
600 MHz

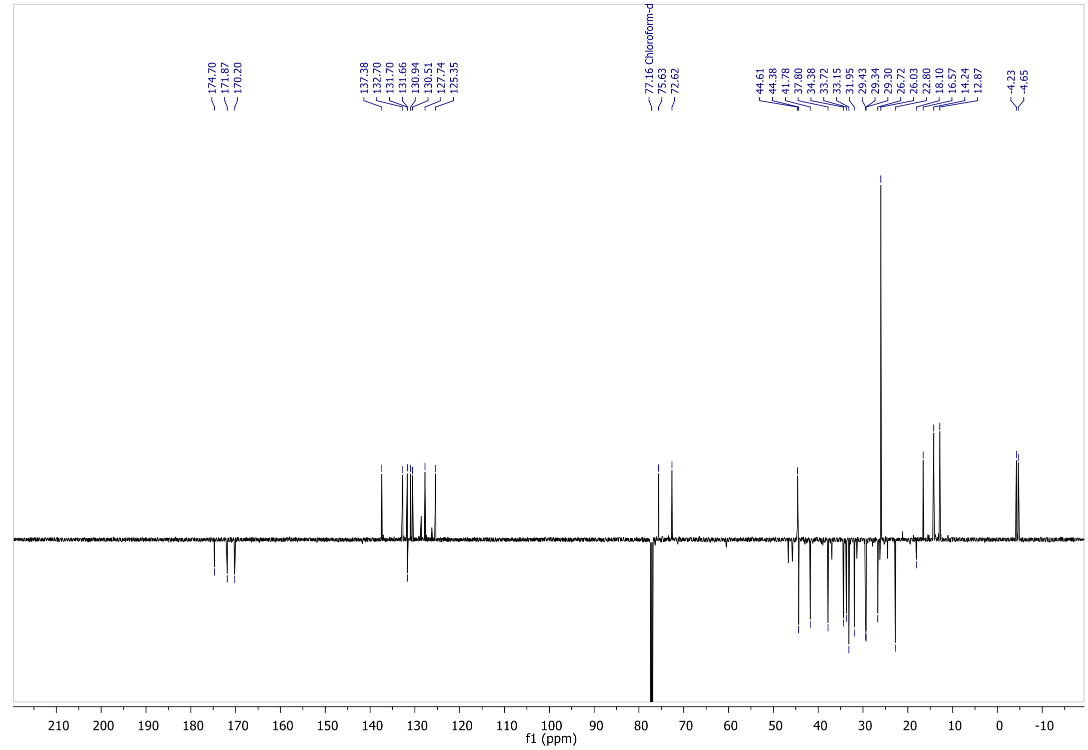


$^{13}C$
$CDCl_3$
151 MHz

Fs-FR12

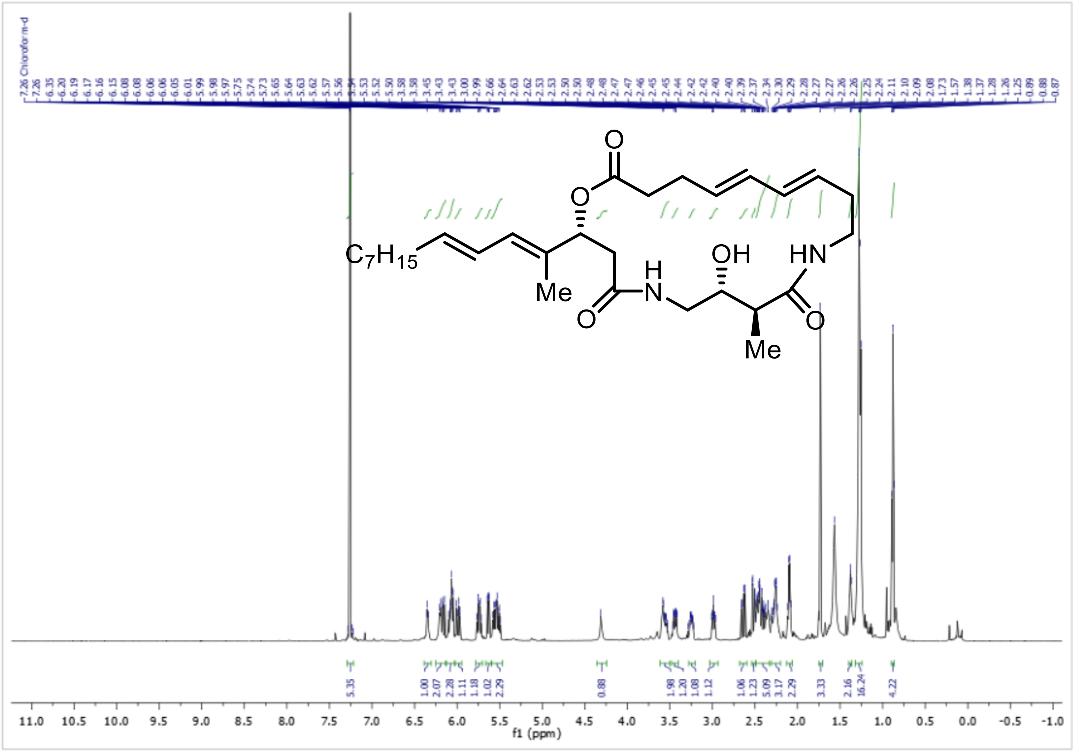



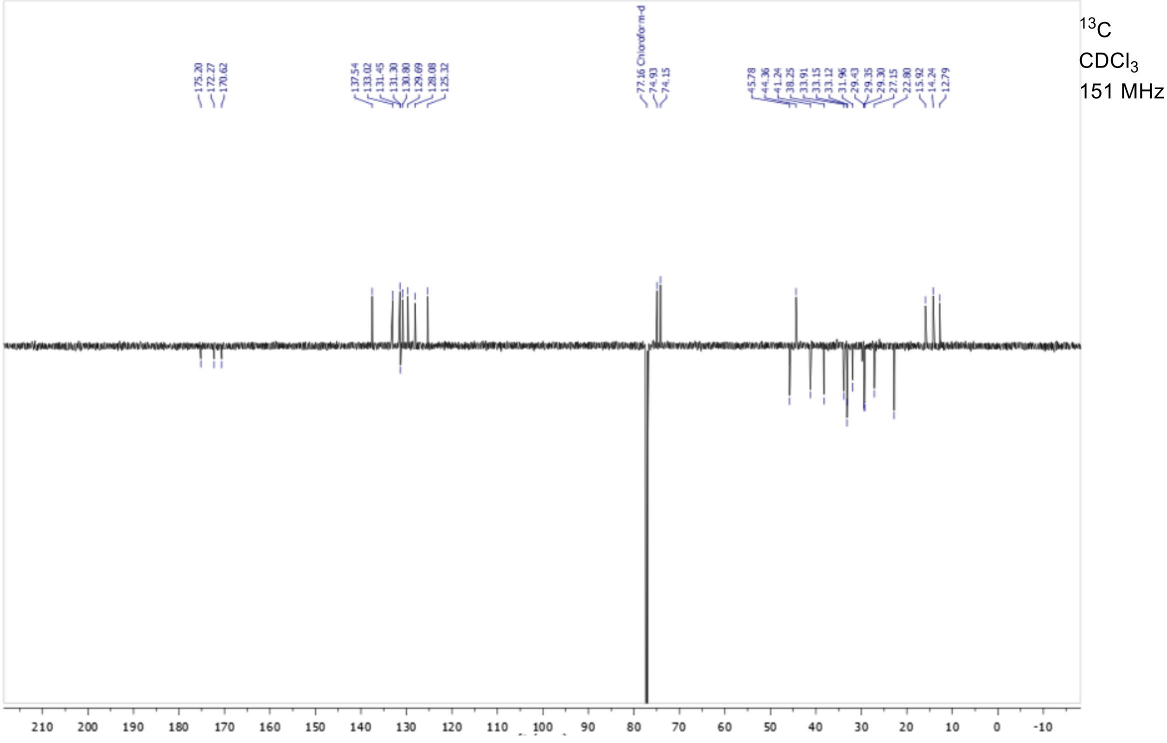

# Methyl 9-((tert-butoxycarbonyl)amino)nonanoate (S-100)

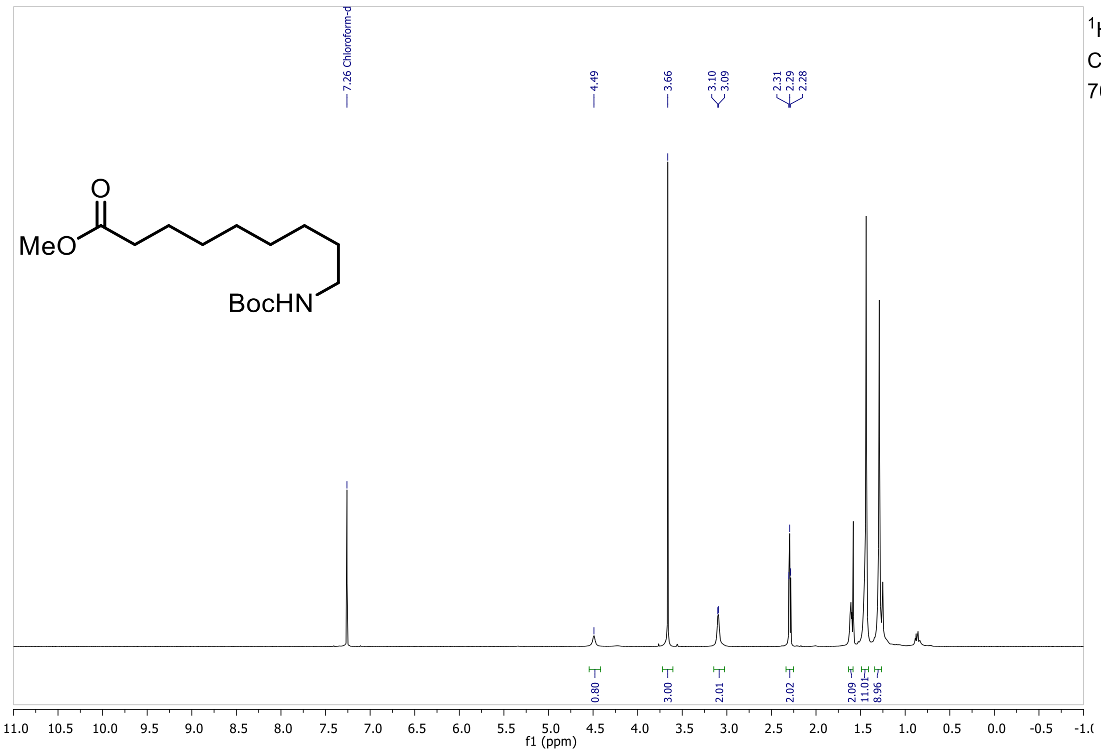


$^1H$
$CDCl_3$
700 MHz

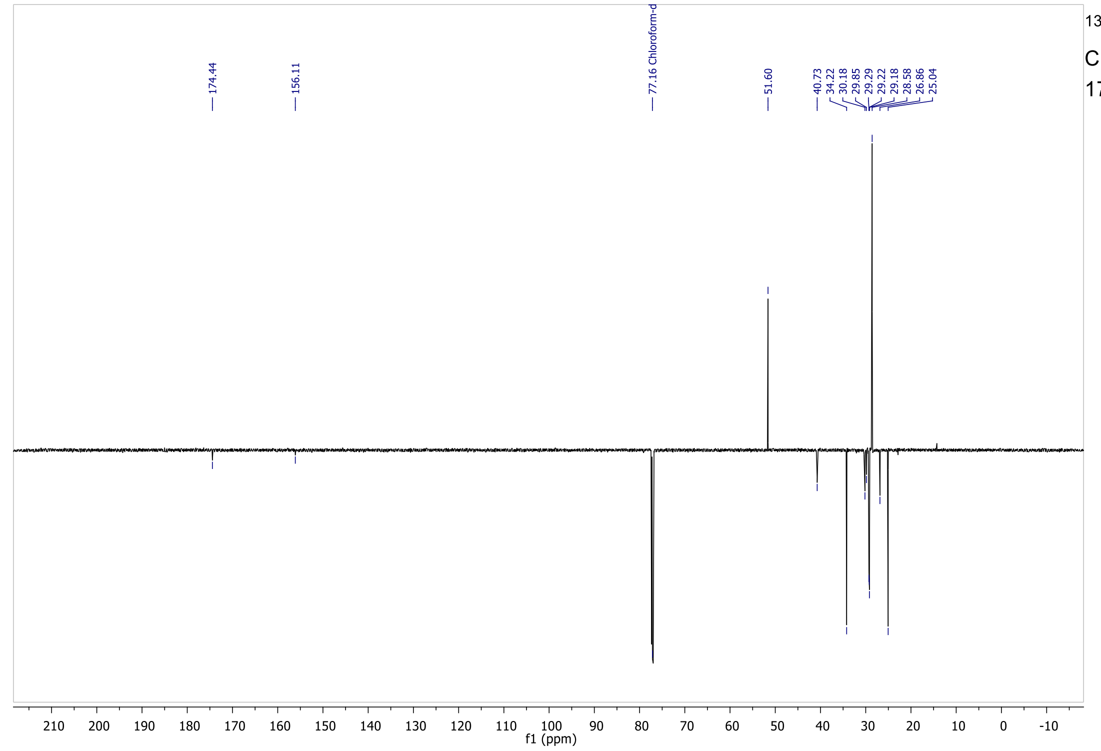


$^{13}C$
$CDCl_3$
176 MHz

## 9-Methoxy-9-oxononan-1-aminium 2,2,2-trifluoroacetate (S-101)

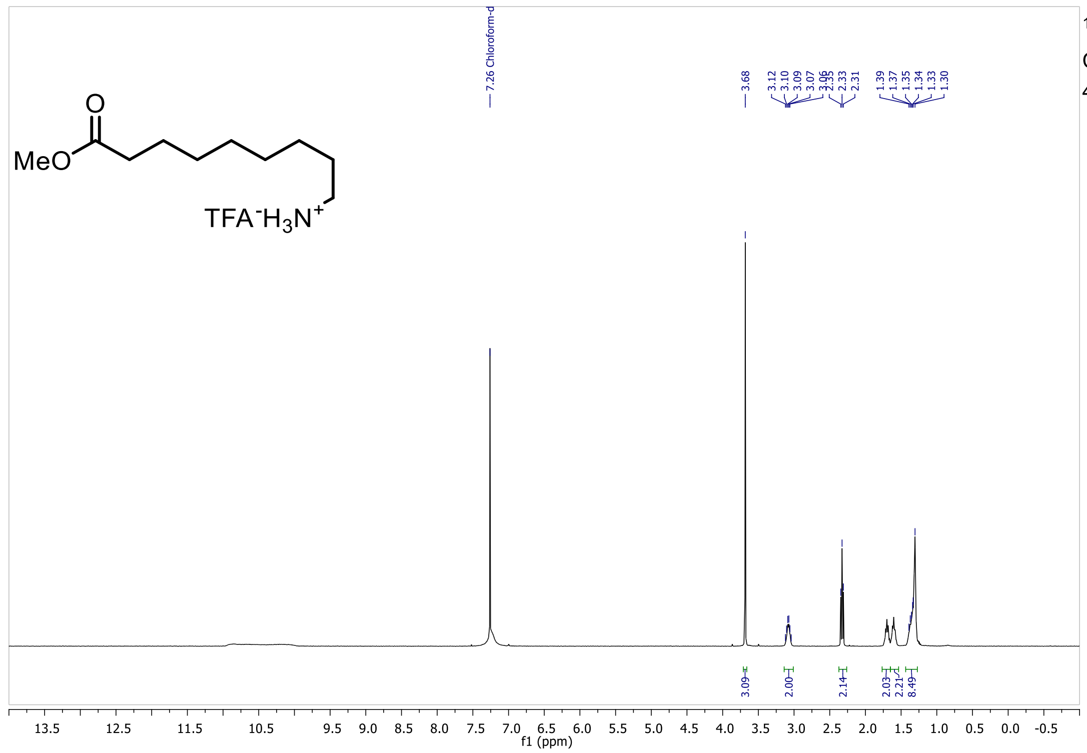


$^1H$
$CDCl_3$
400 MHz

Methyl 9-((2*S*,3*R*)-4-azido-3-((*tert*-butyldimethylsilyl)oxy)-2-methylbutan-amido)nonanoate (S-102)

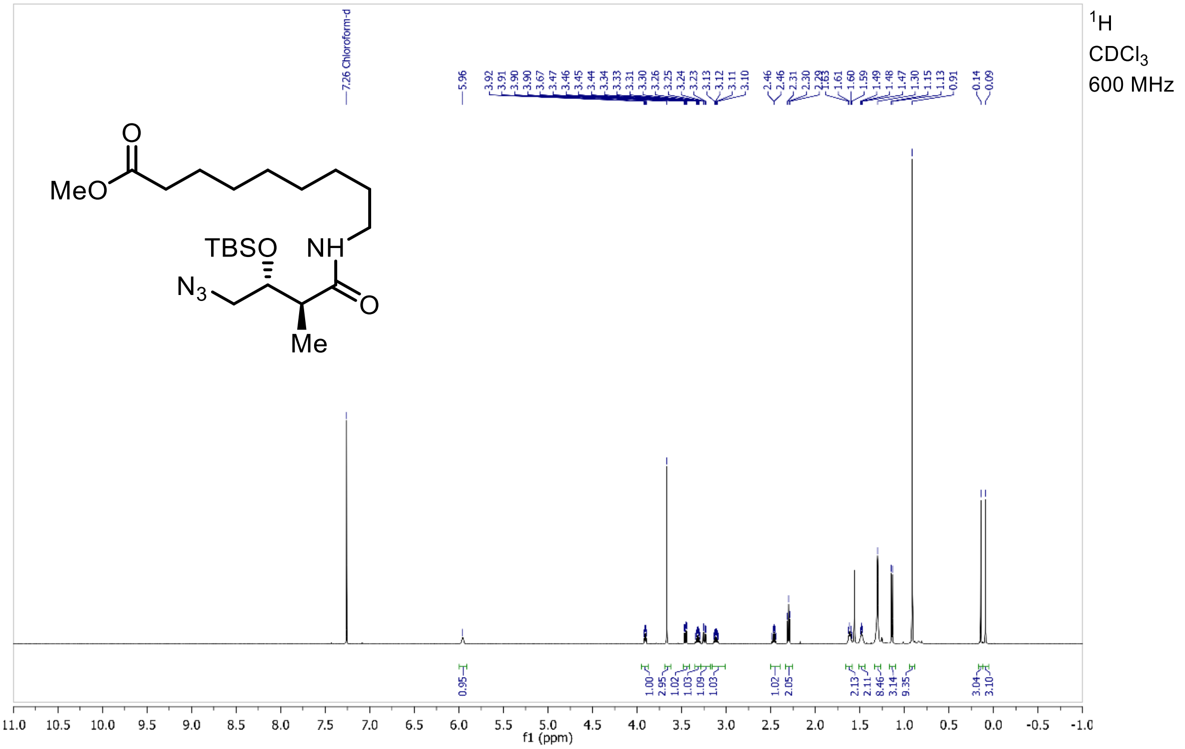


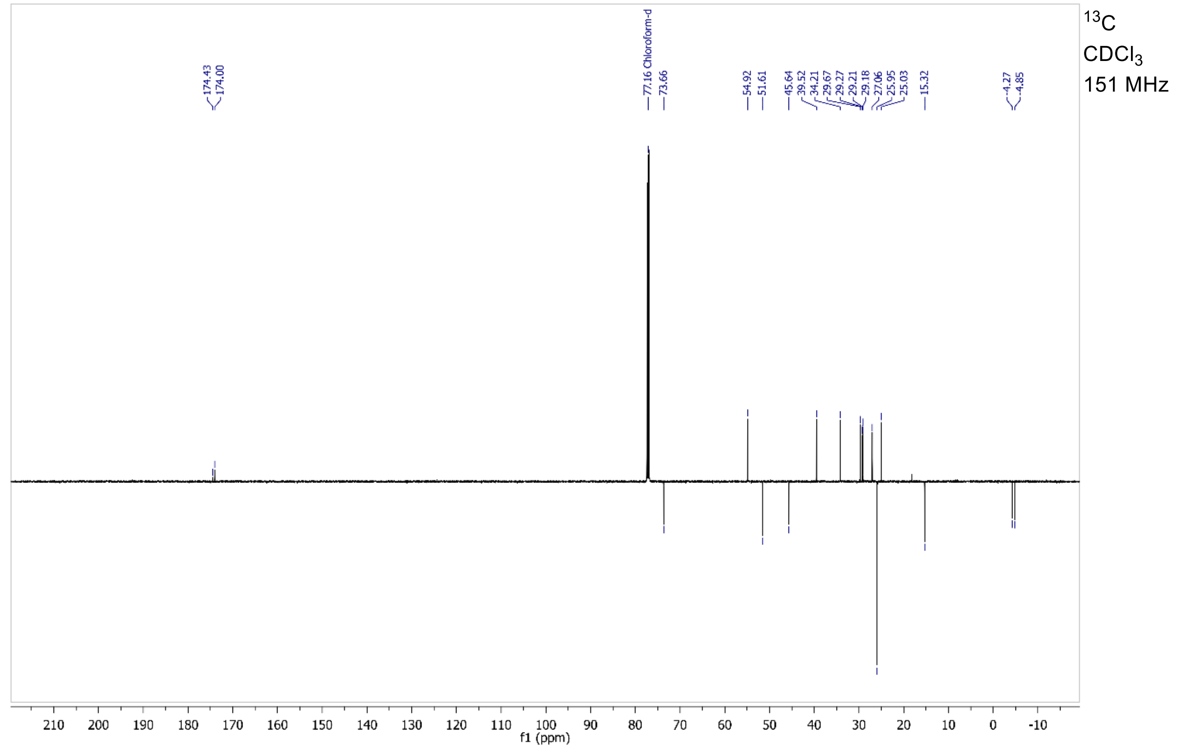

Methyl 9-((2*S*,3R)-3-((*tert*-butyldimethylsilyl)oxy)-4-((*R*,4*E*,6*E*)-3-hydroxy-4-methyltetra-deca-4,6-dienamido)-2-methylbutanamido)nonanoate (S-104)

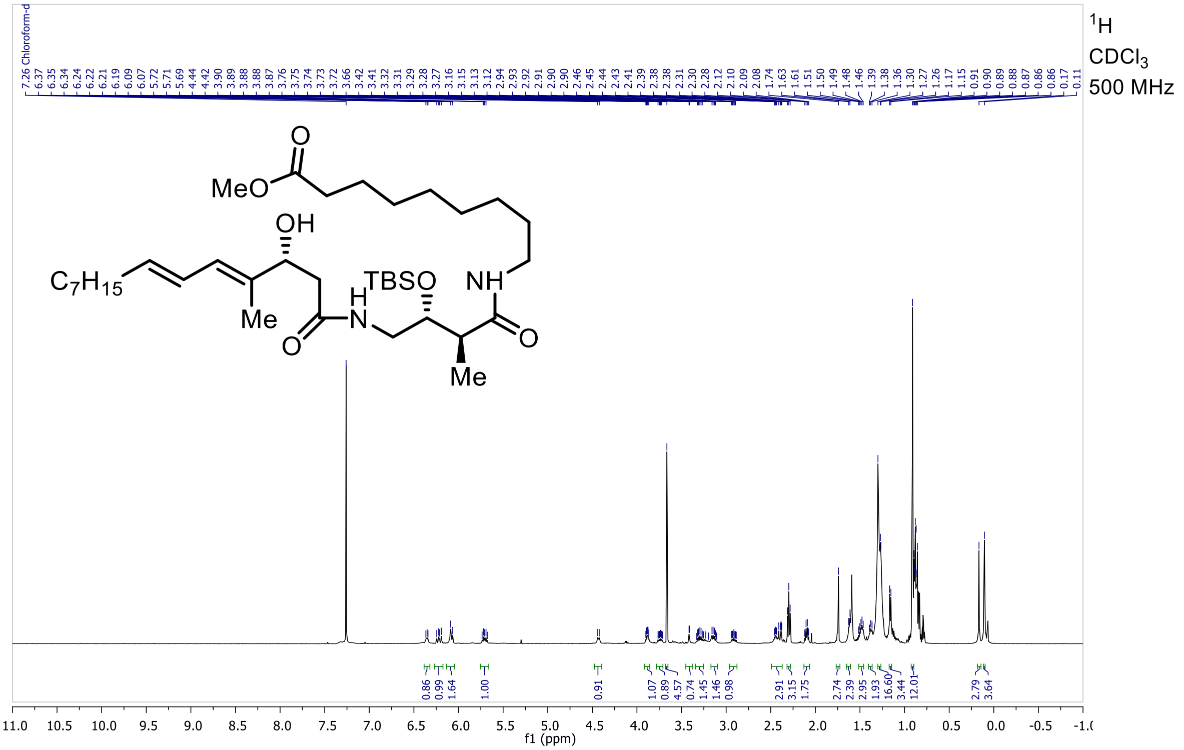


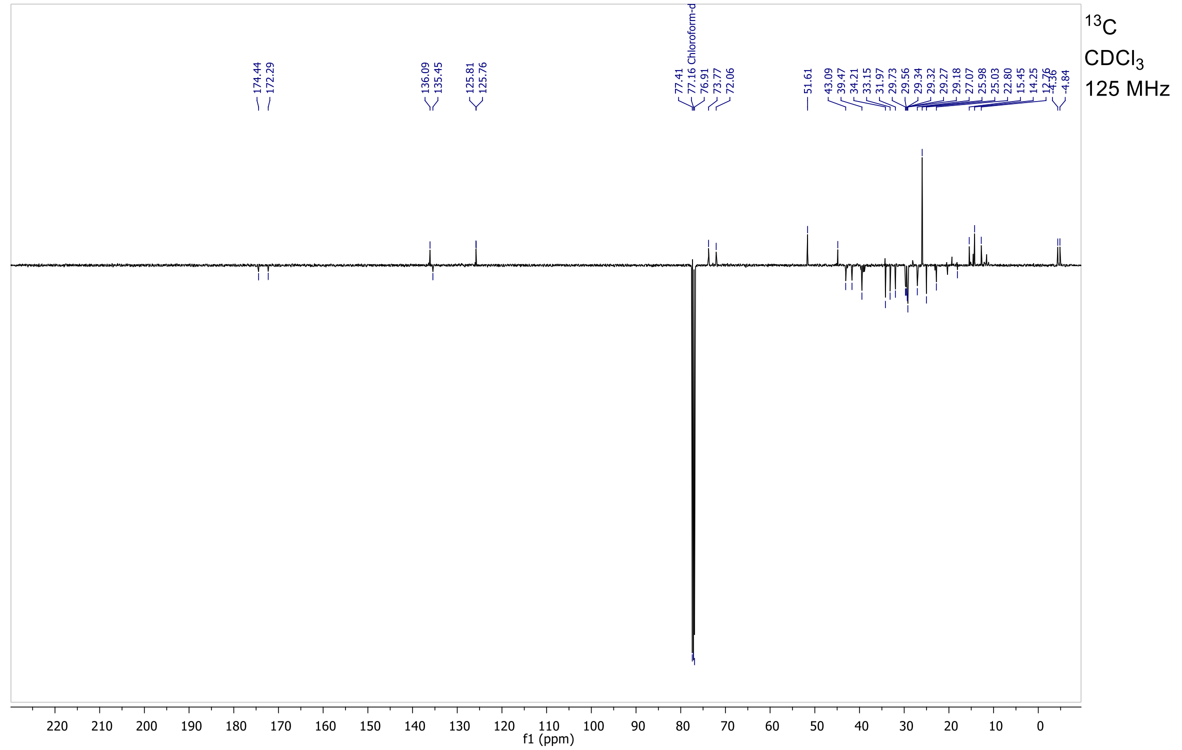

## 9-((2*S*,3*R*)-3-((*tert*-Butyldimethylsilyl)oxy)-4-((*R*,4*E*,6*E*)-3-hydroxy-4-methyltetradeca-4,6-dienamido)-2-methylbutanamido)nonanoic acid (S-105)

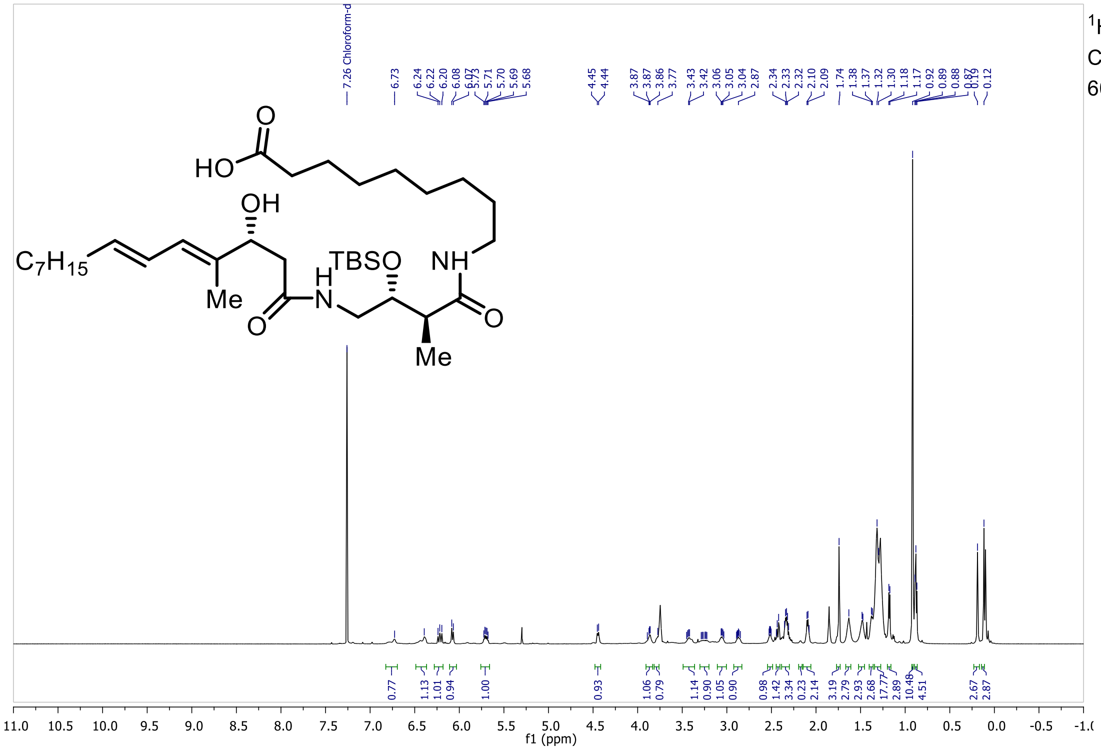

## (2*R*,7*R*,8*S*)-7-((*tert*-Butyldimethylsilyl)oxy)-2-((2*E*,4*E*)-dodeca-2,4-dien-2-yl)-8-methyl-1-oxa-5,10-diazacyclononadecane-4,9,19-trione (S-106)

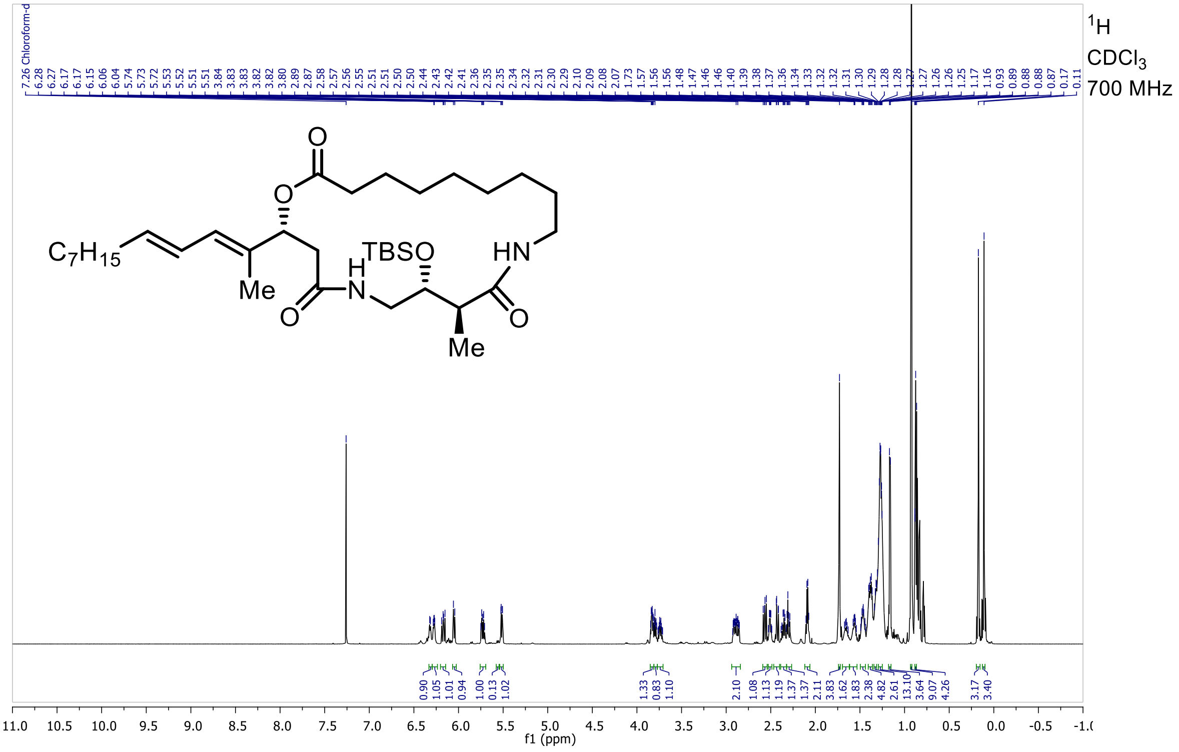


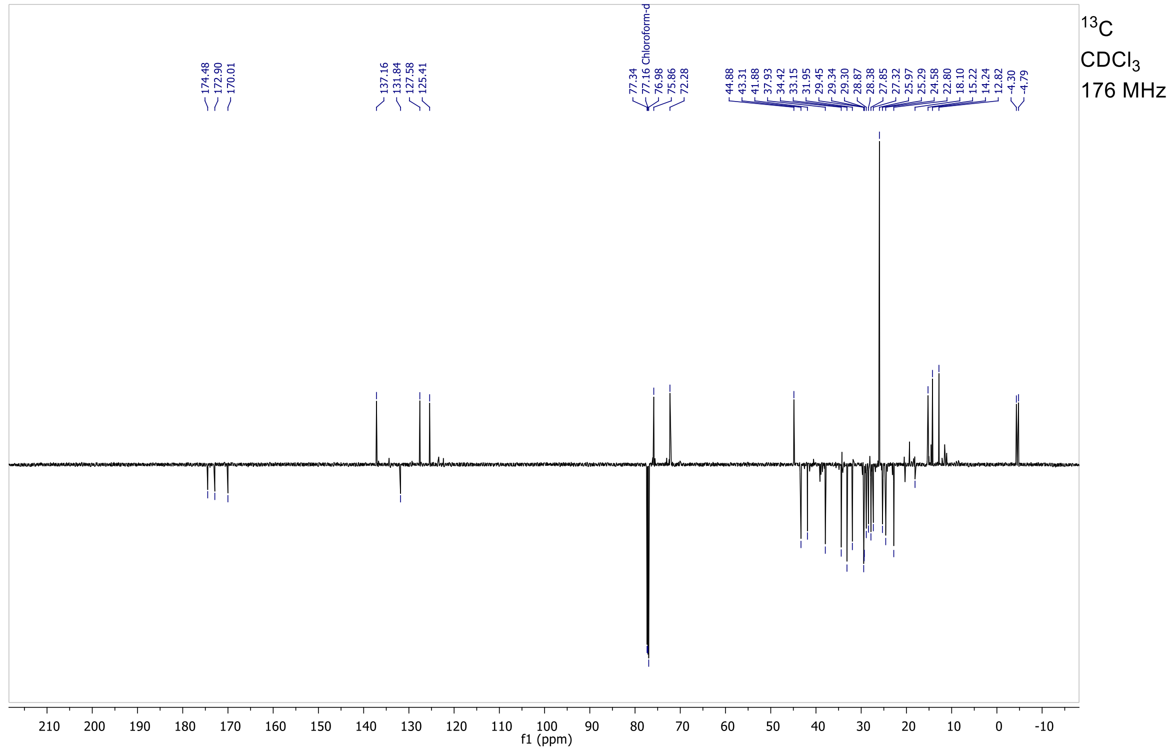

Fs-FR13

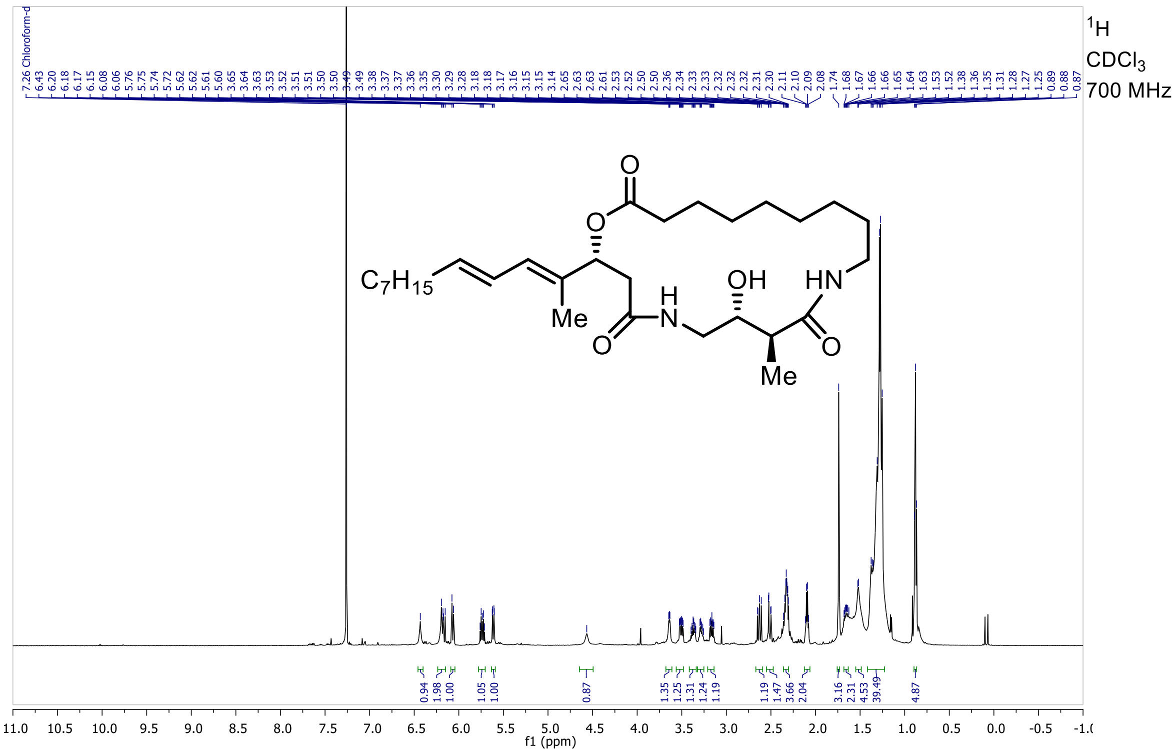

$^1H$
$CDCl_3$
700 MHz
$C_7H_{15}$
Me
OH
HN
H
N
O
Me
f1 (ppm)


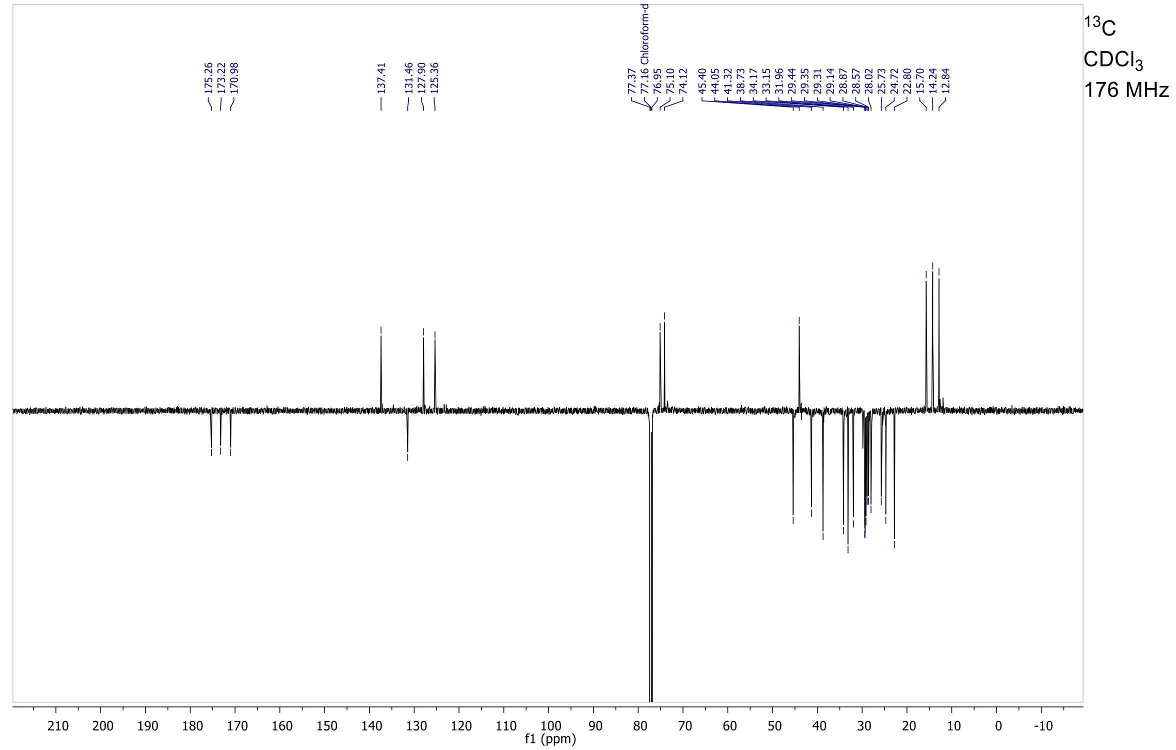

$^{13}C$
$CDCl_3$
176 MHz
175.26
173.22
170.98
137.41
131.46
127.90
125.36
77.37
77.16 Chloroform-d
76.95
75.10
74.12
45.40
44.05
41.32
38.73
34.17
33.15
31.96
29.44
29.35
29.31
29.14
28.87
28.57
28.02
25.73
24.72
22.80
15.70
14.24
12.84
f1 (ppm)

## (±)-*cis*-Methyl-4-chlorocyclobut-2-ene-1-carboxylate ((*rac*)-S-107)

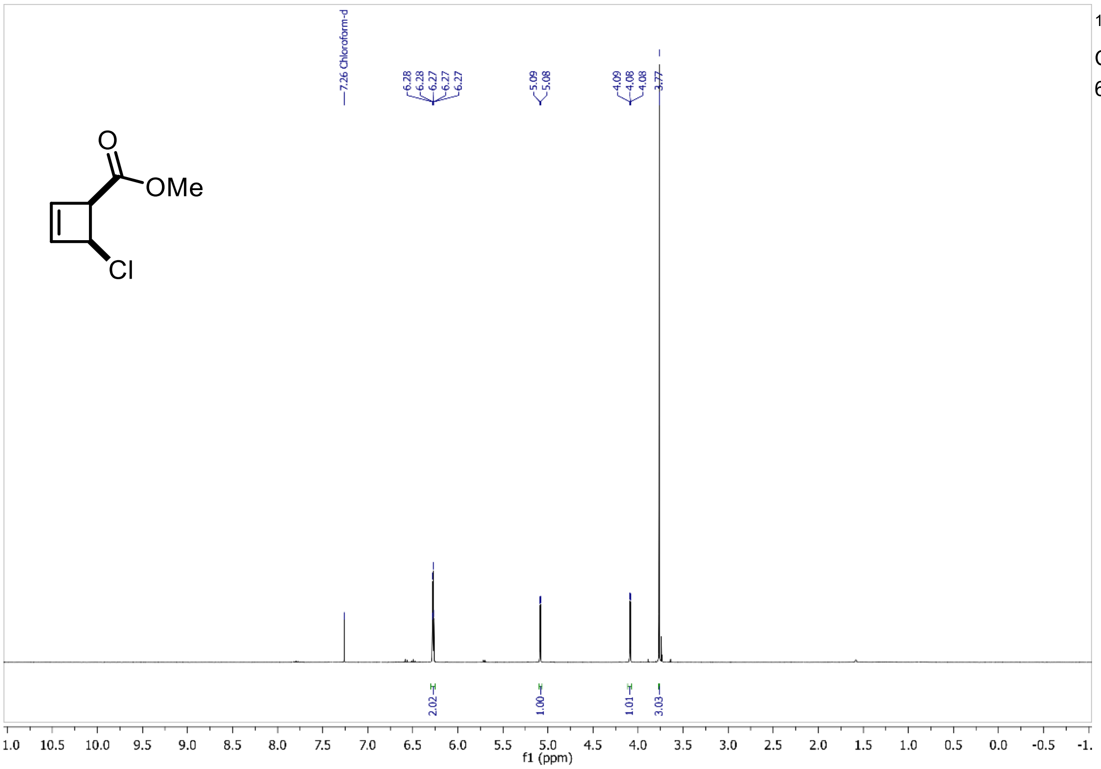


$^{1}H$
$CDCl_3$
600 MHz

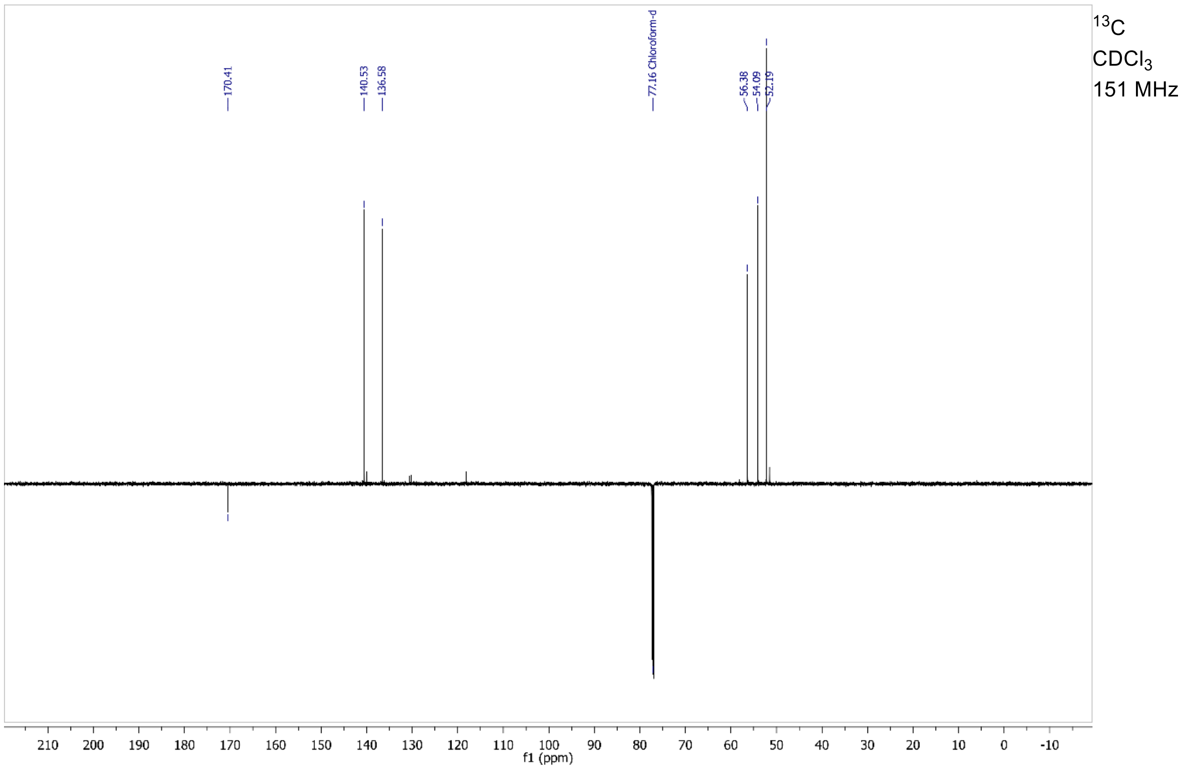


$^{13}C$
$CDCl_3$
151 MHz

Methyl-(2E,4E,6E)-9-((2S,3R)-3-((tert-butyldimethylsilyl)oxy)-4-((R,4E,6E)-3-hydroxy-4-methyltetradeca-4,6-dienamido)-2-methylbutanamido)nona-2,4,6-trienoate (S-108)

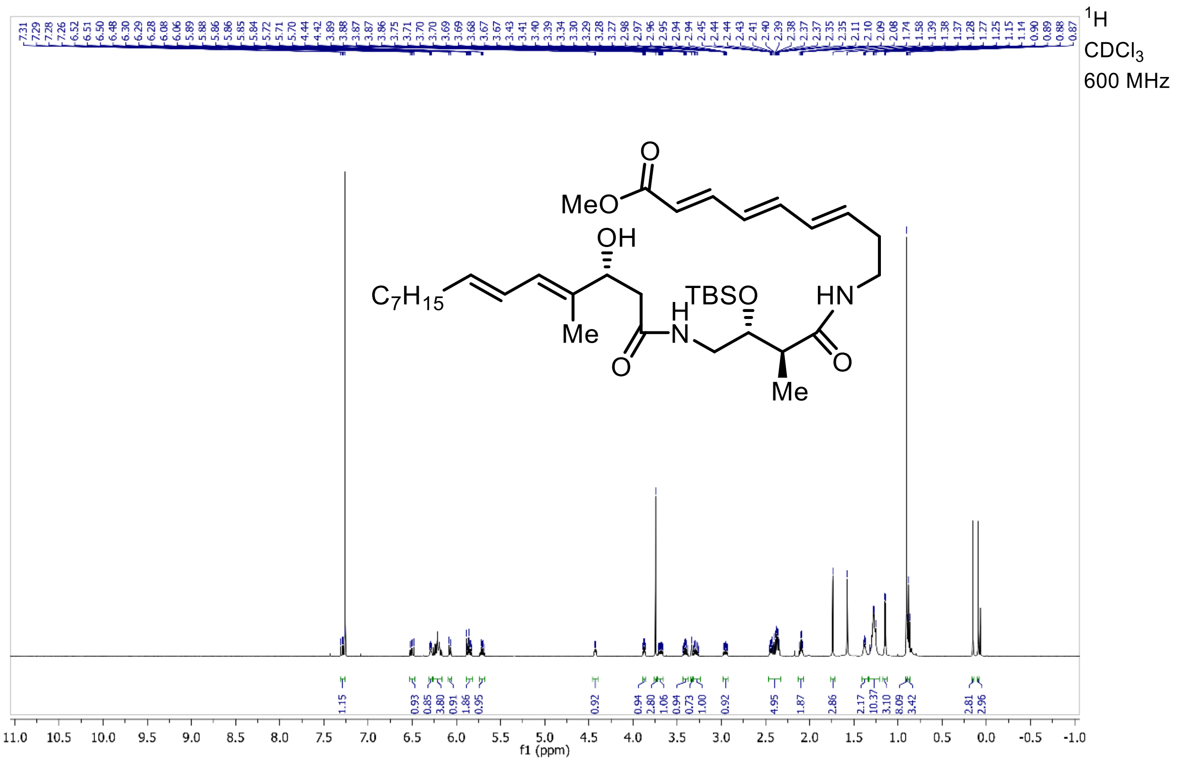


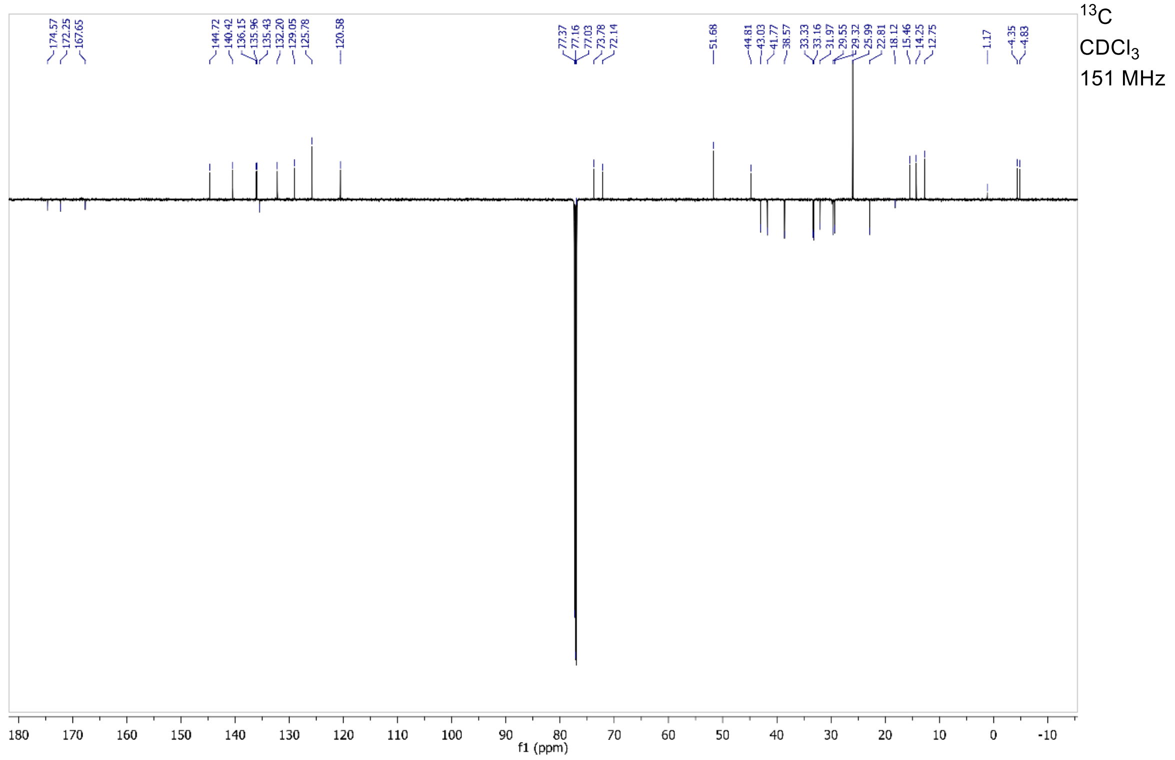

Methyl-(2E,4E,6E)-9-((2S,3R)-3-hydroxy-4-((R,4E,6E)-3-hydroxy-4-methyltetradeca-4,6-dienamido)-2-methylbutanamido)nona-2,4,6-trienoate (S-109)

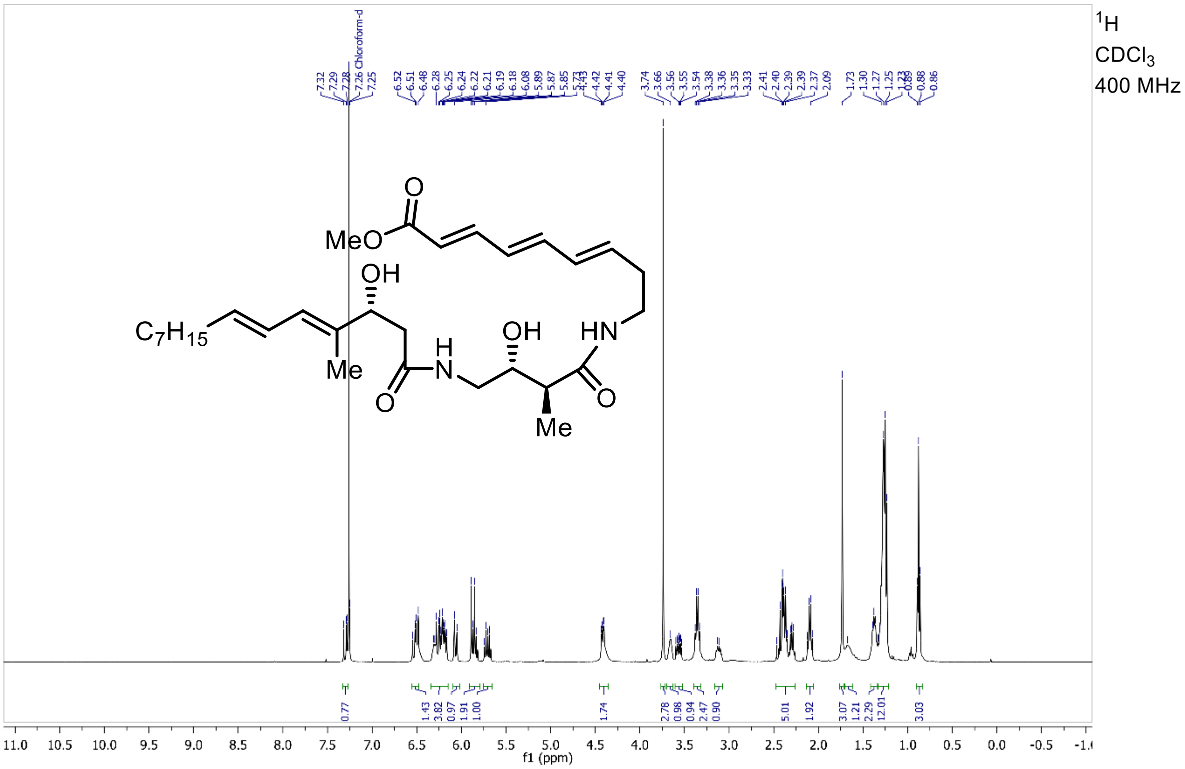

Fs-FR14

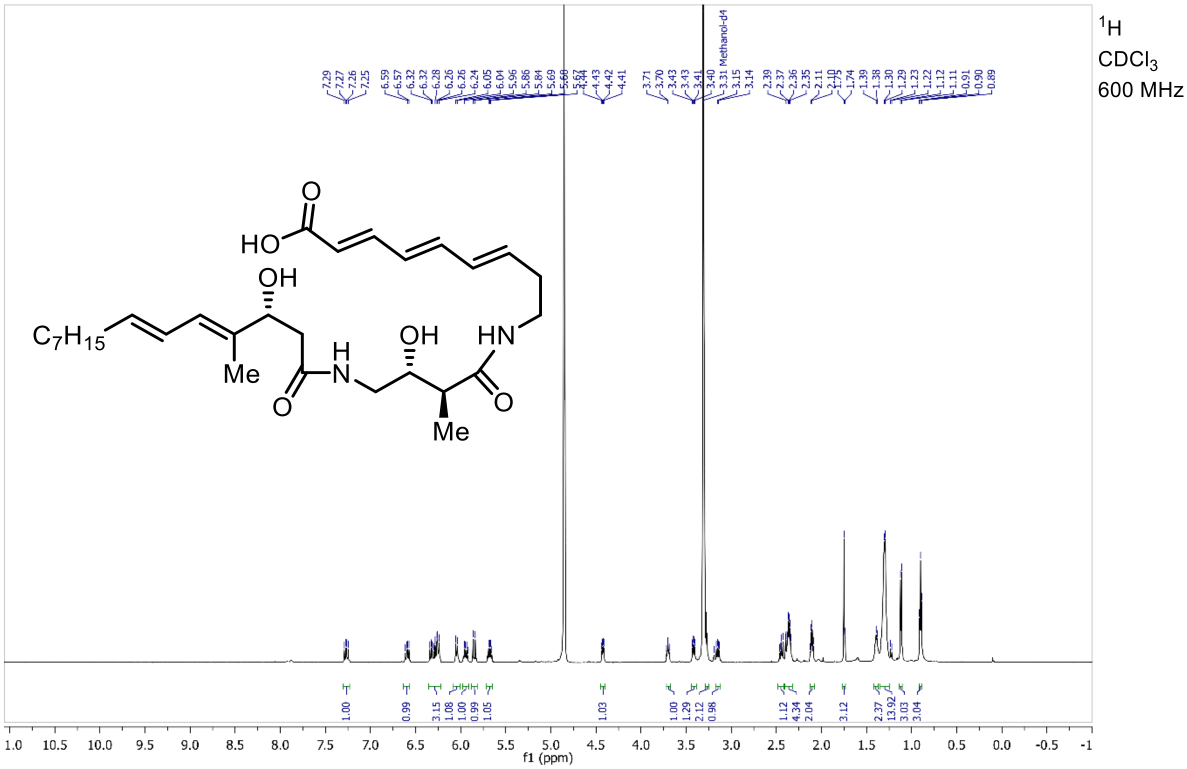

# 7. Immunological Evaluation

## 7.1. EL4 T cell proliferation assay

EL4 T cells (ATCC® TIB-39™) were cultured in Dulbecco's Modified Eagle Medium (DMEM, Thermo Fisher Scientific, D5030-10L) supplemented with 10% heat inactivated fetal bovine serum (FBS, Thermo Fisher Scientific, 10500).

Compounds were dissolved in DMSO (16 mM) and were dispensed with a Labcyte Echo 550 to 96-well plates (Corning, CLS3904-100EA). For each compound multiple volumes were plated to achieve six final assay concentrations ranging from 20 µM to 6.4 nM in 5-fold dilutions. The volume of DMSO in each well was adjusted to 0.25 µL. Eight wells per plate were filled with DMSO only to serve as negative control. 2000 EL4 T cells in 100 µL of medium were plated per well and after 72 h of incubation, cell viability was measured using a CellTiter-Glo Luminescent Cell Viability Assay (Promega, G7572). After equilibration of the plates and the reagent to room temperature, the Cell-Titer-Glo reagent was added to the wells using a Multidrop™ Combi Reagent Dispenser (Thermo Fisher Scientific) and cells were lysed by shaking. The luminescence signal was read after 20 min incubation at room temperature using an EnVision™ Multilabel Plate Reader (PerkinElmer). Signals were normalised to negative control wells on each plate and dose-response curves and $IC_{50}$ values were calculated using GraphPad Prism 7.

## 7.1. Peripheral blood mononuclear cell (PBMC) assay

Blood cells for PBMC isolation were obtained from healthy volunteers at the Department of Transfusion Medicine and Cell Therapy after informed written consent, in line with national law and approved by the ethics committee of the Medical University of Vienna. Peripheral blood mononuclear cells (PBMC) were isolated from whole blood of healthy donors on a Ficoll gradient and resuspended in RPMI (Gibco, #52400-025). Subsequently, $2.5 \cdot 10^5$ cells were seeded per well in a 96-well U-bottom plate and treated with the corresponding compounds at the desired concentration. DMSO (Sigma-Aldrich, #41639) was added to FR-untreated wells as solvent control. Cells were incubated with the compounds for 30 min at 37 °C under an atmosphere containing 5% $CO_2$. Following incubation, the cells were stimulated by adding 1X Cell Activation Cocktail (CAC) containing Brefeldin A (Biolegend, #423303), or 1X Brefeldin A (Biolegend, #420601) only in negative controls. Cells were incubated for stimulation at 37 °C under an atmosphere containing 5% $CO_2$ for 3 h. Afterwards, the cells were harvested and stained following a procedure described by Strobl *et al*.[8]

In short, cells were stained for 15 min at 20 °C in the dark with a fixable viability dye (eBioscience Fixable Viability Dye eFluor 780; ThermoFisher, #65-0865-14 or Zombie UV Fixable Viability Kit; Biolegend, #423107) and subsequently stained for 30 min at 4 °C with surface marker antibodies (Table S1). After fixation and permeabilization (Fixation Buffer, Biolegend #420801; Intracellular Staining Permeabilization Wash Buffer (10X), Biolegend #421002) cells were stained for 25 min at 20 °C with intracellular cytokine antibodies (Table S 1). Washing was performed between each staining step. Stained cells were acquired on a CYTEK Aurora spectral flow cytometer equipped with five lasers and data analysis was performed with FlowJo v10.8.1 and GraphPad Prism 9.0. Cell subsets were identified by gating on live single cells expressing canonical immune cell markers, including CD45 for leukocytes, CD3 for T cells, CD56 for NK cells and CD20 for B cells. Statistical differences between groups were determined by one-way ANOVA with multiple comparison in GraphPad Prism 9.0.

CD (cluster of differentiation) and HLA-DR are surface markers for different cell types; IFN-γ, IL-6 and TNF-α are intracellular cytokines. BV = Brilliant Violet; AF = AlexaFluor; FVD = Fixable viability dye.

| Antigen (Purpose) | Antibody/Fluorophore | Supplier | Catalogue Nr. | Dilution |
|---|---|---|---|---|
| FVD | eFluor780 | Thermo-Fisher | 65-0865-14 | 1:2000 |
| Zombie UV™ FVD | ZombieUV™ | Biolegend | 423107 | 1:2000 |
| CD3 (all T cells) | mouse IgG1 k / PerCP | Biolegend | 344814 | 1:300 |
| CD4 (T helper cells) | Mouse Anti-Human / BUV563 | BD Biosciences | 750979 | 1:200 |
| CD8 (T killer cells) | mouse IgG1 k / BV 510™ | Biolegend | 300934 | 1:200 |
| CD14 (monocytes) | mouse IgG1 k / PerCP-Cy5.5 | Biolegend | 325622 | 1:500 |
| CD20 (B cells) | mouse IgG1 k / AF 700 | Biolegend | 302322 | 1:300 |
| CD45 (leukocytes) | mouse IgG1 k / BV570 | Biolegend | 304033 | 1:100 |
| CD56 (NK cells) | mouse IgG1 k / PE-Cy5 | Biolegend | 362515 | 1:200 |
| CD86 (APCs) | mouse IgG1 k / BV605 | Biolegend | 374231 | 1:100 |
| HLA-DR (APC) | mouse IgG1 k / APC-Cy7 | Biolegend | 307618 | 1:200 |
| IFN-γ | mouse IgG1 k / BV785 | Biolegend | 502542 | 1:80 |
| IL-6 | rat IgG1 k / PE-Dazzle™ 594 | Biolegend | 501122 | 1:100 |
| TNF-α | mouse IgG1 k / APC | Biolegend | 502912 | 1:100 |

Table S 1: Antibodies and FVD used in the PBMC assay with supplier information and dilution.

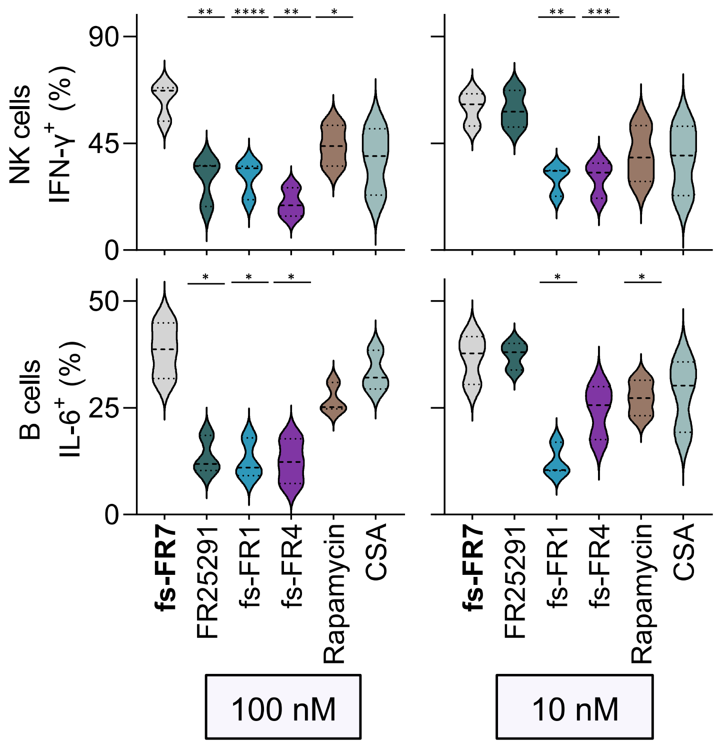


**Figure S1. Active FR molecules suppress cytokine production across human donors.** Violin plots (median +/- quartiles) showing cytokine expression of NK and B cells at indicated conditions (same assay setup as Fig 3A). Significance compared to fs-FR7 as inactive control (bold) is indicated, data represent 3 biological replicates (mean of 3 technical replicates each) per condition, p-value * < 0.05, ** < 0.01, *** < 0.001, **** < 0.0001, RM one-way ANOVA with Dunnett's multiple comparisons test.

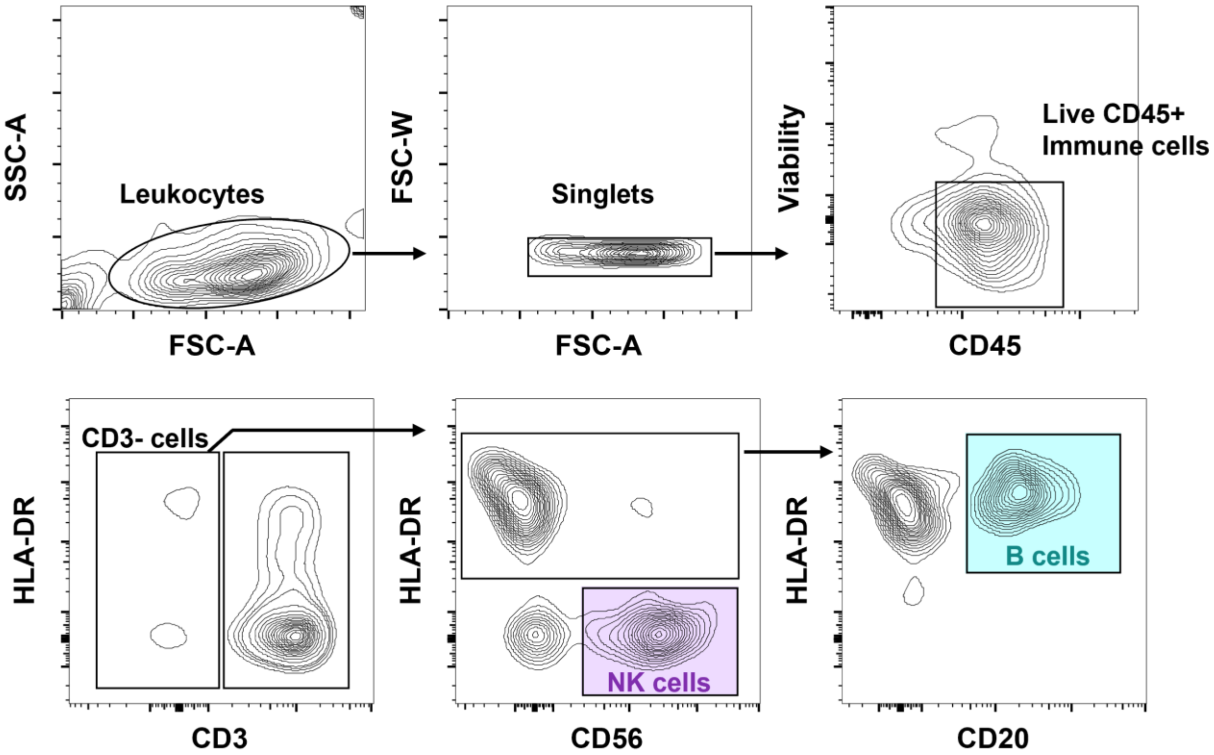


**Figure S2. Visualization of the Gating Strategy applied in the PBMC assays.** Surface marker-staining was used to determine cell type. CD45 served to select immune cells, which were divided into T cells (CD3+) and non-T cells (CD3–). Within the subset of CD3– non-T cells, cells were further divided into NK cells (CD56+ & HLA-DR–) and the remaining HLA-DR+ cells were divided to identify B cells based on CD20+.

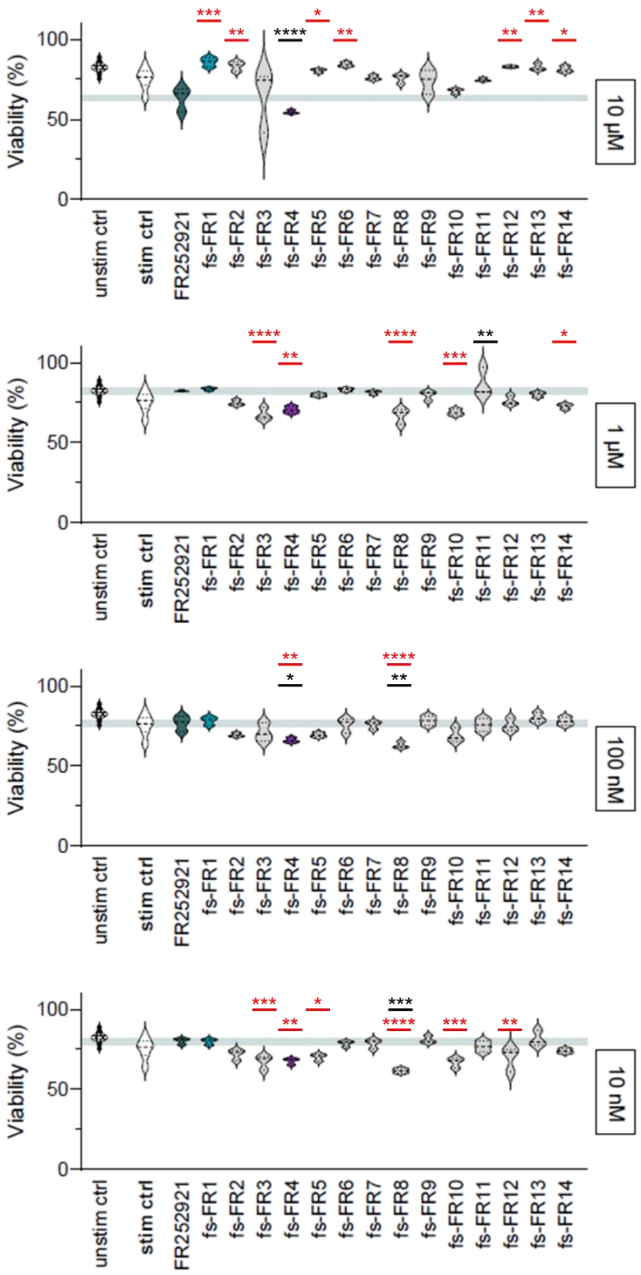


**Figure S3. Cell Viability under exposure to FR compounds and stimulation at a range of concentrations.** Violin plots (median +/- quartiles) showing cell viability of total PBMC at 10 µM, 1 µM, 100 nM and 10 nM under assay conditions. Significance compared to positive control (stimulated control (stim. ctrl)) is indicated; data represent three technical replicates per condition; p-value * < 0.05, ** < 0.01, *** < 0.001, **** < 0.0001; ordinary one-way ANOVA with Dunnett's multiple comparisons test; dark green line indicates mean value of (−)-FR252921; red indicates significant difference of a given compound to (−)-FR252921.

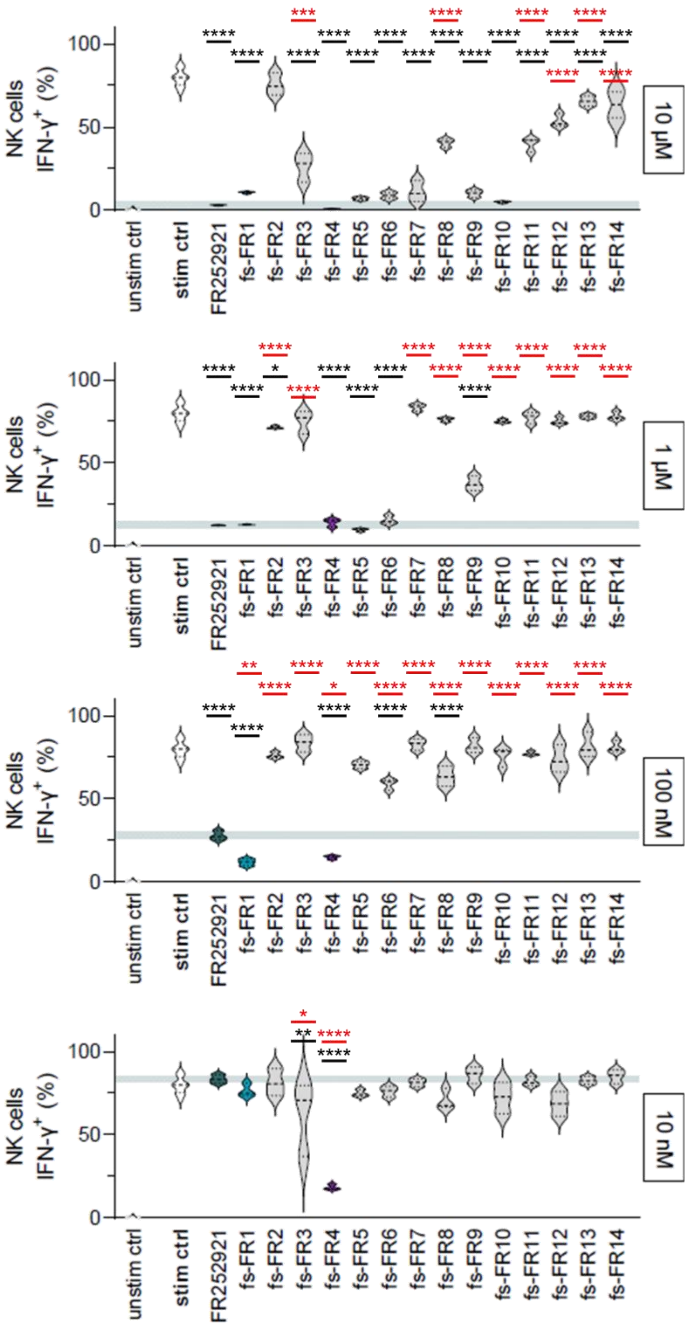


**Figure S4. Suppression of IFN-γ expression in NK cells by FR compounds.** Violin plots (median +/- quartiles) showing cytokine expression of NK cells at 10 µM, 1 µM, 100 nM and 10 nM under assay conditions. Significance compared to positive control (stimulated control (stim. ctrl)) is indicated; data represent three technical replicates per condition; p-value * < 0.05, ** < 0.01, *** < 0.001, **** < 0.0001; ordinary one-way ANOVA with Dunnett's multiple comparisons test; dark green line indicates mean value of (−)-FR252921; red indicates significant difference of a given compound to (−)-FR252921.

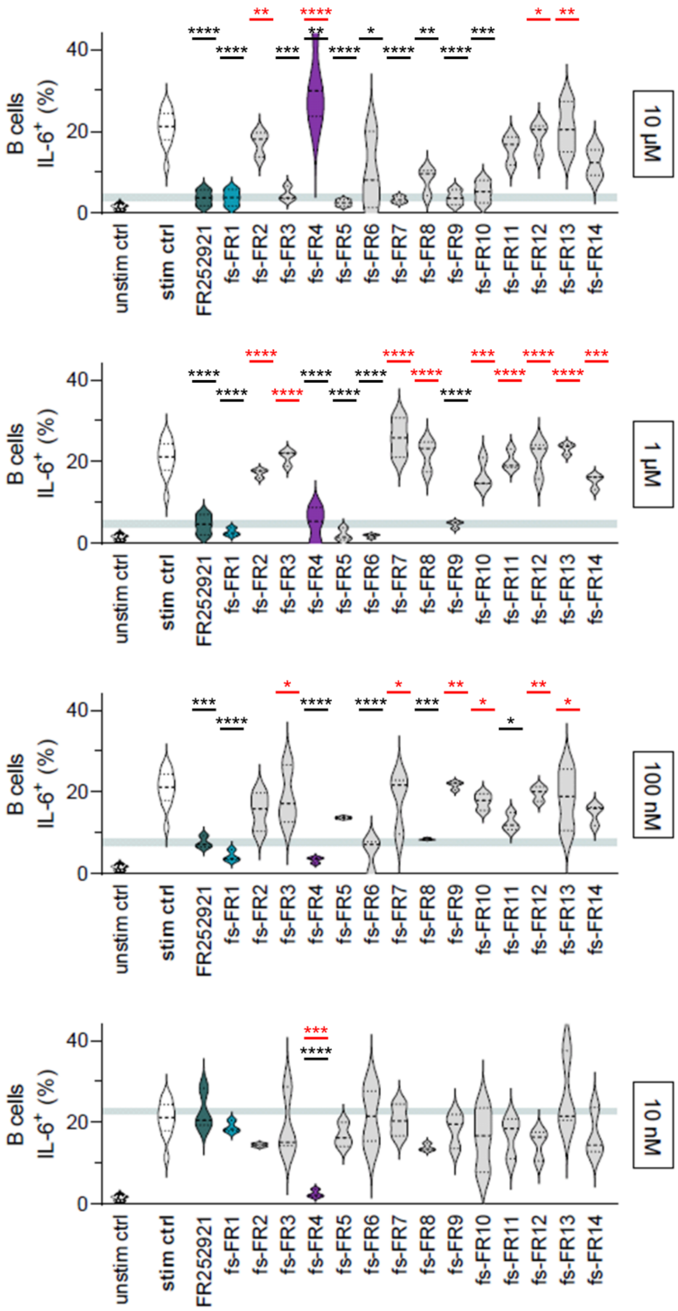


**Figure S5. Suppression of IL-6 expression in B cells by FR compounds.** Violin plots (median +/- quartiles) showing cytokine expression of B cells at 10 µM, 1 µM, 100 nM and 10 nM under assay conditions. Significance compared to positive control (stimulated control (stim. ctrl)) is indicated; data represent three technical replicates per condition; p-value * < 0.05, ** < 0.01, *** < 0.001, **** < 0.0001; ordinary one-way ANOVA with Dunnett's multiple comparisons test; dark green line indicates mean value of (−)-FR252921; red indicates significant difference of a given compound to (−)-FR252921.

## 7.2. Cytotoxicity evaluation against non-immune cells

To assess cytotoxicity of FR compounds against non-immune cells, we selected two immortalized cell lines derived from primary human skin tissue; human epidermal keratinocytes (NHEK-SVTERT3-5, CLHT-011-0026-5, Evercyte) and human dermal fibroblasts (HDF/TERT164, CHT-008-0164, Evercyte). Cells were seeded at ~70 % confluency in tissue-culture treated 24-well plates in KGM-2 (KGM-2 Media Kit, C-20111, Lonza) + 1 % Penicillin-Streptomycin (Gibco, 15140122) or DMEM (Gibco, 41966029) +10 % Fetal Bovine Serum (FBS) (Gibco, A5256801) + 1 % Penicillin-Streptomycin, respectively. Cells were incubated over night at 37 °C, 5 % CO2 to allow for adherence and the next day FR252921 or fs-FR4 were added at the indicated concentrations and incubated for 4 h and 24 h. After incubation, cells were washed and trypsinized (keratinocytes: 7 minutes of Trypsin-EDTA solution (LONCC-5012, Lonza) and stopped with Trypsin Neutralizing solution (LONCC-5002, Lonza); fibroblasts: 4 minutes of Trypsin-EDTA 0.05 % (25300054, Gibco) and stopped with 10 % FBS in DPBS). Cells were collected in tubes, washed and stained with Fixable Viability Dye eFluor 780 (Invitrogen, 65-0865-14) at 1:4000 in DPBS for 20 minutes on ice to label dead cells, subsequently fixed as described above and acquired on a BD FACS Aria III. Cells exposed to 25 % EtOH before trypsinization and untreated cells served as dead and live controls for gating respectively.

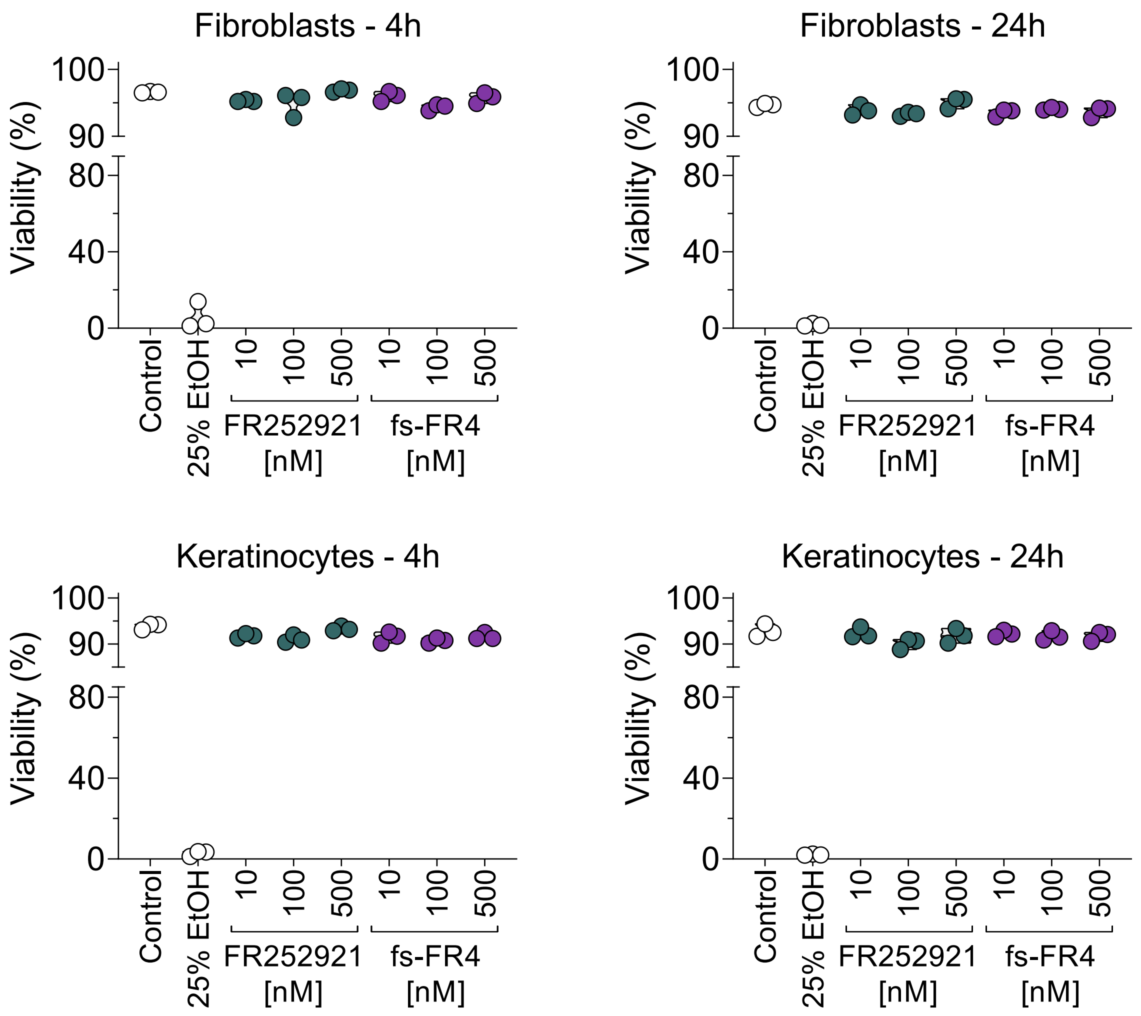


**Figure S6. Cell Viability Assay of immortalized primary skin-tissue derived cells against selected FR compounds.** Control refers to untreated cells (live control), whereas 25 %EtOH-treated cells served as control for cell death.

# 8. References

(1) Chen, Y.; Coussanes, G.; Souris, C.; Aillard, P.; Kaldre, D.; Runggatscher, K.; Kubicek, S.; Di Mauro, G.; Maryasin, B.; Maulide, N. A Domino 10-Step Total Synthesis of FR252921 and Its Analogues, Complex Macrocyclic Immunosuppressants. *J. Am. Chem. Soc.* **2019**, *141* (35), 13772. https://doi.org/10.1021/jacs.9b07185.
(2) Skaanderup, P. R.; Jensen, T. Synthesis of the Macrocyclic Core of (-)-Pladienolide B. *Org. Lett.* **2008**, *10* (13), 2821–2824. https://doi.org/10.1021/ol800946x.
(3) Petroski, R. J.; Vermillion, K.; Cossé, A. A. Two-Carbon Homologation of Aldehydes and Ketones to α,β-Unsaturated Aldehydes. *Molecules* **2011**, *16* (6), 5062–5078. https://doi.org/10.3390/molecules16065062.
(4) DeBoef, B.; Counts, W. R.; Gilbertson, S. R. Rhodium-Catalyzed Synthesis of Eight-Membered Rings. *J. Org. Chem.* **2007**, *72* (3), 799–804. https://doi.org/10.1021/jo0620462.
(5) Patel, B. A.; Kim, J.-I. I.; Bender, D. D.; Kao, L.-C.; Heck, R. F. Palladium-Catalyzed Three Carbon Chain Extension Reactions with Acrolein Acetals. A Convenient Synthesis of Conjugated Dienals. *J. Org. Chem.* **1981**, *46* (6), 1061–1067. https://doi.org/10.1021/jo00319a004.
(6) Kim, T.; Lee, S. I.; Kim, S.; Shim, S. Y.; Ryu, D. H. Total Synthesis of PGF2α and 6,15-Diketo-PGF1α and Formal Synthesis of 6-Keto-PGF1α via Three-Component Coupling. *Tetrahedron* **2019**, *75* (42), 130593. https://doi.org/10.1016/j.tet.2019.130593.
(7) Amans, D.; Bellosta, V.; Cossy, J. Synthesis of a Promising Immunosuppressant: FR252921. *Org. Lett.* **2007**, *9* (23), 4761–4764. https://doi.org/10.1021/ol702110k.
(8) Strobl, J.; Pandey, R. V.; Krausgruber, T.; Kleissl, L.; Reininger, B.; Herac, M.; Bayer, N.; Krall, C.; Wohlfarth, P.; Mitterbauer, M.; Kalhs, P.; Rabitsch, W.; Bock, C.; Hopfinger, G.; Stary, G. Anti-Apoptotic Molecule BCL2 Is a Therapeutic Target in Steroid-Refractory Graft-Versus-Host Disease. *J. Invest. Dermatol.* **2020**, *140* (11), 2188–2198. https://doi.org/10.1016/j.jid.2020.02.029.
